# A Unified Synthetic Approach to 2‑Alkyl Azetidines, Oxetanes, Thietanes and Cyclobutanes from Unactivated Alkenes

**DOI:** 10.1021/jacs.5c11758

**Published:** 2025-11-27

**Authors:** Louis Buck, Maria Pelosi, Subhasis Paul, Emilie Wheatley, Adam Richardson, Soumen Ghosh, Francesca Sardelli, Mattia Silvi

**Affiliations:** § The GSK Carbon Neutral Laboratories for Sustainable Chemistry, 6123 University of Nottingham, Jubilee Campus, Nottingham NG7 2TU, United Kingdom; ∥ School of Chemistry, University of Nottingham, University Park, Nottingham NG7 2RD, United Kingdom; ‡ Department of Pharmacy, University of Pisa, Via Bonanno Pisano 12, Pisa, 56126, Italy

## Abstract

2-Alkyl substituted four-membered carbocycles and heterocycles are relevant motifs in pharmaceuticals and agrochemicals. However, synthetic methods to access these scaffolds mostly rely on functional group interconversion of a narrow subset of preformed cyclic building blocks, impeding access to a broad chemical space. In this report, we introduce a general method to transform readily available unactivated alkenes into 2-alkyl four-membered rings. In contrast to classic [2 + 2] cycloaddition chemistry—which is not viable for this class of products—our strategy leverages a homologative alkene dielectrophilic activation, converting the starting alkene into a transient 1,3-iodo-sulfonium intermediate, which undergoes sequential nucleophilic substitutions to promote ring formation. The chemical versatility of the intermediate enables modulation of the order of substitutions, crucial to targeting the full matrix of four-membered rings—i.e., azetidines, oxetanes, thietanes, and cyclobutanes. This functional group tolerant process is applicable to a wide range of alkenes—including complex structures—opening new avenues for applications in pharmaceutical and agrochemical synthesis.

## Graphical abstract

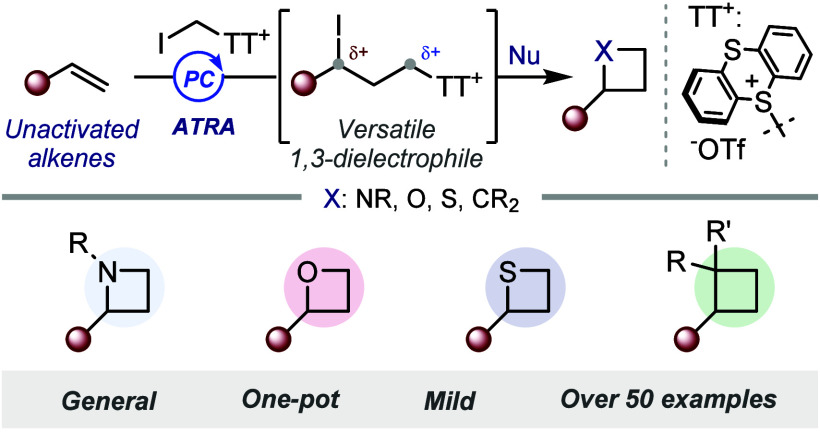

Four-membered carbo- and heterocycles are valuable scaffolds with distinctive structural features that render them highly attractive in medicinal chemistry.^1–6^ Their well-defined sp^3^-rich, conformationally rigid frameworks constitute a valuable tool to modulate target affinity and lipophilicity in drug design, and has been often associated with increased metabolic stability compared to other cyclic analogs.^1^ Among various substitution patterns, 2-alkyl substituted four-membered carbo- and heterocycles represent privileged motifs found in numerous pharmaceuticals, drug candidates, and agrochemicals (Scheme 1a).^7^ Despite their prominence, access to these scaffolds remains challenging and largely limited to classical functional group interconversion (FGI) strategies from a restricted pool of commercially available preformed cyclic building blocks (i.e., **1**). As a result, the structural diversity of products is severely constrained, precluding access to large regions of chemical space. This is undesirable, since structural diversification of the 2-substituent has been shown to be significant in various medicinal chemistry applications.^8^ However, access to relevant compounds not immediately obtainable from available building blocks is challenging, often requiring long multistep synthetic sequences and expensive starting materials (e.g., **2** and **3**, Scheme 1b).^8a,8b^

**Scheme 1:**
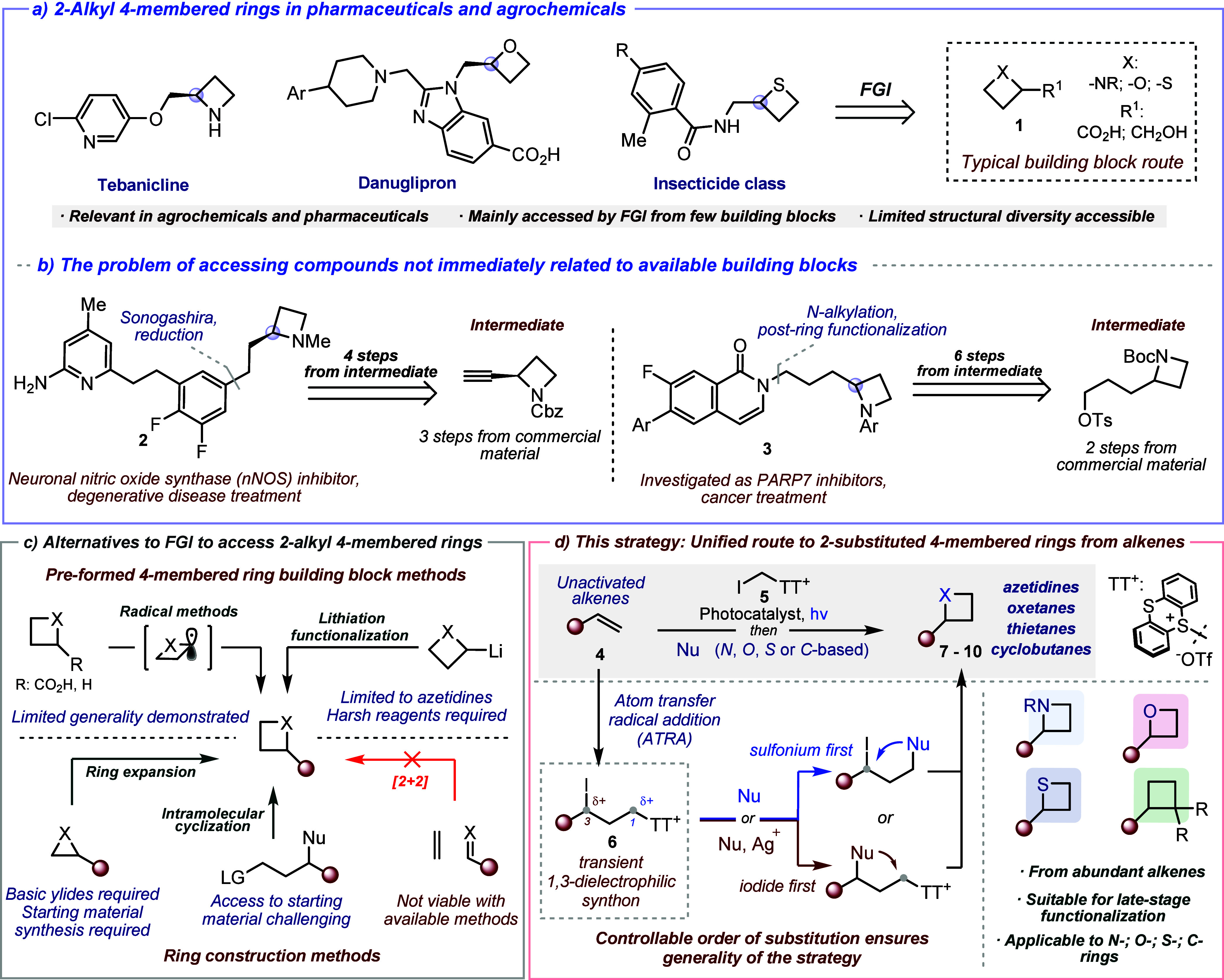
a) Relevant 2-Alkyl Four-Membered Heterocycles; b) Relevance and Synthetic Challenges in the Diversification of 2-Substitution; c) Current Routes to 2-Alkyl Four-Membered Rings; d) This Strategy

Thus, alternative methods have been investigated to enable modular access to 2-alkyl substituted four-membered rings from available preformed cyclic building blocks, including lithiation-functionalization^9^—limited to azetidines^10^ and requiring harsh reagents—and radical methods (Scheme 1c, top).^11–13^ However, the latter have been mostly applied to structurally simple entries and cannot be extended to all classes of four-membered rings. Systematic studies in this regard are rare, and limited to accessing Giese or styrene addition products.^14^ Furthermore, products are mostly obtained in moderate yields, widely reflecting the challenges of taming reactive four-membered cyclic radicals, expected to be more strained and less stabilized than other carbon-centered radicals.^15^

Alternative synthetic strategies to access 2-alkyl substituted four-membered rings involve construction of the cyclic core (Scheme 1c, bottom) via ring expansions^16^ and intramolecular cyclizations.^17^ However, such strategies typically require highly basic reagents and bespoke starting materials, which may be challenging or labor-intensive to access when structures of moderate complexity are involved. Although [2 + 2] cycloaddition chemistry offers a powerful tool to assemble four-membered rings from readily available alkene precursors,^18,19^ four-membered ring heterocycles bearing only a 2-alkyl substituent are outside the scope of such strategy, likely due to the challenges of engaging ethylene or formaldehyde-related unsaturated systems in this reactivity.

Our group recently disclosed a novel homologative dielectrophilic activation of olefins,^20^ extending the established ability of thianthrenium salts to activate double bonds.^21^ This strategy enables the conversion of a simple, unactivated alkene into an elusive formal 1,3-dicationic synthon (intermediate **6**, Scheme 1d), thereby granting access to unconventional disubstitution patterns.^22^ Building on our interest in accessing small rings from readily available materials,^23^ we now report how this powerful activation mode can be harnessed to provide a unified strategy for synthesizing 2-alkyl-substituted azetidines, oxetanes, thietanes, and cyclobutanes from abundant olefins—offering a general solution to the synthetic challenges outlined above. Our strategy, depicted in Scheme 1d, involves reacting the unactivated alkene **4** with iodomethyl thianthrenium salt **5** ^20,24^ via photocatalytic atom-transfer radical addition (ATRA).^25^ Treatment of the resulting intermediate **6** with suitable *N, O, S*, or *C*-nucleophiles promotes ring formation (**7**–**10**) upon a domino-type double substitution process. The exceptional versatility of intermediate **6**, stemming from the distinct chemical nature of the iodide and sulfonium centers, allows control of the substitution sequence, which proved to be essential for achieving generality in our strategy (Scheme 1d, bottom).

Our study began by investigating the formation of both azetidines and oxetanes. The envisioned reaction protocol involved exposure of an acetonitrile mixture of model alkene **4a**, iodomethylsulfonium triflate **5** and photocatalyst 4CzIPN^26^ to blue light irradiation under our previously optimized reaction conditions,^20^ followed by *in situ* addition of a suitable nucleophile **11a**–**g** and base to the unhandled vessel to promote four-member ring formation (Table 1). Using a mixture of *tert*-butyl carbamate **11a** or tosylamine **11b** and potassium *tert*-butoxide did not lead to the desired azetidine formation, and alkene byproducts were instead observed due to competing elimination (Table 1, entry 1). Using a mixture of benzylamine **11c** and 2,6-lutidine as a proton scavenger afforded the desired azetidine, albeit in 30% yield (entry 2) and accompanied by unselective elimination side-pathways. Optimizing the benzylamine stoichiometry and replacing 2,6-lutidine with K_3_PO_4_ improved the results, leading to the desired product in 61% yield (entry 3, full optimization in Supprting Information (SI)). Using the less basic *para*-methoxy aniline **11d** completely inhibited E2 elimination side pathways, forming the product in 43% yield (entry 4) with an uncyclized intermediate remaining. Conversion was improved upon adding DMPU as a cosolvent after irradiation and sodium bicarbonate as a base, affording azetidine **7a** in 83% yield (entry 5, full optimization in SI). Attempts to access oxetanes using OH^–^ as a nucleophilic oxygen source were unsuccessful. Therefore, an alternative strategy was attempted using an oxygen-based nucleophile able to generate a labile intermediate, followed by *in situ* addition of base (KO^*t*^Bu) to promote hydrolysis and cyclization to oxetane. The use of silanolate **11e** was unsuccessful (entry 6), leading to a complex mixture of alkene byproducts. Although the less basic sodium trifluoroacetate **11f** also failed to provide the desired oxetane (entry 7), exchanging the counterion to silver trifluoroacetate was observed to have a striking effect on the reaction outcome, leading to the desired oxetane **8a** in 36% yield (entry 8). The yield was improved to 62% upon tuning the standard reaction parameters and adding *tert*-amyl alcohol as a cosolvent for the hydrolysis/cyclization (entry 9, full optimization in SI).

**Table 1:**
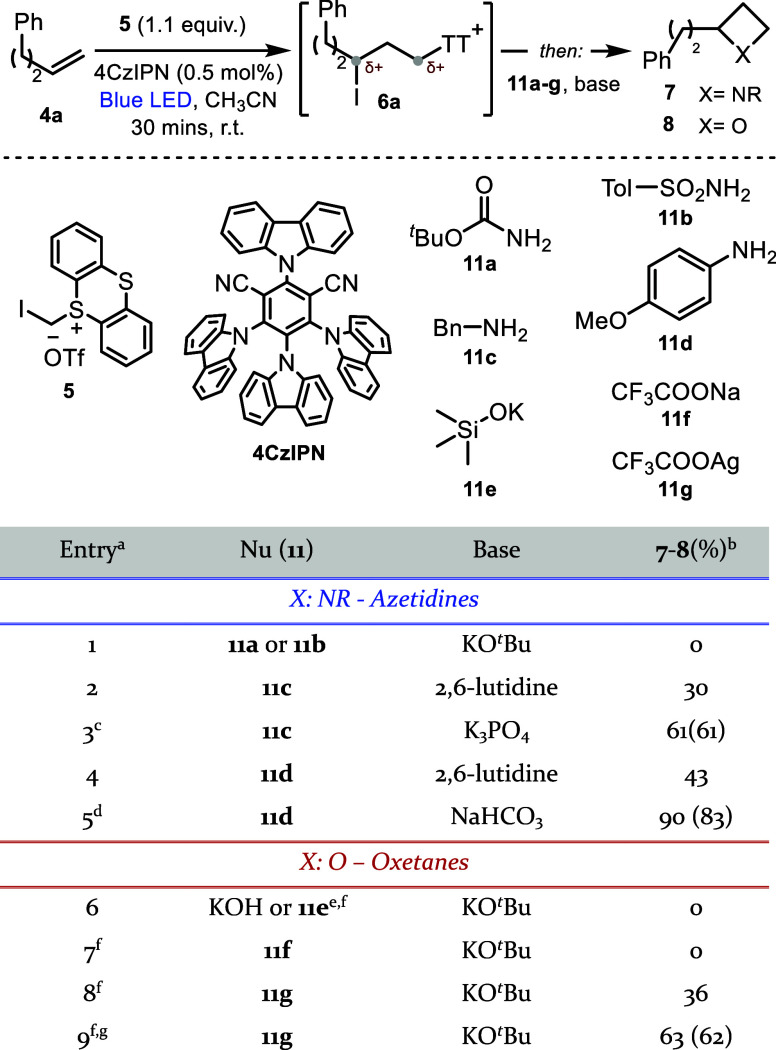
Optimization Studies

a Reactions on a 0.05 mmol scale, using 2–3 equiv of nucleophiles **11**, and 3–4 equiv of bases; see SI for full details.

b NMR yields, in parentheses, yield of isolated material of a 0.2 mmol scale reaction.

c After nucleophile and base addition, reaction stirred at 50 °C for 16 h.

d DMPU was added as a cosolvent with the nucleophile (CH_3_CN:DMPU 1:4), and the reaction was stirred for 16 h at 50 °C.

e Generated *in situ* from TMSOH and KO^*t*^Bu.

f KO^*t*^Bu was added sequentially after the *O*-nucleophile.

g Prior to base addition, ^*t*^AmylOH was added as a cosolvent (CH_3_CN:^*t*^AmylOH 1:4); see SI for full conditions details.

With the optimized conditions in hand, we investigated the scope of our process varying the alkene substrate. Azetidines containing synthetically versatile nitriles, esters, and amides (**7b**–**7d**) can be effectively accessed through our reaction protocol. Remarkably, free carboxylic acids, primary amines, and alcohols are well tolerated by the process, affording azetidines **7e**–**7g** in good yields and suggesting broad applicability of the method without the need for extensive protecting group strategies. Alkenes bearing alcohol functionalities closer to the reaction center afforded products in lower yields (**7h**–**7i**) due to undesired *O*-cyclization, which can be inhibited upon *O*-TBS protection (**7j**–**7k**). A primary alkyl chloride, with versatile functionality susceptible to S_N_2 reactivity, can also be included in the starting material structure, leading to product **7l**. Oxidizable benzyl ether and sulfide groups can be included in our alkene, leading to products (**7m**–**7n**) in good yields. The presence of versatile sulfones, phosphonates, and boronic esters does not hamper the reactivity, leading to azetidines **7o**–**7q**. A variety of unsaturated functionalities are tolerated by the process, including alkynes, Michael acceptors, and pyridines (**7r**–**7t**). Activated alkenes led to reduced yield, with styrene affording **7u** in 47% yield and ethyl acrylate leading to poor yield in a complex mixture. Structurally complex azetidines (**7v**–**7y**) can be accessed from the corresponding alkenes, demonstrating the remarkable functional group tolerance of the method.

A variety of anilines and heteroaromatic amines undergo the desired process (**7a**, **7aa**–**7ad**), with electron-rich systems outperforming electron-poor systems. Alkylamines also afforded the corresponding azetidines **7ae**–**7aj**, with comparable yields for linear and branched systems, suggesting tolerance to sterics.

Importantly, mild PMP-deprotection of model azetidines **7a**, **7d** and **7g**,^27^ furnished azetidines **7ba**–**7bc** (Scheme 2, bottom).

**Scheme 2:**
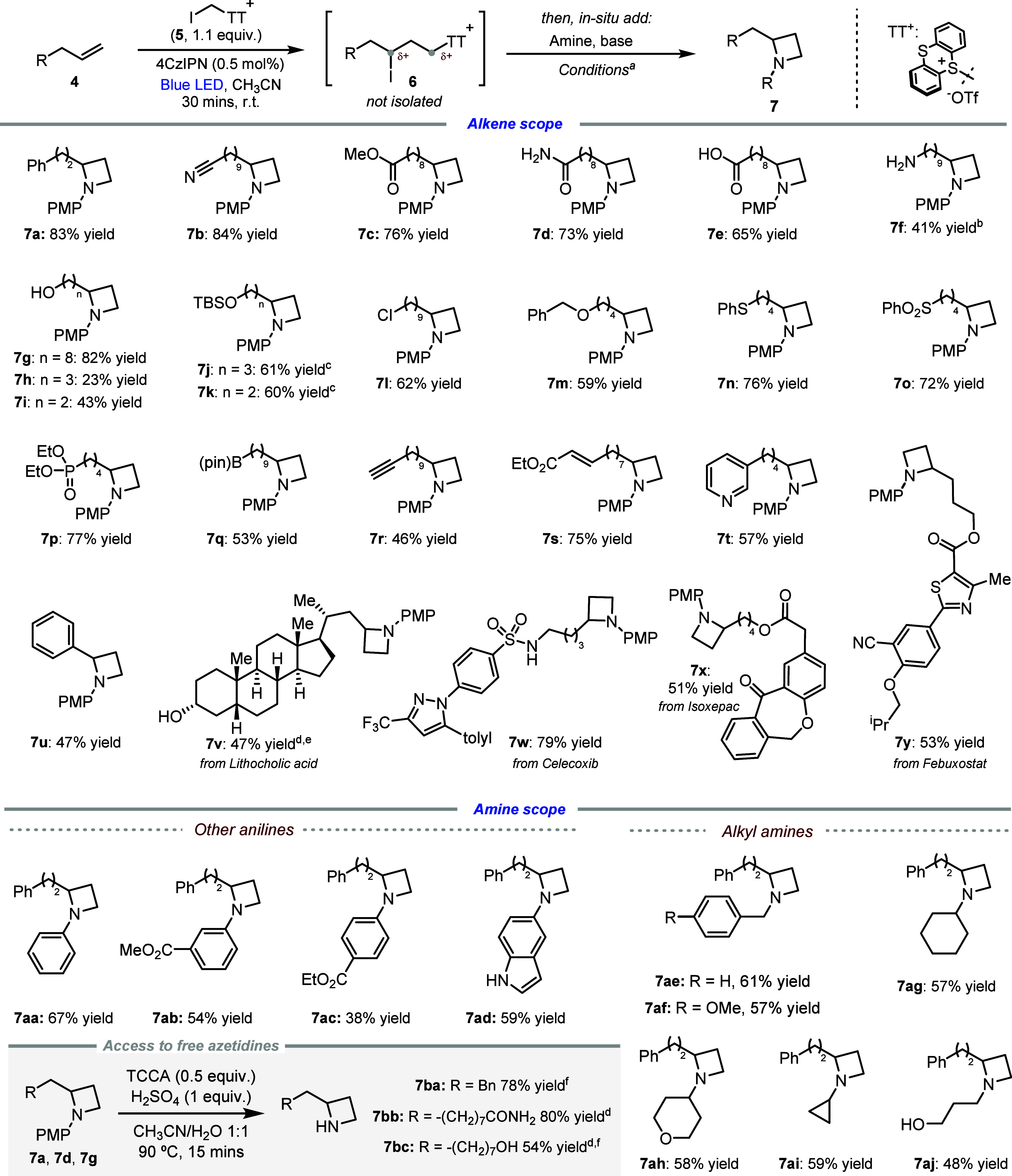
Scope of Azetidines

Reactions performed on a 0.2 mmol scale. Yields of isolated materials. Solvent mixtures resulting from cosolvent addition to the original CH_3_CN mixture. See SI for full experimental details and variations from standard procedures.
a Azetidines from anilines: Ar-NH_2_ (2.0–5.0 equiv), NaHCO_3_, DMPU/CH_3_CN 4:1, heating, 16 h; Azetidines from alkyl amines: Alkyl-NH_2_ (3.0–4.0 equiv), K_3_PO_4_, CH_3_CN, 50 °C, 16 h.
b CF_3_CO_2_H (1 equiv) added prior irradiation.
c 2,6-Lutidine (20 mol %) added prior to irradiation.
d 0.1 mmol scale
e 3:1 d.r.
f Isolated as the trifluoroacetate salt. TCCA: Trichloroisocyanuric acid.

We then explored the generality of this synthetic strategy to access other classes of four-membered rings (Scheme 3). Oxetanes bearing common and synthetically versatile phenyl, ester, nitrile, sulfone, and ether functionalities and even a complex Celecoxib derivative were obtained in moderate to good yields (**8a**–**8f**). Using potassium thioacetate/K_2_CO_3_ analogously enabled direct access to thietanes (**9a**–**9e**; see SI for optimization studies), motifs which are gaining increasing attention in medicinal chemistry and agrochemistry.^4^ Finally, addition of active methylene compounds and base to the reaction mixture immediately after irradiation afforded a variety of cyclobutanes, including pharmaceutically relevant spiro-oxindoles and spiro-lactones (**10a**–**10j**; see the SI for optimization studies). Krapcho decarboxylation of **10a** ^28^ afforded cyclobutane building block **10k**.

**Scheme 3:**
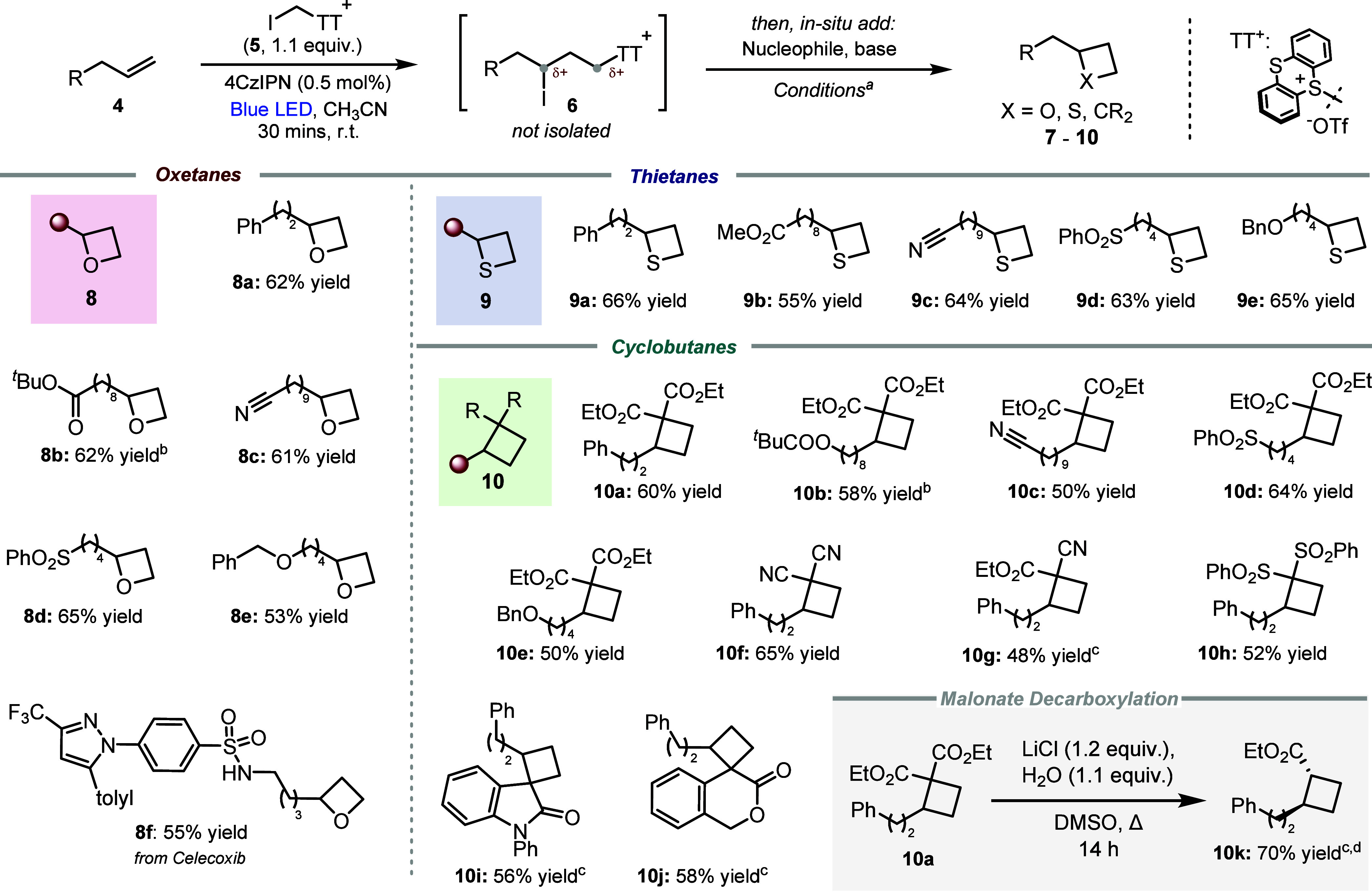
Scope of Oxetanes, Thietanes and Cyclobutanes

Reactions performed on a 0.2 mmol scale, yields of isolated materials. Solvent mixtures resulting from cosolvent addition to the original CH_3_CN mixture or solvent exchange; full details in SI.
a Oxetanes: AgO_2_CCF_3_ (2.0 equiv), −10 °C, 16 h, then KO^*t*^Bu, ^*t*^AmylOH/CH_3_CN 4:1, r.t., 1 h; Thietanes: KSOCCH_3_ (1.2 equiv), r.t., 1.5 h, then K_2_CO_3_, THF/MeOH 3:1, 40 °C, 18 h; Cyclobutanes: (CO_2_Et)_2_CHNa (3.0 equiv), THF/CH_3_CN 3:1, 0 to 60 °C, 18.5 h or pronucleophile (3.0 equiv), K_3_PO_4_, CH_3_CN, 60 °C, 24 h.
b 2,6-Lutidine (20 mol %) added prior irradiation.
c Products formed in 1:1–2:1 d.r.
d 0.1 mmol scale.

Control mechanistic experiments revealed the importance of the distinct chemical nature of the iodine and sulfonium centers within ATRA intermediate **6** in terms of the generality of the process.

A time-course study of azetidine **7a** formation, using both ^1^H NMR and HRMS analysis (Scheme 4a), unambiguously confirmed the expected initial attack of the aniline nucleophile on the primary alkyl sulfonium to give int. **I**, followed by 4-*exo*-*tet* cyclization^29^ to afford azetidine **7a**. Further experiments then highlighted the different behavior observed with *O*-nucleophiles, and the unique role played by the silver counterion of AgO_2_CCF_3_ in promoting oxetane formation (Scheme 4b).

**Scheme 4:**
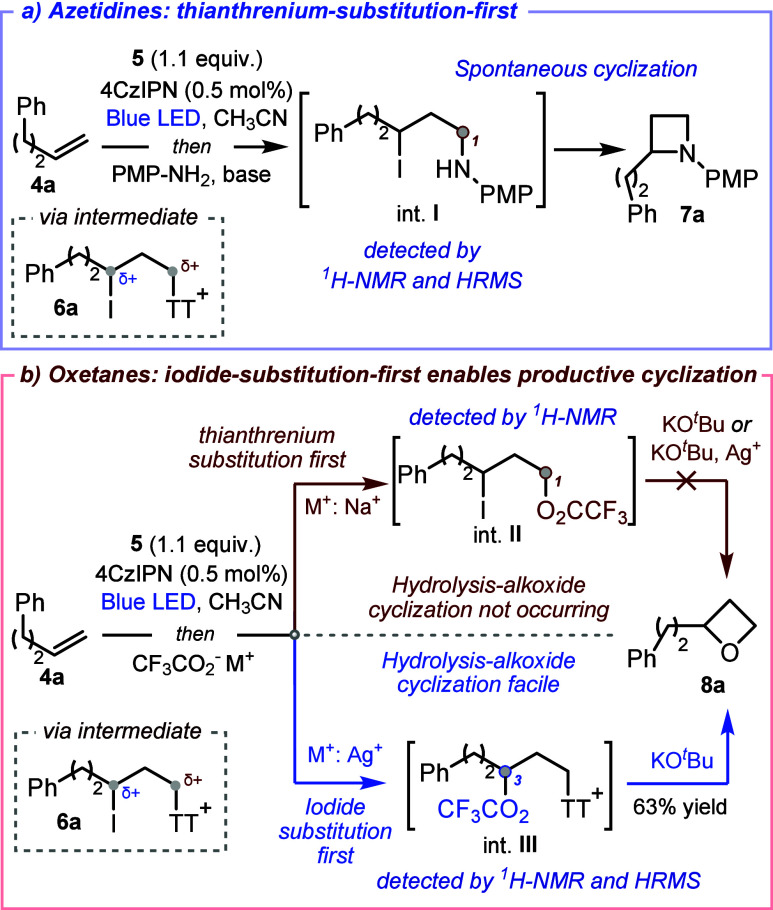
Mechanistic Insights

When NaO_2_CCF_3_ was used as the nucleophile, intermediate **II** was exclusively detected in the reaction mixture by ^1^H NMR analysis (Scheme 4b, top; see SI for details). However, upon the addition of KO^*t*^Bu to promote cyclization, no formation of oxetane **8a** was observed; instead, elimination byproducts were detected. A similar outcome was obtained when AgOTf was added to the basic cyclization mixture, indicating that silver cannot promote cyclization of intermediate **II**, and suggesting the likely involvement of a distinct intermediate species when AgO_2_CCF_3_ is used as a nucleophile. Consistent with this hypothesis, when AgO_2_CCF_3_ was used as a nucleophile, intermediate **II** was not detected in the crude reaction mixture. Instead, intermediate **III** was exclusively observed by ^1^H NMR and HRMS (Scheme 4b, bottom; see SI for details). *In situ* addition of KO^*t*^Bu to this mixture successfully promoted cyclization to afford oxetane **8a** in 63% NMR yield. These experiments highlight that the silver activation of the iodide center switches the natural order of nucleophilic substitution in ATRA intermediate **6a**.^30,31^ The resulting intermediate (**III**) undergoes facile cyclization, as it involves substitution of a highly reactive primary sulfonium. Collectively, the studies above highlight the versatility of iodo-sulfonium ATRA intermediate **6**, bearing two chemically distinct leaving groups, which can be selectively substituted upon tuning the reaction conditions.

In conclusion, we have developed a general and unified synthetic strategy for accessing 2-alkyl azetidines, oxetanes, thietanes, and cyclobutanes from readily available unactivated alkenes. The broad applicability of this method stems from the highly programmable reactivity of an iodo-sulfonium ATRA intermediate, formed via reaction of alkenes with an iodomethyl thianthrenium salt under visible-light photocatalysis. The wide substrate scope and functional group tolerance of this approach are anticipated to significantly extend the structural diversity of products accessible, offering new opportunities for the design and development of pharmaceuticals and agrochemicals.

## Supplementary Material



## Notes

Additional experimental details, materials, and methods, including photographs of experimental setup and spectral data for all compounds (PDF)

## Notes

The authors note that a related report has been recently deposited in a preprint server.^31^
