# Supplementary material for: A Unified Synthetic Approach to 2‑Alkyl Azetidines, Oxetanes, Thietanes and Cyclobutanes from Unactivated Alkenes
Source: J Am Chem Soc. 2025 Nov 27;147(49):44652–60. doi: 10.1021/jacs.5c11758 (PMC12703670; doi:10.1021/jacs.5c11758)

Supporting Information for

A Unified Synthetic Approach to 2-Alkyl Azetidines, Oxetanes, Thietanes and Cyclobutanes from Unactivated Alkenes

Louis Buck,[§†] Maria Pelosi,[§†] Subhasis Paul,[§†] Emilie Wheatley,[§†] Adam Richardson,[§†] Soumen Ghosh,[§†] Francesca Sardelli,[‡] and Mattia Silvi[§†]*

[§]*The GSK Carbon Neutral Laboratories for Sustainable Chemistry, University of Nottingham, Jubilee Campus, Nottingham NG7 2TU, United Kingdom;*
[†]*School of Chemistry, University of Nottingham, Nottingham, NG7 2RD, United Kingdom;*
[‡] *Department of Pharmacy, University of Pisa, Via Bonanno Pisano 12, Pisa, 56126, Italy.*
Correspondence to: mattia.silvi@nottingham.ac.uk

Table of Contents

1.1. Solvents, reagents, and starting materials 2
1.2. Chromatography and instrumental analysis 2
1.3. Naming of compounds 2
1.4. LEDs 3
2. Synthesis of novel starting materials 3
2.1. Synthesis of 5-(iodomethyl)-5H-thianthren-5-ium triflate (5) 3
2.2. Synthesis of dodec-11-enenitrile (4b) 4
2.3. Synthesis of 4,4,5,5-tetramethyl-2-(undec-10-en-1-yl)-1,3,2-dioxaborolane (4q) 4
2.4. Synthesis of (3*R*,8*R*,9*S*,10*S*,13*R*,14*S*,17*R*)-10,13-dimethyl-17-((*R*)-pent-4-en-2-yl)hexadecahydro-1*H*-cyclopenta[*a*]phenanthren-3-ol (4v) 5
3. Optimization studies 7
3.1. Azetidines 7
3.2. Oxetanes 10
3.3. Thietanes 11
3.4. Cyclobutanes 13
4. General procedures & product characterization 16
4.1. Reaction set-up for irradiation of mixtures with blue LEDs 21
4.2. Reaction products and characterization 22
5. Mechanistic Insights 62
5.1. Mechanistic insights into azetidine formation 62
5.2. Mechanistic insights into oxetane formation 63
6. References and Notes 68
7. NMR Spectra 69

1.1. Solvents, reagents, and starting materials

All air and water-sensitive reactions were carried out in oven-dried glassware under argon atmosphere using standard Schlenk manifold technique. The solvents were degassed when needed by bubbling argon for ten minutes. Bulk solutions were evaporated under reduced pressure using a Büchi rotary evaporator. All solvents were commercially supplied or provided by the communal stills of the School of Chemistry, University of Nottingham. Commercially available compounds were purchased from Sigma Aldrich, Alfa Aesar, Acros, Fluorochem, TCI chemicals and used as received. Dried solvents were purchased from Acros Organic, Extra Dry over molecular sieves, AcroSeal®.

Compounds **4d**,[1] **4j**,[2] **4k**,[3] **4l**,[4] **4n**,[5] **4p**,[6] **4q**,[7] **4r**,[8] **4s**,[9] **4t**,[10] **4w**,[11] **4x**,[11] and **4y**,[11] and **4z**,[12] were synthesized following reported procedures.

1.2. Chromatography and instrumental analysis

Flash column chromatography (FCC) was carried out using Sigma-Aldrich silica gel LC60A-40 (63 μm). THF and Et_2O were distilled prior to use. All reactions were followed by thin-layer chromatography (TLC) when practical, using Merck Kieselgel 60 F_{254} fluorescent treated silica which was visualised under UV light, by staining with aqueous basic potassium permanganate, phosphomolybdic acid or with ninhydrin solution.

^{1}H-NMR, **^{13}C-NMR** and **^{19}F-NMR** spectra were recorded using Bruker broadband prodigy cryoprobe AV(III)500HD 500 MHz and Bruker AV(III)400HD 400 MHz spectrometers. $CDCl_3$ was neutralized upon passing through a short path of basic alumina. Chemical shifts (δ) are given in parts per million (ppm) and coupling constants (*J*) are given in hertz (Hz). The ^{1}H-NMR spectra are reported as follows: ppm (multiplicity, coupling constants, number of protons). High resolution mass spectra (**HRMS**) were recorded on a Bruker MicrOTOF II by Electrospray Ionisation (ESI) or on a Jeol AccuTOF GCx by Electron Ionisation (EI). **IR** spectra were recorded on a Bruker Vertex 70 FT-IR ATR as a thin film. Only selected absorption maxima (ν_{max}) are reported in wavenumbers (cm^{-1}). **Melting points** were recorded in degrees Celsius (°C), using a Stuart melting point SMP 20 microscope apparatus and are reported uncorrected. Optical rotation (**$[\alpha]_D^{25}$**) was recorded on an Anton Paar MCP 100 at 25 °C, in chloroform, with a concentration of 1mg/1mL. **Fluorescence spectra** were recorded using a PerkinElmer LS 55 Luminescence spectrometer. **Cyclic voltammetry** experiments were carried out using a EmStat 4s potentiostat. The **UV/Vis absorbance** study were recorded by Agilent Cary 5000 UV-Vis-NIR spectrophotometer.

1.3. Naming of compounds

Compound names are generated by ChemDraw 20.0 software (PerkinElmer), following the IUPAC nomenclature.

1.4. LEDs

Irradiation of reaction mixtures was performed using Kessil lamp A160WE Tuna Blue Saltwater LED Light 40W set to blue at maximum intensity. Lamp emission is observed to decay over long usage due to LED chip deterioration. To ensure good reproducibility of the results, we recommend using new lamps.

2. Synthesis of novel starting materials

2.1. Synthesis of 5-(iodomethyl)-5H-thianthren-5-ium triflate (5)

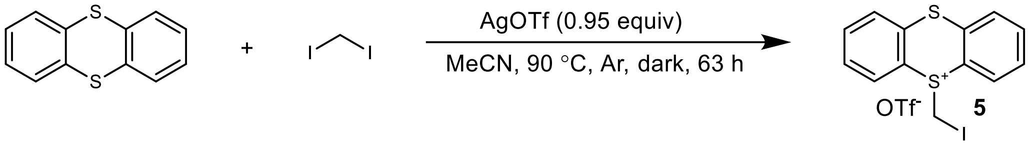

A two-neck 250 mL oven-dried round-bottom flask with atmosphere exchanged to argon was charged sequentially with thianthrene (1.0 equiv., 40.0 mmol, 8.7 g), AgOTf (0.95 equiv., 38.0 mmol, 9.8 g), MeCN (0.7M, 60 mL) and diiodomethane (1.0 equiv., 40.0 mmol, 3.3 mL). A reflux condenser pre-purged with argon was fitted to the primary neck of the flask, and the reaction mixture placed in a pre-heated oil bath at 90 °C for 63 h with sufficient covering using aluminium foil to maintain the dark condition. After completion, the reaction was left to cool to room temperature and filtered through a thick pad of celite, washing with MeCN. The filtrate was concentrated under reduced pressure and the resulting pale-yellow solid redissolved in dichloromethane using a minimum of MeCN to aid dissolution. Celite was added and the solvent removed once again under reduced pressure. The resulting powder was dry-loaded and purified by column chromatography (SiO_2; 95:5 DCM:MeOH) to afford **5** (13.2 g, 65%) as a brilliant white solid. **R_f** (95:5 DCM:MeOH) 0.23; **M.P.**: 180.2–183.7 °C; **^{1}H NMR** (CD_3CN, 500 MHz) δ (ppm): 8.15 (dd, *J* = 8.0, 1.4 Hz, 2H), 7.98 (dd, *J* = 8.0, 1.2 Hz, 2H), 7.87 (td, *J* = 8.0, 1.3 Hz, 2H), 7.74 (td, J = 8.0 Hz, 2H), 4.86 (s, 2H).; **^{13}C NMR** (CD_3CN 101 MHz) δ (ppm): 136.4, 136.2, 135.9, 131.1, 130.4, 118.5, 0.2; **^{19}F NMR** (CD_3CN 376 MHz) δ (ppm): 79.2 (s, 3F); **HRMS** (ESI-TOF) mass calculated for [M]+ $(C_{13}H_{10}IS_2)^+$ expected at m/z 356.9264; found m/z 356.9265.

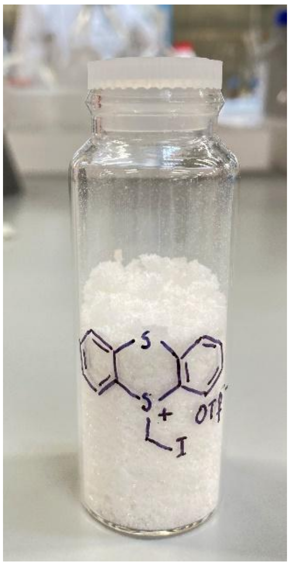

Figure S1: compound **5**

2.2. Synthesis of dodec-11-enenitrile (4b)

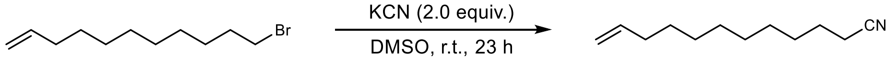

1.0 equiv. **4b**

To an oven dried 25 mL RBF, KCN (2.0 equiv., 2.0 mmol, 131 mg) was introduced first followed by dry DMSO (10 mL) and 11-bromoundec-1-ene (1.0 equiv., 1.0 mmol, 220.0 µL) at room temperature. The mixture was stirred at room temperature for 23 h. EtOAc (100 mL) was then added to the mixture and the organic layers were washed with water (4 × 30 mL)) and once with brine (20 mL). The organic solvent was dried over anhydrous Na_2SO_4, filtered and concentrated under reduced pressure to afford compound **4b** (178.3 mg, 99%) as a transparent oil. **R_f** (19:1 hexane:EtOAc) 0.55; **IR** (film) ν_{max}/cm^{-1}: 3077, 2920, 2855, 2246, 1640, 1464, 1428, 994, 910, 723, 634; **^{1}H NMR** ($CDCl_3$, 500 MHz) δ (ppm): 5.88 – 5.75 (m, 1H), 5.04 – 4.89 (m, 2H), 2.33 (t, *J* = 7.1 Hz, 2H), 2.07 – 2.01 (m, 2H), 1.69 – 1.61 (m, 2H), 1.48 – 1.41 (m, 2H), 1.41 – 1.34 (m, 2H), 1.33 – 1.26 (m, 8H); **^{13}C NMR** ($CDCl_3$, 126 MHz) δ (ppm): 139.3, 120.0, 114.3, 33.9, 29.5, 29.4, 29.2, 29.0, 28.9, 28.8, 25.5, 17.3; **HRMS** (ESI-TOF) mass calculated for $[M+Na]^+$ $(C_{12}H_{21}NNa)^+$ expected *m/z* 202.1566; found *m/z* 202.1564.

2.3. Synthesis of 4,4,5,5-tetramethyl-2-(undec-10-en-1-yl)-1,3,2-dioxaborolane (4q)

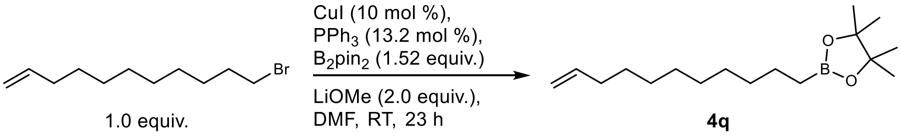

1.0 equiv. **4q**

4,4,5,5-tetramethyl-2-(undec-10-en-1-yl)-1,3,2-dioxaborolane, **4q**, was synthesised through a modification of a known procedure.[7]

In air, CuI (0.19 g, 1.0 mmol, 10 mol %), PPh_3 (0.35 g, 1.32 mmol, 13.2 mol %), LiOMe (0.76 g, 20.0 mmol, 2.0 equiv.), and bis(pinacolato)diboron (3.9 g, 15.2 mmol, 1.52 equiv.) were added to a Schlenk tube equipped with a stirrer bar. The vessel was evacuated and backfilled with argon (three cycles). DMF (20 mL) and 11-bromoundec-1-ene (1.7 mL, 10.0 mmol, 1.0 equiv.) were added in turn by syringe under an argon atmosphere. The resulting reaction mixture was stirred vigorously at 25 ºC for 23 h. The reaction mixture was then diluted with EtOAc, filtered through silica gel with copious washings (EtOAc) and concentrated under reduced pressure. The DMF was removed by diluting the residue with EtOAc and washing with H_2O (4x40 mL) followed by brine (ca. 100 mL). The organics were collected, dried over anhydrous Na_2SO_4, filtered and concentrated under reduced pressure. The residue was purified by column chromatography (SiO_2; gradient 65:35 hexane:DCM to 60:40

hexane:DCM) to afford **4m** (0.76 g, 27%) as a colourless oil. **R**$_f$ (60:40 hexane:DCM) 0.37; **IR** (film) ν_{max}/cm^{-1}: 3076, 2978, 2924, 2854, 1641, 1465, 1408, 1377, 1318, 1271, 1215, 1146, 1112, 992, 968, 909, 881, 847, 721, 672, 577, 545; **^{1}H NMR** ($CDCl_3$, 500 MHz) δ (ppm): 5.81 (ddt, *J* = 16.9, 10.2, 6.7 Hz, 1H), 5.02 – 4.90 (m, 2H), 2.03 (qt, *J* = 6.9, 1.3 Hz, 2H), 1.44 – 1.32 (m, 4H), 1.26 (s, 10H), 1.24 (s, 12H), 0.76 (t, *J* = 7.8 Hz, 2H); **^{13}C NMR** ($CDCl_3$, 101 MHz) δ (ppm): 139.4, 114.2, 83.0 (2C), 34.0, 32.6, 29.7, 29.6, 29.5, 29.3, 29.1, 25.0 (4C), 24.2; **^{11}B NMR** ($CDCl_3$, 128 MHz) δ (ppm): 34.2; **HRMS** (ESI-TOF) mass calculated for $[M+Na]^+$ $(C_{17}H_{33}BNaO_2)^+$ expected *m/z* 303.2466; found *m/z* 303.2467.

2.4. Synthesis of (3*R*,8*R*,9*S*,10*S*,13*R*,14*S*,17*R*)-10,13-dimethyl-17-((*R*)-pent-4-en-2-yl)hexadecahydro-1*H*-cyclopenta[*a*]phenanthren-3-ol (4v)

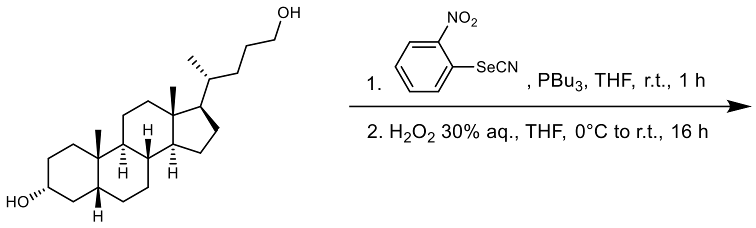

4v

The title compound was prepared according to a modified version of a literature procedure.[13]
A two-neck 25 mL oven-dried round-bottom flask equipped with a stir bar was charged with (3*R*,8*R*,9*S*,10*S*,13*R*,14*S*,17*R*)-17-((*R*)-5-hydroxypentan-2-yl)-10,13-dimethylhexadecahydro-1*H*-cyclopenta[*a*]phenanthren-3-ol) (1.0 equiv., 1.19 mmol, 432.0 mg) and THF (5.2 mL). The solution was stirred for 5 min followed by the addition of 2-nitrophenyl selenocyanate (1.96 equiv., 2.34 mmol, 530.3 mg) and tri-*n*-butylphosphine (1.96 equiv., 2.34 mmol, 583.2 μL) at room temperature. After 1.5 h of stirring at room temperature consumption of alcohol starting material was confirmed by TLC analysis. The reaction mixture was then concentrated *in vacuo*, and the resulting residue passed over a pad of silica into a 50 mL round-bottom flask equipped with a stir bar. The flask was then charged with THF (15 mL) and cooled to 0 ℃. Upon reaching temperature, H_2O_2 (30 % (w/w) in H_2O) (4 mL) was added. Following addition of the H_2O_2 the reaction was stirred for a further 16 h with no ice bath. The reaction mixture was diluted with Et_2O (20 mL) and water (20 mL), and the aqueous layer extracted with Et_2O (3×25 mL). The combined organic layers were washed with brine (25 mL), dried over anhydrous $MgSO_4$, filtered, and concentrated under reduced pressure. The crude residue was purified by flash column chromatography (SiO_2; gradient 98:2 Hexane:EtOAc to 80:20 Hexane:EtOAc) to afford olefin **4v** (246.2 mg; 60%) as a pale-yellow solid. **R**$_f$ (80:20 Hexane:EtOAc) 0.43; **M.P.**: 127-129 °C; **[α]$_D^{25}$** = 12.0 (c = 10.00 $CHCl_3$).; **IR** (film) ν_{max}/cm^{-1}: 3354, 2925, 2854, 1663, 1638, 1463, 1377, 1261, 1041, 909, 803, 729.; **^{1}H NMR** ($CDCl_3$, 500 MHz) δ (ppm): 5.85 – 5.70 (m, 1H), 5.01 – 4.98 (m, 1H), 4.97 (m, 1H), 3.62 (tt, J = 11.1, 4.7 Hz, 1H), 2.20 – 2.12 (m, 1H),

1.99 – 1.92 (m, 1H), 1.91 – 1.70 (m, 5H), 1.70 – 1.61 (m, 1H), 1.58 – 1.42 (m, 4H), 1.41 – 1.31 (m, 6H), 1.28 – 1.20 (m, 3H), 1.18 – 1.01 (m, 5H), 1.00 – 0.89 (m, 7H), 0.65 (s, 3H).; **^{13}C NMR** ($CDCl_3$, 101 MHz) δ (ppm): 137.6, 115.8, 72.0, 56.6, 56.0, 42.9, 42.3, 40.8, 40.6, 40.2, 36.6, 36.0, 36.0, 35.5, 34.7, 30.7, 28.4, 27.4, 26.6, 24.4, 23.5, 21.0, 18.7, 12.2.; **HRMS** (ESI-TOF) mass calculated for $[M+Na]^+$ $(C_{24}H_{40}NaO)^+$ expected at m/z 367.2971; found m/z 367.2958.

3. Optimization studies

3.1. Azetidines

3.1.1 Initial Amine Screening

Experiments were carried out following **General Procedure A** (*vide infra*), and the reported crude NMR yields were recorded using mesitylene (1 equiv.) as an internal standard unless otherwise stated. A 15 µl aliquot was taken and placed in an NMR tube, diluted with neutralized $CDCl_3$ and subjected to ^{1}H NMR analysis.

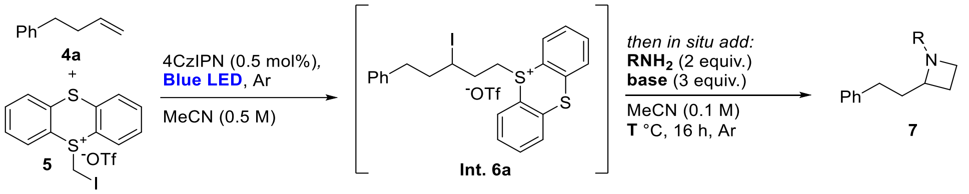

Entry[a]	Amine	Base	Temperature (℃)	Yield of 7 (%)[b]
1	*tert*-Butyl carbamate	tBuOK	25	0
2	*p*-Toluenesulfonamide	tBuOK	25	0
3	Benzylamine	2,6-Lutidine	40	30[c]
4	*p*-Anisidine	2,6-Lutidine	40	43

[a] Unless otherwise stated, reactions performed on 0.05 mmol scale, using **4a** (1.0 equiv.), **5** (1.1 equiv.); $[\mathbf{4a}]_0$ = 0.5 M irradiation time 30 mins. See section 4 for experiment procedure. [b] Unless otherwise stated, ^{1}H-NMR yield using mesitylene as internal standard.

[c] ^{1}H-NMR yield using phenanthrene as internal standard.

The moderate yields observed for benzylamine (30%) and *p*-anisidine (43%) demonstrated the potential for the development of methods to access masked free-azetidines, prompting further investigation of these systems.

3.1.2 Investigation of azetidine formation with benzylamine

Experiments were carried out following **General Procedure B** (*vide infra*), and NMR yields determined after work-up using mesitylene (1 equiv.) as an internal standard.

The NMR sample was prepared using neutralised $CDCl_3$. Integration of the doublet at δ = 3.50 (d, J = 12.6 Hz, 1H) ppm (^{1}H NMR peak corresponding to one of the diastereotopic benzylic protons in the product azetidine, **7ae**) against the standard in the resultant ^{1}H NMR spectrum provided the NMR yields reported below.

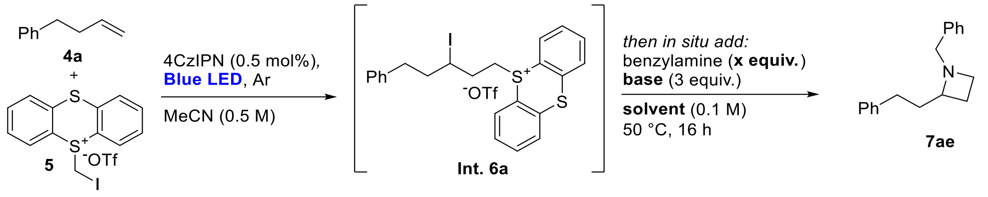

Entry[a]	Base	Solvent	Benzylamine (equiv.)	Yield of 7ae (%)[b]
1[c]	2,6-Lutidine	MeCN	2	30
2	DIPEA	MeCN	2	52
3	K_2CO_3	MeCN	2	45
4	TMG	MeCN	2	11
5	K_2HPO_4	MeCN	2	50
6	K_3PO_4	MeCN	2	54
7	K_3PO_4	THF	2	50
8	K_3PO_4	1,4-Dioxane	2	37
9	K_3PO_4	DCM	2	0
10	K_3PO_4	DMSO	2	38
11	K_3PO_4	DMPU	2	35
12	K_3PO_4	MeCN	1.1	45
13	K_3PO_4	MeCN	1.5	52
**14**	**K_3PO_4**	**MeCN**	**3**	**61**
15	K_3PO_4	MeCN	4	60

[a] Unless otherwise stated, reactions performed on 0.05 mmol scale, using **4a** (1.0 equiv.), **5** (1.1 equiv.); [**4a**]$_0$ = 0.5 M irradiation time 30 mins. See section 4 for experiment procedure. [b] ^{1}H-NMR yield using mesitylene as an internal standard. [c] 40 ℃

3.1.3 Investigation of azetidine formation with *p*-anisidine

Experiments were carried out following **General Procedure A** (*vide infra*), and the reported NMR yields were recorded using dibromomethane (1 equiv.) as internal standard. NMR yields for entries employing polar aprotic solvents (**entries 4-9**) were not obtained from the crude but instead after aqueous work up following **General Procedure A**.

The NMR sample was prepared using neutralised $CDCl_3$. Integration of the multiplet at δ = 3.47 – 3.58 ppm (^{1}H NMR peak corresponding to one of the diastereotopic protons α to the nitrogen in the product azetidine, **7a**) against the standard in the resultant ^{1}H NMR spectrum provided the NMR yields reported below.

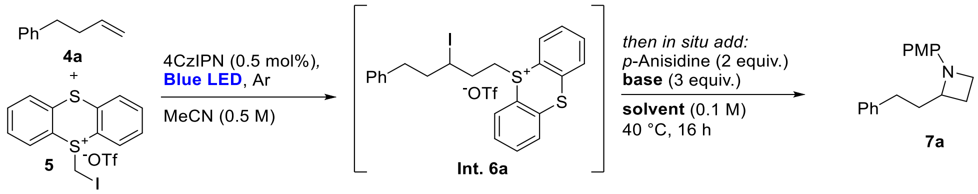

Entry[a]	Base	Solvent	Yield of 7a (%)[b]
1	2,6-Lutidine	MeCN	45
2[c]	2,6-Lutidine	1,4-Dioxane	17
3[c]	2,6-Lutidine	DCM	5
4[c]	2,6-Lutidine	DMA	63
5[c]	2,6-Lutidine	DMSO	70
6[c]	2,6-Lutidine	DMPU	70
7[c]	$NaHCO_3$	DMPU	90
8[c]	$NaHCO_3$	DMSO	70
**9[d]**	**$NaHCO_3$**	**MeCN:DMPU (1:4)**	**90**

[a] Unless otherwise stated, reactions performed on 0.05 mmol scale, using **4a** (1.0 equiv.), **5** (1.1 equiv.); $[\mathbf{4a}]_0$ = 0.5 M irradiation time 30 mins. See section 4 for experiment procedure. [b] ^{1}H-NMR yield using CH_2Br_2 as internal standard. [c] Reaction performed on isolated intermediate **6a**. [d] 50 ˚C

3.2. Oxetanes

For optimization studies, oxetane **8a** was not isolated, but was instead quantified by ^{1}H-NMR using phenanthrene as internal standard. For the study, the reaction was performed following **General Procedure C** (*vide infra*) and the internal standard (1 equiv.) was added to the reaction mixture. A 15µl aliquot was taken and placed in an NMR tube, diluted with $CDCl_3$ and subjected to analysis. Integration of the multiplet at δ = 4.57 – 4.49 (C-H proton in α-position to the oxygen of the oxetane, **8a**) against the standard in the resultant ^{1}H NMR spectrum provided the NMR yields reported below.

3.2.1 Nucleophile screening

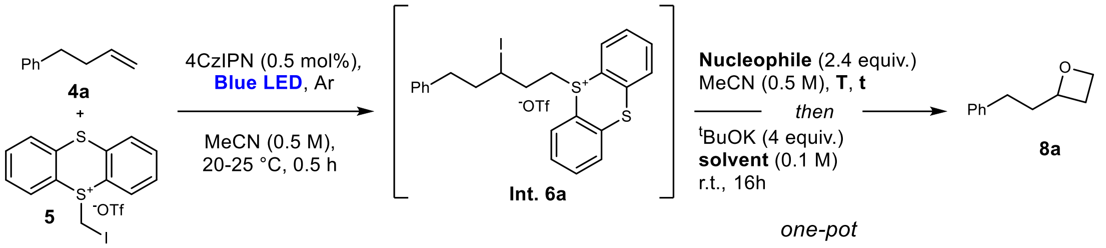

Entry[a]	Nucleophile	T	t	Solvent	Yield of 8a (%)[b]
1	KOH	r.t.	16h	MeCN	0
2	TMSOK	r.t.	16h	MeCN	0
3	NaTFA	45 °C	16h	MeCN	0
4	NaTFA	45 °C	16h	DMPU	0
5	NaTFA	45 °C	16h	THF	0
**6[c]**	**AgTFA**	0 °C	**0.5 h**	**MeCN**	**36**

[a] Unless otherwise stated, reactions performed in 0.05 mmol scale, using **4a**; (1.0 equiv.); [b] Unless otherwise stated, ^{1}H-NMR yield using phenanthrene as internal standard. See section 4 for experiment procedure; [c] Reaction performed using 2 equiv. of AgTFA.

3.2.2 Optimisation of standard reaction parameters

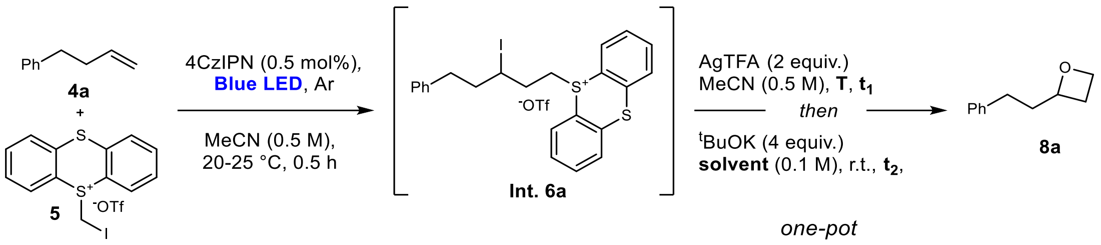

Entry[a]	T	t_1	t_2	Solvent	Yield of 8a (%)[b]
1	0 °C	0.5 h	16h	MeCN	36
2	0 °C	0.5 h	16h	DMSO	9
3	0 °C	0.5 h	16h	THF	25
4	0 °C	0.5 h	16h	DMPU	14
5	0 °C	0.5 h	16h	H_2O	7
6[c]	0 °C	0.5 h	16h	*t*BuOH	47
**7**	**0 °C**	**0.5 h**	**16h**	***t*AmylOH**	**60**
8	0 °C	0.5 h	16h	IPA	46
**9[d]**	**-10 °C**	**16 h**	**1h**	**MeCN:*t*AmylOH (1:4)**	**63**

[a] Unless otherwise stated, reactions performed in 0.05 mmol scale, using **4a**; (1.0 equiv.); [b] Unless otherwise stated, ^{1}H-NMR yield using phenanthrene as internal standard. See section 4 for experiment procedure; [c] Reaction performed at 50 °C; [d] Reaction performed on a 0.2 mmol scale. The reaction was performed without switching the solvent between steps, but simply adding the co-solvent to the vessel.

3.3. Thietanes

Experiments were carried out following **General Procedure D** (*vide infra*), and the reported NMR yields were recorded using dibromomethane (1 equiv.) as internal standard after an aqueous work up to remove inorganic salts. The extracted organic phase was concentrated and redissolved in $CDCl_3$ then subjected to ^{1}H NMR analysis. Integration of the multiplet at $\delta = 3.64 – 3.79$ ppm (C-H proton in α-position to sulfur of thietane **9a**) against the standard in the resultant ^{1}H NMR spectrum provided the NMR yields reported below.

Preliminary investigations looked to afford thietane **9a** via the disubstitution of intermediate **6a** with $Na_2S \cdot 9H_2O$, as shown in the scheme below.

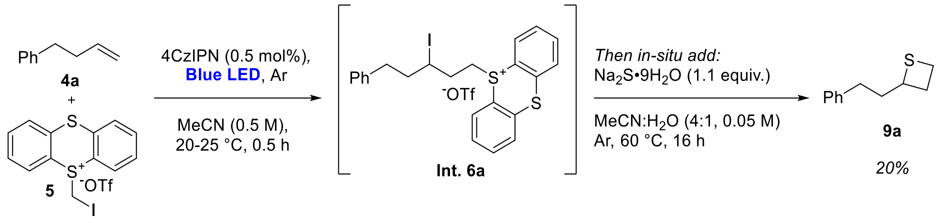

Reaction performed on 0.2 mmol scale, using **4a** (1.0 equiv.), **5** (1.1 equiv.); [**4a**]$_0$ = 0.5 M irradiation time 30 mins. See section 4 for experiment procedure. ^{1}H-NMR yield using CH_2Br_2 as internal standard.

Although formation of thietane **9a** was observed, low yields (despite attempting variety of conditions) and complex product mixtures led us to consider alternative approaches for thietane synthesis. Inspired by the approach employed for oxetane synthesis, and its potential to circumvent unwanted side reactivity, sequential mono-substitution followed by *in-situ* deprotection/cyclisation was investigated.

In initial studies, potassium thioacetate was found to react with **int. 6a** giving thioester intermediates. However, in initial attempts (entry 1 and 2) such intermediates were found to be reluctant to hydrolyse (and thus to cyclize) to give the corresponding products. The use of MeOH and K_2CO_3 allowed to increase conversion to desired thietane **9a** (entries 3 to 6).

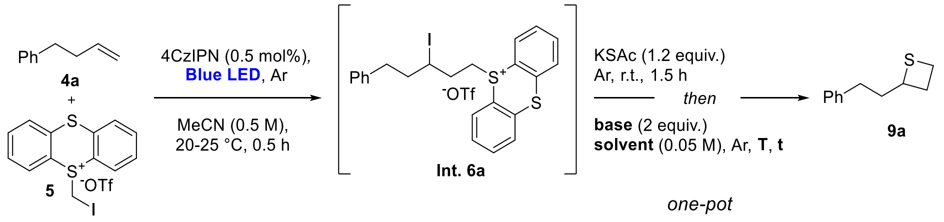

Entry[a]	Base	Solvent	Temperature (℃)	Time (h)	Yield of 9a (%)[b]
1	$NaHCO_3$	Glyme:H_2O (95:5)	85	48	6
2	$NaHCO_3$	EtOH:H_2O (95:5)	85	48	50
3	$NaHCO_3$	MeOH	60	16	54
4	$NaHCO_3$	MeOH	40	40	62
5	K_2CO_3	MeOH	40	16	63
**6**	**K_2CO_3**	**THF:MeOH (3:1)**	**40**	**5**	**67**

[a] Unless otherwise stated, reactions performed on 0.2 mmol scale, using **4a** (1.0 equiv.), **5** (1.1 equiv.); [**4a**]$_0$ = 0.5 M irradiation time 30 mins. See section 4 for experiment procedure. [b] Unless otherwise stated, ^{1}H-NMR yield using CH_2Br_2 as internal standard.

3.4. Cyclobutanes

Experiments were carried out following **General Procedure E** (*vide infra*), and the reported crude NMR yields were recorded using dibromomethane (1 equiv.) as an internal standard. Upon completion, reaction mixtures were passed through a silica plug and washed with EtOAc (5 x 5 mL). The solvent was evaporated, and the crude material was diluted with $CDCl_3$, internal standard was added, and ^{1}H NMR analysis was conducted. Integration of the multiplet at δ = 3.12 – 3.00 ppm (C-H proton in α-position to the alkyl chain of cyclobutane **10a**) against the standard in the resultant ^{1}H NMR spectrum provided the NMR yields reported below.

3.4.1 Malonate equivalence screening

The table below shows how the equivalents of added malonate affects the yield of cyclobutane **10a**. After photochemical step, in-situ solvent exchange to THF was performed prior to adding the malonate stock solution in THF.

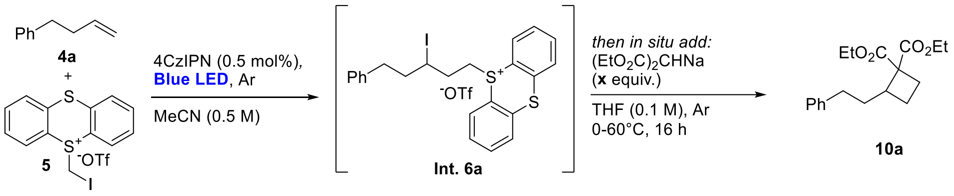

**Entry**[a]	**Malonate equivalents**	**Yield 10a** (%)[b]
1	2.0	42
**2**	**3.0**	**60**
3	4.0	57

[a] Unless otherwise stated, reactions performed on 0.05 mmol scale, using **4a** (1.0 equiv.), **5** (1.1 equiv.); $[\mathbf{4a}]_0$ = 0.5 M irradiation time 30 mins. See section 4 for experiment procedure. [b] Unless otherwise stated, ^{1}H-NMR yield using dibromomethane as internal standard.

3.4.2 Solvent Screening

To assess the effect of different solvents on the yield of cyclobutane, when solvents differing from MeCN were used, in-situ solvent exchange was performed prior to adding the malonate stock solution in THF. The solvent composition reported in table accounts for the THF introduced during addition.

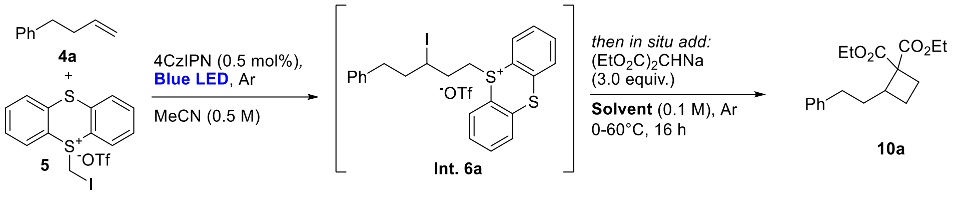

Entry[a]	Solvent/THF (1:3)	Yield **10a** (%)[b]
1	THF	60
**2**	**MeCN**	**63**
3	Toluene	59
4	DCM	63
5	DMPU	60
6	Ethanol	57
7	DME	47

[a] Unless otherwise stated, reactions performed on 0.05 mmol scale, using **4a** (1.0 equiv.), **5** (1.1 equiv.); $[\mathbf{4a}]_0$ = 0.5 M irradiation time 30 mins. See section 4 for experiment procedure.

[b] Unless otherwise stated, ^{1}H-NMR yield using dibromomethane as internal standard.

3.4.3 Investigation of cyclobutane synthesis with malononitrile

Testing the conditions developed above with malononitrile afforded no desired cyclobutane product, likely due to solubility issues of the corresponding sodium salt. Upon reversing the order of addition, (adding **int 6a** to the $(CN)_2CHNa$ solution, instead of adding the $(CN)_2CHNa$ solution to **int 6a** as a modification of **General Procedure E**), the yield was improved to 54%.

The reported crude NMR yields were recorded using dibromomethane (1 equiv.) as an internal standard. Upon completion, reaction mixtures were passed through a silica plug and washed with EtOAc (5 x 5 mL). The solvent was evaporated, and the crude material was diluted with $CDCl_3$, internal standard was added, and ^{1}H NMR analysis was conducted. Integration of the multiplet at δ = 3.12 – 3.00 ppm (C-H proton in α-position to the alkyl chain of cyclobutane **10f**) against the standard in the resultant ^{1}H NMR spectrum provided the NMR yields reported below.

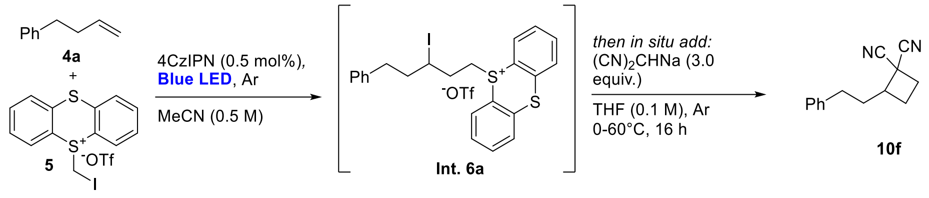

Entry[a]	Order of addition	Yield 10f (%)[b]
1	$(CN)_2CHNa$ solution to **Int 6a**	0
**2**	**Int 6a** to $(CN)_2CHNa$ solution	**54**

[a] Unless otherwise stated, reactions performed on 0.05 mmol scale, using **4a** (1.0 equiv.), **5** (1.1 equiv.); $[\mathbf{4a}]_0$ = 0.5 M irradiation time 30 mins. See section 4 for experiment procedure. [b] ^{1}H-NMR yield using dibromomethane as internal standard.

In addition, a new procedure that circumvented solubility issues was tested. Wherein instead of pre-mixing of the pronucleophile with base the pronucleophile was added directly to **int 6a** along with base. See **General Procedure F** (*vide infra*) for full experimental details. This afforded the desired cyclobutane in 65% after optimisation of the stoichiometry.

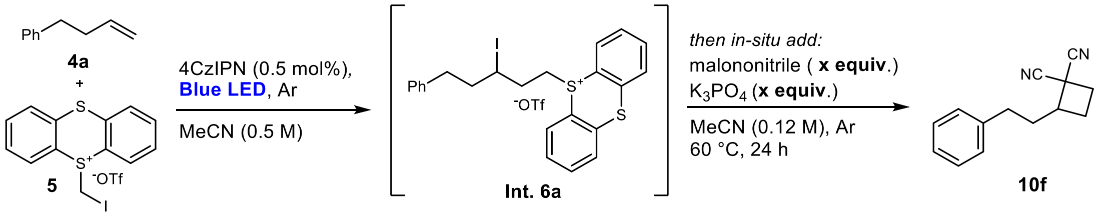

Entry[a]	malononitrile (equiv.)	K_3PO_4 (equiv.)	Yield of 9a (%)[b]
1	1.0	4.0	54
**2**	**3.0**	**6.0**	**65**[c]

[a] Unless otherwise stated, reactions performed on 0.05 mmol scale, using **4a** (1.0 equiv.), **5** (1.1 equiv.); $[\mathbf{4a}]_0$ = 0.5 M irradiation time 30 mins. See section 4 for experiment procedure. [b] ^{1}H-NMR yield using dibromomethane as internal standard. [c] Reaction performed on 0.2 mmol scale.

4. General procedures & product characterization

General procedure A – Anilines

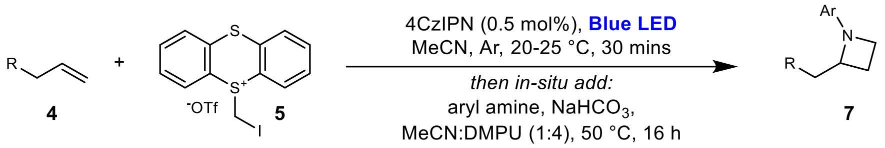

To an oven-dried Schlenk tube under an Ar atmosphere, 5-iodomethyl thianthrenium triflate **5** (1.1 equiv.; 0.22 mmol; 111.4 mg), and 4CzIPN (0.005 equiv.; 0.001 mmol; 0.8 mg) were introduced followed by degassed dry MeCN (0.4 mL, 0.5 M, previously degassed through 10 min argon sparging) through a syringe. Then olefin **4** (1.0 equiv.; 0.2 mmol) was added (if solid, it was introduced before solvent addition, together with 5-(Iodomethyl)-5H-thianthren-5-ium triflate **5** and 4CzIPN), the vessel was sealed with a glass stopper using silicon grease, and was placed in a glass-wall water bath where a fan was blowing air to keep the water temperature between 20 - 25 °C (monitored using a thermometer). See Fig. S2 for visual details of the reaction setup. The reaction was irradiated through the glass wall with blue light (Kessil lamp A160WE Tuna Blue Saltwater LED Light 40 W) for 30 mins under moderate stirring (500 rpm). The vessel was then removed from the water bath and aniline (2.0 equiv.; 0.4 mmol), $NaHCO_3$ (3.0 equiv.; 0.6 mmol; 50.4 mg), and dry 1,3-Dimethyl-3,4,5,6-tetrahydro-2(1*H*)-pyrimidinone (DMPU, 1.6 mL) were introduced into the vessel. The mixture was stirred (500 rpm) at 50 ℃ without irradiation for 16 h. The mixture was diluted with 60 mL of EtOAc, washed with saturated sodium carbonate solution (1×25mL) distilled water (5×25 mL) and brine (25 mL). The organic layer was dried over anhydrous Na_2SO_4 and concentrated under reduced pressure. The crude was subjected to chromatographic purification on silica gel to afford final compounds.

General procedure B – Aliphatic Amines

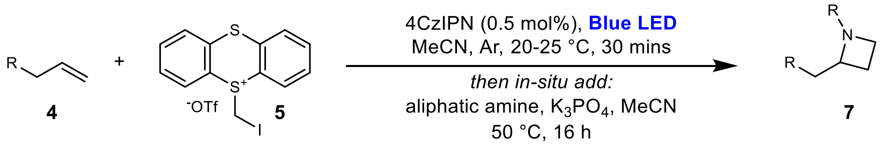

To an oven-dried Schlenk tube under an Ar atmosphere, 5-iodomethyl thianthrenium triflate **5** (1.1 equiv.; 0.22 mmol; 111.4 mg), and 4CzIPN (0.005 equiv.; 0.001 mmol; 0.8 mg) were introduced followed by degassed dry MeCN (0.4 mL, 0.5 M, previously degassed through 10 min argon sparging) through a syringe. Then olefin **4** (1.0 equiv.; 0.2 mmol) was added (if solid, it was introduced before solvent addition, together with 5-(Iodomethyl)-5H-thianthren-5-ium triflate **5** and 4CzIPN), the vessel was sealed with a glass stopper using silicon grease, and was placed in a glass-wall water bath where a fan was blowing air to keep the water temperature between 20 - 25 °C

(monitored using a thermometer). See Fig. S2 for visual details of the reaction setup. The reaction was irradiated through the glass wall with blue light (Kessil lamp A160WE Tuna Blue Saltwater LED Light 40 W) for 30 mins under moderate stirring (500 rpm). The vessel was then removed from the water bath and amine (3.0 - 4.0 equiv.; 0.6 - 0.8 mmol), K_3PO_4 (3.0 equiv.; 0.6 mmol; 127.4 mg), and acetonitrile (1.6 mL, 0.1 M) were introduced into the vessel. The mixture was stirred (500 rpm) at 50 °C without irradiation for 16 h. The mixture was diluted with 20 mL of DCM and washed with 2M NaOH (1×8mL). The organic layer was separated and the aqueous layer extracted with DCM (3 x 20 mL). The combined organic layers were dried over anhydrous Na_2SO_4 and concentrated under reduced pressure. The crude was subjected to chromatographic purification on deactivated silica gel to afford final compounds.

Methods for silica deactivation:

- *Method (i): The silica was packed in a solution of DCM + 1% NEt_3. Then flushed with DCM and subsequently dried with a stream of nitrogen before being re-packed with the desired eluent system.*
- *Method (ii): The silica was stirred in a solution of DCM + 5% (7M NH_3 in MeOH). Then dried under vacuo ensuring no MeOH remains.*

In addition, unless otherwise specified, silica plates for crude mixture evaluation were first dipped in a solution of DCM + 1% NEt_3 and dried before use.

General procedure C

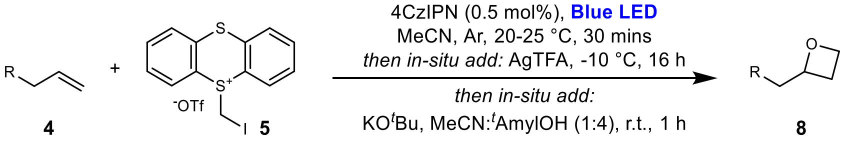

To an oven-dried Schlenk tube under an Ar atmosphere, 5-iodomethyl thianthrenium triflate **5** (1.1 equiv.; 0.22 mmol; 111.4 mg), and 4CzIPN (0.005 equiv.; 0.001 mmol; 0.8 mg) were introduced followed by degassed dry MeCN (0.4 mL, 0.5 M, previously degassed through 10 min argon sparging) through a syringe. Then olefin **4** (1.0 equiv.; 0.2 mmol) was added (if solid, it was introduced before solvent addition, together with 5-(Iodomethyl)-5H-thianthren-5-ium triflate **5** and 4CzIPN), the vessel was sealed with a glass stopper using silicon grease, and was placed in a glass-wall water bath where a fan was blowing air to keep the water temperature between 20 - 25 °C (monitored using a thermometer). See Fig. S2 for visual details of the reaction setup. The reaction was irradiated through the glass wall with blue light (Kessil lamp A160WE Tuna Blue Saltwater LED Light 40 W) for 30 mins under moderate stirring (500 rpm). After completion, the vessel was then removed from the water bath, cooled to -10 °C and AgTFA (2.0 equiv.; 0.4 mmol; 88.4 mg) was introduced into the vessel. The mixture was stirred (500 rpm) at -10 °C without irradiation for 16 h

using a cryostat. Subsequently, ^{t}AmylOH (1.6 mL) was added via syringe, and the Schlenk tube was taken out of the cryostat. Upon warming to room temperature, KO^{t}Bu (4.0 equiv.; 0.8 mmol; 89.8 mg) was introduced into the vessel. The reaction mixture was stirred (500 rpm) at room temperature in the absence of light for an additional 1 h. The reaction mixture was filtered through silica gel using ethyl acetate (30 mL) as eluent. The filtrate was concentrated under reduced pressure, and the resulting crude was purified by flash column chromatography on silica gel to afford final compounds.

General procedure D

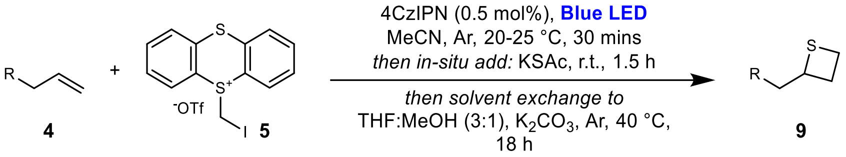

To an oven-dried Schlenk tube under an Ar atmosphere, 5-iodomethyl thianthrenium triflate **5** (1.1 equiv.; 0.22 mmol; 111.4 mg), and 4CzIPN (0.005 equiv.; 0.001 mmol; 0.8 mg) were introduced followed by degassed dry MeCN (0.4 mL, 0.5 M, previously degassed through 10 min argon sparging) through a syringe. Then olefin **4** (1.0 equiv.; 0.2 mmol) was added (if solid, it was introduced before solvent addition, together with 5-(Iodomethyl)-5H-thianthren-5-ium triflate **5** and 4CzIPN), the vessel was sealed with a glass stopper using silicon grease, and was placed in a glass-wall water bath where a fan was blowing air to keep the water temperature between 20 - 25 °C (monitored using a thermometer). See Fig. S2 for visual details of the reaction setup. The reaction was irradiated through the glass wall with blue light (Kessil lamp A160WE Tuna Blue Saltwater LED Light 40 W) for 30 mins under moderate stirring (500 rpm). The vessel was then removed from the water bath and KSAc (1.2 equiv.; 0.24 mmol; 27.4 mg) was introduced into the vessel under an Ar atmosphere. The mixture was stirred (500 rpm) at 20 - 25 °C without irradiation for 1.5 h. The vessel was then removed from the stirring and the solvent evaporated under vacuum using a standard Schlenk manifold. After removal of all MeCN the atmosphere in the vessel was exchanged to argon and the crude was submitted to the next step. Under an Ar atmosphere, K_2CO_3 (2.0 equiv.; 0.4 mmol; 55.28 mg) was added, followed by degassed THF and MeOH (3:1, 0.05 M, previously degassed separately through 10 min argon sparging and added independently to reaction mixture) through a syringe. The reaction was then allowed to stir (500 rpm) for 18 hours at 40 °C (oil bath). The mixture was diluted with 120mL of EtOAc, washed with distilled water (25 mL) and brine (25 mL). The organic layer was dried over anhydrous Na_2SO_4 and concentrated under reduced pressure. The crude was subjected to chromatographic purification on silica gel to afford final compounds.

General procedure E

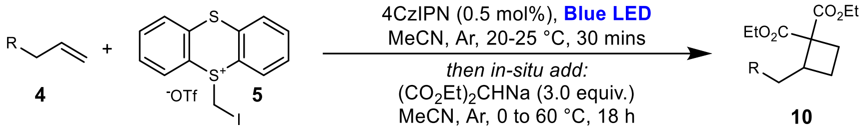

To an oven-dried Schlenk tube under an Ar atmosphere, 5-iodomethyl thianthrenium triflate **5** (1.1 equiv.; 0.22 mmol; 111.4 mg), and 4CzIPN (0.005 equiv.; 0.001 mmol; 0.8 mg) were introduced followed by degassed dry MeCN (0.4 mL, 0.5 M, previously degassed through 10 min argon sparging) through a syringe. Then olefin **4** (1.0 equiv.; 0.2 mmol) was added (if solid, it was introduced before solvent addition, together with 5-(Iodomethyl)-5H-thianthren-5-ium triflate **5** and 4CzIPN), the vessel was sealed with a glass stopper using silicon grease, and was placed in a glass-wall water bath where a fan was blowing air to keep the water temperature between 20 - 25 °C (monitored using a thermometer). See Fig. S2 for visual details of the reaction setup. The reaction was irradiated through the glass wall with blue light (Kessil lamp A160WE Tuna Blue Saltwater LED Light 40 W) for 30 mins under moderate stirring (500 rpm). The vessel was then removed from the water bath and cooled to 0 °C (ice bath) before adding $(CO_2Et)_2CHNa$ in THF (0.5M; 3.0 equiv.; 0.6 mmol; 1.2 mL) to the reaction mixture dropwise (for preparation of the $(CO_2Et)_2CHNa$ stock solution see below). The reaction was allowed to stir at 0 °C for 30 minutes before warming to room temperature and stirring for a further 2 hours. The reaction was then allowed to stir overnight for a further 16 hours at 60 °C (oil bath). Upon completion, the reaction was quenched with water (5 mL) and EtOAc (5 mL) was added. The organic layer was separated and the aqueous layer extracted with EtOAc (3 x 10 mL). The organic layer was dried over anhydrous Na_2SO_4 and concentrated under reduced pressure. The crude was subjected to chromatographic purification on silica gel to afford final compounds.

$(CO_2Et)_2CHNa$ stock solution preparation:

To an oven-dried 25 mL two-necked flask under an Ar atmosphere, NaH 60% dispersion in mineral oil (1.0 equiv.; 1.0 mmol; 40 mg) was introduced followed by dry THF. Diethyl malonate (1.0 equiv.; 1.0 mmol; 152 µL) was added to the stirred solution of NaH dropwise at room temperature (Caution: hydrogen gas evolution). The solution was allowed to stir for 15 minutes at room temperature (gas evolution ceased) and was kept under Ar.

General procedure F

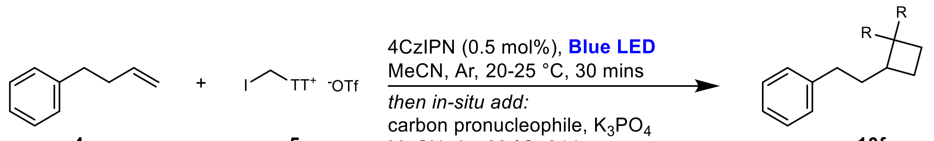

4a 5 10f

To an oven-dried Schlenk tube under an Ar atmosphere, 5-iodomethyl thianthrenium triflate **5** (1.1 equiv.; 0.22 mmol; 111.4 mg), and 4CzIPN (0.005 equiv.; 0.001 mmol; 0.8 mg) were introduced followed by degassed dry MeCN (0.4 mL, 0.5 M, previously degassed through 10 min argon sparging) through a syringe. Then olefin **4** (1.0 equiv.; 0.2 mmol) was added (if solid, it was introduced before solvent addition, together with 5-(Iodomethyl)-5H-thianthren-5-ium triflate **5** and 4CzIPN), the vessel was sealed with a glass stopper using silicon grease, and was placed in a glass-wall water bath where a fan was blowing air to keep the water temperature between 20 - 25 °C (monitored using a thermometer). See Fig. S2 for visual details of the reaction setup. The reaction was irradiated through the glass wall with blue light (Kessil lamp A160WE Tuna Blue Saltwater LED Light 40 W) for 30 mins under moderate stirring (500 rpm). The vessel was then removed from the water bath and carbon pronucleophile (3.0 equiv.; 0.6 mmol), K_3PO_4 (6.0 equiv.; 1.2 mmol; 254.7 mg), and dry acetonitrile (MeCN, 1.2 mL) were introduced into the vessel. The mixture was stirred (700 rpm) at 60 °C (oil bath) without irradiation for 24 h. Upon completion, the reaction was quenched with water (10 mL) and EtOAc (10 mL) was added. The organic layer was separated and the aqueous layer extracted with EtOAc (3 x 10 mL). The organic layer was dried over anhydrous Na_2SO_4 and concentrated under reduced pressure. The crude was subjected to chromatographic purification on silica gel to afford final compounds.

4.1. Reaction set-up for irradiation of mixtures with blue LEDs

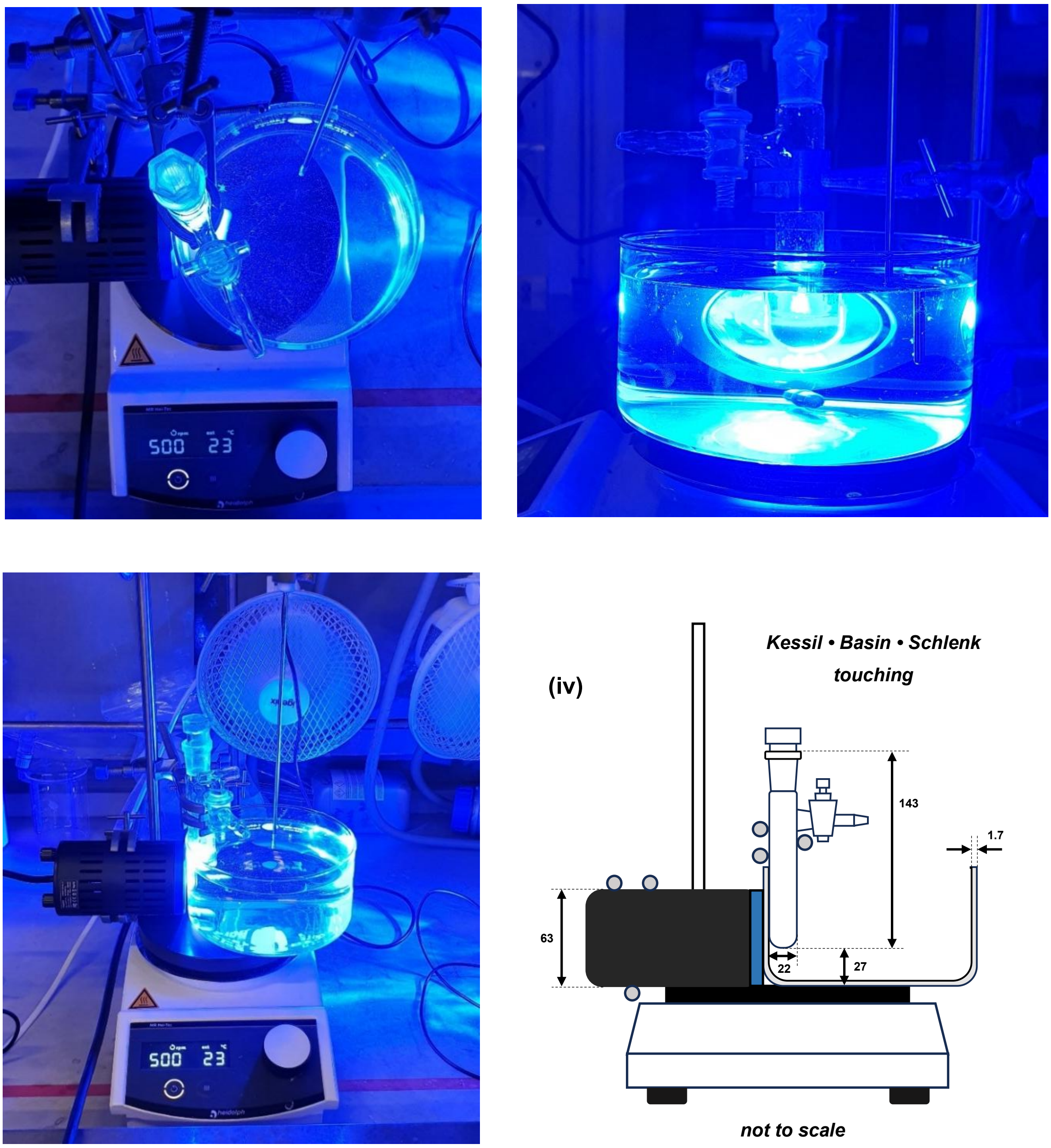

Figure S2. (i-iii) Visual details of the photochemical reaction set-up: Top, lateral and front views. **(iv)** Diagram of set up annotated with measurements (in mm) for a typical experiment. Note: The Schlenk tubes used varied slightly in dimensions. However, in all cases the Schlenk was positioned such that it was in contact with the basin, which in turn was in contact with the kessil lamp. In addition, as can be seen in picture **(ii)**, the reaction mixture was positioned in the centre of the light source to maximise light exposure.

4.2. Reaction products and characterization

1-(4-methoxyphenyl)-2-phenethylazetidine(7a)

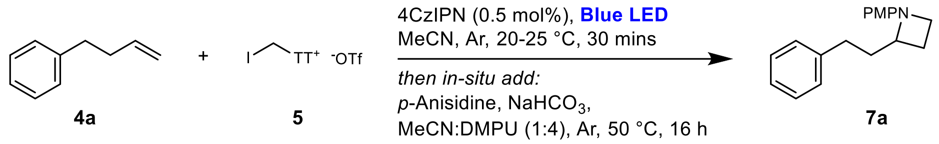

Prepared following **general procedure A**, using 4-Phenyl-1-butene **4a** (1.0 equiv.; 0.2 mmol; 30.0 µL), 5-iodomethyl thianthrenium triflate **5** (1.1 equiv.; 0.22 mmol; 111.4 mg), 4CzIPN (0.005 equiv.; 0.001 mmol; 0.8 mg), *p*-Anisidine (2.0 equiv.; 0.4 mmol; 49.3 mg), and $NaHCO_3$ (3.0 equiv.; 0.6 mmol; 50.4 mg). The crude residue was purified by flash column chromatography (SiO_2; gradient 100 pentane to 95:5 pentane:EtOAc) to afford compound **7a** (44.3mg; 83%) as an off-white solid. $\mathbf{R_f}$ (95:5 pentane:EtOAc) 0.4; **M.P.**: 55–57 °C **IR** (film) ν_{max}/cm^{-1}: 3025, 2993, 2952, 2928, 2855, 2829, 1507, 1236, 1039, 818, 698; **^{1}H NMR** ($CDCl_3$, 500 MHz) δ (ppm): 7.38 – 7.30 (m, 2H), 7.28 – 7.20 (m, 3H), 6.85 – 6.79 (m, 2H), 6.55 – 6.46 (m, 2H), 4.02 – 3.88 (m, 2H), 3.82 – 3.68 (m, 3H), 3.58 – 3.48 (m, 1H), 2.81 – 2.71 (m, 1H), 2.71 – 2.63 (m, 1H), 2.38 – 2.23 (m, 2H), 2.22 – 2.11 (m, 1H), 2.11 – 1.99 (m, 1H); **^{13}C NMR** ($CDCl_3$, 126 MHz) δ (ppm): 152.4, 147.2, 142.0, 128.5, 128.4, 126.0, 114.8, 113.3, 65.0, 55.9, 50.5, 38.7, 31.3, 23.8; **HRMS** (ESI-TOF) mass calculated for $[M+H]^+$ $(C_{18}H_{22}NO)^+$ expected *m/z* 268.1696; found *m/z* 268.1696.

10-(1-(4-methoxyphenyl)azetidin-2-yl)decanenitrile (7b)

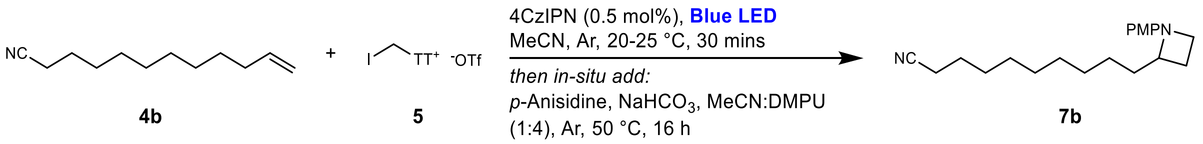

Prepared following **general procedure A**, using dodec-11-enenitrile **4b** (1.0 equiv.; 0.2 mmol; 43.0 µl), 5-iodomethyl thianthrenium triflate **5** (1.1 equiv.; 0.22 mmol; 111.4 mg), 4CzIPN (0.005 equiv.; 0.001 mmol; 0.8 mg), *p*-Anisidine (2.0 equiv.; 0.4 mmol; 49.3 mg), and $NaHCO_3$ (3.0 equiv.; 0.6 mmol; 50.4 mg). The crude residue was purified by flash column chromatography (SiO_2; gradient 93:7 pentane:EtOAc to 87:13 pentane:EtOAc) to afford compound **7b** (52.8 mg; 84%) as a yellow oil. $\mathbf{R_f}$ (87:13 pentane:EtOAc) 0.47; **IR** (film) ν_{max}/cm^{-1}: 2992, 2925, 2830, 2245, 1737, 1508, 1464, 1441, 1423, 1368, 1235, 1176, 1038, 819; **^{1}H NMR** ($CDCl_3$, 500 MHz) δ (ppm): 6.85 – 6.75 (m, 2H), 6.57 – 6.44 (m, 2H), 3.91 – 3.83 (m, 2H), 3.74 (s, 3H), 3.52 – 3.44 (m, 1H), 2.33 (app t, *J* = 7.1 Hz, 2H), 2.30 – 2.24 (m, 1H), 2.13 – 2.05 (m, 1H), 1.96 – 1.89 (m, 1H), 1.73 – 1.62 (m, 3H), 1.48 – 1.42 (m, 2H), 1.36 – 1.28 (m, 10H).; **^{13}C NMR** ($CDCl_3$, 101 MHz) δ (ppm): 152.4, 147.2, 120.0, 114.7,

113.3, 65.6, 55.9, 50.5, 37.2, 29.8, 29.6, 29.3, 28.8, 28.7, 25.5, 24.9, 23.9, 17.2; **HRMS** (ESI-TOF) mass calculated for $[M+H]^+$ $(C_{20}H_{31}N_2O)^+$ expected *m/z* 315.2431; found *m/z* 315.2431.

methyl 9-(1-(4-methoxyphenyl)azetidin-2-yl)nonanoate (**7c**)

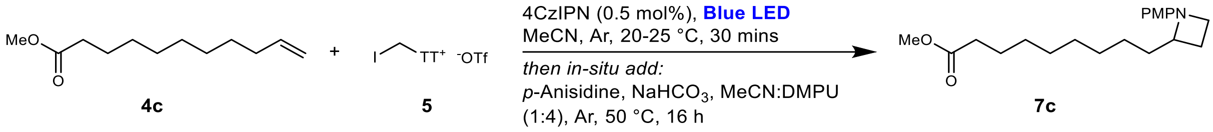

Prepared following **general procedure A**, using methyl undec-10-enoate **4c** (1.0 equiv.; 0.2 mmol; 45 μL), 5-iodomethyl thianthrenium triflate **5** (1.1 equiv.; 0.22 mmol; 111.4 mg), 4CzIPN (0.005 equiv.; 0.001 mmol; 0.8 mg), *p*-Anisidine (2.0 equiv.; 0.4 mmol; 49.3 mg), and $NaHCO_3$ (3.0 equiv.; 0.6 mmol; 50.4 mg). The crude residue was purified by flash column chromatography (SiO_2; gradient 93:7 pentane:EtOAc to 87:13 pentane:EtOAc) to afford compound **7c** (50.9 mg; 76%) as a yellow oil. $\mathbf{R_f}$ (87:13 pentane:EtOAc) 0.55; **IR** (film) ν_{max}/cm^{-1}: 3039, 2993, 2926, 2853, 1738, 1510, 1464, 1437, 1367, 1325, 1294, 1237, 1197, 1176, 1115, 1041, 944, 820, 795, 724, 640, 611, 527; ^{1}**H NMR** ($CDCl_3$, 500 MHz) δ (ppm): 6.83 – 6.76 (m, 2H), 6.53 – 6.43 (m, 2H), 3.91 – 3.82 (m, 2H), 3.75 (s, 3H), 3.67 (s, 3H), 3.51 – 3.43 (m, 1H), 2.33 – 2.24 (m, 3H), 2.13 – 2.04 (m, 1H), 1.97 – 1.85 (m, 1H), 1.71 – 1.59 (m, 3H), 1.36 – 1.28 (m, 10H); ^{13}**C NMR** ($CDCl_3$, 126 MHz) δ (ppm): 174.5, 152.4, 147.5, 114.8, 113.3, 65.6, 55.9, 51.6, 50.5, 37.3, 34.2, 29.8, 29.6, 29.3, 29.3, 25.1, 25.0, 24.0.; **HRMS** (ESI-TOF) mass calculated for $[M+H]^+$ $(C_{20}H_{32}NO_3)^+$ expected *m/z* 334.2377; found *m/z* 334.2371.

9-(1-(4-methoxyphenyl)azetidin-2-yl)nonanamide (**7d**)

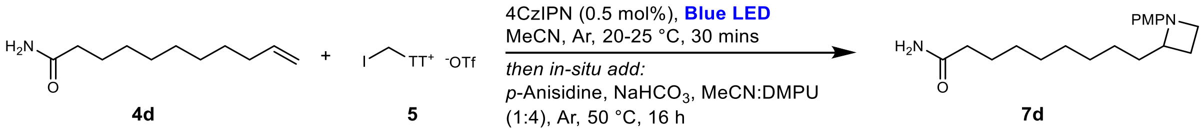

Prepared following **general procedure A**, using undec-10-enamide **4d** (1.0 equiv.; 0.2 mmol; 36.7 mg), 5-iodomethyl thianthrenium triflate **5** (1.1 equiv.; 0.22 mmol; 111.4 mg), 4CzIPN (0.005 equiv.; 0.001 mmol; 0.8 mg), *p*-Anisidine (2.0 equiv.; 0.4 mmol; 49.3 mg), and $NaHCO_3$ (3.0 equiv.; 0.6 mmol; 50.4 mg). The crude residue was purified by flash column chromatography (SiO_2; gradient 20:80 DCM:EtOAc to 100% EtOAc to 95:5 EtOAc:MeOH) to afford compound **7d** (46.4 mg; 73%) as a white solid. $\mathbf{R_f}$ (95:5 EtOAc:MeOH) 0.52; **M.P.**: 137-139 °C; **IR** (film) ν_{max}/cm^{-1}: 3366, 3184, 2921, 2850, 1660, 1632, 1512, 1249, 1034, 822, 702, 529; ^{1}**H NMR** ($CDCl_3$, 500 MHz) δ (ppm): 6.82 – 6.77 (m, 2H), 6.60 – 6.45 (m, 2H), 5.39 (s, 2H), 3.94 – 3.83 (m, 2H), 3.75 (s, 3H), 3.54 – 3.43 (m, 1H), 2.33 – 2.24 (m, 1H), 2.24 – 2.19 (m, 2H), 2.15 – 2.03 (m, 1H), 1.97 – 1.84 (m, 1H), 1.73 – 1.58 (m, 3H), 1.35 – 1.28 (m, 10H); ^{13}**C NMR** ($CDCl_3$, 101 MHz) δ (ppm): 175.5, 152.4, 147.5,

114.8, 113.3, 65.5, 56.0, 50.5, 37.3, 36.0, 29.8, 29.6, 29.4, 29.3, 25.6, 25.0, 24.0; **HRMS** (ESI-TOF) mass calculated for $[M+H]^+$ $(C_{19}H_{31}N_2O_2)^+$ expected *m/z* 319.2380; found *m/z* 319.2386.

9-(1-(4-methoxyphenyl)azetidin-2-yl)nonanoic acid (**7e**)

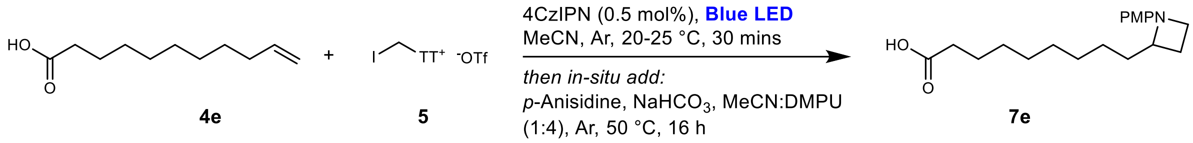

Prepared following **general procedure A**, using undec-10-enoic acid **4e** (1.0 equiv.; 0.2 mmol; 40.4 µL), 5-iodomethyl thianthrenium triflate **5** (1.1 equiv.; 0.22 mmol; 111.4 mg), 4CzIPN (0.005 equiv.; 0.001 mmol; 0.8 mg), *p*-Anisidine (2.0 equiv.; 0.4 mmol; 49.3 mg), and $NaHCO_3$ (4.0 equiv.; 0.8 mmol; 67.2 mg). The crude residue was purified by flash column chromatography (SiO_2; gradient 90:10 pentane:EtOAc to 50:50 pentane:EtOAc) to afford compound **7e** (41.5 mg; 65%) as a pale-yellow oil. $\mathbf{R_f}$ (70:30 pentane:EtOAc) 0.25; **IR** (film) ν_{max}/cm^{-1}: 2926, 2854, 1707, 1510, 1465, 1239, 1179, 1040, 821, 609; **^{1}H NMR** ($CDCl_3$, 500 MHz) δ (ppm): 6.84 – 6.77 (m, 2H), 6.54 – 6.47 (m, 2H), 3.94 – 3.81 (m, 2H), 3.75 (s, 3H), 3.52 – 3.43 (m, 1H), 2.34 (app t, J = 7.5 Hz, 2H), 2.33 – 2.23 (m, 1H), 2.15 – 2.04 (m, 1H), 1.97 – 1.88 (m, 1H), 1.75 – 1.58 (m, 3H), 1.36 – 1.30 (m, 10H); **^{13}C NMR** ($CDCl_3$, 126 MHz) δ (ppm): 179.6, 152.6, 147.3, 114.7, 113.6, 65.7, 55.9, 50.6, 37.2, 34.2, 29.8, 29.6, 29.3, 29.2, 25.0, 24.8, 24.0; **HRMS** (ESI-TOF) mass calculated for $[M+H]^+$ $(C_{19}H_{30}NO_3)^+$ expected *m/z* 320.2220; found *m/z* 320.2232.

9-(1-(4-methoxyphenyl)azetidin-2-yl)nonan-1-amine (**7f**)

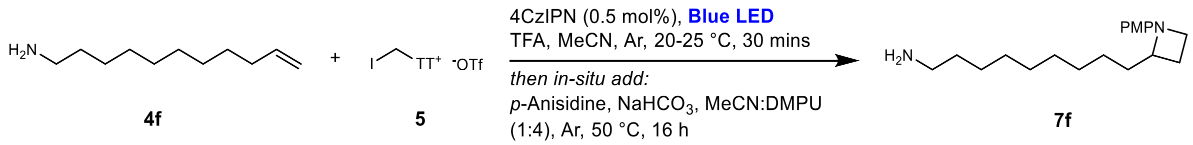

Prepared following **modified general procedure A**, to an oven-dried Schlenk tube exchanged the atmosphere with Ar, undec-10-en-1-amine **4f** (1.0 equiv.; 0.2 mmol; 41.8 µL) and degassed dry MeCN (0.4 mL, 0.5 M, previously degassed through 10 min argon sparging) were introduced followed by TFA (1.0 equiv.; 0.2 mmol; 15.3 µL) and stirred at room temperature for 10 mins. Then 5-iodomethyl thianthrenium triflate **5** (1.1 equiv.; 0.22 mmol; 111.4 mg), 4CzIPN (0.005 equiv.; 0.001 mmol; 0.8 mg) were added to the mixture, sealed the vessel and placed in a glass-wall water bath where a fan was blowing air to keep the water temperature between 20 - 25 °C. See Fig. S2 for visual details of the reaction setup. The reaction was irradiated through the glass wall with blue light (Kessil lamp A160WE Tuna Blue Saltwater LED Light 40 W) for 30 mins under moderate stirring (500 rpm). The vessel was then removed from the water bath and *p*-Anisidine (2.0 equiv.; 0.4 mmol; 49.3 mg), $NaHCO_3$ (4.0 equiv.; 0.8 mmol; 67.2 mg), and dry DMPU (1.6 mL) were introduced into

the vessel. The mixture was stirred (700 rpm) at 50℃ without irradiation for 16 h. Then 6.0 mL NaOH solution (2 M) was introduced in the vessel and continued the stirring for another 10 mins. The mixture was diluted with 60 mL of EtOAc, washed with distilled water (5×25 mL) and brine (25 mL). The organic layer was dried over anhydrous Na_2SO_4 and concentrated under reduced pressure. The crude residue was purified by flash column chromatography gradient 95:5 $CHCl_3$:MeOH + 0.5% aq. NH_3 to 90:10 $CHCl_3$:MeOH + 0.5% aq. NH_3) to afford compound **7f** (24.9 mg; 41%) as a pale-yellow oil. **R_f** (90:10 $CHCl_3$:MeOH + 1% aq. NH_3) 0.3; **IR** (film) ν_{max}/cm^{-1}: 2925, 2852, 1510, 1464, 1326, 1297, 1238, 1115, 1042, 819; **^{1}H NMR** ($CDCl_3$, 500 MHz) δ (ppm): 6.82 – 6.78 (m, 2H), 6.51 – 6.45 (m, 2H), 3.91 – 3.82 (m, 2H), 3.75 (s, 3H), 3.52 – 3.43 (m, 1H), 2.68 (app t, J = 7.0 Hz, 1H), 2.31 – 2.23 (m, 1H), 2.13 – 2.04 (m, 1H), 1.99 – 1.85 (m, 1H), 1.75 – 1.61 (m, 1H), 1.48 – 1.27 (m, 16H); **^{13}C NMR** ($CDCl_3$, 126 MHz) δ (ppm): 152.3, 147.4, 114.7, 113.2, 65.5, 55.9, 50.5, 42.4, 37.3, 33.9, 29.8, 29.7, 29.7, 29.6, 27.0, 25.0, 23.9; **HRMS** (EI-TOF) mass calculated for $[M+H]^+$ $(C_{19}H_{33}N_2O)^+$ expected *m/z* 305.2587; found *m/z* 305.2593.

8-(1-(4-methoxyphenyl)azetidin-2-yl)octan-1-ol (**7g**)

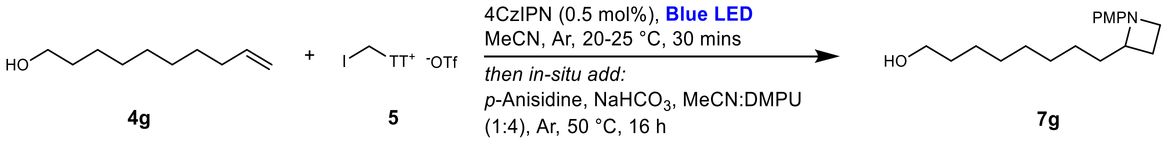

Prepared following **general procedure A**, using 9-decen-1-ol **4g** (1.0 equiv.; 0.2 mmol; 35.7 µL), 5-iodomethyl thianthrenium triflate **5** (1.1 equiv.; 0.22 mmol; 111.4 mg), 4CzIPN (0.005 equiv.; 0.001 mmol; 0.8 mg), *p*-Anisidine (2.0 equiv.; 0.4 mmol; 49.3 mg), and $NaHCO_3$ (3.0 equiv.; 0.6 mmol; 50.4 mg). The crude residue was purified by flash column chromatography (SiO_2; gradient 90:10 DCM:EtOAc to 80:20 DCM:EtOAc) to afford compound **7g** (48 mg; 82%) as a yellow oil. **R_f** (80:20 DCM:EtOAc) 0.4; **IR** (film) ν_{max}/cm^{-1}: 3358, 3039, 2992, 2925, 2853, 1617, 1509, 1464, 1440, 1368, 1326, 1294, 1237, 1179, 1116, 1040, 946, 820, 796, 723, 610, 528; **^{1}H NMR** ($CDCl_3$, 500 MHz) δ (ppm): 6.84 – 6.75 (m, 2H), 6.56 – 6.41 (m, 2H), 3.92 – 3.81 (m, 2H), 3.75 (s, 3H), 3.63 (app t, J = 6.6 Hz, 2H), 3.51 – 3.43 (m, 1H), 2.32 – 2.24 (m, 1H), 2.14 – 2.03 (m, 1H), 2.00 – 1.85 (m, 1H), 1.73 – 1.66 (m, 1H), 1.60 – 1.53 (m, 2H), 1.42 – 1.28 (m, 11H); **^{13}C NMR** ($CDCl_3$, 126 MHz) δ (ppm): 152.4, 147.4, 114.7, 113.3, 65.6, 63.2, 55.9, 50.5, 37.3, 32.9, 29.8, 29.7, 29.5, 25.9, 25.0, 24.0; **HRMS** (ESI-TOF) mass calculated for $[M+H]^+$ $(C_{18}H_{30}NO_2)^+$ expected *m/z* 292.2271; found *m/z* 292.2278.

3-(1-(4-methoxyphenyl)azetidin-2-yl)propan-1-ol (7h)

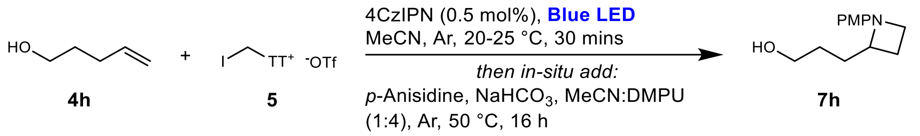

Prepared following **general procedure A**, using pent-4-en-1-ol **4h** (1.0 equiv.; 0.2 mmol; 20.3 µL), 5-iodomethyl thianthrenium triflate **5** (1.1 equiv.; 0.22 mmol; 111.4 mg), 4CzIPN (0.005 equiv.; 0.001 mmol; 0.8 mg), *p*-Anisidine (2.0 equiv.; 0.4 mmol; 49.3 mg), and $NaHCO_3$ (3.0 equiv.; 0.6 mmol; 50.4 mg). The crude residue was purified by flash column chromatography (SiO_2; gradient 50:50 pentane:EtOAc to 40:60 pentane:EtOAc) to afford compound **7h**, alongside trace impurities. The mixture was re-purified by flash column chromatography (SiO_2; gradient 80:20 pentane:acetone to 75:25 pentane:acetone) to afford compound **7h** (9.7 mg; 23%) as a pale-yellow oil. $\mathbf{R_f}$ (50:50 pentane:EtOAc) 0.27; **IR** (film) ν_{max}/cm^{-1}: 3345, 2925, 2854, 1690, 1618, 1509, 1465, 1326, 1294, 1238, 1180, 1115, 1039, 947, 821, 797, 610, 526; **^{1}H NMR** ($CDCl_3$, 400 MHz) δ (ppm): 6.83 – 6.77 (m, 2H), 6.55 – 6.49 (m, 2H), 3.96 (qd, *J* = 7.9, 3.8 Hz, 1H), 3.89 (ddd, *J* = 8.4, 6.9, 3.5 Hz, 1H), 3.75 (s, 3H), 3.73 – 3.61 (m, 2H), 3.55 – 3.46 (m, 1H), 2.32 – 2.10 (m, 3H), 2.05 – 1.94 (m, 1H), 1.87 – 1.77 (m, 1H), 1.76 – 1.69 (m, 1H), 1.69 – 1.62 (m, 1H); **^{13}C NMR** ($CDCl_3$, 101 MHz) δ (ppm): 152.8, 147.1, 114.8 (2C), 113.7 (2C), 65.1, 63.1, 55.9, 50.5, 33.4, 28.0, 22.9; **HRMS** (ESI-TOF) mass calculated for $[M+H]^+$ ($C_{13}H_{20}NO_2^+$) expected *m/z* 222.1489; found *m/z* 222.1489.

2-(1-(4-methoxyphenyl)azetidin-2-yl)ethan-1-ol (7i)

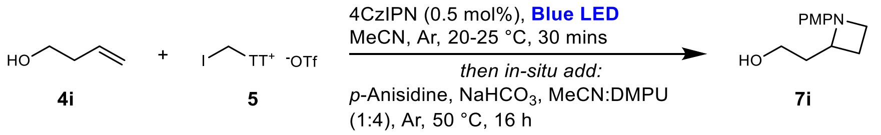

Prepared following **general procedure A**, using but-3-en-1-ol **4i** (1.0 equiv.; 0.2 mmol; 17.2 µL), 5-iodomethyl thianthrenium triflate **5** (1.1 equiv.; 0.22 mmol; 111.4 mg), 4CzIPN (0.005 equiv.; 0.001 mmol; 0.8 mg), *p*-Anisidine (2.0 equiv.; 0.4 mmol; 49.3 mg), and $NaHCO_3$ (3.0 equiv.; 0.6 mmol; 50.4 mg). The crude residue was purified by flash column chromatography (SiO_2; gradient 55:45 pentane:EtOAc to 45:55 pentane:EtOAc) to afford compound **7i**, alongside trace impurities. The mixture was re-purified by flash column chromatography (SiO_2; gradient 80:20 pentane:acetone to 70:30 pentane:acetone) to afford compound **7i** (17.7 mg; 43%) as a pale-yellow oil. $\mathbf{R_f}$ (65:45 pentane:EtOAc) 0.27; **IR** (film) ν_{max}/cm^{-1}: 3388, 2994, 2924, 2852, 1509, 1466, 1442, 1367, 1324, 1293, 1239, 1180, 1116, 1040, 972, 944, 822, 795, 610, 527; **^{1}H NMR** ($CDCl_3$, 400 MHz) δ (ppm): 6.84 – 6.79 (m, 2H), 6.62 – 6.56 (m, 2H), 4.25 – 4.17 (m, 1H), 4.13 (ddd, *J* = 11.1, 9.5, 3.6 Hz, 1H), 3.92 (ddd, *J* = 8.6, 7.0, 3.3 Hz, 1H), 3.80 (dt, *J* = 11.1, 4.8 Hz, 1H), 3.75 (s, 3H), 3.58 – 3.48 (m, 1H),

2.46 – 2.34 (m, 1H), 2.28 – 2.18 (m, 2H), 1.88 – 1.78 (m, 1H); **^{13}C NMR** ($CDCl_3$, 101 MHz) δ (ppm): 153.2, 146.9, 114.7 (2C), 114.2 (2C), 64.2, 59.7, 55.9, 51.0, 36.6, 21.4; **HRMS** (ESI-TOF) mass calculated for $[M+H]^+$ ($C_{12}H_{18}NO_2^+$) expected *m/z* 208.1332; found *m/z* 208.1334.

2-(3-((tert-butyldimethylsilyl)oxy)propyl)-1-(4-methoxyphenyl)azetidine (**7j**)

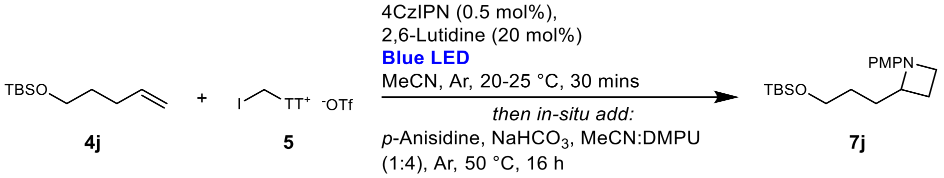

Prepared following a modification to **general procedure A**; To an oven-dried Schlenk tube under an Ar atmosphere, 5-iodomethyl thianthrenium triflate **5** (1.1 equiv.; 0.22 mmol; 111.4 mg), and 4CzIPN (0.005 equiv.; 0.001 mmol; 0.8 mg) were introduced followed by degassed dry MeCN (0.4 mL, 0.5 M, previously degassed through 10 min argon sparging) through a syringe. Then tert-butyldimethyl(pent-4-en-1-yloxy)silane **4j** (1.0 equiv.; 0.2 mmol; 50.1 µL) was added, followed by 2,6-lutidine (20 mol %; 0.04 mmol; 4.7 µL). The vessel was then sealed with a glass stopper using silicon grease and was placed in a glass-wall water bath where a fan was blowing air to keep the water temperature between 20 - 25 °C. See Fig. S2 for visual details of the reaction setup. The reaction was irradiated through the glass wall with blue light (Kessil lamp A160WE Tuna Blue Saltwater LED Light 40 W) for 30 mins under moderate stirring (500 rpm). The vessel was then removed from the water bath and *p*-Anisidine (2.0 equiv.; 0.4 mmol; 49.3 mg), $NaHCO_3$ (3.0 equiv.; 0.6 mmol; 50.4 mg), and dry 1,3-Dimethyl-3,4,5,6-tetrahydro-2(1*H*)-pyrimidinone (DMPU, 1.6 mL) were introduced into the vessel. The mixture was stirred (500 rpm) at 50°C without irradiation for 16 h. The mixture was diluted with 60 mL of EtOAc, washed with saturated sodium carbonate solution (1×25mL) distilled water (5×25 mL) and brine (25 mL). The organic layer was dried over anhydrous Na_2SO_4 and concentrated under reduced pressure. The crude residue was purified by flash column chromatography (SiO_2; deactivated using 99:1 DCM:Et_3N; elution gradient 100% pentane to 80:20 pentane:DCM) to afford compound **7j** (40.7 mg; 61%) as a pale-beige-coloured oil. **R_f** (80:20 pentane:DCM) 0.15; **IR** (film) ν_{max}/cm^{-1}: 2992, 2951, 2928, 2855, 1509, 1471, 1387, 1361, 1326, 1294, 1237, 1180, 1094, 1040, 1006, 989, 940, 834, 817, 774, 713, 662, 610, 527; **^{1}H NMR** ($CDCl_3$, 500 MHz) δ (ppm) 6.84 – 6.76 (m, 2H), 6.53 – 6.46 (m, 2H), 3.95 – 3.85 (m, 2H), 3.75 (s, 3H), 3.66 (t, *J* = 6.5 Hz, 2H), 3.52 – 3.45 (m, 1H), 2.33 – 2.24 (m, 1H), 2.15 – 2.05 (m, 1H), 2.04 – 1.95 (m, 1H), 1.79 – 1.69 (m, 1H), 1.64 – 1.49 (m, 2H), 0.92 (s, 9H), 0.07 (d, *J* = 1.2 Hz, 6H); **^{13}C NMR** ($CDCl_3$, 126 MHz) δ (ppm): 152.4, 147.4, 114.8 (2C), 113.3 (2C), 65.3, 63.1, 55.9, 50.4, 33.4, 28.3, 26.1 (3C), 23.8, 18.5, -5.1 (2C); **HRMS** (ESI-TOF) mass calculated for $[M+H]^+$ ($C_{19}H_{34}NO_2Si^+$) expected *m/z* 336.2353; found *m/z* 336.2355.

2-(2-((tert-butyldimethylsilyl)oxy)ethyl)-1-(4-methoxyphenyl)azetidine (**7k**)

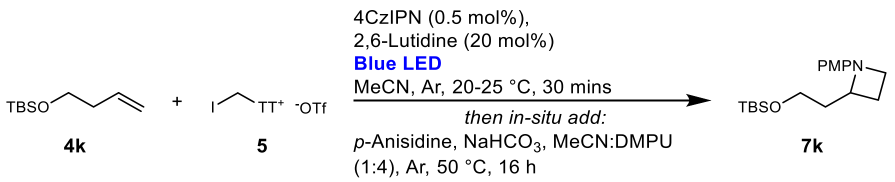

Prepared following a modification to **general procedure A**; To an oven-dried Schlenk tube under an Ar atmosphere, 5-iodomethyl thianthrenium triflate **5** (1.1 equiv.; 0.22 mmol; 111.4 mg), and 4CzIPN (0.005 equiv.; 0.001 mmol; 0.8 mg) were introduced followed by degassed dry MeCN (0.4 mL, 0.5 M, previously degassed through 10 min argon sparging) through a syringe. Then (but-3-en-1-yloxy)(tert-butyl)dimethylsilane **4k** (1.0 equiv.; 0.2 mmol; 46.6 μL) was added, followed by 2,6-lutidine (20 mol %; 0.04 mmol; 4.7 μL). The vessel was then sealed with a glass stopper using silicon grease and was placed in a glass-wall water bath where a fan was blowing air to keep the water temperature between 20 - 25 °C. See Fig. S2 for visual details of the reaction setup. The reaction was irradiated through the glass wall with blue light (Kessil lamp A160WE Tuna Blue Saltwater LED Light 40 W) for 30 mins under moderate stirring (500 rpm). The vessel was then removed from the water bath and *p*-Anisidine (2.0 equiv.; 0.4 mmol; 49.3 mg), $NaHCO_3$ (3.0 equiv.; 0.6 mmol; 50.4 mg), and dry 1,3-Dimethyl-3,4,5,6-tetrahydro-2(1*H*)-pyrimidinone (DMPU, 1.6 mL) were introduced into the vessel. The mixture was stirred (500 rpm) at 50 ℃ without irradiation for 16 h. The mixture was diluted with 60 mL of EtOAc, washed with saturated sodium carbonate solution (1×25mL) distilled water (5×25 mL) and brine (25 mL). The organic layer was dried over anhydrous Na_2SO_4 and concentrated under reduced pressure. The crude residue was purified by flash column chromatography (SiO_2; gradient 98:2 pentane:EtOAc to 95:5 pentane:EtOAc) to afford compound **7k** (38.8 mg; 60%) as a pale-beige-coloured oil. **R_f** (98:2 pentane:EtOAc) 0.3; **IR** (film) ν_{max}/cm^{-1}: 2992, 2952, 2928, 2855, 1509, 1471, 1388, 1361, 1325, 1294, 1237, 1179, 1093, 1043, 1006, 986, 938, 902, 887, 818, 774, 730, 662, 610, 525, 451; **^{1}H NMR** ($CDCl_3$, 400 MHz) δ (ppm) 6.83 – 6.78 (m, 2H), 6.57 – 6.51 (m, 2H), 4.12 – 4.02 (m, 1H), 3.97 – 3.89 (m, 1H), 3.75 (s, 3H), 3.75 – 3.70 (m, 2H), 3.55 – 3.46 (m, 1H), 2.38 – 2.28 (m, 1H), 2.22 – 2.11 (m, 2H), 1.98 – 1.86 (m, 1H), 0.92 (s, 9H), 0.08 (d, *J* = 2.2 Hz, 6H); **^{13}C NMR** ($CDCl_3$, 101 MHz) δ (ppm): 152.4, 147.6, 114.7 (2C), 113.4 (2C), 62.9, 59.8, 55.9, 51.1, 40.5, 26.1 (3C), 24.3, 18.4, -5.2 (d, *J* = 1.9 Hz, 2C); **HRMS** (ESI-TOF) mass calculated for $[M+H]^+$ ($C_{18}H_{32}NO_2Si^+$) expected *m/z* 322.2197; found *m/z* 322.2196.

2-(9-chlorononyl)-1-(4-methoxyphenyl)azetidine (7l)

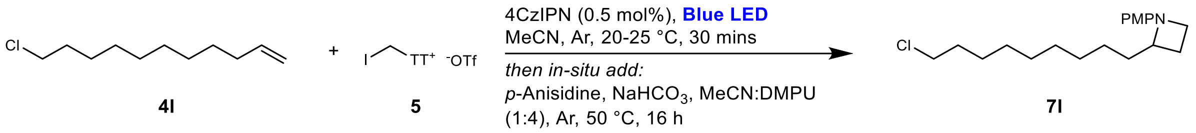

Prepared following **general procedure A**, using 11-chloroundec-1-ene **4l** (1.0 equiv.; 0.2 mmol; 38 μL), 5-iodomethyl thianthrenium triflate **5** (1.1 equiv.; 0.22 mmol; 111.4 mg), 4CzIPN (0.005 equiv.; 0.001 mmol; 0.8 mg), *p*-Anisidine (2.0 equiv.; 0.4 mmol; 49.3 mg), and $NaHCO_3$ (3.0 equiv.; 0.6 mmol; 50.4 mg). The crude residue was purified by flash column chromatography (SiO_2; gradient 95:5 pentane:EtOAc to 85:15 pentane:EtOAc) to afford compound **7l** (40.2 mg; 62%) as a transparent oil. **R$_f$** (85:15 hexane:EtOAc) 0.25; **IR** (film) ν_{max}/cm^{-1}:2925, 2853, 1508, 1464, 1441, 1293, 1235, 1115, 1040, 818; **^{1}H NMR** ($CDCl_3$, 500 MHz) δ (ppm): 6.89 – 6.70 (m, 2H), 6.57 – 6.43 (m, 2H), 3.93 – 3.82 (m, 2H), 3.75 (s, 3H), 3.54 (app t, J = 6.7 Hz, 2H), 3.51 – 3.44 (m, 1H), 2.33 – 2.22 (m, 1H), 2.14 – 2.04 (m, 1H), 1.98 – 1.88 (m, 1H), 1.82 – 1.74 (m, 2H), 1.73 – 1.65 (m, 1H), 1.47 – 1.27 (m, 12H).; **^{13}C NMR** ($CDCl_3$, 126 MHz) δ (ppm): 152.4, 147.4, 114.7, 113.2, 65.5, 55.9, 50.5, 45.3, 37.3, 32.8, 29.8, 29.7, 29.5, 29.0, 27.0, 25.0, 23.9.; **HRMS** (ESI-TOF) mass calculated for $[M+H]^+$ $(C_{19}H_{31}ClNO)^+$ expected *m/z* 324.2089; found *m/z* 324.2096.

2-(4-(benzyloxy)butyl)-1-(4-methoxyphenyl)azetidine (7m)

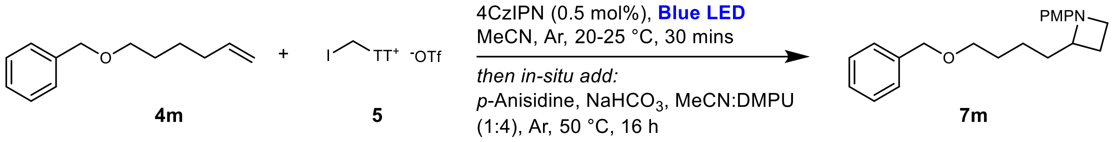

Prepared following **general procedure A**, using ((hex-5-en-1-yloxy)methyl)benzene **4m** (1.0 equiv.; 0.2 mmol; 38.0 mg), 5-iodomethyl thianthrenium triflate **5** (1.1 equiv.; 0.22 mmol; 111.4 mg), 4CzIPN (0.005 equiv.; 0.001 mmol; 0.8 mg), *p*-Anisidine (2.0 equiv.; 0.4 mmol; 49.3 mg), and $NaHCO_3$ (3.0 equiv.; 0.6 mmol; 50.4 mg). The crude residue was purified by flash column chromatography (SiO_2; gradient 100 pentane to 90:10 pentane:EtOAc) to afford compound **7m** (38.7 mg; 59%) as a transparent oil. **R$_f$** (90:10 pentane:EtOAc) 0.5; **IR** (film) ν_{max}/cm^{-1}: 3035, 2992, 2932, 2856, 2830, 1509, 1237, 1099, 820, 611. **^{1}H NMR** ($CDCl_3$, 500 MHz) δ (ppm): 7.44 – 7.31 (m, 4H), 7.31 – 7.26 (m, 1H), 6.89 – 6.72 (m, 2H), 6.58 – 6.38 (m, 2H), 4.52 (s, 2H), 3.94 – 3.83 (m, 2H), 3.75 (s, 3H), 3.54 – 3.45 (m, 3H), 2.37 – 2.24 (m, 1H), 2.15 – 2.05 (m, 1H), 2.02 – 1.91 (m, 1H), 1.76 – 1.65 (m, 3H), 1.51 – 1.37 (m, 2H); **^{13}C NMR** ($CDCl_3$, 126 MHz) δ (ppm): 152.4, 147.3, 138.7, 128.5, 127.8, 127.7, 114.8, 113.3, 73.1, 70.4, 65.4, 55.9, 50.5, 37.0, 30.0, 23.9, 21.7.; **HRMS** (ESI-TOF) mass calculated for $[M+H]^+$ $(C_{21}H_{28}NO_2)^+$ expected *m/z* 326.2115; found *m/z* 326.2121.

1-(4-methoxyphenyl)-2-(4-(phenylthio)butyl)azetidine (7n)

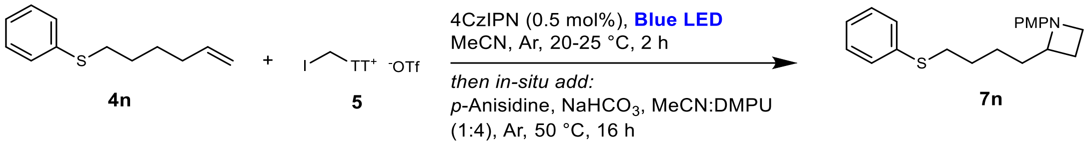

Prepared following **general procedure A**, irradiating for 2 h, using hex-5-en-1-yl(phenyl)sulfane **4n** (1.0 equiv.; 0.2 mmol; 42.7 µL), 5-iodomethyl thianthrenium triflate **5** (1.1 equiv.; 0.22 mmol; 111.4 mg), 4CzIPN (0.005 equiv.; 0.001 mmol; 0.8 mg), *p*-Anisidine (2.0 equiv.; 0.4 mmol; 49.3 mg), and $NaHCO_3$ (3.0 equiv.; 0.6 mmol; 50.4 mg). The crude residue was purified by flash column chromatography (SiO_2; gradient 90:10 pentane:EtOAc to 80:20 pentane:EtOAc) to afford compound **7n** (49.8 mg; 76%) as a pale-yellow oil. **R$_f$** (85:15 hexane:EtOAc) 0.32 ; **IR** (film) ν_{max}/cm^{-1}: 2951, 2854, 1507, 1478, 1462, 1233, 1040, 819, 737, 619; **^{1}H NMR** ($CDCl_3$, 500 MHz) δ (ppm): 7.37 – 7.32 (m, 2H), 7.32 – 7.27 (m, 2H), 7.21 – 7.15 (m, 1H), 6.83 – 6.79 (m, 2H), 6.51 – 6.45 (m, 2H), 3.91 – 3.83 (m, 2H), 3.76 (s, 3H), 3.52 – 3.45 (m, 1H), 2.99 – 2.93 (m, 2H), 2.32 – 2.24 (m, 1H), 2.14 – 2.04 (m, 1H), 1.99 – 1.89 (m, 1H), 1.77 – 1.68 (m, 3H), 1.56 – 1.43 (m, 2H); **^{13}C NMR** ($CDCl_3$, 126 MHz) δ (ppm): 152.4, 147.3, 136.9, 129.1, 129.0, 125.9, 114.8, 113.2, 65.2, 55.9, 50.5, 36.7, 33.7, 29.4, 24.2, 23.8; **HRMS** (ESI-TOF) mass calculated for $[M+H]^+$ $(C_{20}H_{26}NOS)^+$ expected *m/z* 328.1730; found *m/z* 328.1739.

1-(4-methoxyphenyl)-2-(4-(phenylsulfonyl)butyl)azetidine(7o)

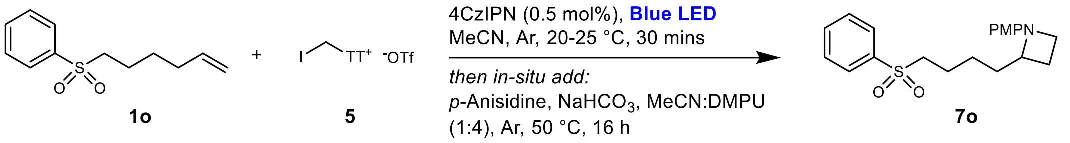

Prepared following **general procedure A**, using (hex-5-en-1-ylsulfonyl)benzene **4o** (1.0 equiv.; 0.2 mmol; 44.8 mg), 5-iodomethyl thianthrenium triflate **5** (1.1 equiv.; 0.22 mmol; 111.4 mg), 4CzIPN (0.005 equiv.; 0.001 mmol; 0.8 mg), *p*-Anisidine (2.0 equiv.; 0.4 mmol; 49.3 mg), and $NaHCO_3$ (3.0 equiv.; 0.6 mmol; 50.4 mg). The crude residue was purified by flash column chromatography (SiO_2; gradient 100 pentane to 70:30 pentane:THF) to afford compound **7o** (51.5 mg; 72%) as a pale-yellow oil. **R$_f$** (80:20 pentane:THF) 0.2; **IR** (film) ν_{max}/cm^{-1}: 2992, 2948, 2855, 2831, 1507, 1233, 1143, 1086, 1035, 821, 533; **^{1}H NMR** ($CDCl_3$, 500 MHz) δ (ppm): 7.95 – 7.85 (m, 2H), 7.70 – 7.62 (m, 1H), 7.60 – 7.52 (m, 2H), 6.81 – 6.75 (m, 2H), 6.48 – 6.39 (m, 2H), 3.87 – 3.80 (m, 2H), 3.74 (s, 3H), 3.52 – 3.39 (m, 1H), 3.15 – 3.08 (m, 2H), 2.27 – 2.19 (m, 1H), 2.07 – 1.99 (m, 1H), 1.91 – 1.83 (m, 1H), 1.81 – 1.73 (m, 2H), 1.71 – 1.63 (m, 1H), 1.49 – 1.37 (m, 2H); **^{13}C NMR** ($CDCl_3$, 126 MHz) δ (ppm): 152.4, 147.0, 139.2, 133.8, 129.4, 128.1, 114.7, 113.1, 64.6, 56.3, 55.9, 50.4, 36.3, 23.7, 23.5,

23.0; **HRMS** (ESI-TOF) mass calculated for $[M+H]^+$ $(C_{20}H_{26}NO_3S)^+$ expected *m/z* 360.1628; found *m/z* 360.1634.

diethyl (4-(1-(4-methoxyphenyl)azetidin-2-yl)butyl)phosphonate (**7p**)

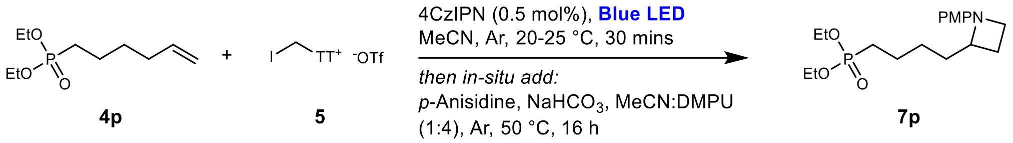

Prepared following **general procedure A**, using diethyl hex-5-en-1-ylphosphonate **4p** (1.0 equiv.; 0.2 mmol; 45.0 µL), 5-iodomethyl thianthrenium triflate **5** (1.1 equiv.; 0.22 mmol; 111.4 mg), 4CzIPN (0.005 equiv.; 0.001 mmol; 0.8 mg), *p*-Anisidine (2.0 equiv.; 0.4 mmol; 49.3 mg), and $NaHCO_3$ (3.0 equiv.; 0.6 mmol; 50.4 mg). The crude residue was purified by flash column chromatography (SiO_2; gradient 75:25 pentane:acetone to 55:45 pentane:acetone) to afford compound **7p** (54.8 mg; 77%) as a yellow oil. **R_f** (55:45 pentane:acetone) 0.55; **IR** (film) ν_{max}/cm^{-1}: 3475, 3040, 2988, 2931, 2905, 2855, 2832, 1619, 1510, 1442, 1391, 1367, 1326, 1294, 1238, 1180, 1116, 1098, 1059, 1026, 961, 821, 791, 611, 534; **^{1}H NMR** ($CDCl_3$, 500 MHz) δ (ppm): 6.82 – 6.76 (m, 2H), 6.52 – 6.40 (m, 2H), 4.13 – 4.05 (m, 4H), 3.91 – 3.84 (m, 2H), 3.74 (s, 3H), 3.53 – 3.42 (m, 1H), 2.31 – 2.23 (m, 1H), 2.12 – 2.05 (m, 1H), 1.96 – 1.89 (m, 1H), 1.80 – 1.73 (m, 2H), 1.71 – 1.60 (m, 3H), 1.49 – 1.39 (m, 2H), 1.32 (app t, J = 7.0 Hz, 6H); **^{13}C NMR** ($CDCl_3$, 126 MHz) δ (ppm): 152.44, 147.27, 114.79, 113.24, 65.10, 61.58 (d, *J* = 6.4 Hz), 55.95, 50.49, 36.69, 25.98 (d, *J* = 16.9 Hz), 25.90 (d, *J* = 140.6 Hz), 23.77, 22.79 (d, *J* = 5.2 Hz), 16.63 (d, *J* = 6.0 Hz); **^{31}P NMR** (202 MHz, $CDCl_3$) δ 32.26 – 32.04 (m); **HRMS** (ESI-TOF) mass calculated for $[M+H]^+$ $(C_{18}H_{31}NO_4P)^+$ expected *m/z* 356.1985; found *m/z* 356.1984.

1-(4-methoxyphenyl)-2-(9-(4,4,5,5-tetramethyl-1,3,2-dioxaborolan-2-yl)nonyl)azetidine (**7q**)

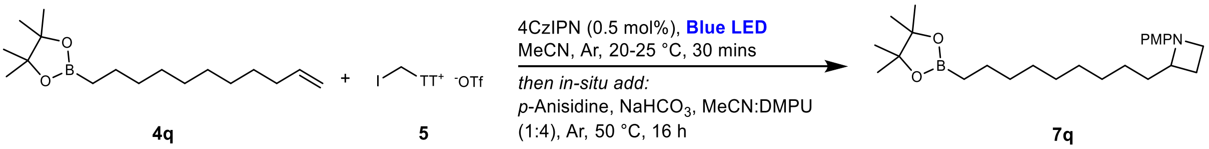

Prepared following **general procedure A**, using 4,4,5,5-tetramethyl-2-(undec-10-en-1-yl)-1,3,2-dioxaborolane **4q** (1.0 equiv.; 0.2 mmol; 65.3 µL), 5-iodomethyl thianthrenium triflate **5** (1.1 equiv.; 0.22 mmol; 111.4 mg), 4CzIPN (0.005 equiv.; 0.001 mmol; 0.8 mg), *p*-Anisidine (2.0 equiv.; 0.4 mmol; 49.3 mg), and $NaHCO_3$ (3.0 equiv.; 0.6 mmol; 50.4 mg). The crude residue was purified by flash column chromatography (SiO_2; gradient 95:5 pentane:EtOAc to 85:15 pentane:EtOAc) to afford compound **7q** (44.0 mg; 53%) as a colourless oil. **R_f** (80:20 hexane:EtOAc) 0.31; **IR** (film) ν_{max}/cm^{-1}: 2995, 2924, 2853, 1509, 1465, 1735, 1318, 1237, 1144, 819; **^{1}H NMR** ($CDCl_3$, 500 MHz) δ (ppm):

6.90 – 6.69 (m, 2H), 6.56 – 6.43 (m, 2H), 3.91 – 3.80 (m, 2H), 3.74 (s, 3H), 3.51 – 3.43 (m, 1H), 2.33 – 2.21 (m, 1H), 2.15 – 2.01 (m, 1H), 1.97 – 1.87 (m, 1H), 1.74 – 1.62 (m, 1H), 1.46 – 1.26 (m, 14H), 1.24 (s, 12H), 0.77 (app t, J = 7.8 Hz, 2H); **^{13}C NMR** ($CDCl_3$, 126 MHz) δ (ppm): 152.3, 147.5, 114.7, 113.26, 83.0, 65.6, 55.9, 50.5, 37.4, 32.6, 29.9, 29.8, 29.6, 29.5, 25.0, 25.0, 24.1, 24.0; **^{11}B NMR** ($CDCl_3$, 128 MHz) δ (ppm): 34.09; **HRMS** (ESI-TOF) mass calculated for $[M+H]^+$ $(C_{25}H_{43}BNO_3)^+$ expected *m/z* 416.3331; found *m/z* 416.3343.

1-(4-methoxyphenyl)-2-(undec-10-yn-1-yl)azetidine (**7r**)

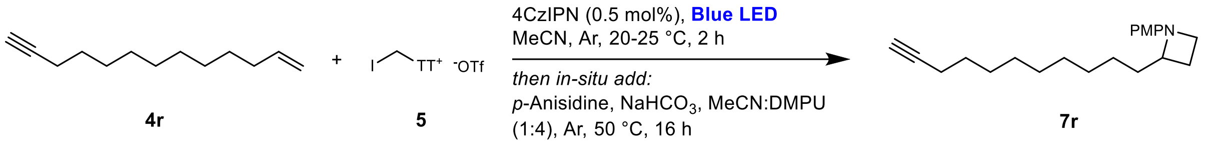

Prepared following **general procedure A**, irradiating for 2 h, using tridec-1-en-12-yne **4r** (1.0 equiv.; 0.2 mmol; 45.2 μL), 5-iodomethyl thianthrenium triflate **5** (1.1 equiv.; 0.22 mmol; 111.4 mg), 4CzIPN (0.005 equiv.; 0.001 mmol; 0.8 mg), *p*-Anisidine (2.0 equiv.; 0.4 mmol; 49.3 mg), and $NaHCO_3$ (3.0 equiv.; 0.6 mmol; 50.4 mg). The crude residue was purified by flash column chromatography (SiO_2; gradient 95:5 pentane: EtOAc to 85:15 pentane:EtOAc) to afford compound **7r** (28.8 mg; 46%) as a colourless oil. **R_f** (85:15 Pentane:EtOAc) 0.32 ; **IR** (film) ν_{max}/cm^{-1}: 3310, 3289, 2856, 2924, 2853, 1508, 1464, 1236, 1179, 1115, 1041, 819; **^{1}H NMR** ($CDCl_3$, 500 MHz) δ (ppm): 7.29 – 7.21 (m, 2H), 6.99 – 6.89 (m, 2H), 4.35 – 4.27 (m, 2H), 4.20 (s, 3H), 3.97 – 3.89 (m, 1H), 2.76 – 2.68 (m, 1H), 2.64 (app td, J = 7.1, 2.7 Hz, 2H), 2.58 – 2.49 (m, 1H), 2.42 – 2.33 (m, 2H), 2.19 – 2.09 (m, 1H), 2.02 – 1.94 (m, 2H), 1.86 – 1.72 (m, 12H); **^{13}C NMR** ($CDCl_3$, 126 MHz) δ (ppm): 152.4, 147.5, 114.8, 113.3, 84.9, 68.2, 65.6, 55.9, 50.5, 37.3, 29.9, 29.7, 29.6, 29.2, 28.9, 28.6, 25.0, 24.0, 18.5. **HRMS** (ESI-TOF) mass calculated for $[M+H]^+$ $(C_{21}H_{32}NO)^+$ expected *m/z* 314.2478; found *m/z* 314.2483.

ethyl (E)-10-(1-(4-methoxyphenyl)azetidin-2-yl)dec-2-enoate (**7s**)

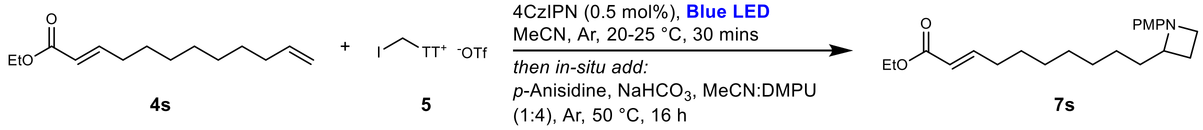

Prepared following **general procedure A**, using ethyl (E)-dodeca-2,11-dienoate **4s** (1.0 equiv.; 0.2 mmol; 50.8 μL), 5-iodomethyl thianthrenium triflate **5** (1.1 equiv.; 0.22 mmol; 111.4 mg), 4CzIPN (0.005 equiv.; 0.001 mmol; 0.8 mg), *p*-Anisidine (2.0 equiv.; 0.4 mmol; 49.3 mg), and $NaHCO_3$ (3.0 equiv.; 0.6 mmol; 50.4 mg). (SiO_2; gradient 95:5 pentane:EtOAc to 90:10 pentane:EtOAc) to afford compound **7s** (53.2 mg; 75%) as a yellow oil. **R_f** (90:10 pentane:EtOAc) 0.5 ; **IR** (film) ν_{max}/cm^{-1}:

2990, 2927, 2855, 1720, 1653, 1511, 1464, 1442, 1367, 1323, 1295, 1238, 1179, 1116, 1041, 982, 820, 610, 525; **^{1}H NMR** ($CDCl_3$, 500 MHz) δ (ppm): 6.96 (app dt, J = 15.7, 7.0 Hz, 1H), 6.83 – 6.73 (m, 2H), 6.54 – 6.44 (m, 2H), 5.81 (app dt, J = 15.5, 1.6 Hz, 1H), 4.18 (q, J = 7.1 Hz, 2H), 3.91 – 3.82 (m, 2H), 3.75 (s, 3H), 3.52 – 3.43 (m, 1H), 2.31 – 2.24 (m, 1H), 2.23 – 2.17 (m, 2H), 2.12 – 2.04 (m, 1H), 1.97 – 1.86 (m, 1H), 1.74 – 1.65 (m, 1H), 1.49 – 1.42 (m, 2H), 1.37 – 1.31 (m, 8H), 1.29 (t, J = 7.1 Hz, 3H); **^{13}C NMR** ($CDCl_3$, 126 MHz) δ (ppm): 166.9, 152.4, 149.5, 147.4, 121.4, 114.8, 113.3, 65.5, 60.3, 55.9, 50.5, 37.3, 32.3, 29.7, 29.6, 29.2, 28.1, 25.0, 24.0, 14.4; **HRMS** (ESI-TOF) mass calculated for $[M+H]^+$ $(C_{22}H_{34}NO_3)^+$ expected m/z 360.2533; found m/z 360.2537.

3-(4-(1-(4-methoxyphenyl)azetidin-2-yl)butyl)pyridine (**7t**)

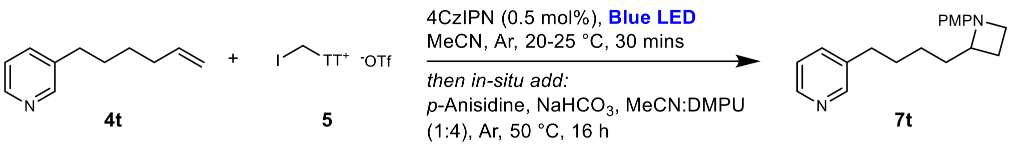

Prepared following **general procedure A**, using 3-(hex-5-en-1-yl)pyridine **4t** (1.0 equiv.; 0.2 mmol; 35.3 µL), 5-iodomethyl thianthrenium triflate **5** (1.1 equiv.; 0.22 mmol; 111.4 mg), 4CzIPN (0.005 equiv.; 0.001 mmol; 0.8 mg), *p*-Anisidine (2.0 equiv.; 0.4 mmol; 49.3 mg), and $NaHCO_3$ (3.0 equiv.; 0.6 mmol; 50.4 mg). The crude residue was purified by flash column chromatography (SiO_2; gradient 90:10 pentane:EtOAc to 80:20 pentane:EtOAc) to afford compound **7t** (33.8 mg; 57%) as a colourless oil. **R_f** (70:30 pentane:EtOAc) 0.3; **IR** (film) ν_{max}/cm^{-1}: 2929, 2856, 1509, 1477, 1422, 1237, 1115, 1039, 820, 714; **^{1}H NMR** ($CDCl_3$, 500 MHz) δ (ppm): δ 8.48 – 8.42 (m, 2H), 7.49 (app dt, J = 7.8, 2.0 Hz, 1H), 7.24 – 7.17 (m, 1H), 6.83 – 6.76 (m, 2H), 6.51 – 6.44 (m, 2H), 3.92 – 3.81 (m, 2H), 3.74 (s, 3H), 3.52 – 3.43 (m, 1H), 2.71 – 2.58 (m, 2H), 2.31 – 2.21 (m, 1H), 2.12 – 2.02 (m, 1H), 2.01 – 1.90 (m, 1H), 1.79 – 1.63 (m, 3H), 1.48 – 1.31 (m, 2H); **^{13}C NMR** ($CDCl_3$, 126 MHz) δ (ppm): 152.4, 150.1, 147.5, 147.3, 137.7, 135.9, 123.4, 114.8, 113.2, 65.2, 55.9, 50.5, 37.0, 33.2, 31.4, 24.5, 23.8; **HRMS** (ESI-TOF) mass calculated for $[M+H]^+$ $(C_{19}H_{25}N_2O)^+$ expected m/z 297.1961; found m/z 297.1966.

1-(4-methoxyphenyl)-2-phenylazetidine (**7u**)

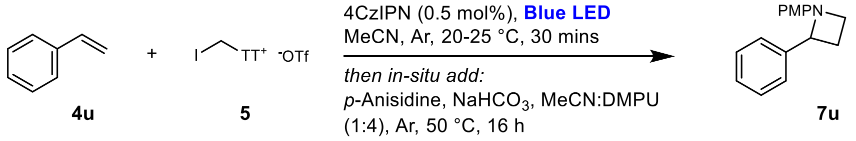

Prepared following **general procedure A**, using styrene **4u** (1.0 equiv.; 0.2 mmol; 22.9 µL), 5-iodomethyl thianthrenium triflate **5** (1.1 equiv.; 0.22 mmol; 111.4 mg), 4CzIPN (0.005 equiv.; 0.001 mmol; 0.8 mg), *p*-Anisidine (2.0 equiv.; 0.4 mmol; 49.3 mg), and $NaHCO_3$ (3.0 equiv.; 0.6 mmol; 50.4 mg). The crude residue was purified by flash column chromatography (SiO_2; gradient 90:10

DCM:EtOAc to 85:15 DCM:EtOAc) to afford compound **7u** (22.4 mg; 47%) as a yellow oil. **R_f** (90:10 Pentane:EtOAc) 0.4; **IR** (film) ν_{max}/cm^{-1}: 2996, 2832, 1508, 1239, 1041, 821, 755, 699, 642, 624; **^{1}H NMR** ($CDCl_3$, 500 MHz) δ (ppm): 7.50 – 7.48 (m, 2H), 7.40 – 7.37 (m, 2H), 7.31 – 7.28 (m, 1H), 6.78 – 6.75 (m, 2H), 6.44 – 6.41 (m, 2H), 4.83 (t, J = 7.9 Hz, 1H), 4.00 (ddd, J = 9.1, 6.6, 3.0 Hz, 1H), 3.73 (s, 3H), 3.70 – 3.65 (m, 1H), 2.63 – 2.57 (m, 1H), 2.38 – 2.31 (m, 1H); **^{13}C NMR** ($CDCl_3$, 126 MHz) δ (ppm): 152.6, 147.0, 144.0, 128.8, 127.5, 126.1, 114.7, 113.4, 68.1, 56.0, 49.8, 28.1.; **HRMS** (ESI-TOF) mass calculated for $[M+H]^+$ $(C_{16}H_{18}NO)^+$ expected *m/z* 240.1383; found *m/z* 240.1383.

(3R,8R,9S,10S,13R,14S,17R)-17-((2R)-1-(1-(4-methoxyphenyl)azetidin-2-yl)propan-2-yl)-10,13-dimethylhexadecahydro-1H-cyclopenta[a]phenanthren-3-ol (**7v**)

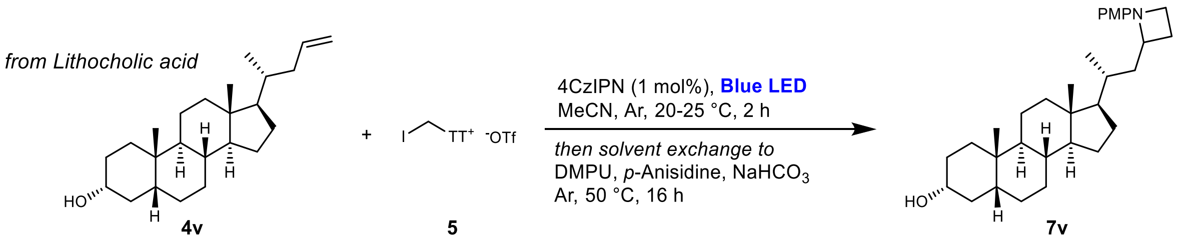

Prepared following **modified general procedure A**: An oven-dried Schlenk tube with atmosphere exchanged to Ar was charged with 5-iodomethyl thianthrenium triflate **5** (1.5 equiv.; 0.15 mmol; 76.0 mg), 4CzIPN (0.01 equiv.; 0.001 mmol; 0.8 mg) and lithocholic acid-derived olefin **4v** (1.0 equiv.; 0.10 mmol; 34.5 mg), followed by degassed dry MeCN (0.4 mL, 0.25 M, previously degassed through 10 min argon sparging). The vessel was sealed and placed in a glass-wall water bath where a fan was circulating air to maintain a water temperature of 20 - 25 °C. See Fig. S2 for visual details of the reaction setup. The reaction was irradiated through the glass wall with blue light (Kessil lamp A160WE Tuna Blue Saltwater LED Light 40 W) for 2 h under moderate-strong stirring (650 rpm). The vessel was then taken out from the water bath and solvent was removed under vacuum using a standard Schlenk manifold (ensuring that no MeCN remains). *p*-Anisidine (2.0 equiv.; 0.2 mmol; 24.6 mg), $NaHCO_3$ (3.0 equiv.; 0.3 mmol; 25.2 mg) and DMPU (1 mL) were then added, the vessel re-sealed, and the mixture stirred strongly (700 rpm) at 50 ˚C without irradiation for 16 h. Upon completion, the reaction mixture was diluted with EtOAc (ca. 60 mL) and washed sequentially with $NaHCO_3$, distilled water (3x60 mL) and brine. The organic layer was dried over anhydrous Na_2SO_4, filtered and concentrated under reduced pressure. The crude residue was purified by flash column chromatography (SiO_2; gradient 80:20 to 70:30 pentane:EtOAc) to afford compound **7v** alongside *p*-Anisidine. The impure mixture was further purified by flash column chromatography (SiO_2; gradient 97:3 to 95:5 DCM:EtOAc) to remove *p*-Anisidine and afford fractions containing the pure *major* diasteroisomer of **7v** (13.9 mg), the pure *minor* diastereoisomer of **7v** (4.1 mg), and mixed fractions

(4.5 mg) containing both diastereomers of **7v**; (total 22.5 mg; 47%, d.r. = 3:1) as a brown oil. The respective data for each diastereomer of **7v** is reported below. The relative configuration of major and minor diastereomers could not be unequivocally assigned.

Major Diastereomer: **R_f** (70:30 pentane:EtOAc) 0.53; **$[\alpha]_D^{25}$** = +4.00 (c = 1.00 $CHCl_3$).; **IR** (film) ν_{max}/cm^{-1}: 3359, 2926, 2863, 1618, 1509, 1467, 1465, 1449, 1443, 1376, 1324, 1293, 1178, 1114, 1090, 1068, 1038, 1013, 992, 965, 945, 908, 820, 795, 731, 646, 618, 605, 532, 485; **^{1}H NMR** ($CDCl_3$, 400 MHz) δ (ppm): 6.83 – 6.77 (m, 2H), 6.54 – 6.44 (m, 2H), 4.04 – 3.93 (m, 1H), 3.91 – 3.83 (m, 1H), 3.75 (s, 3H), 3.68 – 3.57 (m, 1H), 3.51 – 3.41 (m, 1H), 2.33 – 2.21 (m, 1H), 2.11 – 2.02 (m, 1H), 2.02 – 1.91 (m, 2H), 1.91 – 1.70 (m, 5H), 1.70 – 1.61 (m, 2H), 1.55 – 1.45 (m, 2H), 1.43 – 1.34 (m, 6H), 1.30 – 1.22 (m, 3H), 1.21 – 1.11 (m, 3H), 1.11 – 1.03 (m, 3H), 1.02 – 0.96 (m, 4H), 0.92 (s, 3H), 0.67 (s, 3H); **^{13}C NMR** ($CDCl_3$, 101 MHz) δ (ppm): 152.5, 147.6, 114.8, 113.4, 72.0, 63.8, 56.7 (2C overlapping), 55.9, 50.5, 43.8, 42.9, 42.2, 40.6, 40.4, 36.6, 36.0, 35.5, 34.7, 32.9, 30.7, 28.9, 27.3, 26.6, 24.5, 24.4, 23.5, 21.0, 19.4, 12.2; **HRMS** (ESI-TOF) mass calculated for $[M+H]^+$ $(C_{32}H_{50}NO_2)^+$ expected m/z 480.3836; found m/z 480.3842.

Minor Diastereomer: **R_f** (70:30 pentane:EtOAc) 0.45; **$[\alpha]_D^{25}$** = −8.00 (c = 1.00 $CHCl_3$).; **IR** (film) ν_{max}/cm^{-1}: 3349, 2934, 2863, 1510, 1468, 1444, 1378, 1361, 1325, 1295, 1239, 1179, 1115, 1089, 1068, 1041, 1012, 946, 909, 819, 794, 733, 645, 616, 516, 446; **^{1}H NMR** ($CDCl_3$, 400 MHz) δ (ppm): 6.83 – 6.77 (m, 2H), 6.52 – 6.42 (m, 2H), 3.95 – 3.88 (m, 1H), 3.88 – 3.80 (m, 1H), 3.75 (s, 3H), 3.68 – 3.57 (m, 1H), 3.51 – 3.42 (m, 1H), 2.41 – 2.30 (m, 1H), 2.24 – 2.14 (m, 1H), 2.12 – 2.04 (m, 1H), 2.03 – 1.96 (m, 1H), 1.94 – 1.83 (m, 2H), 1.83 – 1.62 (m, 4H), 1.53 – 1.45 (m, 2H), 1.44 – 1.36 (m, 6H), 1.36 – 1.27 (m, 3H), 1.22 – 1.17 (m, 1H), 1.17 – 0.99 (m, 6H), 0.99 – 0.87 (m, 7H), 0.68 (s, 3H); **^{13}C NMR** ($CDCl_3$, 101 MHz) δ (ppm): 152.5, 147.5, 114.8, 113.4, 72.0, 65.6, 57.0, 56.7, 55.9, 51.5, 44.1, 43.0, 42.3, 40.6, 40.4, 36.6, 36.0, 35.5, 35.3, 34.7, 30.7, 29.0, 27.3, 27.0, 26.6, 24.4, 23.5, 21.0, 19.8, 12.2; **HRMS** (ESI-TOF) mass calculated for $[M+H]^+$ $(C_{32}H_{50}NO_2)^+$ expected m/z 480.3836; found m/z 480.3840.

N-(4-(1-(4-methoxyphenyl)azetidin-2-yl)butyl)-4-(5-(p-tolyl)-3-(trifluoromethyl)-1H-pyrazol-1-yl)benzenesulfonamide (**7w**)

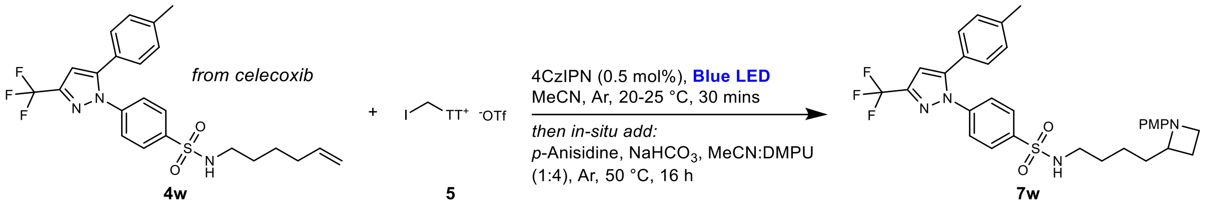

Prepared following **general procedure A**, using Celecoxib-derived olefin **4w** (1.0 equiv.; 0.2 mmol; 92.7 mg), 5-iodomethyl thianthrenium triflate **5** (1.1 equiv.; 0.22 mmol; 111.4 mg), 4CzIPN (0.005 equiv.; 0.001 mmol; 0.8 mg), *p*-Anisidine (2.0 equiv.; 0.4 mmol; 49.3 mg), and $NaHCO_3$ (3.0 equiv.;

0.6 mmol; 50.4 mg). The crude residue was purified by flash column chromatography (SiO_2; gradient 60:40 toluene:Et_2O to 50:50 toluene:Et_2O) to afford compound **7w** (95.0 mg; 79%) as a yellow oil. **Rf** (60:40 toluene:Et_2O) 0.4; **IR** (film) ν_{max}/cm^{-1}: 3291, 2931, 2859, 1617, 1597, 1510, 1471, 1444, 1409, 1374, 1333, 1271, 1236, 1161, 1133, 1096, 1038, 975, 911, 844, 843, 823, 760, 733, 629, 627, 616, 614, 573, 480; **^{1}H NMR** ($CDCl_3$, 400 MHz) δ (ppm): 7.86 – 7.78 (m, 2H), 7.48 – 7.43 (m, 2H), 7.20 – 7.14 (m, 2H), 7.13 – 7.06 (m, 2H), 6.82 – 6.76 (m, 2H), 6.74 (s, 1H), 6.48 – 6.42 (m, 2H), 4.60 (br t, 1H, NH), 3.93 – 3.80 (m, 2H), 3.74 (s, 3H), 3.52 – 3.42 (m, 1H), 3.02 – 2.90 (m, 2H), 2.38 (s, 3H), 2.30 – 2.19 (m, 1H), 2.11 – 2.00 (m, 1H), 1.91 – 1.80 (m, 1H), 1.72 – 1.62 (m, 1H), 1.56 – 1.44 (m, 2H), 1.41 – 1.29 (m, 2H); **^{13}C NMR** ($CDCl_3$, 126 MHz) δ (ppm): 152.6, 146.9, 145.4, 144.2 (q, *J* = 38.5 Hz), 142.6, 139.9, 139.6, 129.9 (2C), 128.8 (2C), 128.2 (2C), 125.8, 125.7 (2C), 121.2 (q, *J* = 269.2 Hz), 114.8 (2C), 113.5 (2C), 106.4, 65.2, 55.9, 50.6, 43.2, 36.2, 29.8, 23.5, 21.8, 21.4; **^{19}F NMR** ($CDCl_3$, 376 MHz) δ (ppm): -62.5 (s, 3F); **HRMS** (ESI-TOF) mass calculated for $[M+H]^+$ $(C_{31}H_{34}F_3N_4O_3S)^+$ expected *m/z* 599.2298; found *m/z* 599.2295.

4-(1-(4-methoxyphenyl)azetidin-2-yl)butyl-2-(11-oxo-6,11-dihydrodibenzo[b,e]oxepin-2-yl)acetate (7x)

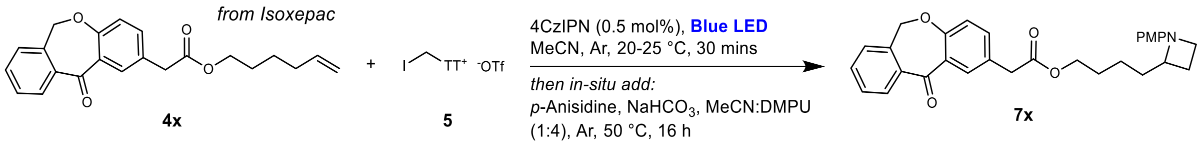

Prepared following **general procedure A**, using Isoxepac-derived olefin **4x** (1.0 equiv.; 0.2 mmol; 70.1 mg), 5-iodomethyl thianthrenium triflate **5** (1.1 equiv.; 0.22 mmol; 111.4 mg), 4CzIPN (0.005 equiv.; 0.001 mmol; 0.8 mg), *p*-Anisidine (2.0 equiv.; 0.4 mmol; 49.3 mg), and $NaHCO_3$ (3.0 equiv.; 0.6 mmol; 50.4 mg). The crude residue was purified by flash column chromatography (SiO_2; gradient 80:20 pentane:EtOAc to 70:30 pentane:EtOAc) to afford compound **7s** alongside minor impurities. The mixture was re-purified once more by flash column chromatography (SiO_2; 80:20 Toluene:Et_2O) to afford compound **7x** (49.3 mg; 51%) as a yellow oil. **Rf** (75:25 pentane:EtOAc) 0.38; **IR** (film) ν_{max}/cm^{-1}: 3059, 3039, 2931, 2857, 1732, 1648, 1612, 1599, 1510, 1490, 1455, 1413, 1379, 1300, 1238, 1163, 1139, 1119, 1039, 1016, 943, 821, 800, 761, 698, 641, 612, 530; **^{1}H NMR** ($CDCl_3$, 500 MHz) δ (ppm): 8.13 (d, *J* = 2.4 Hz, 1H), 7.89 (dd, *J* = 7.8, 1.4 Hz, 1H), 7.56 (td, *J* = 7.4, 1.4 Hz, 1H), 7.46 (td, *J* = 7.6, 1.3 Hz, 1H), 7.43 (dd, *J* = 8.4, 2.4 Hz, 1H), 7.36 (dd, *J* = 7.5, 0.7 Hz, 1H), 7.03 (d, *J* = 8.4 Hz, 1H), 6.82 – 6.76 (m, 2H), 6.49 – 6.44 (m, 2H), 5.17 (s, 2H), 4.13 (t, *J* = 6.6 Hz, 2H), 3.89 – 3.80 (m, 2H), 3.74 (s, 3H), 3.64 (s, 2H), 3.49 – 3.42 (m, 1H), 2.29 – 2.20 (m, 1H), 2.10 – 2.00 (m, 1H), 1.97 – 1.88 (m, 1H), 1.75 – 1.64 (m, 3H), 1.46 – 1.30 (m, 2H); **^{13}C NMR** ($CDCl_3$, 126 MHz) δ (ppm): 190.9, 171.6, 160.6, 152.4, 147.2, 140.6, 136.5, 135.7, 132.9, 132.6, 129.6, 129.4, 128.1, 127.9, 125.3, 121.2, 114.8 (2C), 113.3 (2C), 73.7, 65.2, 65.0, 55.9, 50.5, 40.4, 36.7, 28.8, 23.7, 21.4;

HRMS (ESI-TOF) mass calculated for $[M+H]^+$ $(C_{30}H_{32}NO_5)^+$ expected *m/z* 486.2275; found *m/z* 486.2277.

3-(1-(4-methoxyphenyl)azetidin-2-yl)propyl-2-(3-cyano-4-isobutoxyphenyl)-4-methylthiazole-5-carboxylate (**7y**)

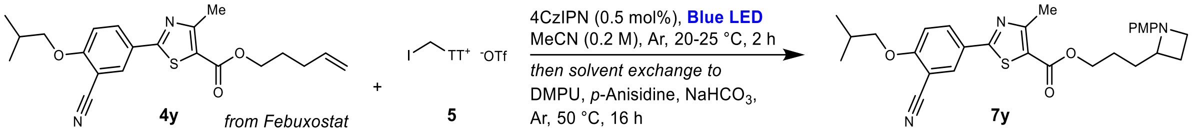

Prepared following **modified general procedure A**; An oven-dried Schlenk tube with atmosphere exchanged to Ar was charged with 5-iodomethyl thianthrenium triflate **5** (1.1 equiv.; 0.22 mmol; 111.4 mg), 4CzIPN (0.005 equiv.; 0.001 mmol; 0.8 mg) and febuxostat-derived olefin **4y** (1.0 equiv.; 0.20 mmol; 76.9 mg), followed by degassed dry MeCN (1.0 mL, 0.2 M, previously degassed through 10 min argon sparging). The vessel was sealed and placed in a glass-wall water bath where a fan was circulating air to maintain the water bath temperature of 20 - 25 °C. See Fig. S2 for visual details of the reaction setup. The reaction was irradiated through the glass wall with blue light (Kessil lamp A160WE Tuna Blue Saltwater LED Light 40 W) for 2 h under moderate stirring (500 rpm). The vessel was then taken out from the water bath and solvent was removed under vacuum using a standard Schlenk manifold (ensuring that no MeCN remains). *p*-Anisidine (2.0 equiv.; 0.4 mmol; 49.3 mg), $NaHCO_3$ (3.0 equiv.; 0.6 mmol; 50.4 mg), and dry DMPU (2 mL) were introduced into the vessel. The mixture was stirred (700 rpm) at 50℃ without irradiation for 16 h. The reaction mixture was then diluted with EtOAc (60 mL) and washed with distilled water (5×25 mL) and brine (25 mL). The organic layer was dried over anhydrous Na_2SO_4, filtered and concentrated under reduced pressure. The crude residue was purified by flash column chromatography (SiO_2; gradient 90:10 Toluene: Et_2O to 85:15 Toluene: Et_2O) to afford compound **7y** (55.1 mg; 53%) as a yellow oil. **R_f** (80:20 Toluene :Et_2O) 0.30; **IR** (film) ν_{max}/cm^{-1}: 2959, 2931, 2871, 2228, 1712, 1507, 1256, 1235, 1090, 819; **^{1}H NMR** ($CDCl_3$, 500 MHz) δ (ppm): 8.17 (d, J = 2.3 Hz, 1H), 8.08 (dd, J = 8.8, 2.3 Hz, 1H), 7.01 (d, J = 8.9 Hz, 1H), 6.83 – 6.76 (m, 2H), 6.55 – 6.38 (m, 2H), 4.39 – 4.30 (m, 2H), 4.00 – 3.87 (m, 4H), 3.73 (s, 3H), 3.56 – 3.47 (m, 1H), 2.77 (s, 3H), 2.37 – 2.28 (m, 1H), 2.25 – 2.10 (m, 2H), 2.08 – 1.98 (m, 1H), 1.92 – 1.77 (m, 3H), 1.09 (d, J = 6.7 Hz, 6H).; **^{13}C NMR** ($CDCl_3$, 126 MHz) δ (ppm): 167.4, 162.6, 162.1, 161.4, 152.5, 147.0, 132.7, 132.2, 126.1, 121.8, 115.5, 114.8, 113.2, 112.7, 103.1, 75.8, 65.3, 64.6, 55.9, 50.4, 33.2, 28.3, 24.2, 23.5, 19.2, 17.6; **HRMS** (ESI-TOF) mass calculated for $[M+H]^+$ $(C_{29}H_{34}N_3O_4S)^+$ expected *m/z* 520.2265; found *m/z* 520.2262.

2-Phenethyl-1-phenylazetidine (7aa)

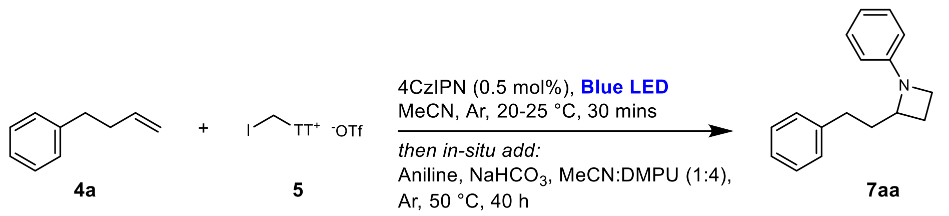

Prepared following **modified general procedure A**, to an oven-dried Schlenk tube under an Ar atmosphere, 5-iodomethyl thianthrenium triflate 5 (1.1 equiv.; 0.22 mmol; 111.4 mg), and 4CzIPN (0.005 equiv.; 0.001 mmol; 0.8 mg) were introduced followed by degassed dry MeCN (0.4 mL, 0.5 M, previously degassed through 10 min argon sparging) through a syringe. Then 4-Phenyl-1-butene **4a** (1.0 equiv.; 0.2 mmol; 30.0 μL) was added, the vessel was sealed with a glass stopper using silicon grease and was placed in a glasswall water bath where a fan was blowing air to keep the water temperature between 20 - 25 °C (monitored using a thermometer). See Fig. S2 for visual details of the reaction setup. The reaction was irradiated through the glass wall with blue light (Kessil lamp A160WE Tuna Blue Saltwater LED Light 40 W) for 30 mins under moderate stirring (500 rpm). The vessel was then removed from the water bath and Aniline (2.0 equiv.; 0.4 mmol; 37.3 mg), $NaHCO_3$ (3.0 equiv.; 0.6 mmol; 50.4 mg), and dry 1,3-Dimethyl-3,4,5,6-tetrahydro-2(1H)-pyrimidinone (DMPU, 1.6 mL) were introduced into the vessel. The mixture was stirred (500 rpm) at 50 °C without irradiation for 40 h. The mixture was diluted with 60 mL of EtOAc, washed with saturated sodium carbonate solution (1×25mL) distilled water (5×25 mL) and brine (25 mL). The organic layer was dried over anhydrous Na_2SO_4 and concentrated under reduced pressure. The crude residue was purified by flash column chromatography (SiO_2; gradient 100 pentane to 97:3 pentane:EtOAc) to afford compound **7aa** (31.9 mg; 67%) as a yellow oil. **R_f** (97:3 pentane:EtOAc) 0.5; **IR** (film) ν_{max}/cm^{-1}: 3025, 2924, 2856, 1599, 1499, 1475, 1454, 1333, 750, 693; **^{1}H NMR** ($CDCl_3$, 500 MHz) δ (ppm): 7.35 – 7.28 (m, 2H), 7.26 – 7.17 (m, 5H), 6.81 – 6.66 (m, 1H), 6.58 – 6.42 (m, 2H), 4.09 – 4.01 (m, 1H), 3.99 – 3.92 (m, 1H), 3.63 – 3.54 (m, 1H), 2.79 – 2.72 (m, 1H), 2.71 – 2.63 (m, 1H), 2.38 – 2.27 (m, 2H), 2.19 – 2.13 (m, 1H), 2.12 – 2.03 (m, 1H); **^{13}C NMR** ($CDCl_3$, 126 MHz) δ (ppm): 152.5, 142.0, 129.0, 128.6, 128.5, 126.0, 117.8, 112.0, 64.6, 50.0, 38.4, 31.3, 23.6; **HRMS** (ESI-TOF) mass calculated for $[M+H]^+$ $(C_{17}H_{20}N)^+$ expected *m/z* 238.1590; found *m/z* 268.1590.

Methyl 3-(2-phenethylazetidin-1-yl)benzoate (**7ab**)

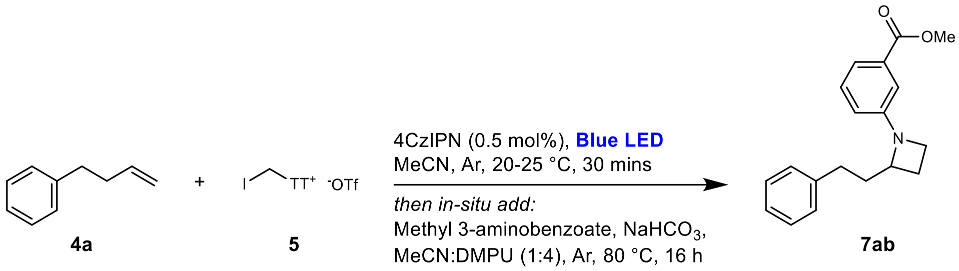

Prepared following **modified general procedure A**, to an oven-dried Schlenk tube under an Ar atmosphere, 5-iodomethyl thianthrenium triflate 5 (1.1 equiv.; 0.22 mmol; 111.4 mg), and 4CzIPN (0.005 equiv.; 0.001 mmol; 0.8 mg) were introduced followed by degassed dry MeCN (0.4 mL, 0.5 M, previously degassed through 10 min argon sparging) through a syringe. Then 4-Phenyl-1-butene **4a** (1.0 equiv.; 0.2 mmol; 30.0 µL) was added, the vessel was sealed with a glass stopper using silicon grease and was placed in a glasswall water bath where a fan was blowing air to keep the water temperature between 20 - 25 °C (monitored using a thermometer). See Fig. S2 for visual details of the reaction setup. The reaction was irradiated through the glass wall with blue light (Kessil lamp A160WE Tuna Blue Saltwater LED Light 40 W) for 30 mins under moderate stirring (500 rpm). The vessel was then removed from the water bath and Methyl 3-aminobenzoate (5.0 equiv.; 1.0 mmol; 151.2 mg), $NaHCO_3$ (3.0 equiv.; 0.6 mmol; 50.4 mg), and dry 1,3-Dimethyl-3,4,5,6-tetrahydro-2(1H)-pyrimidinone (DMPU, 1.6 mL) were introduced into the vessel. The mixture was stirred (500 rpm) at 80 °C without irradiation for 16 h. The mixture was diluted with 60 mL of EtOAc, washed with saturated sodium carbonate solution (1×25mL) distilled water (5×25 mL) and brine (25 mL). The organic layer was dried over anhydrous Na_2SO_4 and concentrated under reduced pressure. The crude residue was purified by flash column chromatography (SiO_2; gradient 100 pentane to 93:7 pentane:EtOAc) to afford compound **7ab** (31.7 mg; 54%) as a yellow oil. **R_f** (93:7 pentane:EtOAc) 0.5; **IR** (film) ν_{max}/cm^{-1}: 3026, 2949, 2858, 1720, 1602, 1580, 1491, 1451, 1343, 1253, 1106, 753, 700; **^{1}H NMR** ($CDCl_3$, 500 MHz) δ (ppm): 7.41 – 7.38 (m, 1H), 7.32 – 7.29 (m, 2H), 7.25 – 7.19 (m, 4H), 7.15 – 7.14 (m, 1H), 6.67 – 6.65 (m, 1H), 4.12 – 4.06 (m, 1H), 4.02 – 3.98 (m, 1H), 3.90 (s, 3H), 3.65 – 3.60 (m, 1H), 2.78 – 2.65 (m, 2H), 2.40 – 2.29 (m, 2H), 2.20 – 2.13 (m, 1H), 2.12 – 2.03 (m, 1H); **^{13}C NMR** ($CDCl_3$, 126 MHz) δ (ppm): 167.7, 152.3, 141.8, 130.9, 129.0, 128.6, 128.5, 126.1, 118.8, 116.2, 112.7, 64.6, 52.2, 50.1, 38.1, 31.2, 23.6; **HRMS** (ESI-TOF) mass calculated for $[M+H]^+$ $(C_{19}H_{22}NO_2)^+$ expected *m/z* 296.1645; found *m/z* 296.1648.

Ethyl 4-(2-phenethylazetidin-1-yl)benzoate (**7ac**)

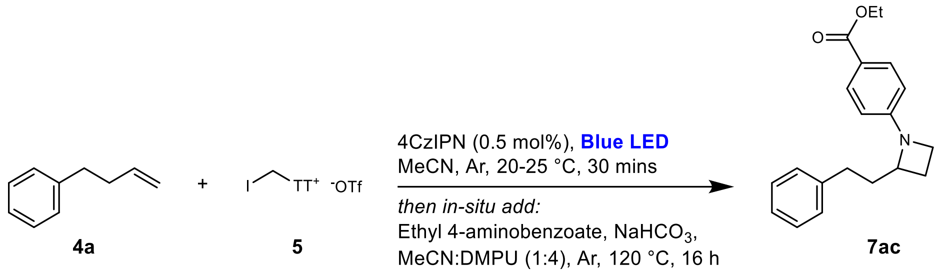

Prepared following **modified general procedure A**, to an oven-dried Schlenk tube under an Ar atmosphere, 5-iodomethyl thianthrenium triflate 5 (1.1 equiv.; 0.22 mmol; 111.4 mg), and 4CzIPN (0.005 equiv.; 0.001 mmol; 0.8 mg) were introduced followed by degassed dry MeCN (0.4 mL, 0.5 M, previously degassed through 10 min argon sparging) through a syringe. Then 4-Phenyl-1-butene **4a** (1.0 equiv.; 0.2 mmol; 30.0 µL) was added, the vessel was sealed with a glass stopper using silicon grease and was placed in a glasswall water bath where a fan was blowing air to keep the water temperature between 20 - 25 °C (monitored using a thermometer). See Fig. S2 for visual details of the reaction setup. The reaction was irradiated through the glass wall with blue light (Kessil lamp A160WE Tuna Blue Saltwater LED Light 40 W) for 30 mins under moderate stirring (500 rpm). The vessel was then removed from the water bath and Ethyl 4-aminobenzoate (5.0 equiv.; 1.0 mmol; 165.2 mg), $NaHCO_3$ (3.0 equiv.; 0.6 mmol; 50.4 mg), and dry 1,3-Dimethyl-3,4,5,6-tetrahydro-2(1H)-pyrimidinone (DMPU, 1.6 mL) were introduced into the vessel. The mixture was stirred (500 rpm) at 120 °C without irradiation for 16 h. The mixture was diluted with 60 mL of EtOAc, washed with saturated sodium carbonate solution (1×25mL) distilled water (5×25 mL) and brine (25 mL). The organic layer was dried over anhydrous Na_2SO_4 and concentrated under reduced pressure. The crude residue was purified by flash column chromatography (SiO_2; gradient 100 pentane to 94:6 pentane:EtOAc) to afford compound **7ac** (23.5 mg; 38%) as a yellow oil. **R_f** (94:6 pentane:EtOAc) 0.5; **IR** (film) ν_{max}/cm^{-1}: 2930, 2861, 1701, 1604, 1519, 1364, 1272, 1170, 1105, 1027, 836, 771, 700; **^{1}H NMR** ($CDCl_3$, 500 MHz) δ (ppm): 7.92 – 7.82 (m, 2H), 7.35 – 7.27 (m, 2H), 7.24 – 7.17 (m, 3H), 6.43 – 6.37 (m, 2H), 4.32 (q, *J* = 7.1 Hz, 2H), 4.22 – 4.15 (m, 1H), 4.04 – 3.99 (m, 1H), 3.75 – 3.67 (m, 1H), 2.78 – 2.62 (m, 2H), 2.47 – 2.40 (m, 1H), 2.35 – 2.26 (m, 1H), 2.21 – 2.13 (m, 1H), 2.11 – 2.03 (m, 1H), 1.36 (t, *J* = 7.1 Hz, 3H); **^{13}C NMR** ($CDCl_3$, 126 MHz) δ (ppm): 167.1, 154.8, 141.6, 131.2, 128.6, 128.4, 126.2, 118.7, 110.4, 64.1, 60.3, 49.5, 37.5, 31.0, 23.3, 14.6; **HRMS** (ESI-TOF) mass calculated for $[M+H]^+$ $(C_{20}H_{24}NO_2)^+$ expected *m/z* 310.1802; found *m/z* 310.1800.

5-(2-phenethylazetidin-1-yl)-1H-indole (**7ad**)

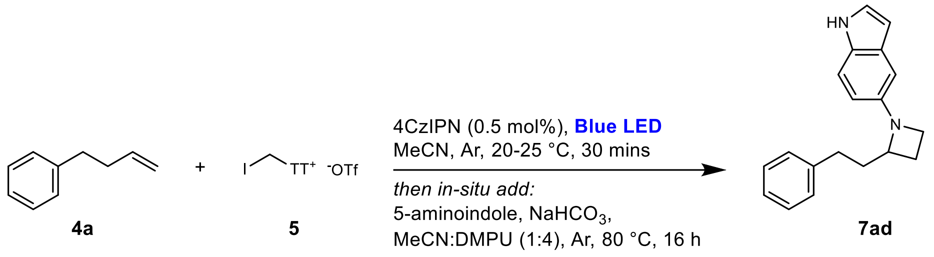

Prepared following **modified general procedure A**, to an oven-dried Schlenk tube under an Ar atmosphere, 5-iodomethyl thianthrenium triflate 5 (1.1 equiv.; 0.22 mmol; 111.4 mg), and 4CzIPN (0.005 equiv.; 0.001 mmol; 0.8 mg) were introduced followed by degassed dry MeCN (0.4 mL, 0.5 M, previously degassed through 10 min argon sparging) through a syringe. Then 4-Phenyl-1-butene **4a** (1.0 equiv.; 0.2 mmol; 30.0 µL) was added, the vessel was sealed with a glass stopper using silicon grease and was placed in a glasswall water bath where a fan was blowing air to keep the water temperature between 20 - 25 °C (monitored using a thermometer). See Fig. S2 for visual details of the reaction setup. The reaction was irradiated through the glass wall with blue light (Kessil lamp A160WE Tuna Blue Saltwater LED Light 40 W) for 30 mins under moderate stirring (500 rpm). The vessel was then removed from the water bath and 5-aminoindole (4.0 equiv.; 0.8 mmol; 105.7 mg), $NaHCO_3$ (3.0 equiv.; 0.6 mmol; 50.4 mg), and dry 1,3-Dimethyl-3,4,5,6-tetrahydro-2(1H)-pyrimidinone (DMPU, 1.6 mL) were introduced into the vessel. The mixture was stirred (500 rpm) at 80 °C without irradiation for 16 h. The mixture was diluted with 60 mL of EtOAc, washed with saturated sodium carbonate solution (1×25mL) distilled water (5×25 mL) and brine (25 mL). The organic layer was dried over anhydrous Na_2SO_4 and concentrated under reduced pressure. The crude residue was purified by flash column chromatography (SiO_2; gradient 88:12 pentane:EtOAc to 78:22 pentane:EtOAc) to afford compound **7ad** (32.6 mg; 59%) as a yellow oil. **R_f** (78:22 pentane:EtOAc) 0.5; **IR** (film) ν_{max}/cm^{-1}: 3412, 3025, 2926, 2856, 1625, 1577, 1473, 1454, 1319, 1240, 1177, 754, 721, 699; **^{1}H NMR** ($CDCl_3$, 500 MHz) δ (ppm): 7.94 (brs, 1H), 7.33 – 7.29 (m, 2H), 7.28 – 7.17 (m, 4H), 7.13 (t, *J* = 2.8 Hz, 1H), 6.74 (d, *J* = 2.2 Hz, 1H), 6.55 (dd, *J* = 8.7, 2.2 Hz, 1H), 6.44 – 6.36 (m, 1H), 4.03 – 3.96 (m, 2H), 3.59 – 3.53 (m, 1H), 2.81 – 2.74 (m, 1H), 2.73 – 2.66 (m, 1H), 2.41 – 2.27 (m, 2H), 2.19 – 2.07 (m, 2H); **^{13}C NMR** ($CDCl_3$, 126 MHz) δ (ppm): 147.3, 142.2, 130.4, 128.6, 128.5, 128.5, 126.0, 124.7, 111.5, 110.1, 102.3, 102.0, 65.2, 51.0, 38.7, 31.5, 23.9; **HRMS** (ESI-TOF) mass calculated for $[M+H]^+$ $(C_{19}H_{21}N_2)^+$ expected *m/z* 277.1699; found *m/z* 277.1702.

1-benzyl-2-phenethylazetidine (7ae)

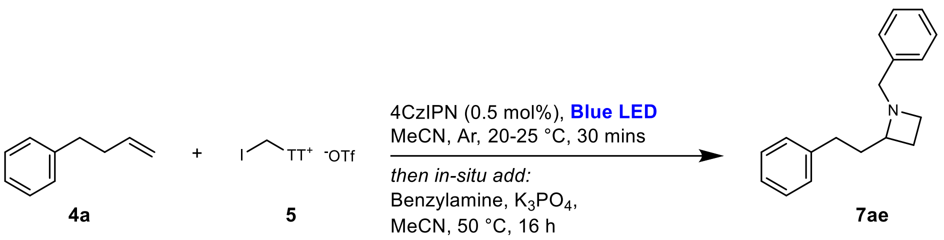

Prepared following **general procedure B**, using 4-Phenyl-1-butene **4a** (1.0 equiv.; 0.2 mmol; 30.0 µL), 5-iodomethyl thianthrenium triflate **5** (1.1 equiv.; 0.22 mmol; 111.4 mg), 4CzIPN (0.005 equiv.; 0.001 mmol; 0.8 mg), benzylamine (3.0 equiv.; 0.6 mmol; 65.5 µL), and K_3PO_4 (3.0 equiv.; 0.6 mmol; 127.4 mg). The crude residue was purified by flash column chromatography (SiO_2; gradient 98:2 chloroform: $NH_{3\ (MeOH)}$ to 90:10 chloroform: $NH_{3\ (MeOH)}$ *then* SiO_2 deactivated by method (i) (*vide supra*) and gradient 100 pentane to 70:30 pentane:acetone) to afford compound **7ae** (30.5 mg; 61%) as a pale yellow oil. **R$_f$** (95:5 chloroform: $NH_{3(MeOH)}$) 0.4; **IR** (film) ν_{max}/cm^{-1}: 3085, 3061, 3026, 2985, 2926, 2852, 1603, 1495, 1453, 1359, 733, 698; **^{1}H NMR** ($CDCl_3$, 500 MHz) δ (ppm): δ 7.31 – 7.24 (m, 7H), 7.20 – 7.10 (m, 3H), 3.70 (d, J = 12.5 Hz, 1H), 3.49 (d, J = 12.6 Hz, 1H), 3.39 – 3.29 (m, 1H), 3.24 – 3.12 (m, 1H), 2.89 – 2.76 (m, 1H), 2.65 – 2.46 (m, 2H), 2.06 – 1.99 (m, 1H), 1.91 – 1.71 (m, 3H). **^{13}C NMR** ($CDCl_3$, 126 MHz) δ (ppm): 142.3, 138.5, 129.1, 128.4, 128.4, 128.4, 127.2, 125.8, 66.7, 63.2, 51.8, 37.8, 31.8, 24.1; **HRMS** (ESI-TOF) mass calculated for $[M+H]^+$ $(C_{18}H_{22}N)^+$ expected *m/z* 252.1747; found *m/z* 252.1748.

Note: $NH_{3(MeOH)}$ refers to a 2M solution of ammonia in methanol made using 35% aqueous ammonia solution. In addition, silica plates for crude mixture evaluation were first dipped in this solution and dried before use.

1-(4-methoxybenzyl)-2-phenethylazetidine (7af)

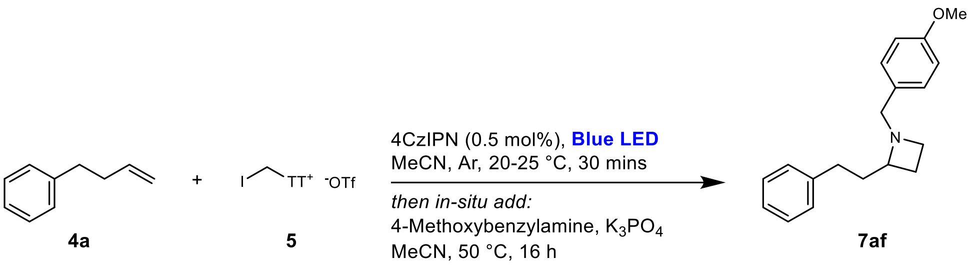

Prepared following **general procedure B**, using 4-Phenyl-1-butene **4a** (1.0 equiv.; 0.2 mmol; 30.0 µL), 5-iodomethyl thianthrenium triflate **5** (1.1 equiv.; 0.22 mmol; 111.4 mg), 4CzIPN (0.005 equiv.; 0.001 mmol; 0.8 mg), 4-Methoxybenzylamine (3.0 equiv.; 0.6 mmol; 78.4 µL), and K_3PO_4 (3.0 equiv.; 0.6 mmol; 127.4 mg). The crude residue was purified by flash column chromatography

(deactivated SiO_2 (*method i*); and gradient 100 pentane to 70:30 pentane:acetone) to afford compound **7af** (32.1 mg; 57%) as a pale yellow oil. **R$_f$** (80:20 pentane: acetone) 0.4; **IR** (film) ν_{max}/cm^{-1}: 3061, 3026, 2992, 2931, 2833, 1612, 1511, 1454, 1246, 1171, 1036, 699; **^{1}H NMR** ($CDCl_3$, 500 MHz) δ (ppm): δ 7.29 – 7.24 (m, 2H), 7.23 – 7.15 (m, 3H), 7.15 – 7.09 (m, 2H), 6.87 – 6.74 (m, 2H), 3.79 (s, 3H), 3.62 (d, *J* = 12.4 Hz, 1H), 3.42 (d, *J* = 12.4 Hz, 1H), 3.35 – 3.23 (m, 1H), 3.21 – 3.09 (m, 1H), 2.85 – 2.72 (m, 1H), 2.61 – 2.44 (m, 2H), 2.05 – 1.98 (m, 1H), 1.92 – 1.68 (m, 3H). **^{13}C NMR** ($CDCl_3$, 126 MHz) δ (ppm): 158.8, 142.3, 130.7, 130.2, 128.4, 128.4, 125.8, 113.8, 66.5, 62.6, 55.4, 51.5, 37.9, 31.9, 24.1; **HRMS** (ESI-TOF) mass calculated for $[M+H]^+$ $(C_{19}H_{24}NO)^+$ expected *m/z* 282.1852; found *m/z* 282.1850.

1-cyclohexyl-2-phenethylazetidine (**7ag**)

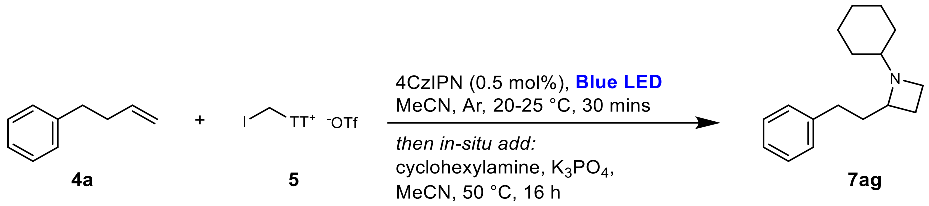

Prepared following **general procedure B**, using 4-Phenyl-1-butene **4a** (1.0 equiv.; 0.2 mmol; 30.0 μL), 5-iodomethyl thianthrenium triflate **5** (1.1 equiv.; 0.22 mmol; 111.4 mg), 4CzIPN (0.005 equiv.; 0.001 mmol; 0.8 mg), cyclohexylamine (4.0 equiv.; 0.8 mmol; 92 μL), and K_3PO_4 (3.0 equiv.; 0.6 mmol; 127.4 mg). The crude residue was purified by flash column chromatography (deactivated SiO_2 (*method ii*); gradient 95:5 pentane:acetone to 90:10 pentane:acetone) to afford compound **7ag** (27.8 mg; 57%) as a transparent oil. **R$_f$** (90:10 pentane:acetone) 0.5; **IR** (film) ν_{max}/cm^{-1}: 3026, 2926, 2853, 2818, 1603, 1496, 1449, 1362, 1293, 1228, 1195, 1092, 1030, 746, 698 ; **^{1}H NMR** ($CDCl_3$, 500 MHz) δ (ppm): 7.30 – 7.23 (m, 2H), 7.21 – 7.13 (m, 3H), 3.39 – 3.32 (m, 1H), 3.13 – 3.03 (m, 1H), 2.77 – 2.68 (m, 1H), 2.64 – 2.54 (m, 1H), 2.51 – 2.41 (m, 1H), 2.06 – 1.95 (m, 3H), 1.94 – 1.83 (m, 1H), 1.83 – 1.75 (m, 2H), 1.72 – 1.66 (m, 3H), 1.62 – 1.53 (m, 1H), 1.24 – 1.07 (m, 3H), 1.06 – 0.90 (m, 2H); **^{13}C NMR** ($CDCl_3$, 126 MHz) δ (ppm): 142.3, 128.44, 128.42, 125.8, 67.3, 65.4, 49.8, 39.9, 31.8, 31.6, 30.2, 26.1, 24.80, 24.78, 23.4; **HRMS** (ESI) mass calculated for $[M+H]^+$ $(C_{17}H_{26}N^+)$ expected *m/z* 244.2060; found *m/z* 244.2061.

2-phenethyl-1-(tetrahydro-2H-pyran-4-yl)azetidine (**7ah**)

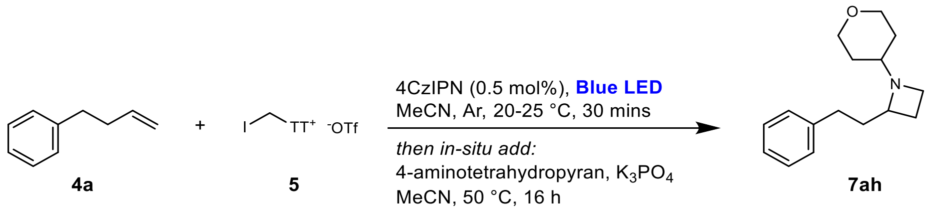

Prepared following **general procedure B**, using 4-Phenyl-1-butene **4a** (1.0 equiv.; 0.2 mmol; 30.0 µL), 5-iodomethyl thianthrenium triflate **5** (1.1 equiv.; 0.22 mmol; 111.4 mg), 4CzIPN (0.005 equiv.; 0.001 mmol; 0.8 mg), 4-aminotetrahydropyran (4.0 equiv.; 0.8 mmol; 83 µL), and K_3PO_4 (3.0 equiv.; 0.6 mmol; 127.4 mg). The crude residue was purified by flash column chromatography (deactivated SiO_2 (*method ii*); gradient 95:5 pentane:acetone to 50:50 pentane:acetone) to afford compound **7ah** (28.3 mg; 58%) as a transparent oil. **R**$_f$ (50:50 pentane:acetone) 0.5; **IR** (film) ν_{max}/cm^{-1}: 3062, 3026, 2938, 2840, 1454, 1301, 1091, 913, 748, 699; **^{1}H NMR** ($CDCl_3$, 500 MHz) δ (ppm): 7.31 – 7.23 (m, 2H), 7.21 – 7.13 (m, 3H), 3.98 – 3.88 (m, 2H), 3.40 – 3.29 (m, 3H), 3.17 – 3.08 (m, 1H), 2.80 – 2.71 (m, 1H), 2.66 – 2.57 (m, 1H), 2.51 – 2.42 (m, 1H), 2.30 – 2.21 (m, 1H), 2.07 – 1.99 (m, 1H), 1.99 – 1.86 (m, 2H), 1.86 – 1.78 (m, 1H), 1.66 – 1.56 (m, 2H), 1.45 – 1.28 (m, 2H); **^{13}C NMR** ($CDCl_3$, 126 MHz) δ (ppm): 142.1, 128.5, 128.4, 125.9, 66.7, 66.4, 65.1, 63.9, 49.3, 39.6, 31.9, 31.7, 30.4, 23.4; **HRMS** (ESI) mass calculated for $[M+H]^+$ ($C_{16}H_{24}NO^+$) expected *m/z* 246.1852; found *m/z* 246.1854.

1-cyclopropyl-2-phenethylazetidine (**7ai**)

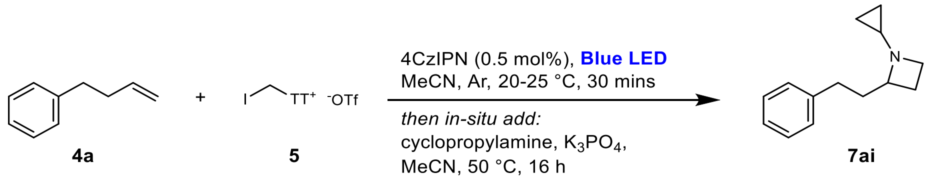

Prepared following **general procedure B**, using 4-Phenyl-1-butene **4a** (1.0 equiv.; 0.2 mmol; 30.0 µL), 5-iodomethyl thianthrenium triflate **5** (1.1 equiv.; 0.22 mmol; 111.4 mg), 4CzIPN (0.005 equiv.; 0.001 mmol; 0.8 mg), cyclopropylamine (4.0 equiv.; 0.8 mmol; 55 µL), and K_3PO_4 (3.0 equiv.; 0.6 mmol; 127.4 mg). The crude residue was purified by flash column chromatography (deactivated SiO_2 (*method ii*); gradient 98:2 pentane:acetone to 95:5 pentane:acetone) to afford compound **7ai** (23.5 mg; 59%) as a transparent oil. **R**$_f$ (95:5 pentane:acetone) 0.5; **IR** (film) ν_{max}/cm^{-1}: 3086, 3007, 2921, 2831, 1603, 1495, 1363, 1057, 1013, 879, 819, 747, 697; **^{1}H NMR** ($CDCl_3$, 500 MHz) δ (ppm): 7.33 – 7.22 (m, 2H), 7.20 – 7.16 (m, 3H), 3.39 – 3.20 (m, 2H), 3.02 – 2.91 (m, 1H), 2.69 – 2.52 (m, 2H), 2.07 – 1.93 (m, 2H), 1.84 – 1.70 (m, 3H), 0.48 – 0.38 (m, 2H), 0.38 – 0.26 (m, 2H); **^{13}C NMR** ($CDCl_3$, 126 MHz) δ (ppm): 142.4, 128.5, 128.4, 125.8, 67.1, 51.0, 38.2, 37.7, 32.3, 24.1, 6.4, 3.8; **HRMS** (ESI) mass calculated for $[M+H]^+$ ($C_{14}H_{20}N^+$) expected *m/z* 202.1590; found *m/z* 202.1591.

3-(2-phenethylazetidin-1-yl)propan-1-ol (**7aj**)

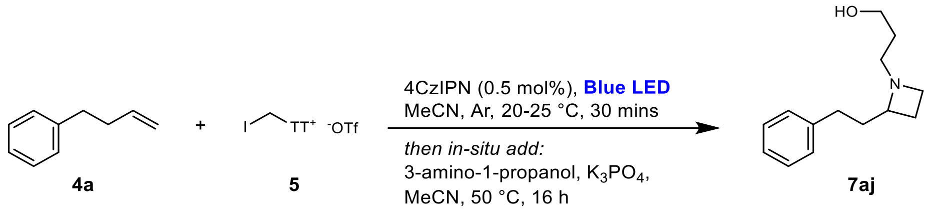

Prepared following **general procedure B**, using 4-Phenyl-1-butene **4a** (1.0 equiv.; 0.2 mmol; 30.0 µL), 5-iodomethyl thianthrenium triflate **5** (1.1 equiv.; 0.22 mmol; 111.4 mg), 4CzIPN (0.005 equiv.; 0.001 mmol; 0.8 mg), 3-amino-1-propanol (4.0 equiv.; 0.8 mmol; 61 µL), and K_3PO_4 (3.0 equiv.; 0.6 mmol; 127.4 mg). The crude residue was purified by flash column chromatography (deactivated SiO_2 (*method ii*); gradient 98:2 $CHCl_3$:[2M NH_3 in MeOH] to 90:10 [2M NH_3 in MeOH]) followed by further purification by preparative TLC (90:10 $CHCl_3$:[2M NH_3 in MeOH]) to afford compound **7aj** (21.0 mg; 48%) as a transparent oil. **R_f** (90:10 $CHCl_3$:[2M NH_3 in MeOH]) 0.5; **IR** (film) ν_{max}/cm^{-1}: 3328 (br), 2928, 2850, 1603, 1496, 1454, 1372, 1187, 1166, 1097, 1067, 1030, 748, 699; **^{1}H NMR** ($CDCl_3$, 500 MHz) δ (ppm): 7.32 – 7.23 (m, 2H), 7.22 – 7.13 (m, 3H), 3.80 – 3.72 (m, 2H), 3.55 – 3.36 (m, 1H), 3.11 – 2.99 (m, 1H), 2.86 – 2.77 (m, 1H), 2.77 – 2.66 (m, 1H), 2.63 – 2.53 (m, 3H), 2.09 – 1.94 (m, 2H), 1.89 – 1.73 (m, 2H), 1.71 – 1.58 (m, 1H), 1.53 – 1.41 (m, 1H); **^{13}C NMR** ($CDCl_3$, 126 MHz) δ (ppm): 142.0, 128.50, 128.45, 126.0, 66.9, 64.6, 59.2, 51.8, 38.1, 31.8, 28.4, 23.7; **HRMS** (ESI) mass calculated for $[M+H]^+$ ($C_{14}H_{22}NO^+$) expected *m/z* 220.1696; found *m/z* 220.1697.

2-phenethylazetidine trifluoroacetic acid salt (**7ba**)

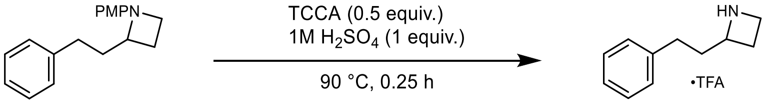

The *p*-methoxyphenyl (PMP) group was removed according to a modified version of a literature procedure.[14]

To a solution of protected azetidine **7a** (1.0 equiv., 0.2 mmol, 53.5 mg) in MeCN/H_2O (1:1, 0.05 M, 4 mL) in a 30 mL microwave vial were added trichloroisocyanuric acid (0.5 equiv., 0.1 mmol, 23.2 mg) and 1M aqueous H_2SO_4 (1 equiv., 0.2 mmol, 200 µL). The vial was then sealed, and the mixture was stirred at 90 ℃ for 0.25 h. The reaction mixture was diluted with 5 mL H_2O and washed with DCM (3×20 mL). The resultant aqueous layer was basified to pH 10.5 with 5M KOH and extracted with DCM (5×20 mL). The combined organic layers were acidified with trifluoroacetic acid (1 mL) and concentrated to afford the crude TFA salt. The crude residue was purified by flash column chromatography (SiO_2; gradient 100% $CHCl_3$ to 70:30 $CHCl_3$:MeOH) to afford compound **7ba** (42.9 mg; 78%) as a pale brown oil. **R_f** (80:20 $CHCl_3$:MeOH) 0.2; **IR** (film) ν_{max}/cm^{-1}: 3365, 2960, 2644,

2444, 1670, 1603, 1497, 1453, 1390, 1319, 1204, 1178, 1130, 800, 722, 700; **^{1}H NMR** (CD_3OD, 500 MHz) δ (ppm): 7.34 – 7.26 (m, 2H), 7.26 – 7.13 (m, 3H), 4.50 – 4.38 (m, 1H), 4.09 – 4.00 (m, 1H), 3.91 – 3.81 (m, 1H), 2.71 – 2.59 (m, 2H), 2.57 – 2.47 (m, 1H), 2.38 – 2.29 (m, 1H), 2.28 – 2.20 (m, 1H), 2.20 – 2.11 (m, 1H); **^{13}C NMR** (CD_3OD, 126 MHz) δ (ppm): 141.3, 129.7, 129.4, 127.5, 62.2, 44.0, 36.6, 31.8, 26.0; **^{19}F NMR** (CD_3OD, 376 MHz) δ (ppm): -77.03; **HRMS** (ESI-TOF) mass calculated for $[M]^+$ $(C_{11}H_{16}N)^+$ expected *m/z* 162.1277; found *m/z* 162.1278.

9-(azetidin-2-yl)nonanamide (**7bb**)

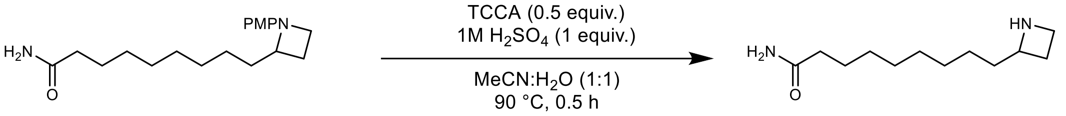

The *p*-methoxyphenyl (PMP) group was removed according to a modified version of a literature procedure.[14]

To a solution of protected azetidine **7d** (1.0 equiv., 0.1 mmol, 31.9 mg) in $MeCN/H_2O$ (1:1, 0.05 M, 2 mL) in a 30 mL microwave vial were added trichloroisocyanuric acid (0.5 equiv., 0.1 mmol, 11.6 mg) and 1M aqueous H_2SO_4 (1 equiv., 0.1 mmol, 100 µL). The vial was then sealed, and the mixture was stirred at 90 °C for 0.5 h. The reaction mixture was diluted with 5 mL H_2O and washed with DCM (4×10 mL). The resultant aqueous layer was basified to pH 10.5 with 5M KOH and extracted with DCM (5×10 mL). The aqueous layer was then saturated with NaCl and further extracted with DCM (3×20 mL). The combined organic layers were dried over anhydrous Na_2SO_4, filtered, and subsequently concentrated. The crude residue was purified by flash column chromatography (SiO_2; gradient 95:5 $CHCl_3$:MeOH to 80:20 $CHCl_3$:MeOH) to afford compound **7bb** (17.0 mg; 80%) as an off white solid. **R_f** (80:20 $CHCl_3$:MeOH) 0.2; **M.P.**: 96.3-99.2 °C; **IR** (film) ν_{max}/cm^{-1}: 3351, 3187, 2924, 2853, 1660, 1531, 1404, 1363, 909, 726, 643; **^{1}H NMR** ($CDCl_3$, 500 MHz) δ (ppm): 5.61 (bs, 2H), 3.91 – 3.75 (m, 1H), 3.64 – 3.53 (m, 1H), 3.38 – 3.30 (m, 1H), 2.57 (bs, 1H), 2.27 – 2.18 (m, 3H), 2.02 – 1.95 (m, 1H), 1.63 – 1.49 (m, 4H), 1.31 – 1.21 (m, 10H); **^{13}C NMR** ($CDCl_3$, 126 MHz) δ (ppm): 175.8, 59.7, 43.7, 38.4, 36.0, 29.5 (2C), 29.3 (2C), 27.7, 25.6, 25.4; **HRMS** (ESI-TOF) mass calculated for $[M]^+$ $(C_{12}H_{25}N_2O)^+$ expected *m/z* 213.1961; found *m/z* 213.1960.

8-(azetidin-2-yl)octan-1-ol trifluoroacetic acid salt (**7bc**)

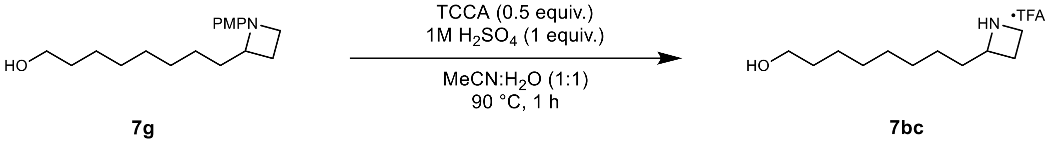

The *p*-methoxyphenyl (PMP) group was removed according to a modified version of a literature procedure.[14]

To a solution of protected azetidine **7g** (1.0 equiv., 0.1 mmol, 29.1 mg) in MeCN/H_2O (1:1, 0.05 M, 2 mL) in a 5 mL microwave vial were added trichloroisocyanuric acid (0.5 equiv., 0.05 mmol, 11.6 mg) and 1M aqueous H_2SO_4 (1 equiv., 0.1 mmol, 100 µL). The vial was then sealed, and the mixture was stirred at 90℃ for 1 h. The reaction mixture was diluted with 3 mL H_2O and washed with DCM (3×20 mL). The resultant aqueous layer was basified to pH 10.5 with 5M KOH and extracted with DCM (7×20 mL). The aqueous layer was then saturated with NaCl and further extracted with DCM (3×20 mL). The combined organic layers were dried over anhydrous Na_2SO_4, filtered, and subsequently acidified with trifluoroacetic acid (0.8 equiv., 0.08 mmol, 6.1 µL) and concentrated to afford the crude TFA salt. The crude residue was purified by flash column chromatography (SiO_2; gradient 100 $CHCl_3$ to 60:40 $CHCl_3$:MeOH) to afford compound **7bc** (15.8 mg; 54%) as a pale brown oil. **R$_f$** (85:15 $CHCl_3$:MeOH) 0.2; **IR** (film) ν_{max}/cm^{-1}: 3367, 2929, 2857, 1674, 1428, 1201, 1133, 1055, 835, 799, 721, 518; **^{1}H NMR** (CD_3OD, 500 MHz) δ (ppm): 4.53 – 4.35 (m, 1H), 4.09 – 4.01 (m, 1H), 3.89 – 3.81 (m, 1H), 3.54 (app t, *J* = 6.6 Hz, 2H), 2.59 – 2.50 (m, 1H), 2.37 – 2.27 (m, 1H), 1.96 – 1.80 (m, 2H), 1.55 – 1.50 (m, 2H), 1.37 – 1.31 (m, 10H); **^{13}C NMR** (CD_3OD, 126 MHz) δ (ppm): 62.9, 62.8, 43.9, 34.9, 33.6, 30.5, 30.4, 30.0, 26.9, 26.1, 25.6; **^{19}F NMR** (CD_3OD, 376 MHz) δ (ppm): -76.94; **HRMS** (ESI-TOF) mass calculated for $[M]^+$ $(C_{11}H_{24}NO)^+$ expected *m/z* 186.1852; found *m/z* 186.1852

2-phenethyloxetane (**8a**)

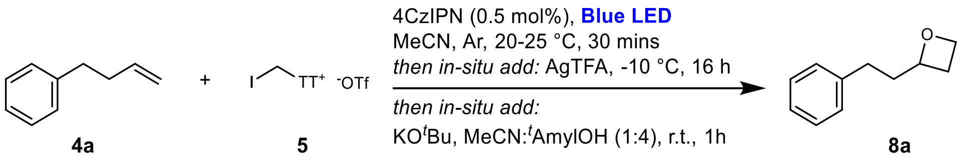

Prepared following **general procedure C**, using 4-Phenyl-1-butene **4a** (1.0 equiv.; 0.2 mmol; 30.0 µL), 5-iodomethyl thianthrenium triflate **5** (1.1 equiv.; 0.22 mmol; 111.4 mg), 4CzIPN (0.005 equiv.; 0.001 mmol; 0.8 mg), AgTFA (2.0 equiv.; 0.4 mmol; 88.4 mg), and KO^{t}Bu (4.0 equiv.; 0.8 mmol; 89.8 mg). The crude residue was purified by flash column chromatography (SiO_2; gradient 95:5 pentane:diethyl ether to 80:20 pentane:diethyl ether) to afford compound **8a** (20.2 mg; 62%) as a yellow oil. **R$_f$** (80:20 pentane:diethyl ether) 0.5; **IR** (film) νmax/cm^{-1}: 3083, 3061, 3027, 2980, 2928,

2879, 2369, 2334, 2021, 1727, 1603, 1496, 1454, 1380, 1263, 1227, 1176, 1111, 1030, 977,954, 863, 747, 699, 576, 508, 454, 417; **^{1}H NMR** ($CDCl_3$, 500 MHz) δ (ppm): 7.32 – 7.24 (m, 2H), 7.22 – 7.16 (m, 3H), 4.89 – 4.80 (m, 1H), 4.72 – 4.74 (m, 1H), 4.57 – 4.49 (m, 1H), 2.76 – 2.55 (m, 3H), 2.39 – 2.28 (m, 1H), 2.15 (dddd, 1H), 2.02 – 1.91 (m, 1H); **^{13}C NMR** ($CDCl_3$, 126 MHz) δ (ppm): 141.8, 128.6, 128.5, 126.0, 82.1, 68.3, 39.8, 30.6, 27.7; **HRMS** (ESI-TOF) mass calculated for $[M+Na]^+$ $(C_{11}H_{14}NaO)^+$ expected m/z 185.0937 found m/z 185.0934.

tert-butyl 9-(oxetan-2-yl)nonanoate (8b)

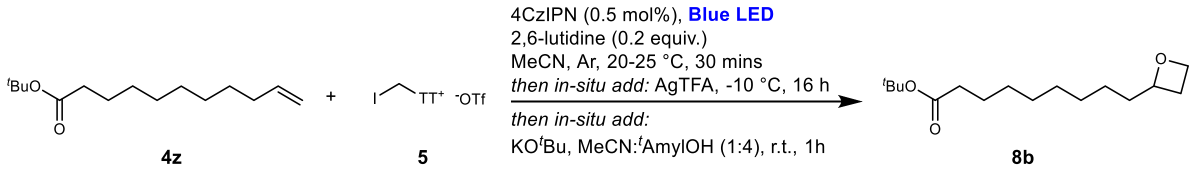

Prepared following **modified general procedure C**; An oven-dried Schlenk tube with atmosphere exchanged to Ar was charged with 5-iodomethyl thianthrenium triflate **5** (1.1 equiv.; 0.22 mmol; 111.4 mg) and 4CzIPN (0.005 equiv.; 0.001 mmol; 0.8 mg), followed by degassed dry MeCN (0.4 mL, 0.5 M, previously degassed through 10 min argon sparging). Then, tert-butyl undec-10-enoate **4z** (1.0 equiv.; 0.2 mmol; 48.1 mg), and 2,6-lutidine (0.2 equiv.; 0.04 mmol, 4.7 µL) were sequentially added to the mixture, the vessel sealed and placed in a glass-wall water bath where a fan was circulating air to maintain a water temperature of 20 - 25 °C. See Fig. S2 for visual details of the reaction setup. The reaction was irradiated through the glass wall with blue light (Kessil lamp A160WE Tuna Blue Saltwater LED Light 40 W) for 30 minutes under moderate stirring (500 rpm). The vessel was then removed from the water bath and cooled to -10 °C, then AgTFA (2.0 equiv.; 0.4 mmol; 88.4 mg) was introduced into the vessel. The mixture was stirred (500 rpm) at -10 °C without irradiation for 16 h using a cryostat. Subsequently, ^{t}AmylOH (1.6 mL) was added via syringe, and the Schlenk tube was taken out of the cryostat. Upon warming to room temperature, KO^{t}Bu (4.0 equiv.; 0.8 mmol; 89.8 mg) was introduced into the vessel. The reaction mixture was stirred (500 rpm) at room temperature in the absence of light for an additional 1 h. The reaction mixture was filtered through silica gel using ethyl acetate (30 mL) as eluent. The filtrate was concentrated under reduced pressure, and the resulting crude residue was purified by flash column chromatography (SiO_2; gradient 95:5 pentane:EtOAc to 80:20 pentane:EtOAc) to afford compound **8b** (33.4 mg; 62%) as a yellow oil. **R_f** (80:20 pentane:EtOAc) 0.5; **IR** (film) νmax/cm^{-1}: 2979, 2928, 2877, 2855, 1730, 1454, 1392, 1367, 1255, 1225, 1152, 1116, 1092, 981, 979, 921, 849, 755, 725; **^{1}H NMR** ($CDCl_3$, 500 MHz) δ (ppm): 4.84 – 4.76 (m, 1H), 4.68 – 4.62 (m, 1H), 4.52 – 4.45 (m, 1H), 2.68 – 2.59 (m, 1H), 2.35 – 2.28 (m, 1H), 2.21 – 2.17 (m, 2H), 1.83 – 1.75 (m, 1H), 1.67 – 1.61 (m, 1H), 1.60 – 1.55 (m, 2H), 1.44 (s, 9H), 1.32 – 1.25 (m, 10H).; **^{13}C NMR** ($CDCl_3$, 126 MHz) δ (ppm): 173.5, 83.0,

80.0, 68.2, 38.2, 35.8, 29.6 (2C overlapping), 29.3, 29.2, 28.3, 27.8, 25.2, 24.2; **HRMS** (ESI-TOF) mass calculated for $[M+Na]^+$ $(C_{16}H_{30}NaO_3)^+$ expected m/z 293.2087; found m/z 293.2084.

10-(oxetan-2-yl)decanenitrile (8c)

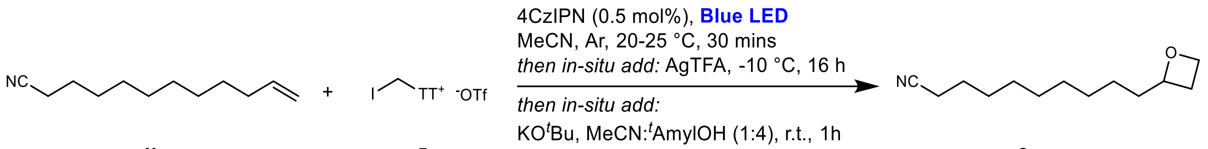

Prepared following **general procedure C**, using dodec-11-enenitrile **4b** (1.0 equiv.; 0.2 mmol; 43.0 µl), 5-iodomethyl thianthrenium triflate **5** (1.1 equiv.; 0.22 mmol; 111.4 mg), 4CzIPN (0.005 equiv.; 0.001 mmol; 0.8 mg), AgTFA (2.0 equiv.; 0.4 mmol; 88.4 mg), and KO^{t}Bu (4.0 equiv.; 0.8 mmol; 89.8 mg). The crude residue was purified by flash column chromatography (SiO_2; gradient 80:20 pentane:diethyl ether to 60:40 pentane:diethyl ether) to afford compound **8c** (25.4 mg; 61%) as a yellow oil. **R$_f$** (60:40 pentane:diethyl ether) 0.43; **IR** (film) vmax/cm^{-1}: 2927, 2855, 2245, 1466, 1427, 1377, 1226, 1092, 973, 919, 861, 721, 440; **^{1}H NMR** ($CDCl_3$, 500 MHz) δ (ppm): 4.85 – 4.76 (m, 1H), 4.70 – 4.62 (m, 1H), 4.52 – 4.45 (m, 1H), 2.70 – 2.58 (m, 1H), 2.37 – 2.29 (m, 3H), 1.84 – 1.73 (m, 1H), 1.70 – 1.59 (m, 3H), 1.48 – 1.39 (m, 2H), 1.37 – 1.22 (m, 10H); **^{13}C NMR** ($CDCl_3$, 126 MHz) δ (ppm): 120.0, 82.9, 68.2, 38.2, 29.6, 29.5, 29.3, 28.9, 28.8, 27.8, 25.5, 24.2, 17.3; **HRMS** (ESI-TOF) mass calculated for $[M+Na]^+$ $(C_{13}H_{23}NNaO)^+$ expected m/z 232.1672; found m/z 232.1672.

2-(4-(phenylsulfonyl)butyl)oxetane (8d)

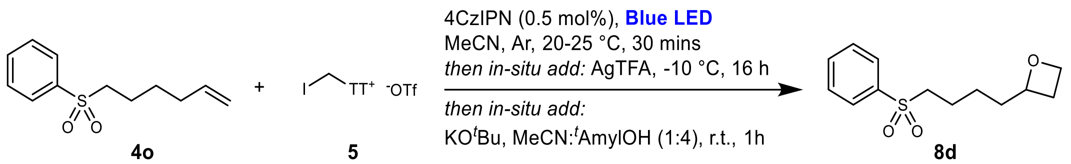

Prepared following **general procedure C**, using (hex-5-en-1-ylsulfonyl)benzene **4o** (1.0 equiv.; 0.2 mmol; 44.8 mg), 5-iodomethyl thianthrenium triflate **5** (1.1 equiv.; 0.22 mmol; 111.4 mg), 4CzIPN (0.005 equiv.; 0.001 mmol; 0.8 mg), AgTFA (2.0 equiv.; 0.4 mmol; 88.4 mg), and KO^{t}Bu (4.0 equiv.; 0.8 mmol; 89.8 mg). The crude residue was purified by flash column chromatography (SiO_2; gradient 60:40 pentane:EtOAc to 40:60 pentane: EtOAc) to afford compound **8d** (33.2 mg; 65%) as a yellow oil. **R$_f$** (40:60 EtOAc) 0.47; **IR** (film) vmax/cm^{-1}: 3064, 2927, 2876, 1585, 1479, 1447, 1406, 1382, 1320, 1304, 1290, 1288, 1228, 1145, 1086, 1024, 970, 968, 959, 925, 855, 795, 750, 729, 690, 594, 566, 535, 430; **^{1}H NMR** ($CDCl_3$, 500 MHz) δ (ppm): 7.99 – 7.86 (m, 2H), 7.68 – 7.63 (m, 1H), 7.60 – 7.54 (m, 2H), 4.84 – 4.70 (m, 1H), 4.68 – 4.58 (m, 1H), 4.52 – 4.40 (m, 1H), 3.12 – 3.07 (m, 2H),

2.71 – 2.57 (m, 1H), 2.33 – 2.24 (m, 1H), 1.80 – 1.71 (m, 3H), 1.65 – 1.60 (m, 1H), 1.47 – 1.34 (m, 2H); **^{13}C NMR** ($CDCl_3$, 126 MHz) δ (ppm): 139.3, 133.8, 129.4, 128.2, 82.1, 68.2, 56.3, 37.4, 27.6, 23.1, 22.7; **HRMS** (ESI-TOF) mass calculated for $[M+Na]^+$ $(C_{13}H_{18}NaO_3S)^+$ expected m/z 277.0869; found m/z 277.0870.

2-(4-(benzyloxy)butyl)oxetane (**8e**)

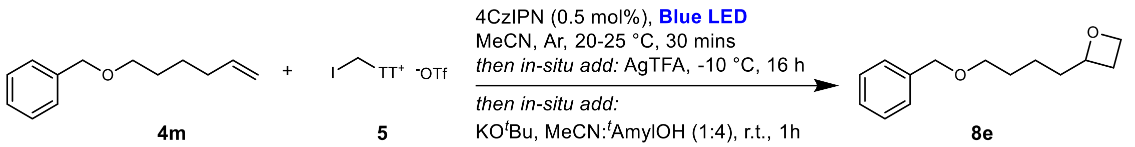

Prepared following **general procedure C**, using ((hex-5-en-1-yloxy)methyl)benzene **4m** (1.0 equiv.; 0.2 mmol; 38.0 mg), 5-iodomethyl thianthrenium triflate **5** (1.1 equiv.; 0.22 mmol; 111.4 mg), 4CzIPN (0.005 equiv.; 0.001 mmol; 0.8 mg), AgTFA (2.0 equiv.; 0.4 mmol; 88.4 mg), and KO^{t}Bu (4.0 equiv.; 0.8 mmol; 89.8 mg). The crude residue was purified by flash column chromatography (SiO_2; gradient 90:10 pentane:diethyl ether to 70:30 pentane:diethyl ether) to afford compound **8e** (23.6 mg; 53%) as a yellow oil. **R$_f$** (70:30 pentane:diethyl ether) 0.5; **IR** (film) vmax/cm^{-1}: 3063, 3031, 2990, 2936, 2877, 2794, 1496, 1453, 1362, 1309, 1227, 1203, 1117, 1101, 1028, 974, 865, 812, 735, 698, 612, 591, 471, 456, 452, 426, 424; **^{1}H NMR** ($CDCl_3$, 500 MHz) δ (ppm): 7.39 – 7.31 (m, 4H), 7.31 – 7.27 (m, 1H), 4.87 – 4.77 (m, 1H), 4.69 – 4.63 (m, 1H), 4.53 – 4.46 (m, 3H), 3.48 (t, J = 6.6 Hz, 2H), 2.69 – 2.60 (m, 1H), 2.38 – 2.27 (m, 1H), 1.86 – 1.78 (m, 1H), 170 – 1.61 (m, 3H), 1.47 – 1.34 (m, 2H); **^{13}C NMR** ($CDCl_3$, 126 MHz) δ (ppm): 138.8, 128.5, 127.8, 127.7, 82.8, 73.1, 70.4, 68.2, 37.9, 29.7, 27.8, 20.9; **HRMS** (ESI-TOF) mass calculated for $[M+Na]^+$ $(C_{14}H_{20}NaO_2)^+$ expected m/z 243.1356; found m/z 243.1358.

N-(4-(oxetan-2-yl)butyl)-4-(5-(p-tolyl)-3-(trifluoromethyl)-1H-pyrazol-1-yl) benzenesulfonamide (**8f**)

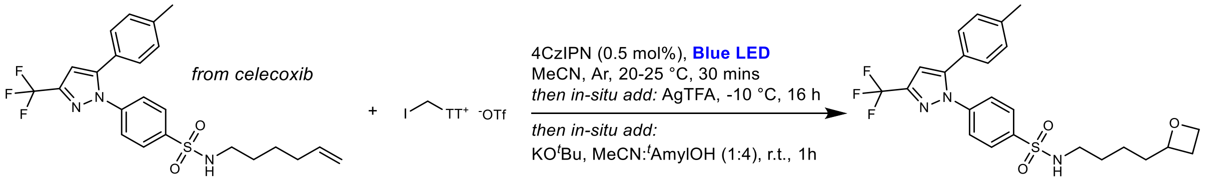

Prepared following **general procedure C**, using celecoxib-derived olefin **4w** (1.0 equiv.; 0.2 mmol; 92.7 mg), 5-iodomethyl thianthrenium triflate **5** (1.1 equiv.; 0.22 mmol; 111.4 mg), 4CzIPN (0.005 equiv.; 0.001 mmol; 0.8 mg), AgTFA (2.0 equiv.; 0.4 mmol; 88.4 mg), and KO^{t}Bu (4.0 equiv.; 0.8 mmol; 89.8 mg). The crude residue was purified by flash column chromatography (SiO_2; gradient 65:35 pentane:EtOAc to 55:45 pentane:EtOAc) to afford compound **8f** (54.6 mg; 55%) as a yellow oil. **R$_f$** (55:45 pentane: EtOAc) 0.5; **IR** (film) vmax/cm^{-1}: 3279, 2934, 2881, 1597, 1471, 1332, 1236,

1159, 1133, 1096, 975, 843, 808, 732, 626, 616, 572, 479; **^{1}H NMR** ($CDCl_3$, 500 MHz) δ (ppm): 7.85 – 7.82 (m, 2H), 7.48 – 7.45 (m, 2H), 7.19 – 7.15 (m, 2H), 7.12 – 7.08 (m, 2H), 6.74 (s, 1H), 4.77 (qd, *J* = 7.3, 5.2 Hz, 1H), 4.74 – 4.68 (m, 1H), 4.64 (td, *J* = 8.1, 5.9 Hz, 1H), 4.47 (dt, *J* = 9.2, 5.8 Hz, 1H), 2.96 (q, *J* = 6.7 Hz, 2H), 2.68 – 2.59 (m, 1H), 2.38 (s, 3H), 2.33 – 2.25 (m, 1H), 1.81 – 1.72 (m, 1H), 1.63 – 1.55 (m, 1H), 1.53 – 1.47 (m, 2H), 1.39 – 1.26 (m, 2H); **^{13}C NMR** ($CDCl_3$, 126 MHz) δ (ppm):145.4, 144.22 (q, J = 38.6 Hz), 142.6, 140.0, 139.7, 129.9, 128.9, 128.2, 125.8, 125.7, 121.20 (q, J = 269.2 Hz), 106.4, 82.6, 68.3, 43.3, 37.2, 29.4, 27.6, 21.5 (2C); **^{19}F NMR** ($CDCl_3$, 376 MHz) δ (ppm): -62.5 (s, 3F); **HRMS** (ESI-TOF) mass calculated for $[M+H]^+$ $(C_{24}H_{27}F_3N_3O_3S)^+$ expected m/z 494.1720; found m/z 494.1717.

2-phenethylthietane (9a)

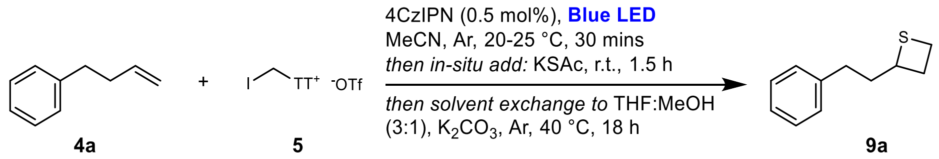

Prepared following **general procedure D**, using 4-Phenyl-1-butene **4a** (1.0 equiv.; 0.2 mmol; 30.0 µL), 5-iodomethyl thianthrenium triflate **5** (1.1 equiv.; 0.22 mmol; 111.4 mg), 4CzIPN (0.005 equiv.; 0.001 mmol; 0.8 mg), KSAc (1.2 equiv.; 0.24 mmol; 27.4 mg), and K_2CO_3 (2.0 equiv.; 0.4 mmol; 55.3 mg). The crude residue was purified by flash column chromatography (SiO_2; gradient 100 pentane to 95:5 pentane:DCM) to afford compound **9a** (23.7 mg; 66%) as a pale yellow oil. **R$_f$** (90:10 pentane:DCM) 0.5; **IR** (film) ν_{max}/cm^{-1}: 3061, 3025, 2928, 2855, 1603, 1495, 1453, 1266, 1103, 1030, 747, 608; **^{1}H NMR** ($CDCl_3$, 500 MHz) δ (ppm): 7.32 – 7.26 (m, 2H), 7.23 – 7.13 (m, 3H), 3.79 – 3.64 (m, 1H), 3.27 – 3.20 (m, 1H), 3.02 – 2.96 (m, 1H), 2.94 – 2.85 (m, 1H), 2.66 – 2.58 (m, 2H), 2.54 – 2.47 (m, 1H), 2.13 – 2.02 (m, 2H); **^{13}C NMR** ($CDCl_3$, 126 MHz) δ (ppm): 141.5, 128.6, 128.5, 126.0, 42.1, 41.2, 33.7, 32.9, 21.9; **HRMS** (EI-TOF) mass calculated for $M^{+\bullet}$ $(C_{11}H_{14}S)^{+\bullet}$ expected m/z 178.0811; found m/z 178.0804.

tert-butyl 9-(thietan-2-yl)nonanoate (9b)

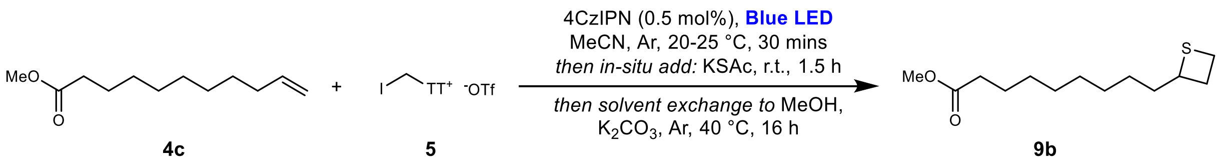

Prepared following **modified general procedure D**; to an oven-dried Schlenk tube under an Ar atmosphere, 5-iodomethyl thianthrenium triflate **5** (1.1 equiv.; 0.22 mmol; 111.4 mg), and 4CzIPN (0.005 equiv.; 0.001 mmol; 0.8 mg) were introduced followed by degassed dry MeCN (0.4 mL, 0.5 M, previously degassed through 10 min argon sparging) through a syringe. Then methyl undec-10-

enoate **4c** (1.0 equiv.; 0.2 mmol; 45 µL) was added and the vessel was sealed with a glass stopper using silicon grease. The Schlenk was then placed in a glass-wall water bath where a fan was blowing air to keep the water temperature between 20 - 25 °C. See Fig. S2 for visual details of the reaction setup. The reaction was irradiated through the glass wall with blue light (Kessil lamp A160WE Tuna Blue Saltwater LED Light 40 W) for 30 mins under moderate stirring (500 rpm). The vessel was then removed from the water bath and KSAc (1.2 equiv.; 0.24 mmol; 27.4 mg) was introduced into the vessel under an Ar atmosphere. The mixture was stirred (500 rpm) at 20 - 25 °C without irradiation for 1.5 h. The vessel was then removed from the stirring and the solvent evaporated under vacuum using a standard Schlenk manifold. After removal of all MeCN the atmosphere in the vessel was exchanged to argon and the crude was submitted to the next step. Under an Ar atmosphere, K_2CO_3 (2.0 equiv.; 0.4 mmol; 55.3 mg) was added, followed by degassed MeOH (4 mL, 0.05 M, previously degassed through 10 min argon sparging) through a syringe. The reaction was then allowed to stir (500 rpm) for 16 hours at 40 °C (oil bath).The mixture was diluted with 120mL of EtOAc, washed with distilled water (25 mL) and brine (25 mL). The organic layer was dried over anhydrous Na_2SO_4 and concentrated under reduced pressure. The crude was subjected to chromatographic purification on silica gel to afford final compounds. The crude residue was purified by flash column chromatography (SiO_2; gradient 100 pentane to 95:5 pentane:Et_2O) to afford compound **9b** (27.1 mg; 55%) as a pale-yellow oil. **R$_f$** (95:5 pentane:Et_2O) 0.5; **IR** (film) ν_{max}/cm^{-1}: 2924, 2653, 1730, 1464, 1457, 1435, 1361, 1259, 1242, 1196, 1169, 724; **^{1}H NMR** ($CDCl_3$, 500 MHz) δ (ppm): 3.76 – 3.67 (m, 1H), 3.66 (s, 3H), 3.26 – 3.17 (m, 1H), 2.97 – 2.84 (m, 2H), 2.63 – 2.55 (m, 1H), 2.30 – 2.26 (m, 2H), 1.77 – 1.65 (m, 2H), 1.62 – 1.56 (m, 2H), 1.29 – 1.14 (m, 10H); **^{13}C NMR** ($CDCl_3$, 126 MHz) δ (ppm): 174.4, 51.6, 43.0, 39.6, 34.2, 34.0, 29.4, 29.3 (2C overlapping), 29.2, 26.6, 25.0, 21.9; **HRMS** (ESI-TOF) mass calculated for $[M+Na]^+$ $(C_{13}H_{24}NaO_2S)^+$ expected *m/z* 267.1389; found *m/z* 267.1388.

10-(thietan-2-yl)decanenitrile (**9c**)

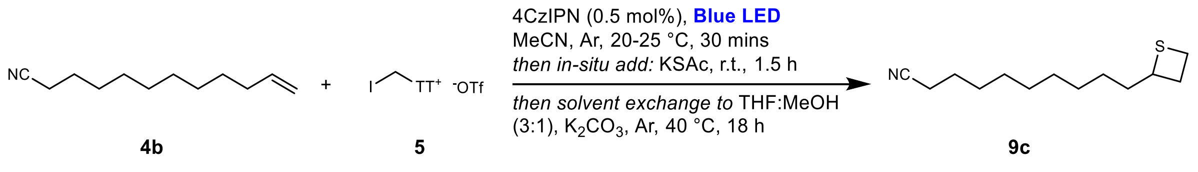

Prepared following **general procedure D**, using dodec-11-enenitrile **4b** (1.0 equiv.; 0.2 mmol; 43.0 µl), 5-iodomethyl thianthrenium triflate **5** (1.1 equiv.; 0.22 mmol; 111.4 mg), 4CzIPN (0.005 equiv.; 0.001 mmol; 0.8 mg), KSAc (1.2 equiv.; 0.24 mmol; 27.4 mg), and K_2CO_3 (2.0 equiv.; 0.4 mmol; 55.3 mg). The crude residue was purified by flash column chromatography (SiO_2; gradient 100 pentane to 90:10 pentane:Et_2O) to afford compound **9c** (28.9 mg; 64%) as a pale-yellow oil. **R$_f$** (90:10

pentane:Et_2O) 0.5; **IR** (film) ν_{max}/cm^{-1}:2926, 2853, 2245, 1464, 1425, 1263, 722; **^{1}H NMR** ($CDCl_3$, 500 MHz) δ (ppm): 3.77 – 3.67 (m, 1H), 3.27 – 3.17 (m, 1H), 3.00 – 2.84 (m, 2H), 2.65 – 2.52 (m, 1H), 2.37 – 2.28 (m, 2H), 1.80 – 1.60 (m, 4H), 1.48 – 1.38 (m, 2H), 1.36 – 1.12 (m, 10H).**^{13}C NMR** ($CDCl_3$, 126 MHz) δ (ppm): 120.0, 43.0, 39.6, 34.0, 29.5, 29.3, 29.3, 28.8, 28.8, 26.6, 25.5, 22.0, 17.3; **HRMS** (ESI-TOF) mass calculated for $[M+Na]^+$ $(C_{13}H_{23}NNaS)^+$ expected *m/z* 248.1443; found *m/z* 248.1444.

2-(4-(phenylsulfonyl)butyl)thietane (**9d**)

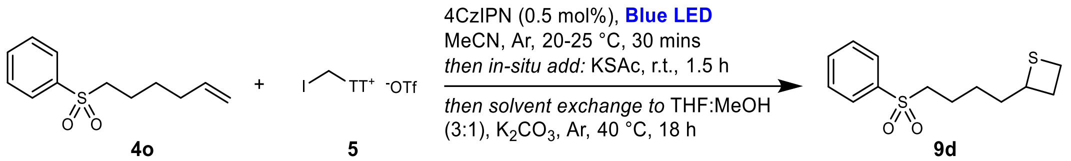

Prepared following **general procedure D**, using (hex-5-en-1-ylsulfonyl)benzene **4o** (1.0 equiv.; 0.2 mmol; 44.8 mg), 5-iodomethyl thianthrenium triflate **5** (1.1 equiv.; 0.22 mmol; 111.4 mg), 4CzIPN (0.005 equiv.; 0.001 mmol; 0.8 mg), KSAc (1.2 equiv.; 0.24 mmol; 27.4 mg), and K_2CO_3 (2.0 equiv.; 0.4 mmol; 55.3 mg). The crude residue was purified by flash column chromatography (SiO_2; gradient 100 pentane to 85:15 pentane:EtOAc *then* 100 toluene to 95:5 toluene:EtOAc) to afford compound **9d** (34.3 mg; 63%) as a transparent oil. **R_f** (90:10 toluene:EtOAc) 0.5; **IR** (film) ν_{max}/cm^{-1}: 3062, 2940, 2856, 1585, 1446, 1320, 1147, 1067, 690, 534; **^{1}H NMR** ($CDCl_3$, 500 MHz) δ (ppm): 7.94 – 7.87 (m, 2H), 7.71 – 7.61 (m, 1H), 7.61 – 7.53 (m, 2H), 3.71 – 3.58 (m, 1H), 3.26 – 3.14 (m, 1H), 3.13 – 3.01 (m, 2H), 2.99 – 2.83 (m, 2H), 2.62 – 2.48 (m, 1H), 1.81 – 1.62 (m, 4H), 1.37 – 1.21 (m, 2H); **^{13}C NMR** ($CDCl_3$, 126 MHz) δ (ppm): 139.3, 133.8, 129.4, 128.2, 56.3, 42.1, 38.8, 33.8, 25.4, 22.5, 21.9; **HRMS** (ESI-TOF) mass calculated for $[M+Na]^+$ $(C_{13}H_{18}NaO_2S_2)^+$ expected *m/z* 293.0640; found *m/z* 293.0640.

2-(4-(benzyloxy)butyl)thietane (**9e**)

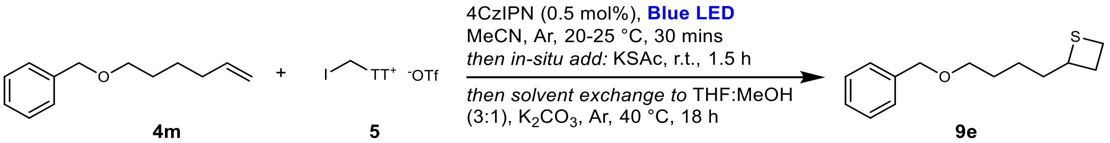

Prepared following **general procedure D**, using ((hex-5-en-1-yloxy)methyl)benzene **4m** (1.0 equiv.; 0.2 mmol; 38.0 mg), 5-iodomethyl thianthrenium triflate **5** (1.1 equiv.; 0.22 mmol; 111.4 mg), 4CzIPN (0.005 equiv.; 0.001 mmol; 0.8 mg), KSAc (1.2 equiv.; 0.24 mmol; 27.4 mg), and K_2CO_3 (2.0 equiv.; 0.4 mmol; 55.3 mg). The crude residue was purified by flash column chromatography (SiO_2; gradient 95:5 pentane:diethyl ether to 85:15 pentane:diethyl ether) to afford compound **9e**

(31.1 mg; 65%) as a yellow oil. **R$_f$** (85:15 pentane:diethyl ether) 0.5; **IR** (film) ν_{max}/cm^{-1}: 3063, 2931, 2855, 1729, 1495, 1453, 1363, 1309, 1160, 1100, 1028, 734, 698; **^{1}H NMR** ($CDCl_3$, 500 MHz) δ (ppm): 7.41 – 7.26 (m, 5H), 4.52 (s, 2H), 3.81 – 3.69 (m, 1H), 3.54 – 3.43 (m, 2H), 3.31 – 3.18 (m, 1H), 3.07 – 2.85 (m, 2H), 2.73 – 2.51 (m, 1H), 1.87 – 1.69 (m, 2H), 1.72 – 1.56 (m, 2H), 1.43 – 1.23 (m, 2H); **^{13}C NMR** ($CDCl_3$, 126 MHz) δ (ppm): 138.7, 128.5, 127.8, 127.6, 73.1, 70.3, 42.8, 39.4, 34.0, 29.5, 23.4, 22.0; **HRMS** (ESI-TOF) mass calculated for $[M+Na]^+$ $(C_{14}H_{20}NaOS)^+$ expected *m/z* 259.1127; found *m/z* 259.1129.

diethyl 2-phenethylcyclobutane-1,1-dicarboxylate (**10a**)

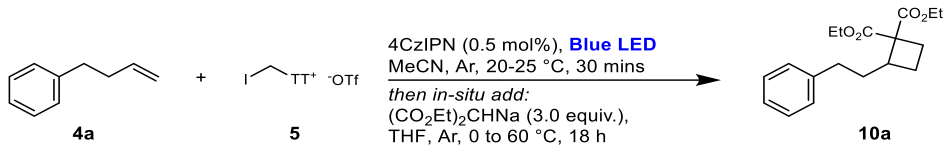

Prepared following **general procedure E**, using 4-Phenyl-1-butene **4a** (1.0 equiv.; 0.2 mmol; 30.0 µL), 5-iodomethyl thianthrenium triflate **5** (1.1 equiv.; 0.22 mmol; 111.4 mg), 4CzIPN (0.005 equiv.; 0.001 mmol; 0.8 mg), and $(CO_2Et)_2CHNa$ in THF (3.0 equiv.; 0.6 mmol; 1.20 mL). The crude residue was purified by flash column chromatography (SiO_2; gradient 98:2 pentane:EtOAc to 90:10 pentane:EtOAc) to afford compound **10a** (36.4 mg; 60%) as a transparent oil. **R$_f$** (90:10 pentane:EtOAc) 0.5; **IR** (film) ν_{max}/cm^{-1}: 3027, 2980, 2940, 2857, 1723, 1603, 1496, 1453, 1391, 1367, 1257, 1195, 1178, 1093, 1017, 859, 749, 700; **^{1}H NMR** ($CDCl_3$, 500 MHz) δ (ppm): 7.30 – 7.23 (m, 2H), 7.22 – 7.13 (m, 3H), 4.30 – 4.10 (m, 4H), 3.05 (qd, J = 9.1, 6.0 Hz, 1H), 2.67 – 2.51 (m, 3H), 2.19 – 2.09 (m, 1H), 2.06 – 1.96 (m, 1H), 1.90 – 1.72 (m, 2H), 1.63 – 1.52 (m, 1H), 1.29 – 1.21 (m, 6H); **^{13}C NMR** ($CDCl_3$, 126 MHz) δ (ppm): 172.1, 170.5, 142.2, 128.5, 128.4, 125.9, 61.3, 61.2, 56.9, 40.5, 33.9, 33.1, 26.1, 23.0, 14.4, 14.2; **HRMS** (ESI-TOF) mass calculated for $[M+Na]^+$ $(C_{18}H_{24}NaO_4)^+$ expected *m/z* 327.1567; found *m/z* 327.1564.

diethyl 2-(9-(tert-butoxy)-9-oxononyl)cyclobutane-1,1-dicarboxylate (**10b**)

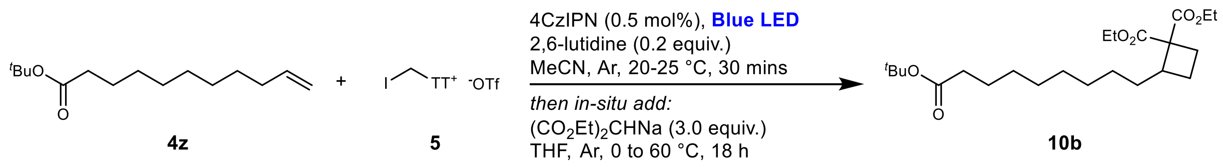

Prepared following **modified general procedure E**; An oven-dried Schlenk tube with atmosphere exchanged to Ar was charged with 5-iodomethyl thianthrenium triflate **5** (1.1 equiv.; 0.22 mmol; 111.4 mg) and 4CzIPN (0.005 equiv.; 0.001 mmol; 0.8 mg), followed by degassed dry MeCN (0.4 mL, 0.5 M, previously degassed through 10 min argon sparging). Then, tert-butyl undec-10-enoate **4z** (1.0 equiv.; 0.2 mmol; 48.1 mg), and 2,6-lutidine (0.2 equiv.; 0.04 mmol, 4.7 µL) were sequentially

added to the mixture, the vessel sealed and placed in a glass-wall water bath where a fan was circulating air to maintain a water temperature of 20 - 25 °C. See Fig. S2 for visual details of the reaction setup. The reaction was irradiated through the glass wall with blue light (Kessil lamp A160WE Tuna Blue Saltwater LED Light 40 W) for 30 minutes under moderate stirring (500 rpm). The vessel was then removed from the water bath and cooled to 0 °C (ice bath) before adding $(CO_2Et)_2CHNa$ in THF (0.5M; 3.0 equiv.; 0.6 mmol; 1.2 mL) (for preparation of the $(CO_2Et)_2CHNa$ stock solution see **general procedure E**) to the reaction mixture dropwise. The reaction was allowed to stir at 0 °C for 30 minutes before warming to room temperature and stirring for a further 2 hours. The reaction was then allowed to stir overnight for a further 16 hours at 60 °C (oil bath). Upon completion, the reaction was quenched with water (5 mL) and EtOAc (5 mL) was added. The organic layer was separated and the aqueous layer extracted with EtOAc (3 x 10 mL). The organic layer was dried over anhydrous Na_2SO_4 and concentrated under reduced pressure. The crude was subjected to chromatographic purification on silica gel to afford final compounds. The crude residue was purified by flash column chromatography (SiO_2; gradient 97:3 pentane:EtOAc to 95:5 pentane:EtOAc) to afford compound **10b** (47.8 mg; 58%) as a colourless oil. **R_f** (95:5 pentane:EtOAc) 0.3; **IR** (film) ν_{max}/cm^{-1}: 2979, 2928, 2855, 1727, 1464, 1367, 1259, 1194, 1150, 1095, 1041; **^1H NMR** ($CDCl_3$, 500 MHz) δ (ppm): 4.27 – 4.09 (m, 4H), 2.97 (qd, J = 8.7 5.7 Hz, 1H), 2.64 – 2.56 (m, 1H), 2.18 (t, J = 7.5 Hz, 2H), 2.09 (dt, J = 11.7, 9.1 Hz, 1H), 2.02 – 1.92 (m, 1H), 1.76 – 1.65 (m, 1H), 1.59 – 1.51 (m, 2H), 1.51 – 1.45 (m, 1H), 1.43 (s, 9H), 1.32 – 1.16 (m, 17H); **^{13}C NMR** ($CDCl_3$, 126 MHz) δ (ppm): 173.5, 172.2, 170.6, 80.0, 61.1, 61.1, 56.9, 40.8, 35.8, 32.1, 29.7, 29.6, 29.4, 29.2, 28.3, 26.7, 26.1, 25.2, 23.0, 14.4, 14.2; **HRMS** (ESI-TOF) mass calculated for $[M+Na]^+$ $(C_{23}H_{40}NaO_6)^+$ expected *m/z* 435.2717; found *m/z* 435.2716.

diethyl 2-(9-cyanononyl)cyclobutane-1,1-dicarboxylate (**10c**)

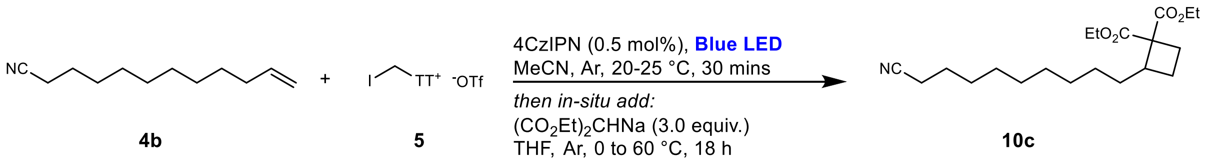

Prepared following **general procedure E**, using dodec-11-enenitrile **4b** (1.0 equiv.; 0.2 mmol; 43.0 µL), 5-iodomethyl thianthrenium triflate **5** (1.1 equiv.; 0.22 mmol; 111.4 mg), 4CzIPN (0.005 equiv.; 0.001 mmol; 0.8 mg), and $(CO_2Et)_2CHNa$ in THF (3.0 equiv.; 0.6 mmol; 1.20 mL). The crude residue was purified by flash column chromatography (SiO_2; gradient 90:10 pentane:EtOAc to 80:20 pentane:EtOAc) to afford compound **10c** (35.1 mg; 50%) as a transparent oil. **R_f** (85:15 pentane:EtOAc) 0.3; **IR** (film) ν_{max}/cm^{-1}: 2981, 2926, 2855, 1725, 1465, 1367, 1261, 1194, 1096; **^1H NMR** ($CDCl_3$, 500 MHz) δ (ppm): 4.28 – 4.08 (m, 4H), 2.97 (qd, J = 8.8, 5.7 Hz, 1H), 2.65 – 2.56 (m, 1H), 2.33 (t, J = 7.1 Hz, 2H), 2.13 – 2.04 (m, 1H), 2.02 – 1.92 (m, 1H), 1.78 – 1.60 (m, 3H), 1.52

– 1.38 (m, 3H), 1.36 – 1.16 (m, 17H); **^{13}C NMR** ($CDCl_3$, 126 MHz) δ (ppm): 172.2, 170.6, 120.0, 61.2, 61.1, 56.9, 40.8, 32.1, 29.6, 29.6, 29.4, 28.9, 28.8, 26.6, 26.1, 25.5, 23.0, 17.3, 14.4, 14.2; **HRMS** (ESI-TOF) mass calculated for $[M+Na]^+$ $(C_{20}H_{33}NNaO_4)^+$ expected *m/z* 374.2302; found *m/z* 374.2307.

diethyl 2-(4-(phenylsulfonyl)butyl)cyclobutane-1,1-dicarboxylate (**10d**)

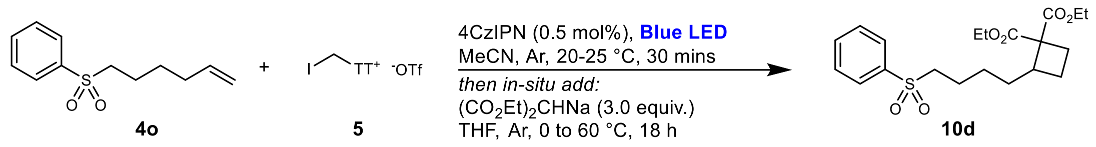

Prepared following **general procedure E**, using (hex-5-en-1-ylsulfonyl)benzene **4o** (1.0 equiv.; 0.2 mmol; 40.1 µL), 5-iodomethyl thianthrenium triflate **5** (1.1 equiv.; 0.22 mmol; 111.4 mg), 4CzIPN (0.005 equiv.; 0.001 mmol; 0.8 mg), and $(CO_2Et)_2CHNa$ in THF (3.0 equiv.; 0.6 mmol; 1.20 mL). The crude residue was purified by flash column chromatography (SiO_2; gradient 100% pentane to 80:20 pentane:EtOAc) to afford compound **10d** (50.8 mg; 64%) as a pale yellow oil. **R_f** (80:20 pentane:EtOAc) 0.3; **IR** (film) ν_{max}/cm^{-1}: 3066, 2981, 2942, 2870, 1729, 1585, 1447, 1391, 1321, 1304, 1146, 1088, 691, 595, 567, 535; **^{1}H NMR** ($CDCl_3$, 500 MHz) δ (ppm): 7.94 – 7.87 (m, 2H), 7.68 – 7.63 (m, 1H), 7.59 – 7.54 (m, 2H), 4.24 – 4.10 (m, 4H), 3.08 – 3.02 (m, 2H), 2.91 (qd, J = 9.0, 6.2 Hz, 1H), 2.63 – 2.50 (m, 1H), 2.12 – 2.01 (m, 1H), 1.96 – 1.89 (m, 1H), 1.73 – 1.64 (m, 3H), 1.51 – 1.41 (m, 1H), 1.36 – 1.21 (m, 9H); **^{13}C NMR** ($CDCl_3$, 126 MHz) δ (ppm): 171.9, 170.4, 139.3, 133.8, 129.4, 128.2, 61.3, 61.2, 56.9, 56.3, 40.4, 31.6, 26.1, 25.6, 22.9, 22.7, 14.4, 14.2; **HRMS** (ESI-TOF) mass calculated for $[M+Na]^+$ $(C_{20}H_{28}NaO_6S)^+$ expected *m/z* 419.1499; found *m/z* 419.1501.

diethyl 2-(4-(benzyloxy)butyl)cyclobutane-1,1-dicarboxylate (**10e**)

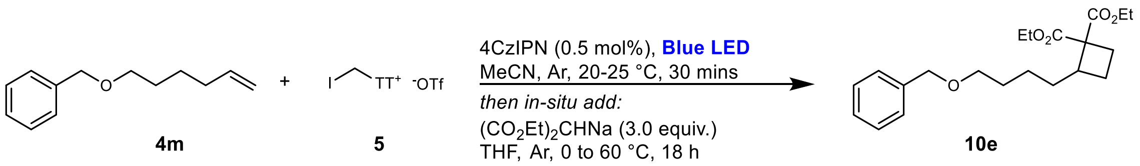

Prepared following **general procedure E**, using ((hex-5-en-1-yloxy)methyl)benzene **4m** (1.0 equiv.; 0.2 mmol; 42.0 µL), 5-iodomethyl thianthrenium triflate **5** (1.1 equiv.; 0.22 mmol; 111.4 mg), 4CzIPN (0.005 equiv.; 0.001 mmol; 0.8 mg), and $(CO_2Et)_2CHNa$ in THF (3.0 equiv.; 0.6 mmol; 1.20 mL). The crude residue was purified by flash column chromatography (SiO_2; gradient 97:3 pentane:EtOAc to 95:5 pentane:EtOAc) to afford compound **10e** (36.2 mg; 50%) as a transparent oil. **R_f** (95:5 pentane:EtOAc) 0.3; **IR** (film) ν_{max}/cm^{-1}: 3029, 2981, 2937, 2854, 1728, 1496, 1261, 1096, 735, 699; **^{1}H NMR** ($CDCl_3$, 500 MHz) δ (ppm): 7.30 – 7.24 (m, 4H), 7.24 – 7.17 (m, 1H), 4.42 (s, 2H), 4.20 – 4.03 (m, 4H), 3.38 (t, J = 6.6 Hz, 2H), 2.92 (qd, J = 9.0, 5.7 Hz, 1H), 2.59 – 2.49 (m, 1H),

2.09 – 1.98 (m, 1H), 1.96 – 1.87 (m, 1H), 1.72 – 1.60 (m, 1H), 1.57 – 1.49 (m, 2H), 1.51 – 1.42 (m, 1H), 1.31 – 1.15 (m, 9H); **^{13}C NMR** ($CDCl_3$, 126 MHz) δ (ppm): 172.1, 170.5, 138.8, 128.5, 127.7, 127.6, 73.0, 70.4, 61.2, 61.1, 56.9, 40.8, 32.0, 29.8, 26.1, 23.4, 23.0, 14.3, 14.2; **HRMS** (ESI-TOF) mass calculated for $[M+Na]^+$ $(C_{21}H_{30}NaO_5)^+$ expected *m/z* 385.1985; found *m/z* 385.1982.

2-phenethylcyclobutane-1,1-dicarbonitrile (**10f**)

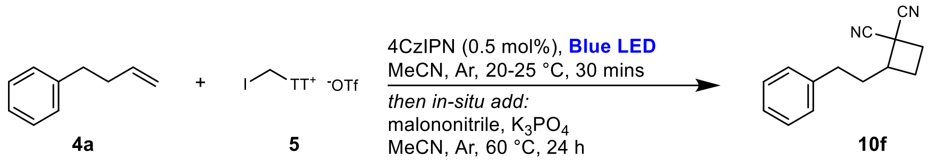

Prepared following **general Procedure F** using 4-Phenyl-1-butene **4a** (1.0 equiv.; 0.2 mmol; 30.0 µL), 5-iodomethyl thianthrenium triflate **5** (1.1 equiv.; 0.22 mmol; 111.4 mg), 4CzIPN (0.005 equiv.; 0.001 mmol; 0.8 mg), Malononitrile (3.0 equiv.; 0.6 mmol; 39.6 mg), and K_3PO_4 (6.0 equiv.; 1.2 mmol; 254.7 mg). The crude residue was purified by flash column chromatography (SiO_2; gradient 100 pentane to 90:10 pentane:EtOAc) to afford compound **10f** (27.3mg; 65%) as a colourless oil. **R_f** (90:10 pentane:EtOAc) 0.4; **IR** (film) ν_{max}/cm^{-1}: 3028, 2926, 2859, 2246, 1603, 1496, 1454, 749, 699, 507; **^{1}H NMR** ($CDCl_3$, 500 MHz) δ (ppm): 7.33 – 7.30 (m, 2H), 7.25 – 7.20 (m, 3H), 2.99 (dq, *J* = 10.1, 8.0 Hz, 1H), 2.75 – 2.60 (m, 4H), 2.26 – 2.20 (m, 1H), 2.17 – 1.96 (m, 3H); **^{13}C NMR** ($CDCl_3$, 126 MHz) δ (ppm): 140.1, 128.8, 128.5, 126.6, 115.9, 114.6, 44.9, 34.5, 32.1, 30.6, 30.4, 24.8. **HRMS** (ESI-TOF) mass calculated for $[M+Na]^+$ $(C_{14}H_{14}N_2Na)^+$ expected *m/z* 233.1049; found *m/z* 233.1050.

ethyl 1-cyano-2-phenethylcyclobutane-1-carboxylate (**10g**)

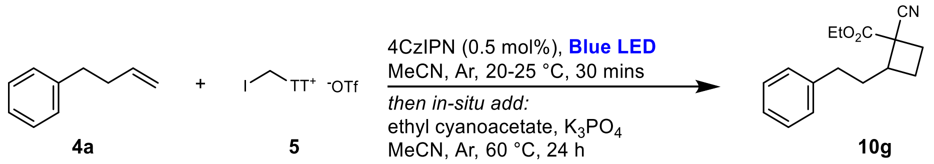

Prepared following **general Procedure F** using 4-Phenyl-1-butene **4a** (1.0 equiv.; 0.2 mmol; 30.0 µL), 5-iodomethyl thianthrenium triflate **5** (1.1 equiv.; 0.22 mmol; 111.4 mg), 4CzIPN (0.005 equiv.; 0.001 mmol; 0.8 mg), Ethyl cyanoacetate (3.0 equiv.; 0.6 mmol; 64.0 µL), and K_3PO_4 (6.0 equiv.; 1.2 mmol; 254.7 mg). The crude residue was purified by flash column chromatography (SiO_2; gradient 100 pentane to 90:10 pentane:EtOAc) to afford compound **10g** (24.7mg; 48% with 1:1.1 dr) as a colourless oil. **R_f** (90:10 pentane:EtOAc) 0.3; **IR** (film) ν_{max}/cm^{-1}: 2982, 2940, 1736, 1603, 1496, 1454, 1249, 1217, 749, 700; **^{1}H NMR** ($CDCl_3$, 500 MHz) δ (ppm): 7.31 – 7.26 (m, 2H), 7.23 – 7.12 (m, 3H), 4.35 – 4.22 (m, 2H), 3.10 – 3.00 (m, 1H, diastereoisomer 1), 2.91 – 2.83 (m, 1H, diastereoisomer 2), 2.68 – 2.51 (m, 3H), 2.47 – 2.37 (m, 1H), 2.19 – 1.90 (m, 3H), 1.89 – 1.81 (m, 1H, diastereoisomer 1), 1.71 – 1.61 (m, 1H, diastereoisomer 1), 1.37 – 1.29 (m, 3H); **^{13}C NMR**

(CDCl3, 126 MHz) δ (ppm): 168.6, 167.7, 141.1, 140.9, 128.7, 128.6, 128.5 (2C), 126.3, 126.2, 120.3, 118.5, 62.9, 62.7, 45.8, 45.3, 43.0, 42.6, 35.0, 33.5, 32.5, 32.4, 27.8, 27.2, 24.3, 23.8, 14.3, 14.1. **HRMS** (ESI-TOF) mass calculated for $[M+Na]^+$ $(C_{16}H_{19}NNaO_2)^+$ expected *m/z* 280.1308; found *m/z* 280.1308.

(2-phenethylcyclobutane-1,1-disulfonyl)dibenzene (**10h**)

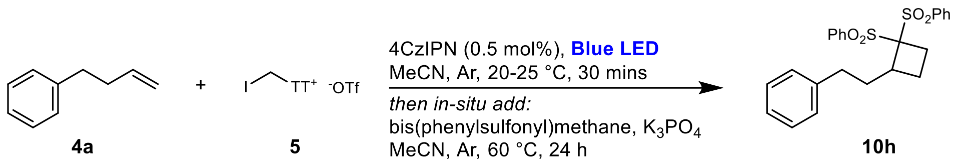

Prepared following **general Procedure F** using 4-Phenyl-1-butene **4a** (1.0 equiv.; 0.2 mmol; 30.0 µL), 5-iodomethyl thianthrenium triflate **5** (1.1 equiv.; 0.22 mmol; 111.4 mg), 4CzIPN (0.005 equiv.; 0.001 mmol; 0.8 mg), Bis(phenylsulfonyl)methane (3.0 equiv.; 0.6 mmol; 39.6 mg), and K_3PO_4 (6.0 equiv.; 1.2 mmol; 254.7 mg). The crude residue was purified by flash column chromatography (SiO_2; gradient 100 pentane to 90:10 pentane:EtOAc) to afford compound **10h** (45.8mg; 52%) as a light-yellow oil. **R_f** (80:20 pentane:EtOAc) 0.3; **IR** (film) ν_{max}/cm^{-1}: 3063, 2958, 1583, 1447, 1308, 1142, 1074, 912, 723, 686, 595, 573, 547; **^{1}H NMR** (CDCl3, 500 MHz) δ (ppm): 8.12 – 8.09 (m, 2H), 7.80 – 7.78 (m, 2H), 7.72 – 7.65 (m, 2H), 7.60 – 7.56 (m, 2H), 7.50 – 7.46 (m, 2H), 7.33 – 7.29 (m, 2H), 7.26 – 7.22 (m, 1H), 7.05 – 7.03 (m, 2H), 3.35 – 3.26 (m, 1H), 3.12 (dt, *J* = 13.7, 9.7 Hz, 1H), 2.68 – 2.55 (m, 2H), 2.50 – 2.26 (m, 3H), 2.18 (qd, *J* = 9.6, 2.9 Hz, 1H), 1.45 (dddd, *J* = 12.9, 9.0, 7.2, 3.5 Hz, 1H); **^{13}C NMR** (CDCl3, 126 MHz) δ (ppm): 140.8, 138.0, 136.3, 134.5, 134.4, 131.3, 130.8, 128.9, 128.8, 128.6, 128.6, 126.3, 87.5, 43.0, 32.6, 31.2, 24.6, 23.5. **HRMS** (ESI-TOF) mass calculated for $[M+Na]^+$ $(C_{24}H_{24}NaO_4S_2)^+$ expected *m/z* 463.1008; found *m/z* 463.1014.

2-phenethyl-1'-phenylspiro[cyclobutane-1,3'-indolin]-2'-one (**10i**)

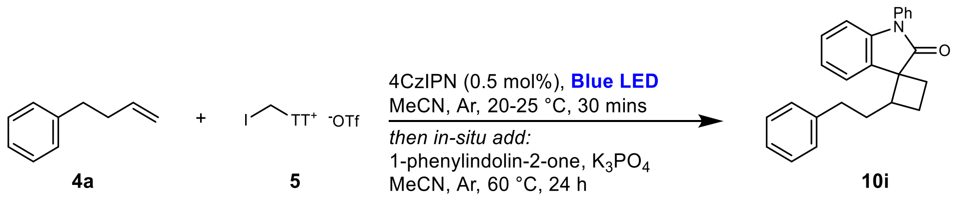

Prepared following **general Procedure F** using 4-Phenyl-1-butene **4a** (1.0 equiv.; 0.2 mmol; 30.0 µL), 5-iodomethyl thianthrenium triflate **5** (1.1 equiv.; 0.22 mmol; 111.4 mg), 4CzIPN (0.005 equiv.; 0.001 mmol; 0.8 mg), 1-phenylindolin-2-one (3.0 equiv.; 0.6 mmol; 125.5 mg), and K_3PO_4 (6.0 equiv.; 1.2 mmol; 254.7 mg). The crude residue was purified by flash column chromatography (SiO_2; gradient 100 pentane to 90:10 pentane:EtOAc) to afford compound **10i** (39.5 mg; 56% with 2:1 dr) as a colourless oil.

Major Diastereomer: **R$_f$** (90:10 pentane:EtOAc) 0.3; **IR** (film) ν_{max}/cm^{-1}: 3060, 3027, 2935, 2853, 1717, 1610, 1499, 1480, 1464, 1454, 1373, 1329, 1305, 1276, 1228, 1207, 1175, 1103, 1072, 1027, 974, 866, 752, 698, 643, 620, 569, 493, 426; **^{1}H NMR** ($CDCl_3$, 500 MHz) δ (ppm): 7.57 (dd, J = 7.4, 1.4 Hz, 1H), 7.54 – 7.48 (m, 2H), 7.43 – 7.36 (m, 3H), 7.26 – 7.22 (m, 1H), 7.20 – 7.13 (m, 3H), 7.13 – 7.09 (m, 1H), 6.89 – 6.83 (m, 3H), 3.18 – 3.08 (m, 1H), 2.82 – 2.72 (m, 1H), 2.37 – 2.26 (m, 2H), 2.25 – 2.17 (m, 1H), 2.13 – 2.04 (m, 2H), 1.79 – 1.69 (m, 1H), 1.69 – 1.59 (m, 1H); **^{13}C NMR** ($CDCl_3$, 126 MHz) δ (ppm): 178.9, 143.7, 142.0, 134.9, 130.9, 129.7 (2C), 128.4 (2C), 128.3 (2C), 128.0, 127.8, 126.7 (2C), 125.9, 124.3, 122.7, 109.4, 52.2, 44.2, 34.5, 33.0, 27.9, 24.7; **HRMS** (ESI-TOF) mass calculated for $[M+Na]^+$ $(C_{25}H_{23}NNaO)^+$ expected *m/z* 376.1672; found *m/z* 376.1675.

Minor Diastereomer: **R$_f$** (90:10 pentane:EtOAc) 0.28; **IR** (film) ν_{max}/cm^{-1}: 3061, 3026, 2979, 2939, 2856, 1714, 1612, 1596, 1499, 1481, 1464, 1454, 1373, 1327, 1301, 1266, 1198, 1177, 1161, 1101, 1074, 1028, 749, 699, 622, 573, 514, 494; **^{1}H NMR** ($CDCl_3$, 500 MHz) δ (ppm): 7.54 – 7.48 (m, 2H), 7.44 – 7.36 (m, 4H), 7.22 – 7.15 (m, 3H), 7.15 – 7.09 (m, 2H), 6.94 – 6.89 (m, 2H), 6.81 (d, J = 7.8 Hz, 1H), 2.93 – 2.83 (m, 1H), 2.53 – 2.46 (m, 1H), 2.43 – 2.35 (m, 2H), 2.35 – 2.24 (m, 2H), 2.17 – 2.09 (m, 1H), 2.05 – 1.89 (m, 2H); **^{13}C NMR** ($CDCl_3$, 126 MHz) δ (ppm): 178.6, 143.0, 141.8, 134.9, 133.3, 129.7 (2C), 128.5 (2C), 128.3 (2C), 127.9, 127.8, 126.6 (2C), 125.8, 123.1, 123.1, 109.0, 52.2, 46.0, 33.2, 33.1, 28.4, 23.3; **HRMS** (ESI-TOF) mass calculated for $[M+Na]^+$ $(C_{25}H_{23}NNaO)^+$ expected *m/z* 376.1672; found *m/z* 376.1675.

2-phenethylspiro[cyclobutane-1,4'-isochroman]-3'-one (**10j**)

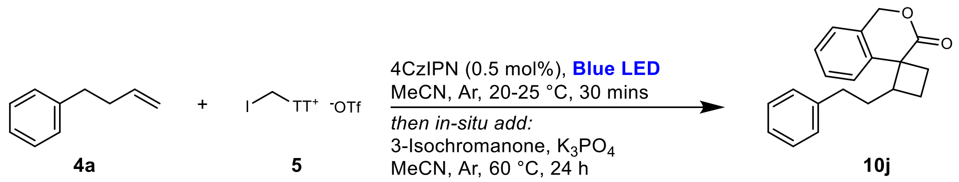

Prepared following **general Procedure F** using 4-Phenyl-1-butene **4a** (1.0 equiv.; 0.2 mmol; 30.0 µL), 5-iodomethyl thianthrenium triflate **5** (1.1 equiv.; 0.22 mmol; 111.4 mg), 4CzIPN (0.005 equiv.; 0.001 mmol; 0.8 mg), isochroman-3-one (3.0 equiv.; 0.6 mmol; 88.9 mg), and K_3PO_4 (6.0 equiv.; 1.2 mmol; 254.7 mg). The crude residue was purified by flash column chromatography (SiO_2; gradient 100 pentane to 90:10 pentane:EtOAc) to afford compound **10j** (33.9 mg; 58% with 2:1 dr) as a colourless oil.

Major Diastereomer: **R$_f$** (90:10 pentane:EtOAc) 0.25; **IR** (film) ν_{max}/cm^{-1}: 3026, 2937, 1736, 1493, 1455, 1241, 1091, 1028, 752, 700; **^{1}H NMR** ($CDCl_3$, 500 MHz) δ (ppm): 7.55 – 7.49 (m, 1H), 7.45 – 7.37 (m, 1H), 7.32 – 7.26 (m, 1H), 7.26 – 7.21 (m, 2H), 7.20 – 7.13 (m, 2H), 7.14 – 7.07 (m, 2H), 5.23 (d, J = 14.1 Hz, 1H), 5.12 (d, J = 14.0 Hz, 1H), 3.04 – 2.92 (m, 1H), 2.70 – 2.49 (m, 2H), 2.49

– 2.31 (m, 2H), 2.31 – 2.18 (m, 1H), 2.15 – 1.99 (m, 1H), 1.95 – 1.81 (m, 2H); **^{13}C NMR** ($CDCl_3$, 126 MHz) δ (ppm): 173.2, 141.4, 138.1, 131.3, 129.0, 128.54, 128.47, 127.1, 126.1, 125.0, 124.1, 69.2, 51.1, 48.6, 35.2, 32.9, 23.0, 21.4. **HRMS** (ESI-TOF) mass calculated for $[M+Na]^+$ $(C_{20}H_{20}NaO_2)^+$ expected *m/z* 315.1356; found *m/z* 315.1354.

Minor Diastereomer: **R$_f$** (90:10 pentane:EtOAc) 0.24; **IR** (film) ν_{max}/cm^{-1}: 3060, 2933, 1737, 1493, 1454, 1385, 1238, 1029, 750, 700; **^{1}H NMR** ($CDCl_3$, 500 MHz) δ (ppm): 7.47 – 7.37 (m, 2H), 7.34 – 7.27 (m, 1H), 7.24 – 7.09 (m, 4H), 7.00 – 6.91 (m, 2H), 5.19 – 5.12 (d, J = 13.9 Hz, 1H), 5.12 – 5.05 (d, J = 13.9 Hz, 1H), 3.03 – 2.91 (m, 1H), 2.91 – 2.80 (m, 1H), 2.72 – 2.60 (m, 1H), 2.54 – 2.43 (m, 1H), 2.43 – 2.33 (m, 1H), 2.32 – 2.19 (m, 1H), 1.80 – 1.68 (m, 1H), 1.34 – 1.15 (m, 2H); **^{13}C NMR** ($CDCl_3$, 126 MHz) δ (ppm): 174.9, 141.4, 134.5, 132.2, 128.8, 128.5, 128.4, 127.4, 126.1, 125.9, 124.9, 69.3, 50.6, 43.2, 34.1, 32.4, 22.4, 22.2. **HRMS** (ESI-TOF) mass calculated for $[M+Na]^+$ $(C_{20}H_{20}NaO_2)^+$ expected *m/z* 315.1356; found *m/z* 315.1356.

ethyl 2-phenethylcyclobutane-1-carboxylate (10k)

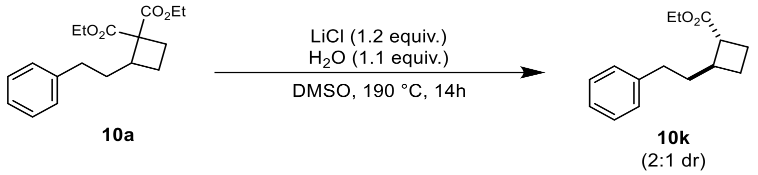

Cyclobutane **10k** was prepared according to a modified literature procedure.[15]

A microwave vial was charged with cyclobutane **10a** (1.0 equiv.; 0.1 mmol; 30.4 mg) and a magnetic stirrer bar. DMSO (200 µL; 0.5 M) was added followed by water (1.1 equiv.; 0.11 mmol; 2 µL), and LiCl (1.05 equiv.; 0.105 mmol; 9 mg). A cap was placed on the microwave vial and crimped closed to secure it. The reaction was allowed to stir at 190 °C for 14 hours. Upon completion, the reaction was cooled to room temperature and the microwave vial cap was removed. The reaction was diluted with water (2 mL) and EtOAc (2 mL). The organic layer was separated, and the aqueous layer was extracted with EtOAc (3 x 2 mL). The organic layer was dried over anhydrous Na_2SO_4 and concentrated under reduced pressure. The crude residue was purified by flash column chromatography (SiO_2; gradient 98:2 pentane:EtOAc to 90:10 pentane:EtOAc) to afford compound **10k** (16.3 mg; 70%) as a 2:1 ratio of diastereomers. The minor diastereomer was assigned as the cis-substituted cyclobutane upon NOESY analysis, observing an NOE interaction between the protons of the two C-H signals alpha to the ester and alkyl chain. The same interaction was not observed for the same protons in the major distereoisomer, confirming the stereochemical assignment. The compound obtained was a colourless oil. **R$_f$** (90:10 pentane:EtOAc) 0.6; **IR** (film) ν_{max}/cm^{-1}: 3027, 2982, 2934, 2855, 1727, 1603, 1496, 1454, 1373, 1345, 1262, 1244, 1179, 1042; **^{1}H NMR** ($CDCl_3$,

500 MHz) δ (ppm): 7.31 – 7.22 (m, 2H), 7.22 – 7.11 (m, 3H), 4.21 – 4.04 (m, 2H), 3.25 – 3.12 (m, 1H, minor diastereomer), 2.80 – 2.69 (m, 1H, major diastereomer), 2.69 – 2.44 (m, 3H), 3.36 – 1.95 (m, 3H), 1.93 – 1.50 (m, 3H), 1.36 – 1.20 (m, 3H); **^{13}C NMR** ($CDCl_3$, 126 MHz) δ (ppm): 175.1, 174.5, 142.4, 142.3, 128.54, 128.46, 128.4 (2C overlapping), 125.9, 125.8, 60.3, 60.2, 44.6, 41.7, 39.6, 38.3, 37.4, 33.5, 33.20, 33.15, 25.2, 24.8, 21.6, 20.6, 14.6, 14.4; **HRMS** (ESI-TOF) mass calculated for $[M+Na]^+$ $(C_{15}H_{20}NaO_2)^+$ expected *m/z* 255.1356; found *m/z* 255.1350.

5. Mechanistic Insights

5.1. Mechanistic insights into azetidine formation

After subjecting olefin **4a** to the standard photochemical reaction conditions (see **General Procedure A**), the vessel was removed from the water bath and the solvent *in-situ* exchanged to dry DMSO (1.6 mL), followed by addition of *p*-Anisidine (2.0 equiv.; 0.4 mmol; 49.3 mg), and 2,6-lutidine (3.0 equiv.; 0.6 mmol; 69.9 μL). DMSO selected over DMPU to circumvent solvent interference in the desired NMR region, whilst also still being an effective reaction media (see Table in section 3.1.2). The mixture was stirred (500 rpm) at 40 ℃ without irradiation and monitored at regular intervals by ^{1}H NMR and HRMS (**Figure S3 (a)**).

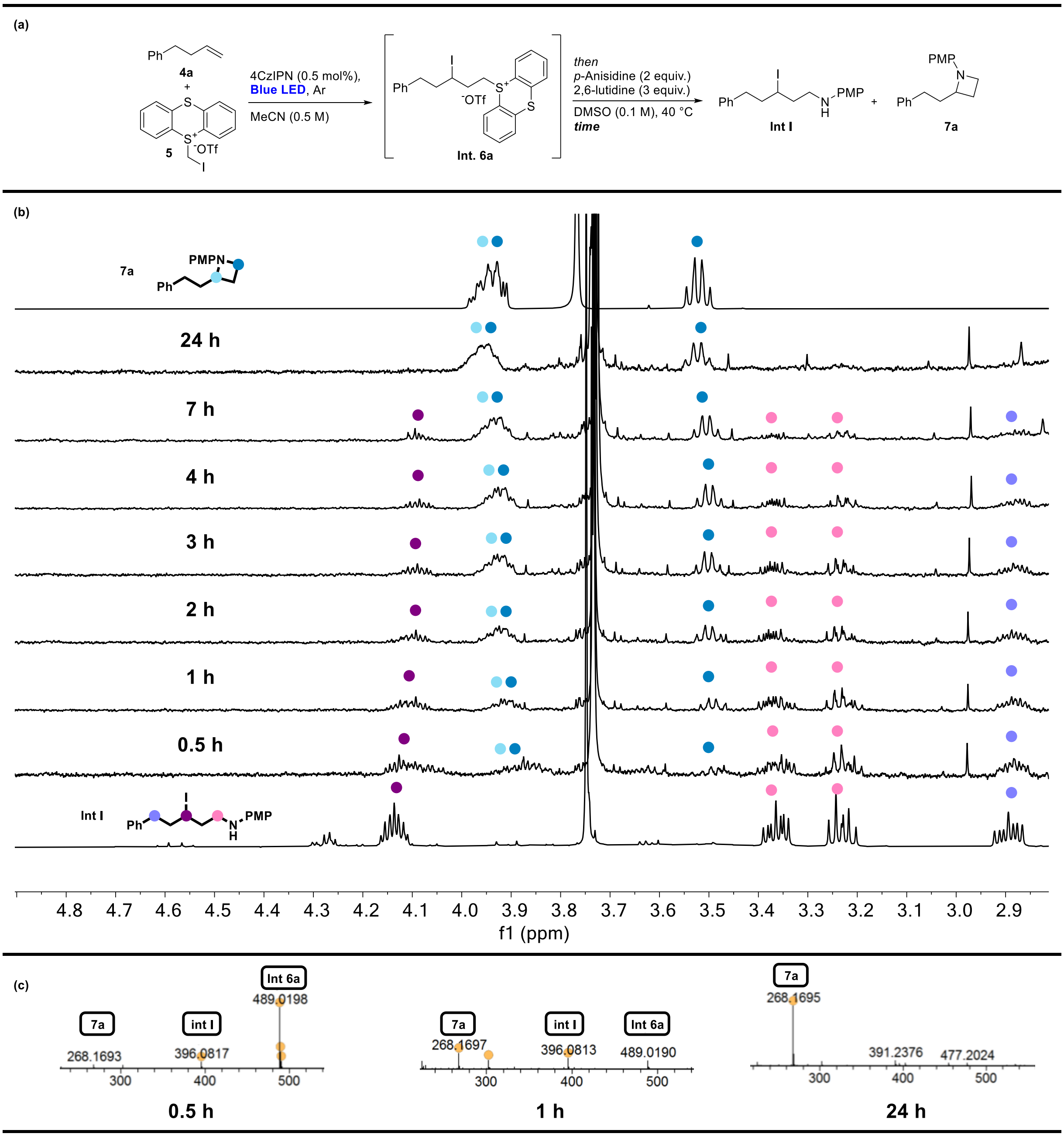

Figure S3 Reaction monitoring over time. (a) Reaction conditions. (b) ^{1}H NMR of crude reaction mixture at specified time interval. (c) HRMS of reaction mixture.

Figure S3 (b) stacks the ^{1}H NMRs of crude aliquots at regular time intervals, with reference gamma iodoamine **Int I**, and azetidine **7a** on the bottom and top respectively. **Figure S3 (c)** shows the HRMS of the crude at 0.5 h, 1 h, and after reaction completion, respectively. The initial substitution at the thianthrenium of dielectrophile **Int 6a** occurs rapidly, coinciding with formation of gamma-iodoamine **Int I,** from which intramolecular cyclisation (4-*exo-tet*) to azetidine **7a** proceeds comparatively slowly. No substitution at the iodo-position of the dielectrophile **Int 6a** is observed by both ^{1}H NMR and HRMS analysis. Hence, the process follows the expected order of substitutions, in which the primary sulfonium within the photochemical ATRA intermediate is first substituted by *p*-anisidine, followed by cyclization of the resulting intermediate **Int I** upon nucleophilic attack of the nitrogen centre on the secondary halide to give azetidine **7a**.

5.2. Mechanistic insights into oxetane formation

Experiments were carried out following **General Procedure C** (*vide supra*), and the reported crude NMR yields were recorded using phenanthrene (1 equiv.) as internal standard. A 15 µl aliquot was taken and placed in an NMR tube, diluted with neutralised $CDCl_3$ and subjected to ^{1}H NMR analysis. Integration of the multiplet at $\delta = 4.57 - 4.49$ (C-H proton in α-position to the oxygen) against the standard in the resultant ^{1}H NMR spectrum provided the NMR yields reported below.

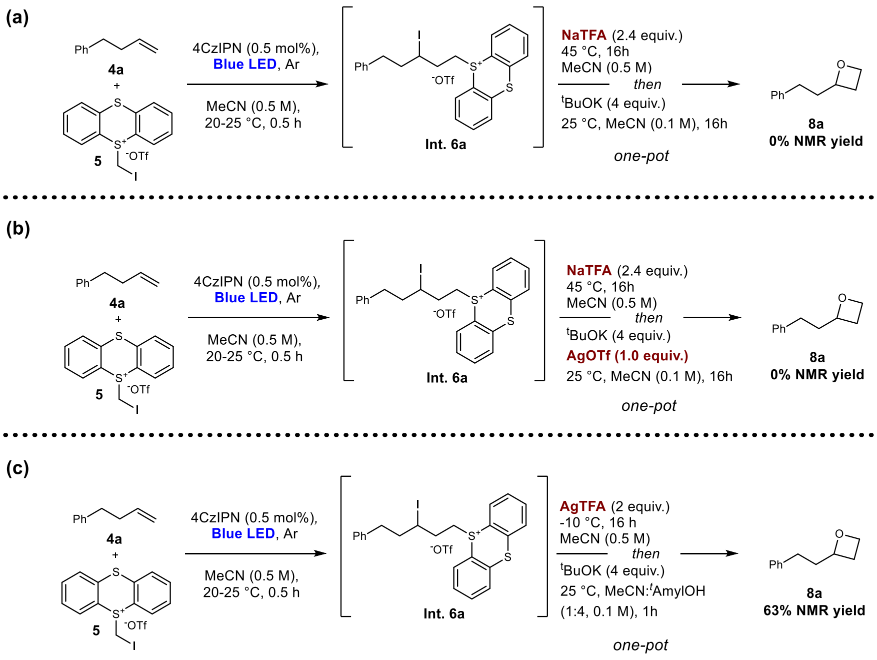

Scheme S4 Investigations into oxetane formation using NaTFA **(a)**, NaTFA/AgOTf **(b)** and AgTFA **(c)**, respectively. Reactions performed on 0.2 mmol scale, using **4a** (1.0 equiv.); ^{1}H-NMR yield using phenanthrene as internal standard. See section 4 for experiment procedure.

Initial studies into the formation of oxetane **8a** employed sodium trifluoroacetate (NaTFA) as a nucleophile, followed by the *in-situ* addition of potassium tert-butoxide (^{t}BuOK) to promote cyclization (**Scheme S4 (a)**). However, under these conditions, no oxetane product was detected.

Silver triflate (AgOTf) (**Scheme S4 (b)**) was added alongside the base to potentially enhance cyclization, however this afforded significant elimination and no desired oxetane. In contrast, when silver trifluoroacetate (AgTFA) was employed directly as the nucleophile, under optimized conditions, the desired oxetane **10a** was obtained in 63% yield (**Scheme S4 (c)**). This suggests that NaTFA and AgTFA proceed through formation of different key intermediates.

^{1}H-NMR analysis of the crude reaction mixtures, *prior to base addition,* confirmed the formation of two distinct intermediates, depending on whether NaTFA (**Int II – Scheme S5 (a)**) or AgTFA (**Int II– Scheme S5 (c)**) was used as the nucleophile. These are stacked around the crude photochemical reaction mixture *prior to the addition of the nucleophile* (intermediate **6a**), **Scheme S5 (b)**, for reference.

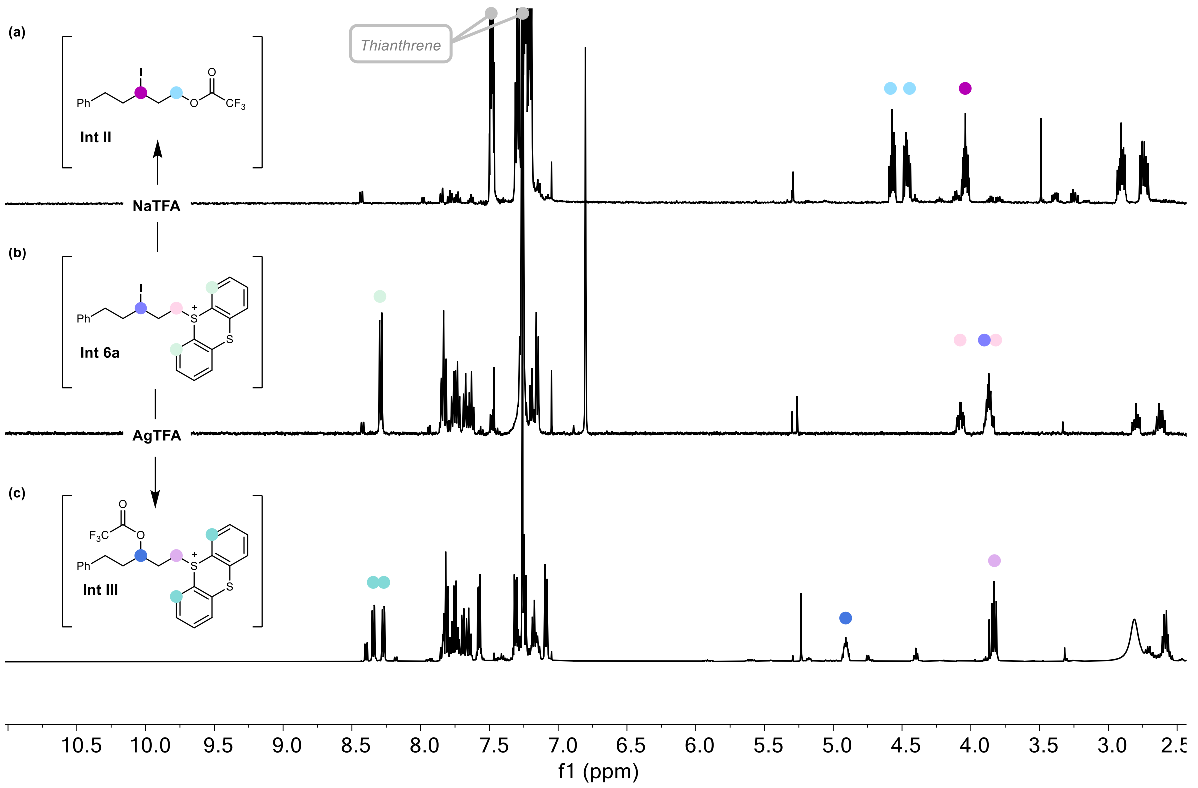

Figure S5 Comparison of the crude reaction mixtures obtained using NaTFA (conditions from **Figure S4, a) (a)**, and AgTFA (conditions from **Figure S4, c) (c)** as nucleophiles against the crude photochemical reaction mixture **(b)**.

In **Scheme S5 (a)**, **Int II** does not show the typical ^{1}H-NMR pattern of the aromatic thianthrenium protons (8.0 – 8.5 ppm). Instead, stoichiometric neutral thianthrene was observed (signals of thianthrene unambiguously assigned by comparison with a pure sample. This suggests the expected substitution of the primary sulfonium centre over the secondary iodide.

In contrast, in **Scheme S5 (c)**, **Int III** shows the typical ^{1}H-NMR pattern of the aromatic thianthrenium protons (8.0 – 8.5 ppm)—note that the two protons closer to the charged sulfonium (in violet in **Scheme S5 (c)**) are split in two signals due to their non-equivalent nature. A highly deshielded aliphatic proton (multiplet at 4.8-4.9 ppm) was also observed, tentatively assigned to the

CH in alpha position to the trifluoroacetate group. This suggests that substitution of the secondary iodide occurred over substitution of the thianthrenium center. To further confirm identity of **Ints. II** and **III** further studies with the aid of NMR spectroscopy and HRMS were performed.

Upon completion of the reaction conducted under the conditions reported in **Scheme S4 (a)**, the crude mixture was filtered through Celite, then concentrated under reduced pressure to remove volatiles. Despite efforts, it was not possible to detect the target compound by mass spectrometry (presumably due to instability under MS analysis conditions or lack of ionization under ESI), and the compound proved difficult to isolate by standard chromatographic techniques due to its susceptibility to hydrolysis. Therefore, a quick chromatographic purification was performed using a Pasteur pipette charged with silica gel (SiO_2; gradient 100 pentane to 98:2 pentane:EtOAc), with elution carried out for no more than 2 minutes, yielding a small sample (approx. 3 mg) of pure compound, along with large amounts of hydrolysis product. The pure material was immediately analysed by NMR spectroscopy using an instrument equipped with a cryoprobe. The ^{1}H-NMR, ^{13}C-NMR, COSY and HSQC spectra were recorded and are fully consistent with the proposed structure. Characteristic quartet patterns observed in the ^{13}C-NMR spectrum (**Scheme S6 (b)**) for both the CF_3 group and the carbonyl carbon—arising from their respective C–F couplings—provide further strong support for the structural assignment. The regioisomeric identity is further confirmed by the chemical shifts of the CH and CH_2 carbons, appearing at 31.0 ppm and 68.0 ppm, respectively. The assignments, established through COSY (**Scheme S7**) and HSQC (**Scheme S8**) correlations, are consistent with a secondary iodide and a primary trifluoroacetate moiety.[16,17]

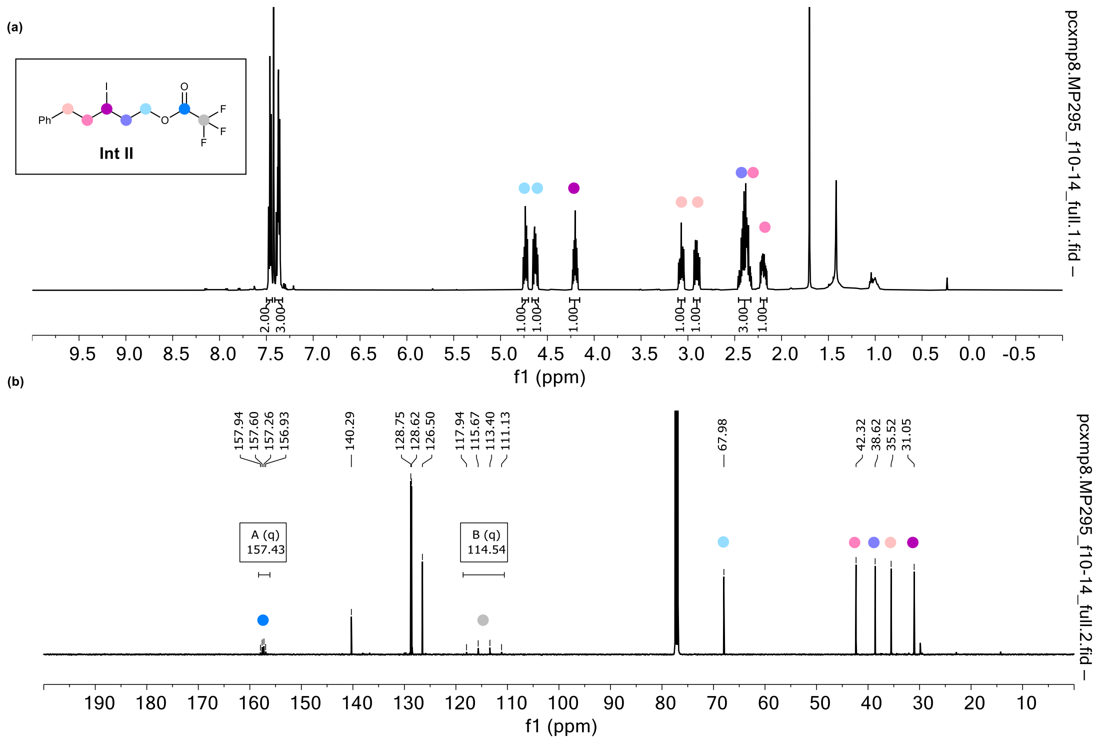

Scheme S6 (a) ^{1}H NMR spectrum and **(b)** ^{13}C NMR spectrum of **Int II.**

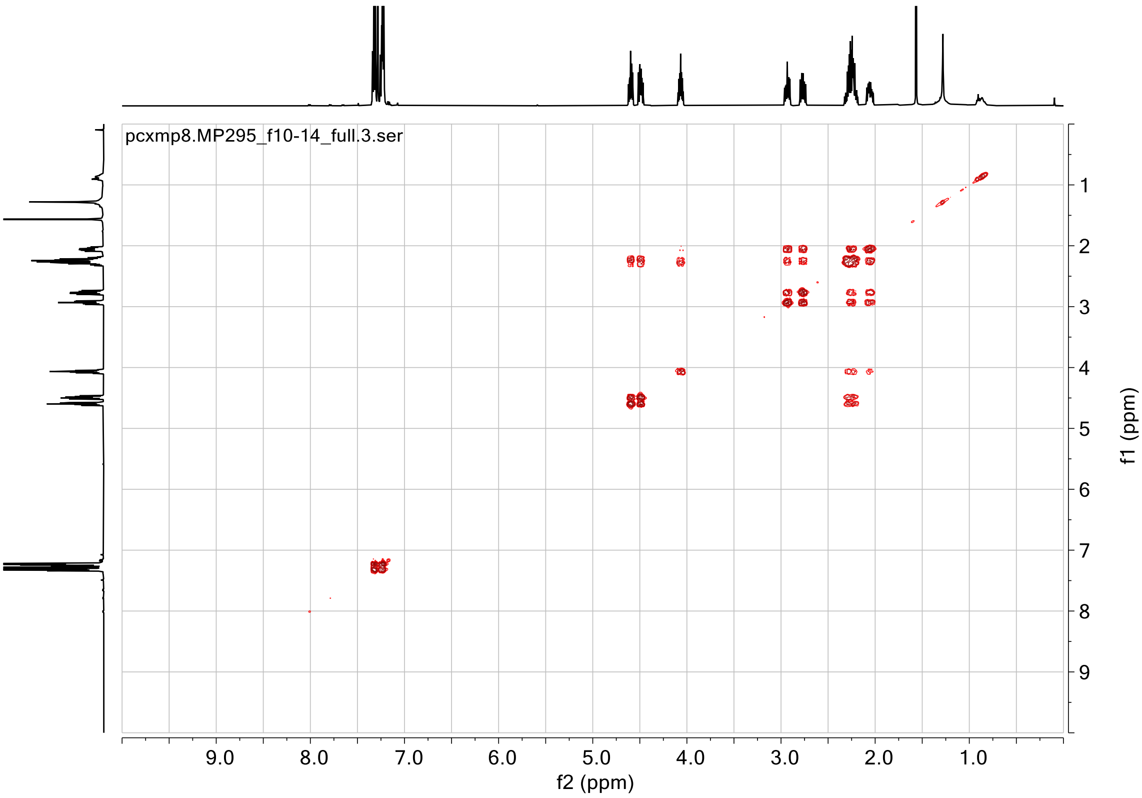

Scheme S7 COSY spectrum of **Int II.**

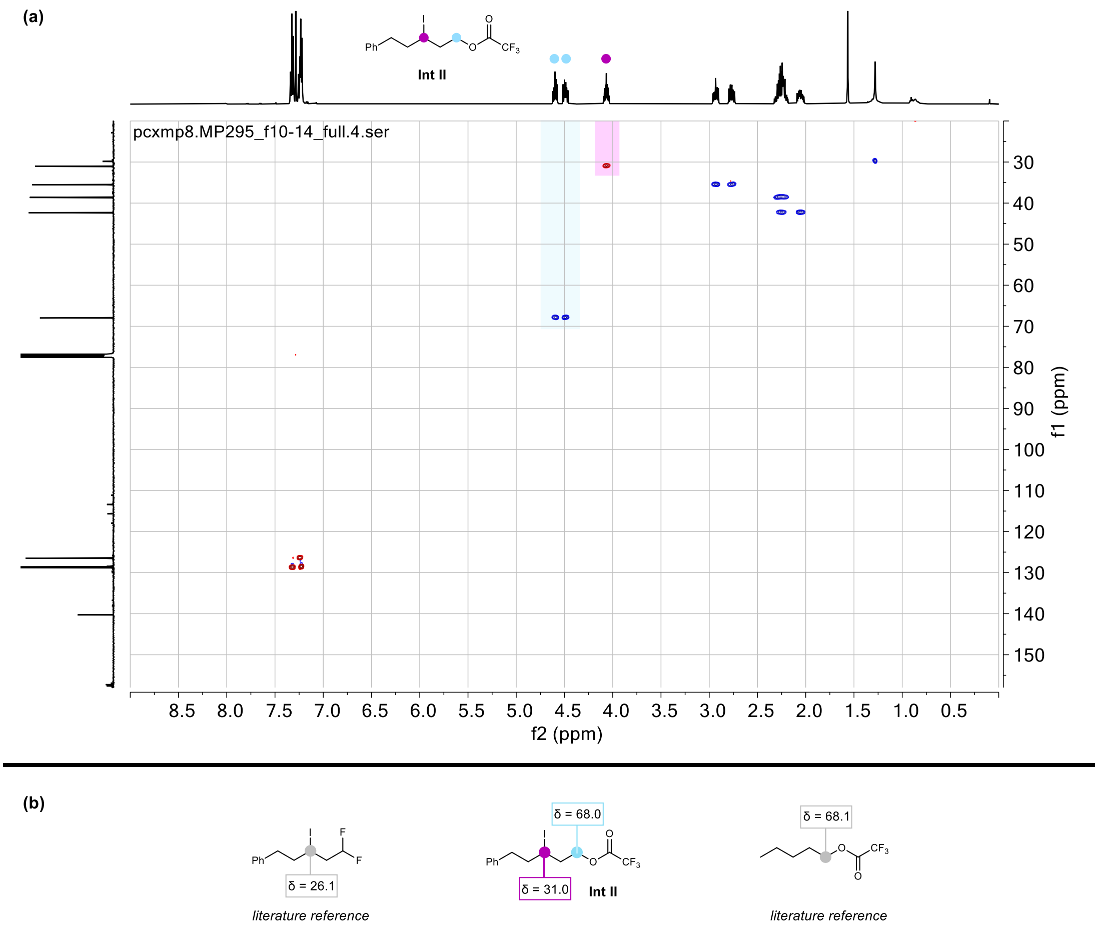

Scheme S8 (a) HSQC spectrum of **Int II (b)** Comparison with literature ^{13}C NMR data.[16,17]

Unlike the previous case, **Int III** could not be isolated by chromatographic purification. Its identification was therefore performed via HSQC experiments (**Figure S9 (a)**), the ^{13}C NMR signals consistent with literature data (**Figure S9 (b)**).[17,18] ESI-TOF HRMS mass spectrometry further unambiguously confirmed the identity of the compound (**Figure S9 (c)**).

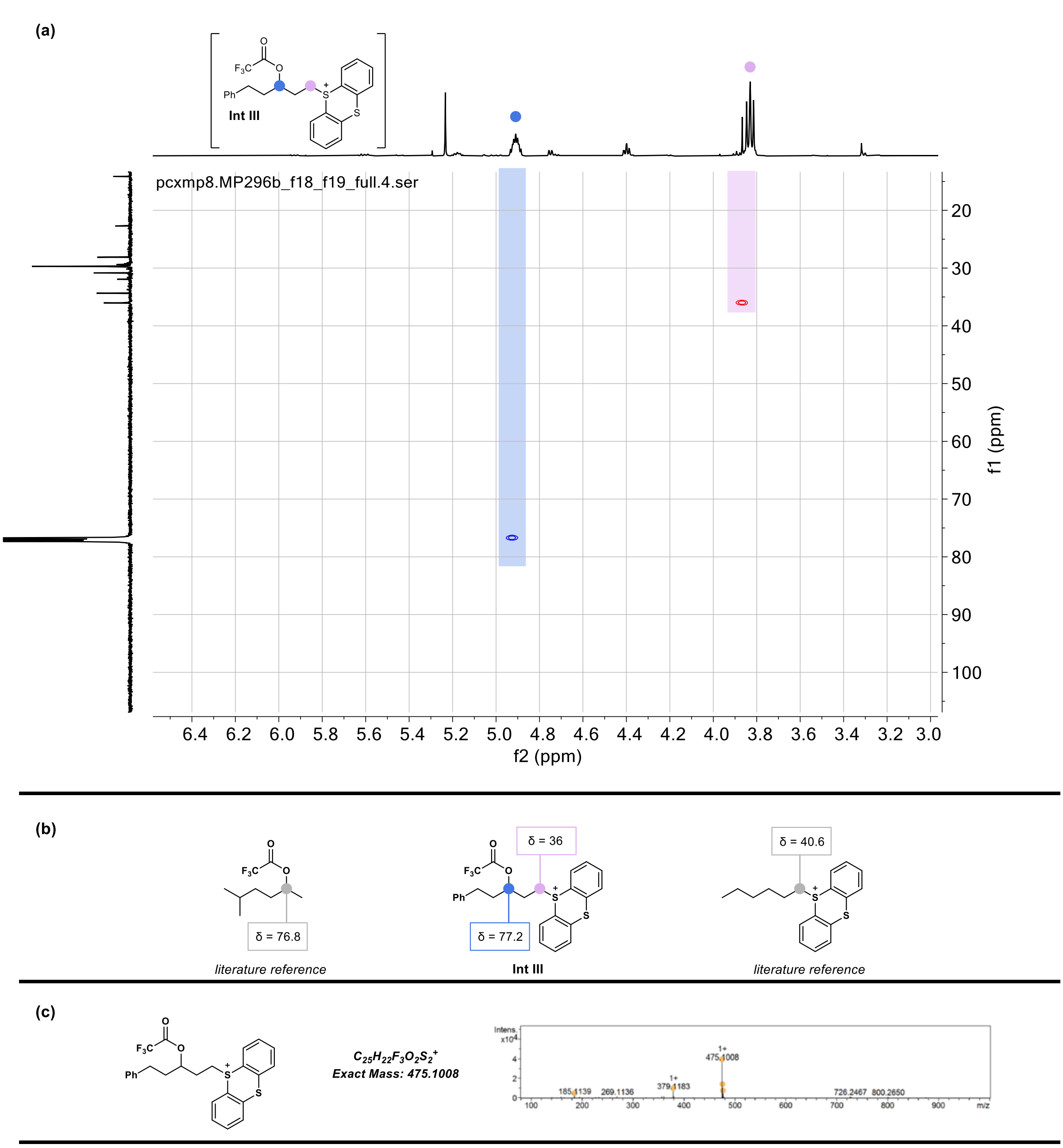

Scheme S9 (a) HSQC spectrum of **Int III (b)** Comparison with literature ^{13}C NMR data.[17,18] **(c)** HRMS of **Int III**

6. References and Notes

[1] Chu, J. C. K.; Rovis, T. *Nature* ***2016**, 539*, 272–275.

[2] Stang, E. M.; White, M. C. *J. Am. Chem. Soc.* **2011**, *133*, 14892–14895.

[3] Johnson, S.; Rahmani, R.; Drew, D. R.; Williams, M. J.; Wilkinson, M.; Tan, Y. H.; Huang, J. X.; Tonkin, C. J.; Beeson, J. G.; Baum, J.; Smith, B. J.; Baell, J. B. *J. Med. Chem.* **2016**, *59*, 10994–11005.

[4] Wang, H.; Cheng, F.; Li, M.; Peng, W.; Qu, J. *Langmuir* **2015**, 31, 3413–3421.

[5] Leicht, H.; Göttker-Schnetmann, I.; Mecking, S. *J. Am. Chem. Soc.* **2017**, *139*, 6823–6826.

[6] Basauri-Molina, M.; Verhoeven, D. G. A.; Van Schaik, A. J.; Kleijn, H.; Klein Gebbink, R. J. M. *Chem. Eur. J.* **2015**, 21, 15676–15685.

[7] Yang, C.-T.; Zhang, Z.-Q.; Tajuddin, H.; Wu, C.-C.; Liang, J.; Liu, J.-H.; Fu, Y.; Czyzewska, M.; Steel, P. G.; Marder, T. B.; Liu, L. *Angew. Chem.* **2012**, 124, 543–547.

[8] Srinivasan, S.; McGuigan, C.; Andrei, G.; Snoeck, R.; De Clercq, E.; Balzarini, J. Bioorg. Med. *Chem. Lett.* **2001**, 11, 391–393.

[9] Park, S. R.; Kim, C.; Kim, D. G.; Thrimurtulu, N.; Yeom, H. S.; Jun, J.; Shin, S.; Rhee, Y. H. *Org. Lett.* **2013**, 15, 1166–1169.

[10] Goundry, W. R. F.; Lee, V.; Baldwin, J. E. *Tet. Lett.* **2002**, 43, 2745–2747.

[11] Lindner, H.; Amberg, W. M.; Carreira, E. M. *J. Am. Chem. Soc.* **2023**, 145, 22347–22353.

[12] Reichle, M. A.; Breit, B. *Angew. Chem. Int. Ed.* **2012**, 51, 5730–5734.

[13] Amaya, T.; Rebek, J. *J. Am. Chem. Soc.* **2004**, 126, 6216–6217.

[14] Verkade, J. M. M.; van Hemert, L. J. C.; Quaedflieg, P. J. L. M.; Alsters, P. L.; van Delft, F. L.; Rutjes, F. P. J. T. *Tet. Lett.* **2006**, 47, 8109–8113.

[15] Varney, M. D.; Romines, W. H.; Boritzki, T.; Margosiak, S. A.; Bartlett, C.; Howland, E. J. J. *Heterocycl. Chem.* **1995**, 32, 1493–1498.

[16] Trifonov, A. L.; Panferova, L. I.; Levin, V. V.; Kokorekin, V. A.; Dilman, A. D. *Org. Lett.* **2020**, 22, 2409–2413.

[17] Le, T. V.; Romero, I.; Daugulis, O. *Chem. Eur. J.* **2023**, 29, e202301672

[18] Zhang, J.; Jiao, M.; Lu, Z.; Lu, H.; Wang, M.; Shi, Z. *Angew. Chem. Int. Ed.* **2024**, 63, e202409862.

7. NMR Spectra

5-(iodomethyl)-5H-thianthren-5-ium triflate (5)

^{1}H-NMR (CD_3CN, 500 MHz)

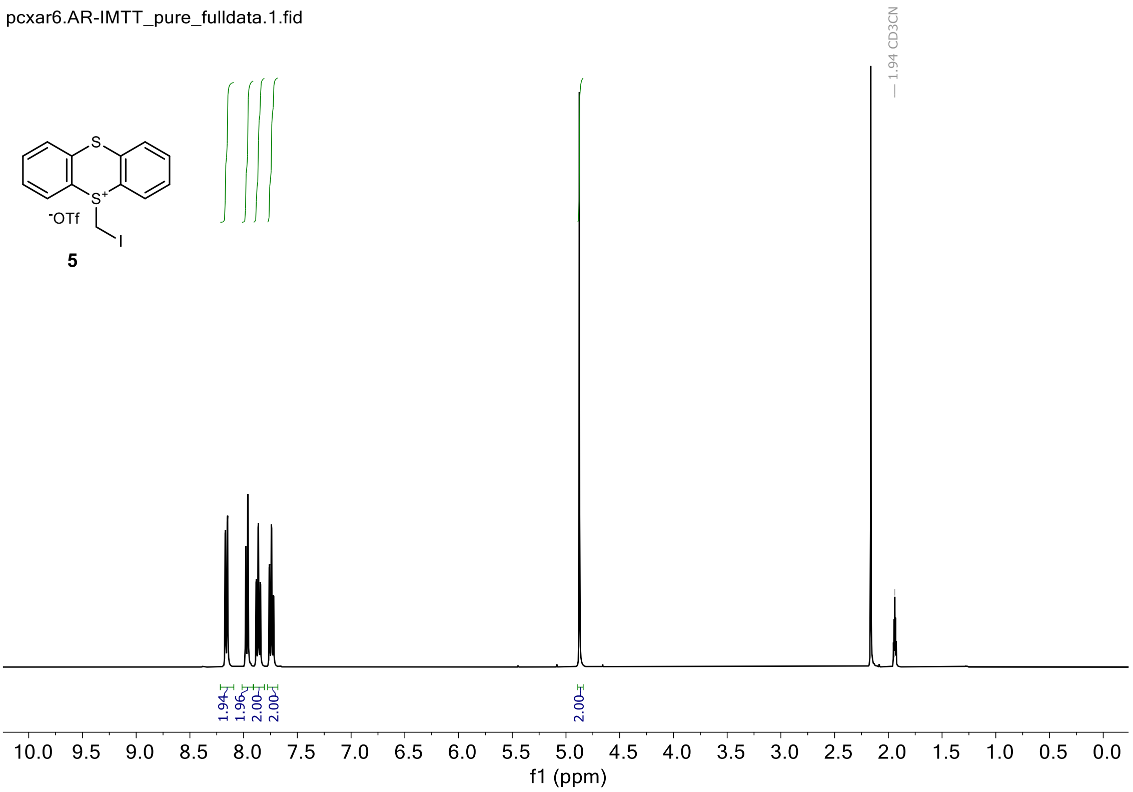

^{13}C-NMR (CD_3CN, 126 MHz)

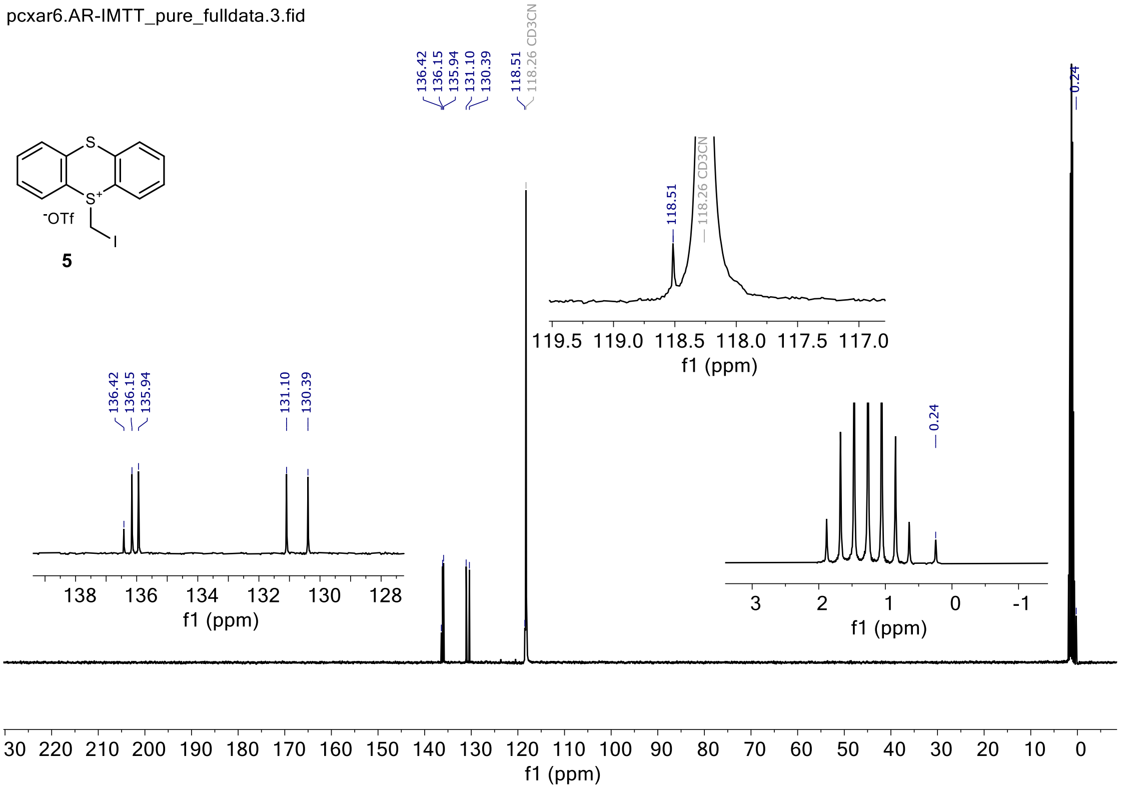

^{19}F-NMR (CD_3CN, 376 MHz)

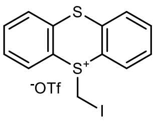

5

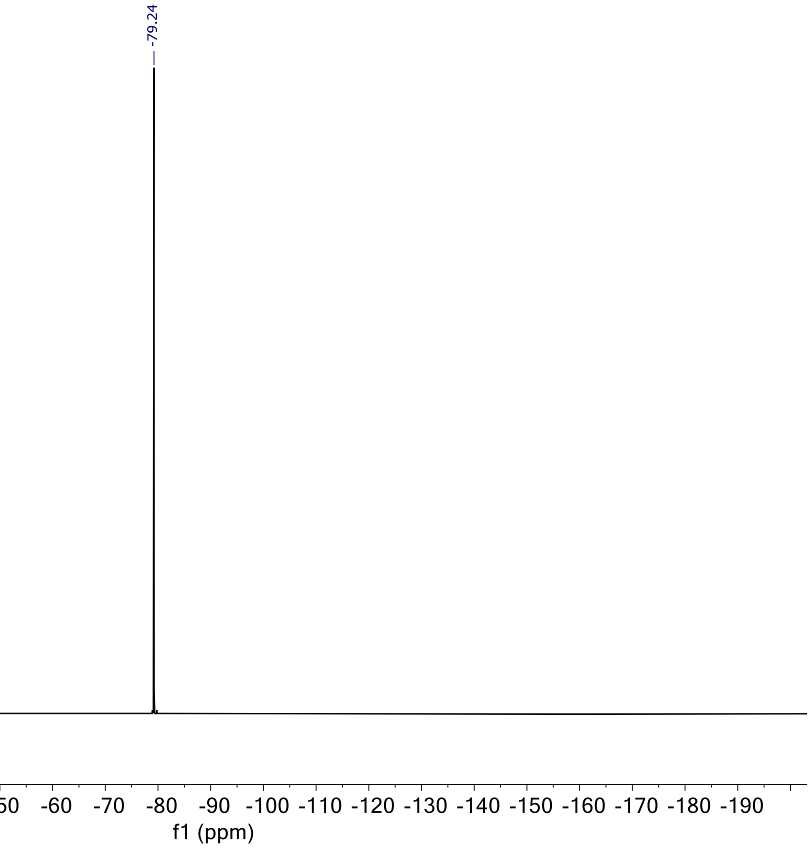

dodec-11-enenitrile (4b)

^{1}H-NMR ($CDCl_3$, 500 MHz)

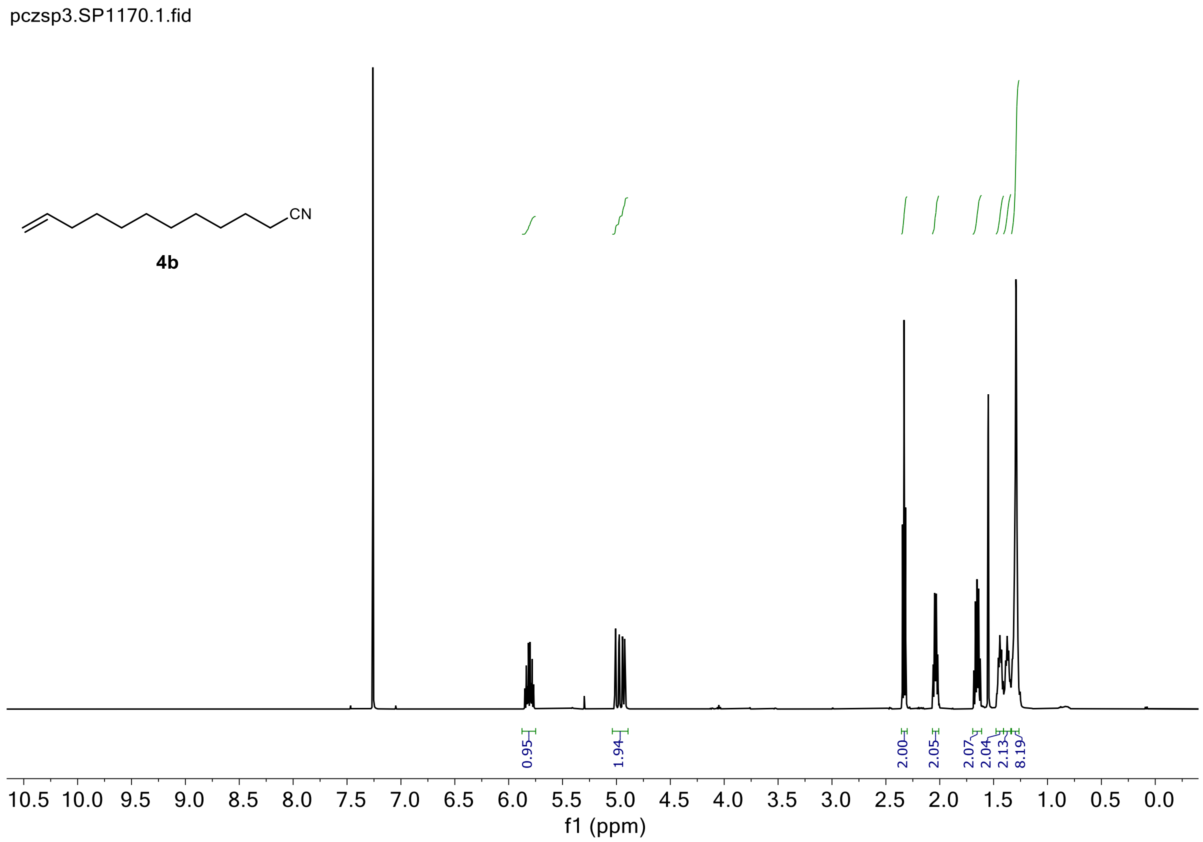

^{13}C-NMR ($CDCl_3$, 126 MHz)

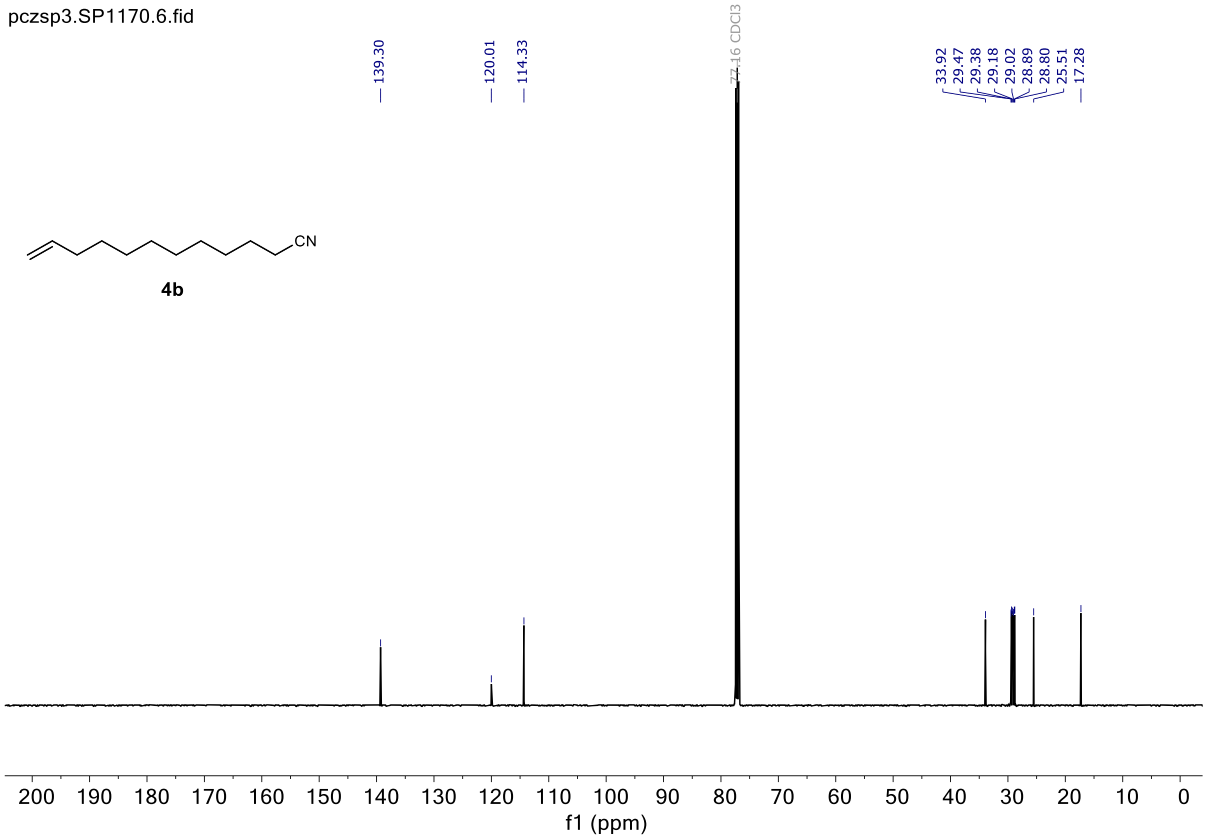

4,4,5,5-tetramethyl-2-(undec-10-en-1-yl)-1,3,2-dioxaborolane (4q)

^{1}H-NMR ($CDCl_3$, 500 MHz)

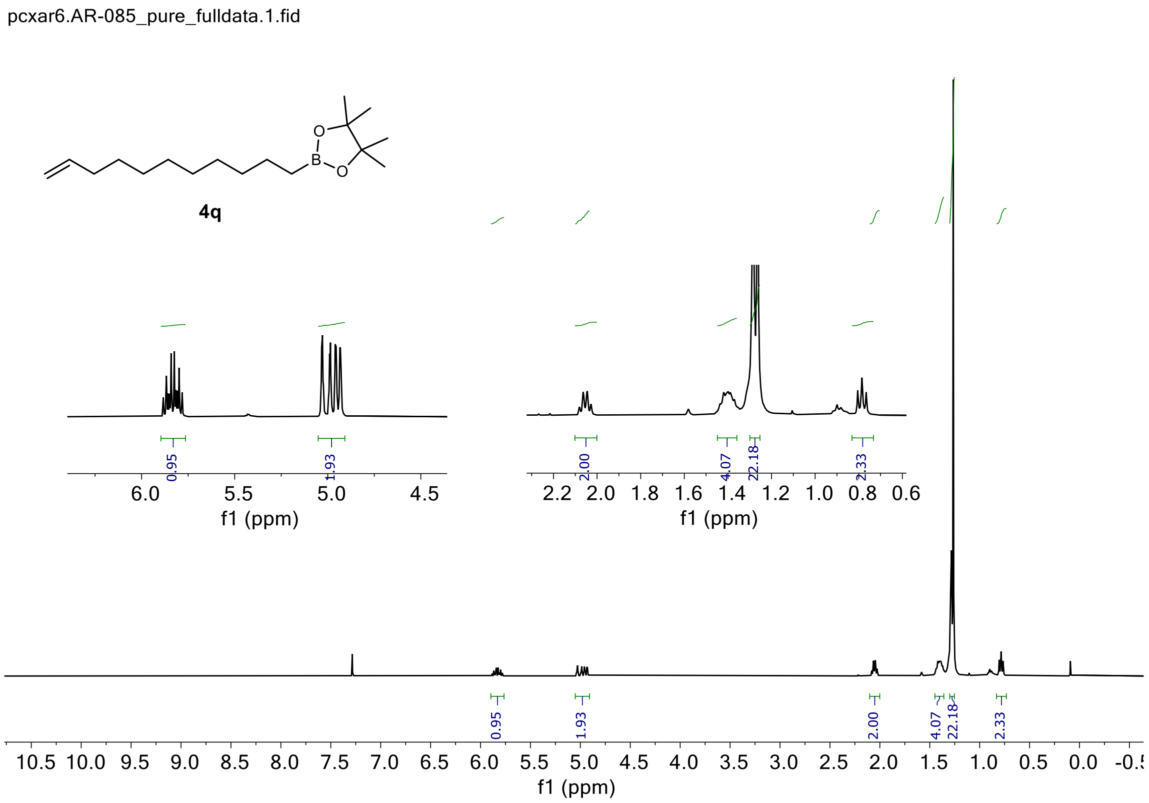

^{13}C-NMR ($CDCl_3$, 126 MHz)

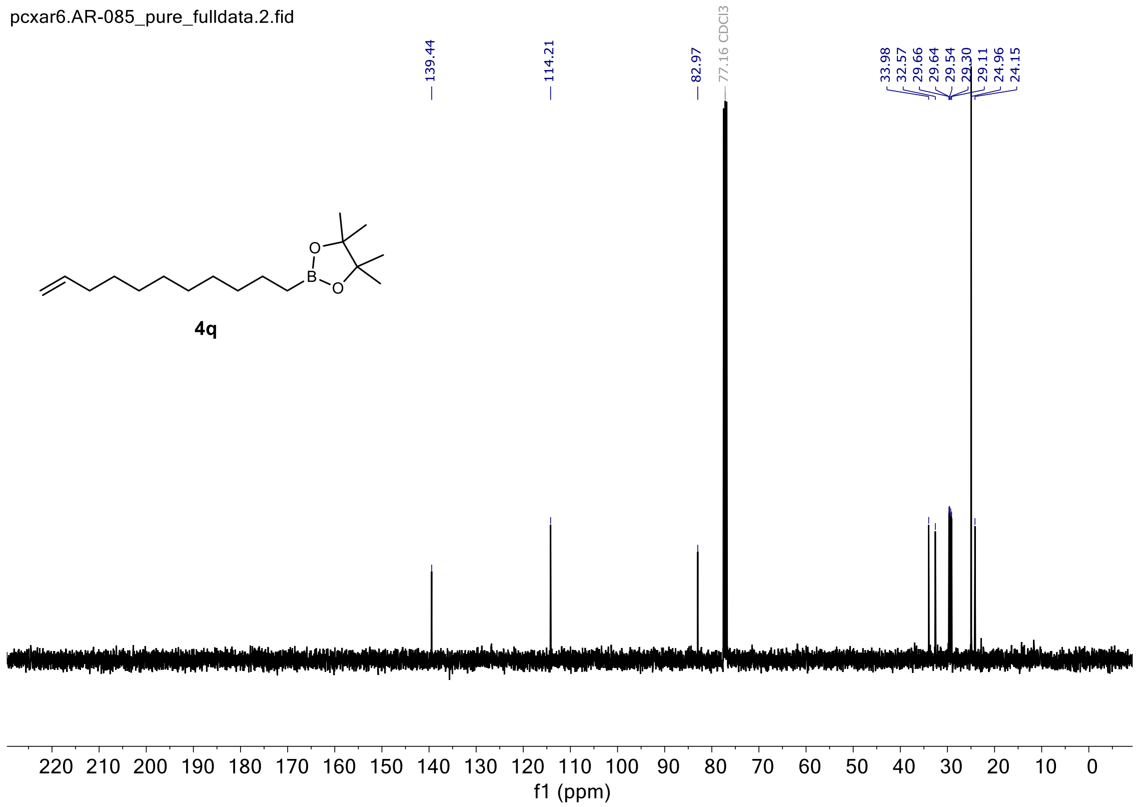

<u>^{11}B-NMR ($CDCl_3$, 128 MHz)</u>

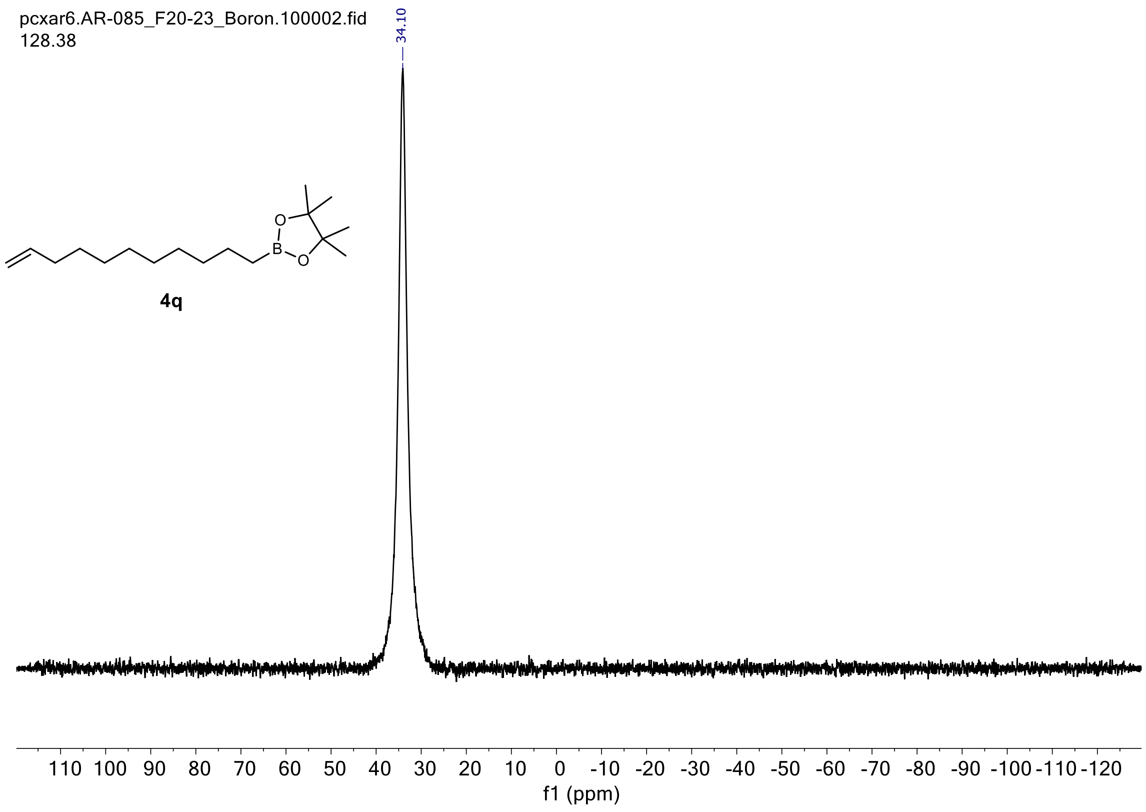

4q

(3*R*,8*R*,9*S*,10*S*,13*R*,14*S*,17*R*)-10,13-dimethyl-17-((*R*)-pent-4-en-2-yl)hexadecahydro-1*H*-cyclopenta[*a*]phenanthren-3-ol (4v)

^{1}H-NMR ($CDCl_3$, 500 MHz)

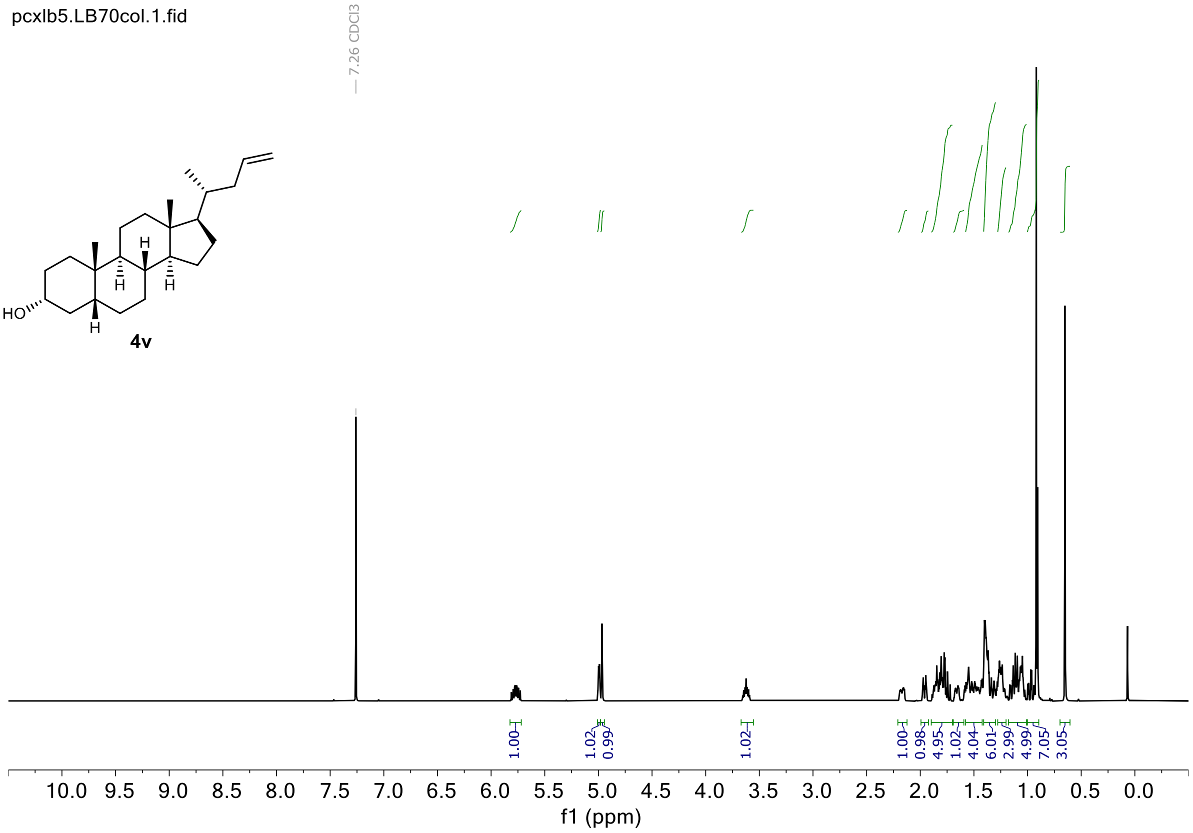

^{13}C-NMR ($CDCl_3$, 126 MHz)

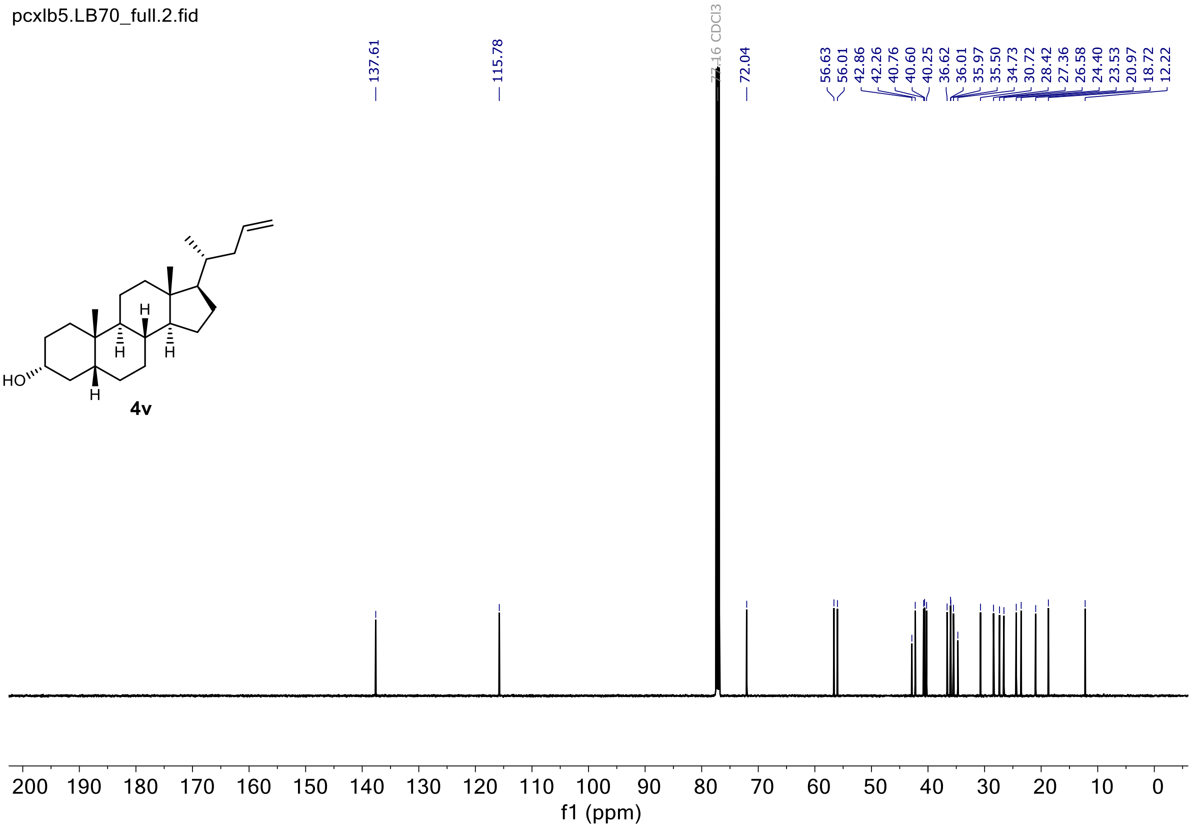

1-(4-methoxyphenyl)-2-phenethylazetidine (7a)

^{1}H-NMR ($CDCl_3$, 500 MHz)

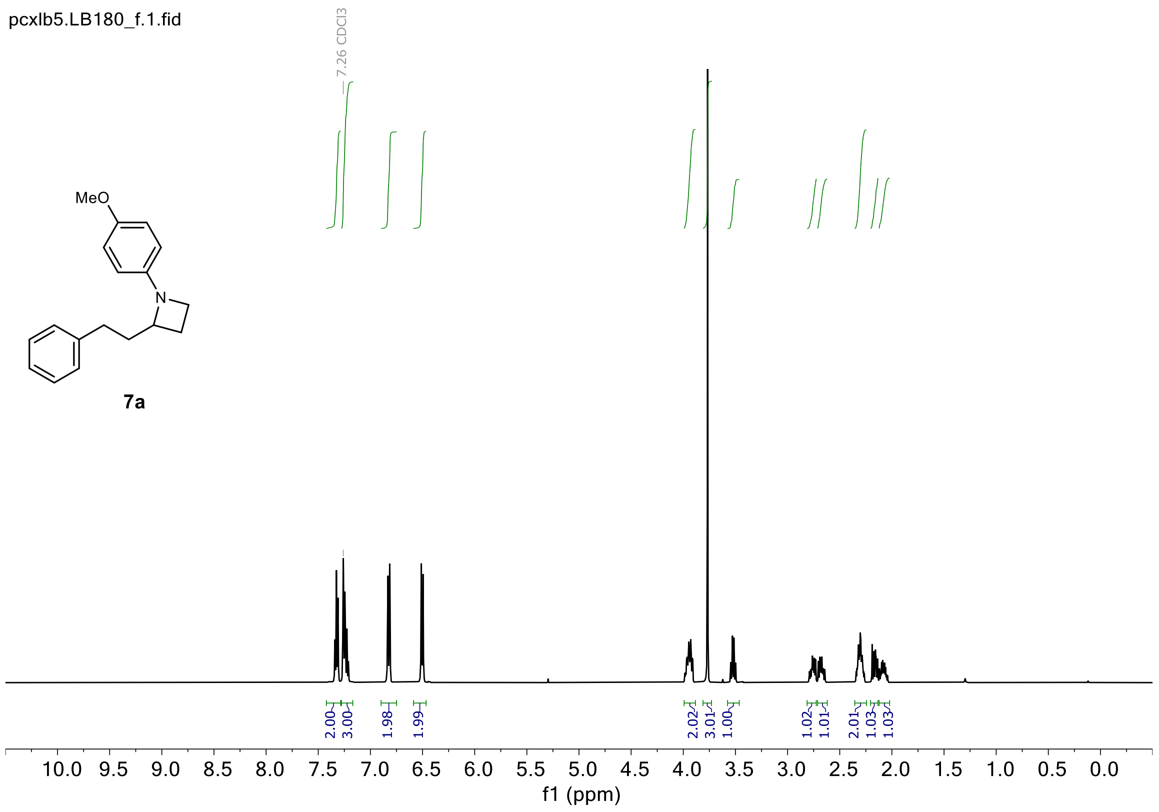

^{13}C-NMR ($CDCl_3$, 126 MHz)

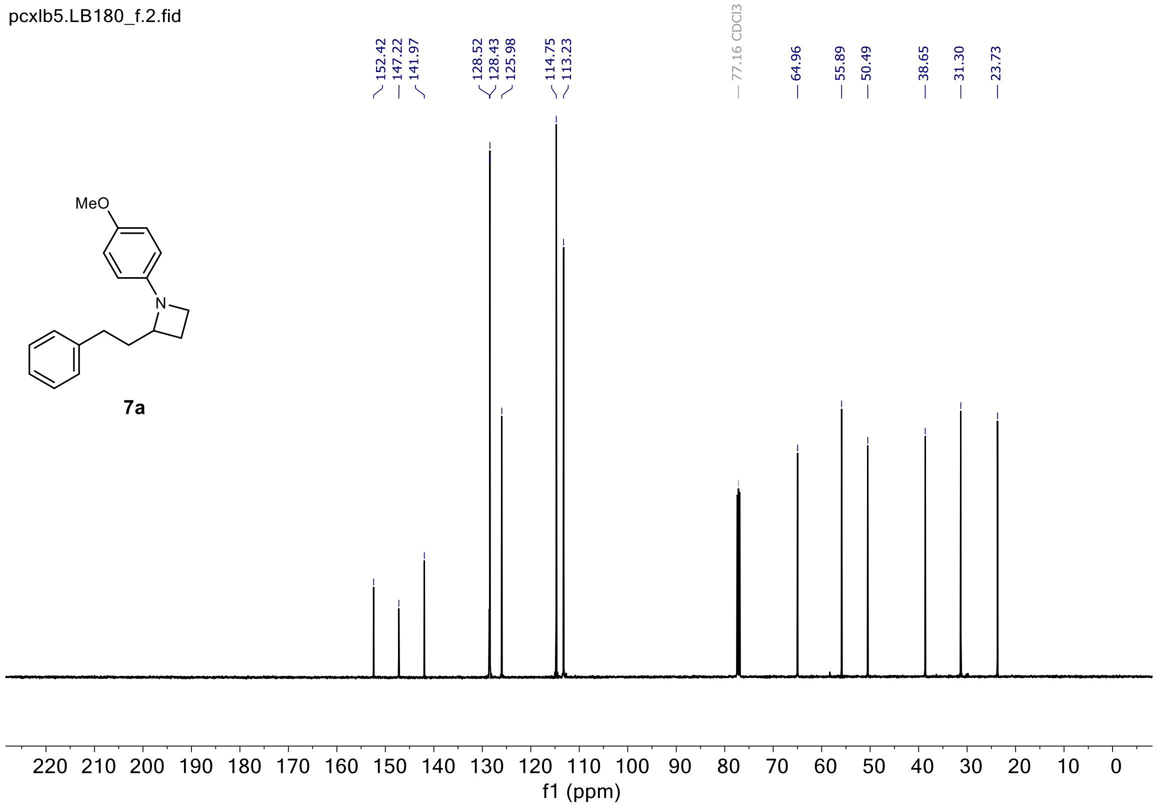

10-(1-(4-methoxyphenyl)azetidin-2-yl)decanenitrile (7b)

^{1}H-NMR (CDCl$_3$, 500 MHz)

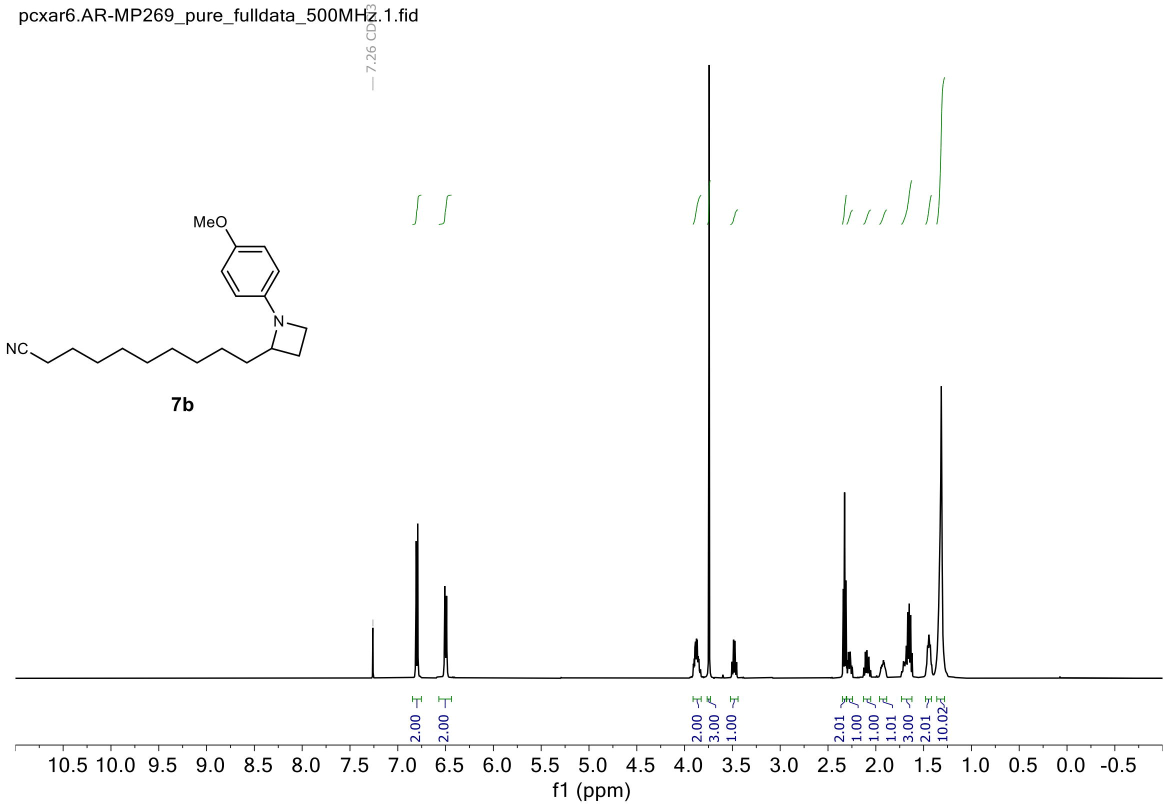

^{13}C-NMR (CDCl$_3$, 126 MHz)

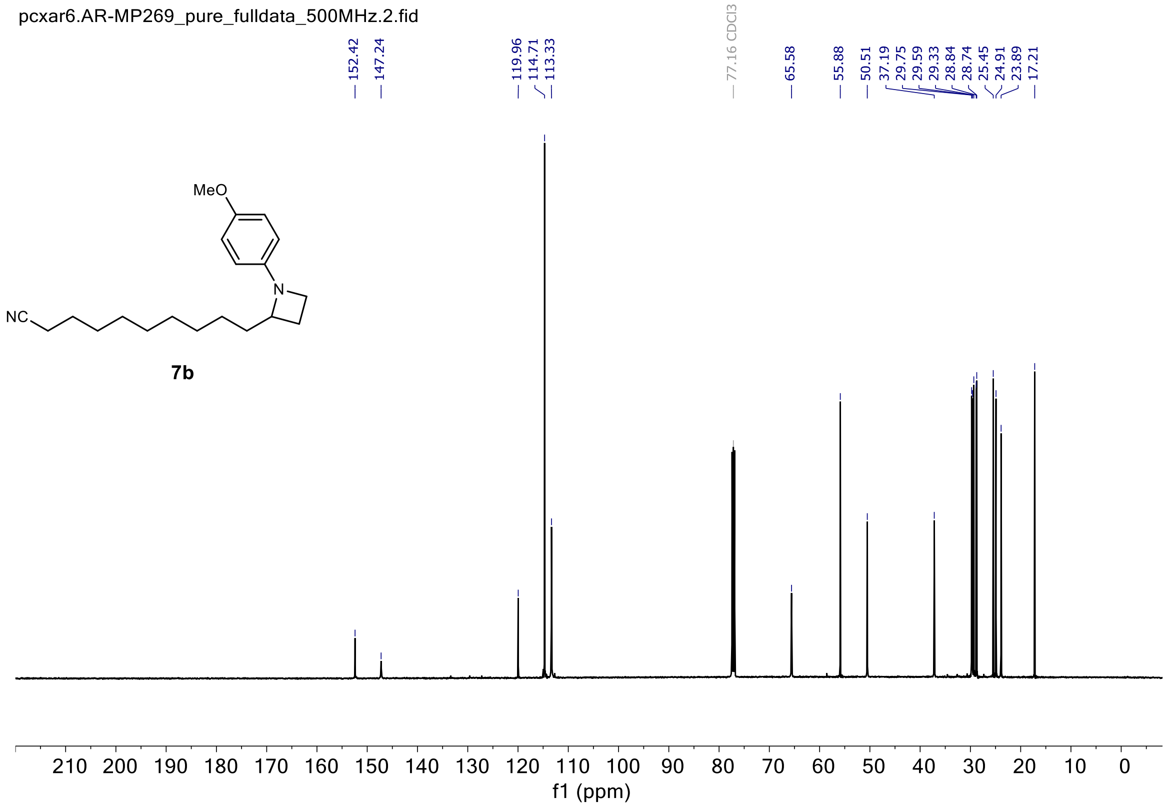

methyl 9-(1-(4-methoxyphenyl)azetidin-2-yl)nonanoate (7c)

^{1}H-NMR ($CDCl_3$, 500 MHz)

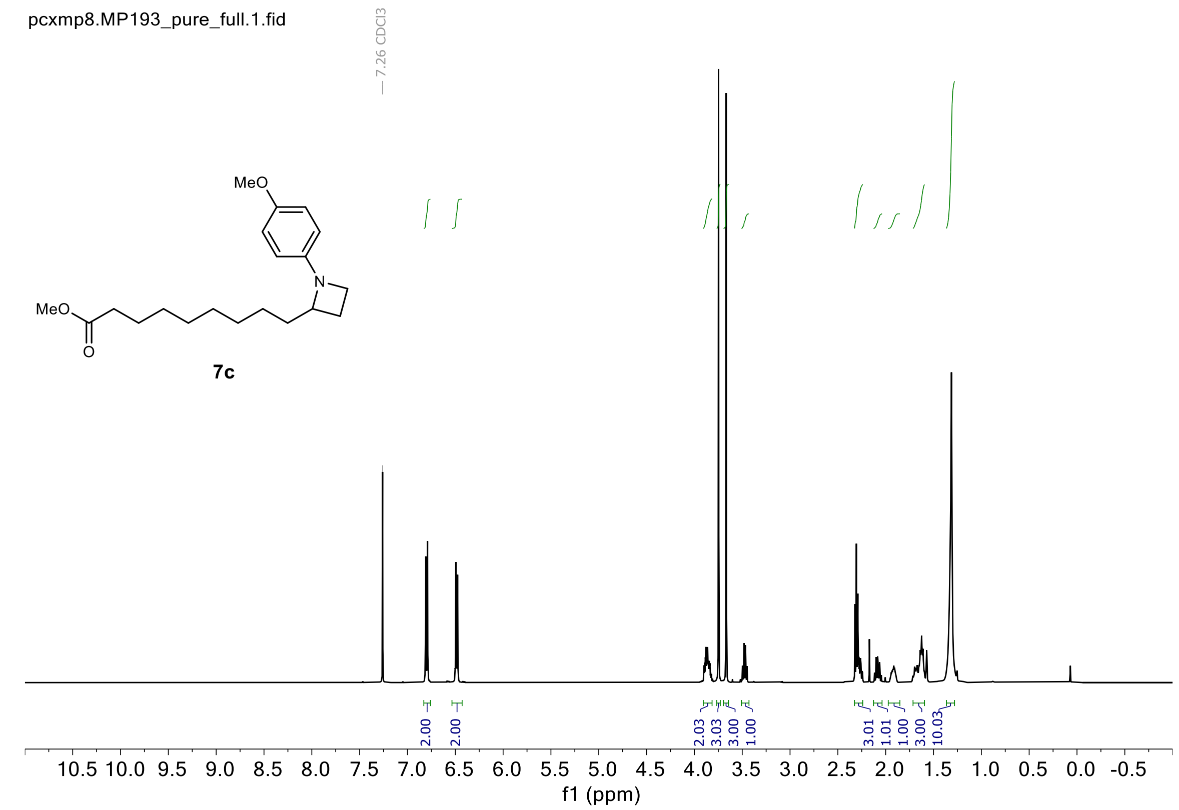

^{13}C-NMR ($CDCl_3$, 126 MHz)

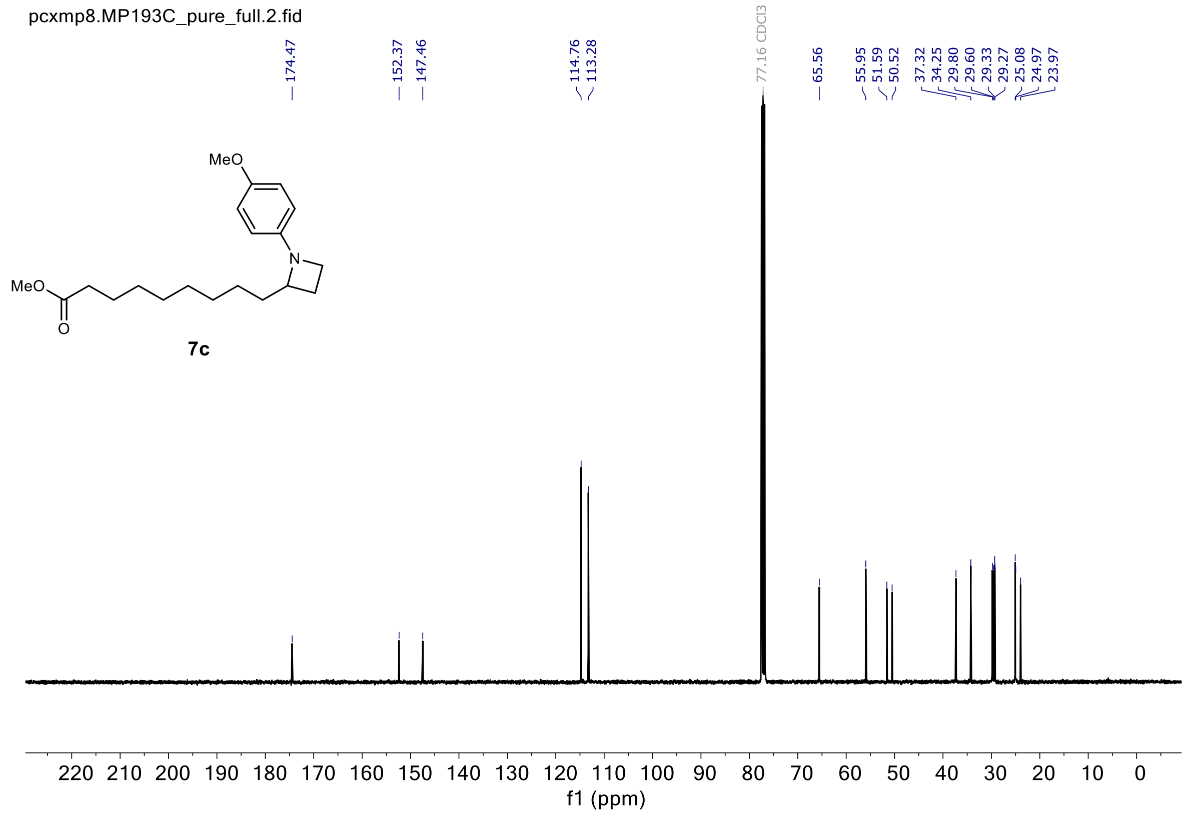

9-(1-(4-methoxyphenyl)azetidin-2-yl)nonanamide (7d)

^{1}H-NMR ($CDCl_3$, 500 MHz)

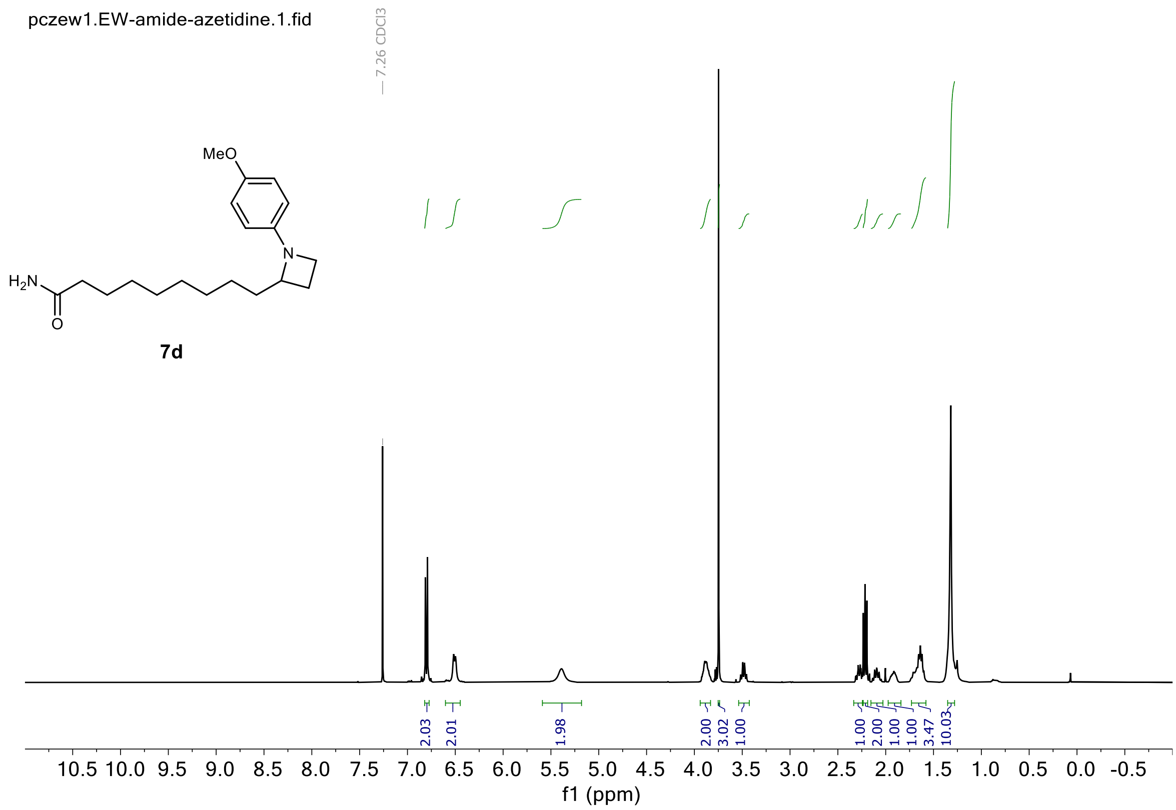

^{13}C-NMR ($CDCl_3$, 126 MHz)

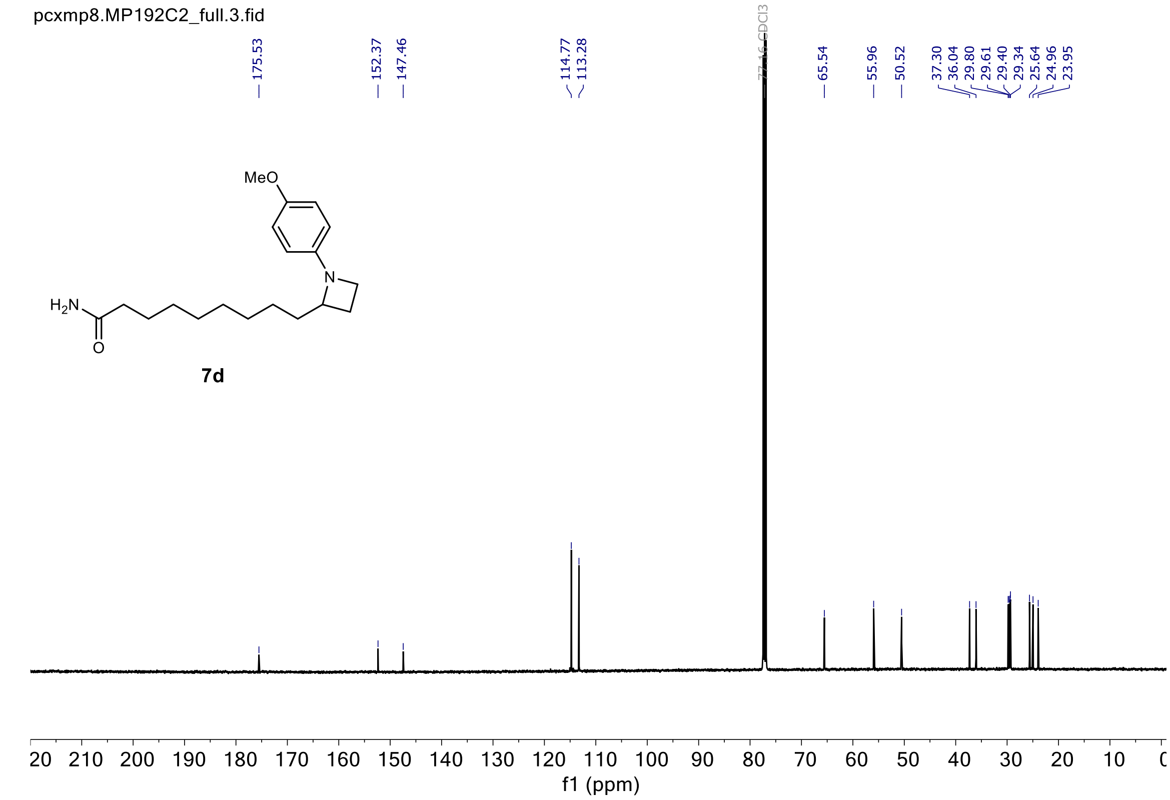

9-(1-(4-methoxyphenyl)azetidin-2-yl)nonanoic acid (7e)

^{1}H-NMR ($CDCl_3$, 500 MHz)

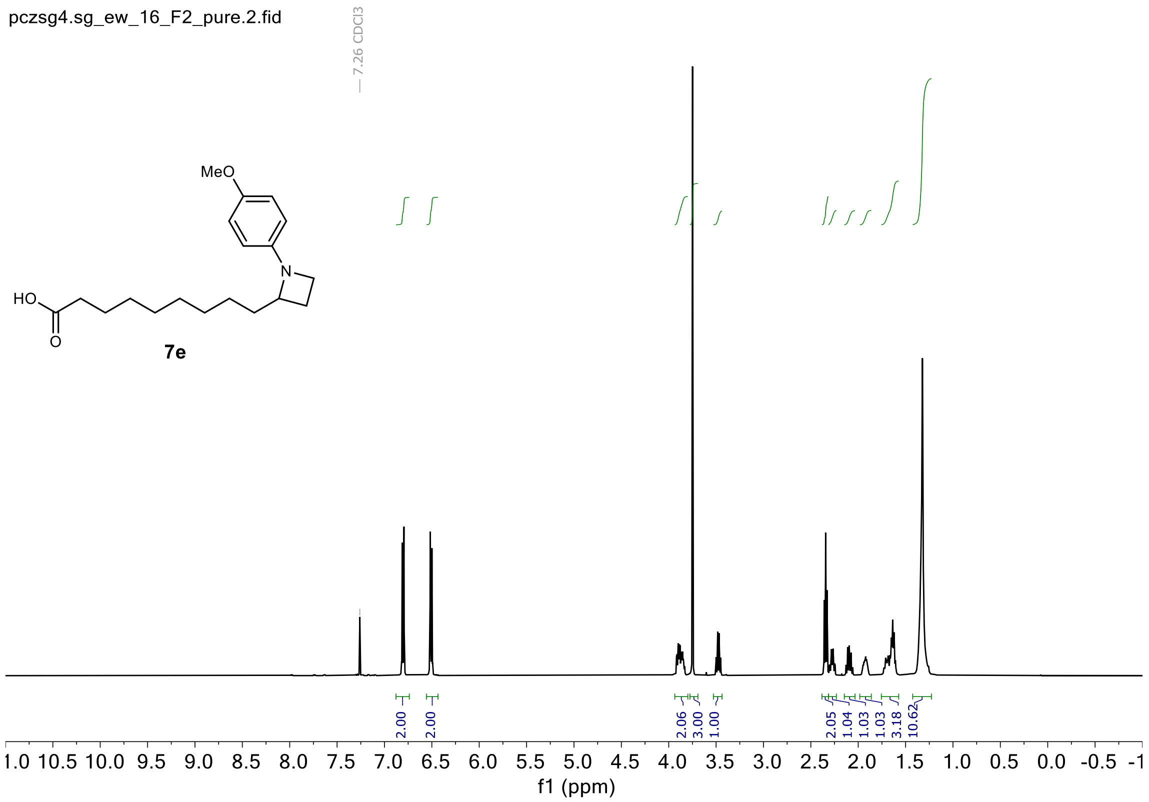

^{13}C-NMR ($CDCl_3$, 126 MHz)

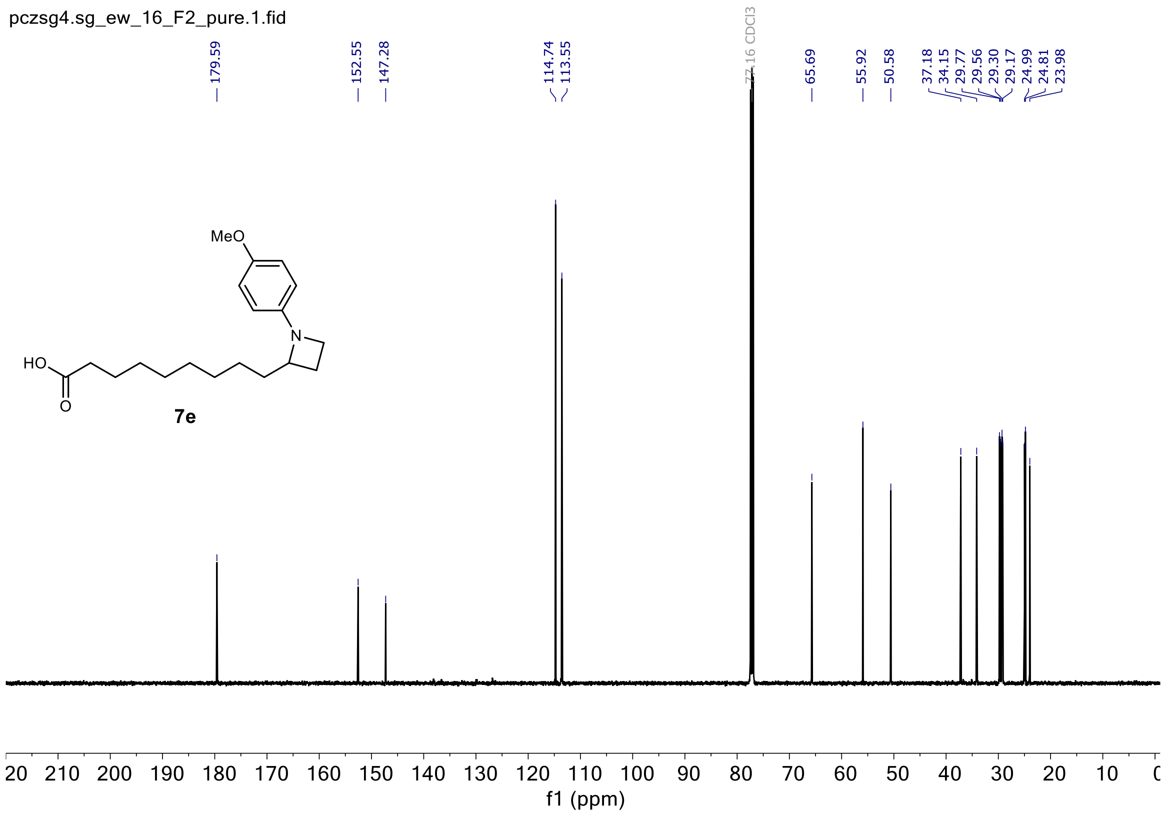

9-(1-(4-methoxyphenyl)azetidin-2-yl)nonan-1-amine (7f)

^{1}H-NMR ($CDCl_3$, 500 MHz)

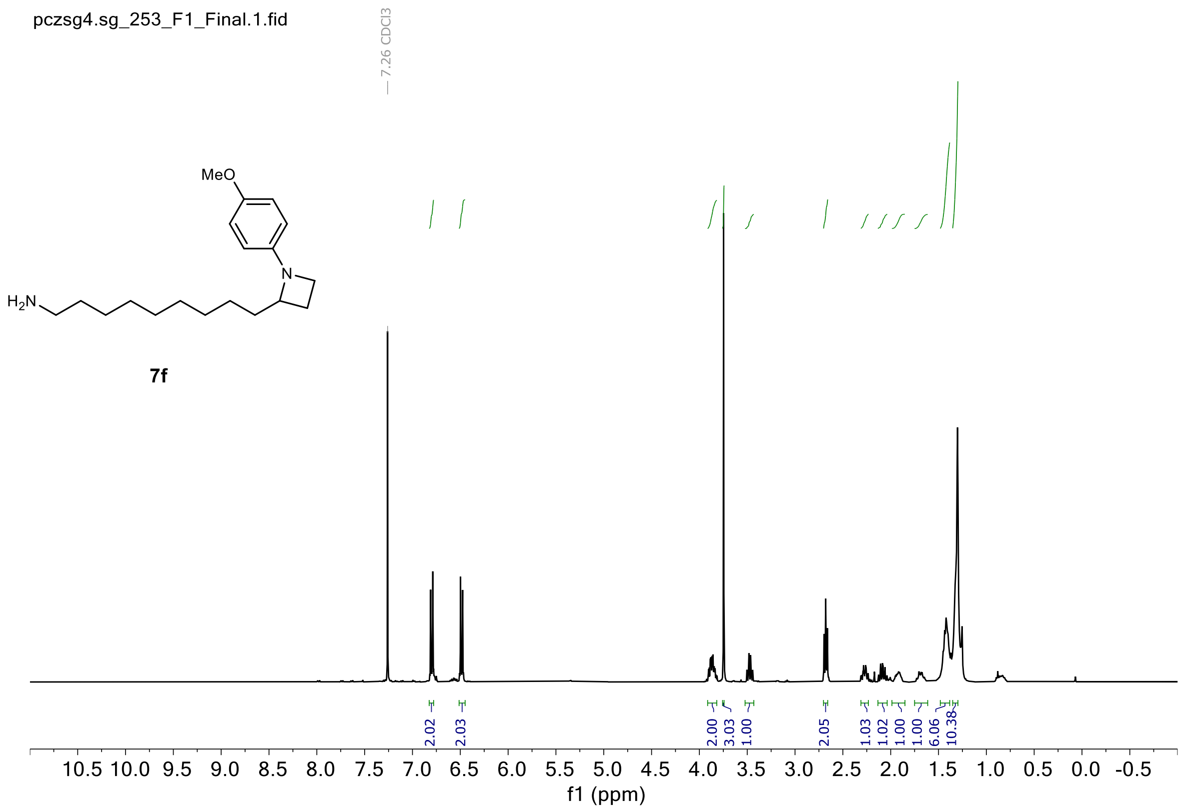

^{13}C-NMR ($CDCl_3$, 126 MHz)

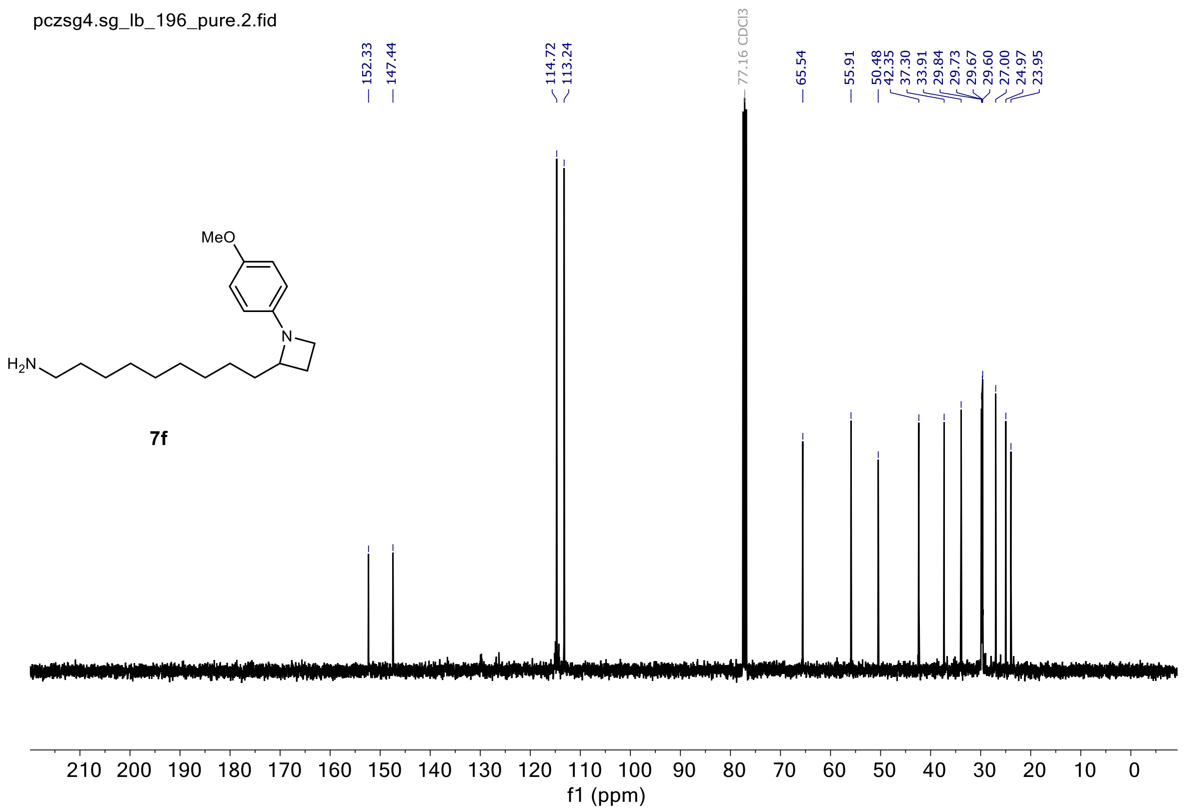

8-(1-(4-methoxyphenyl)azetidin-2-yl)octan-1-ol (7g)

^{1}H-NMR ($CDCl_3$, 500 MHz)

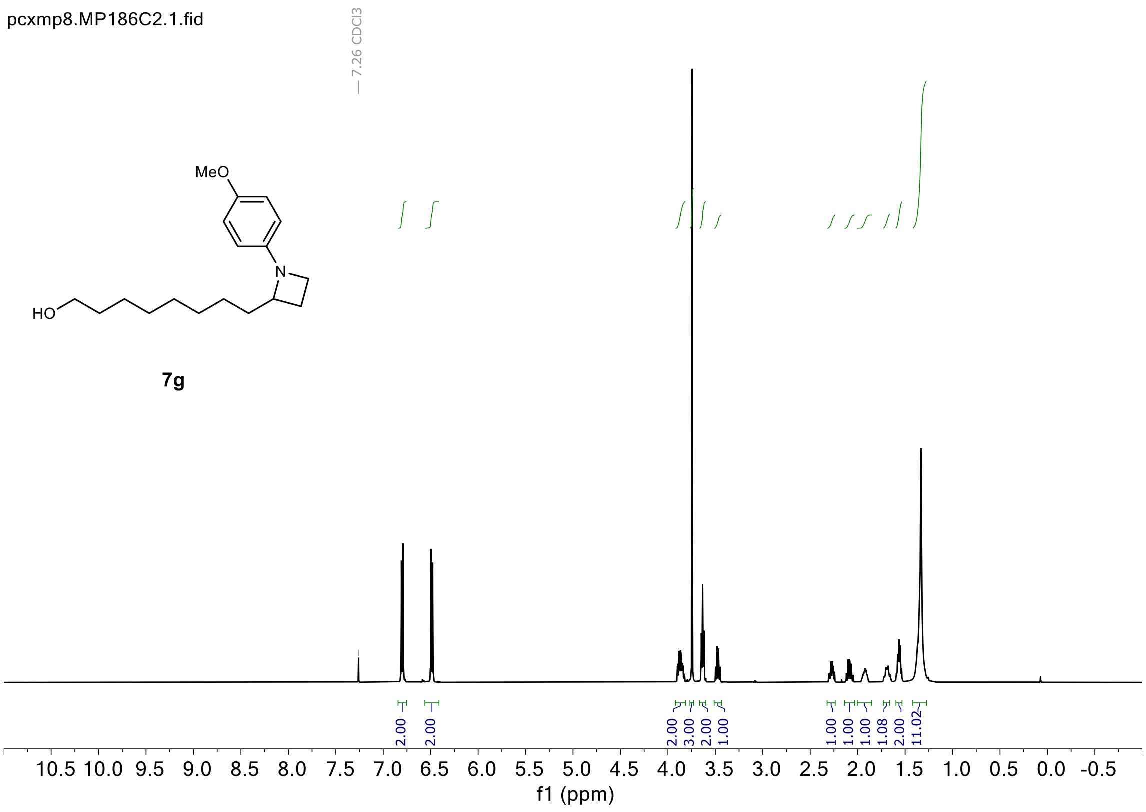

^{13}C-NMR ($CDCl_3$, 126 MHz)

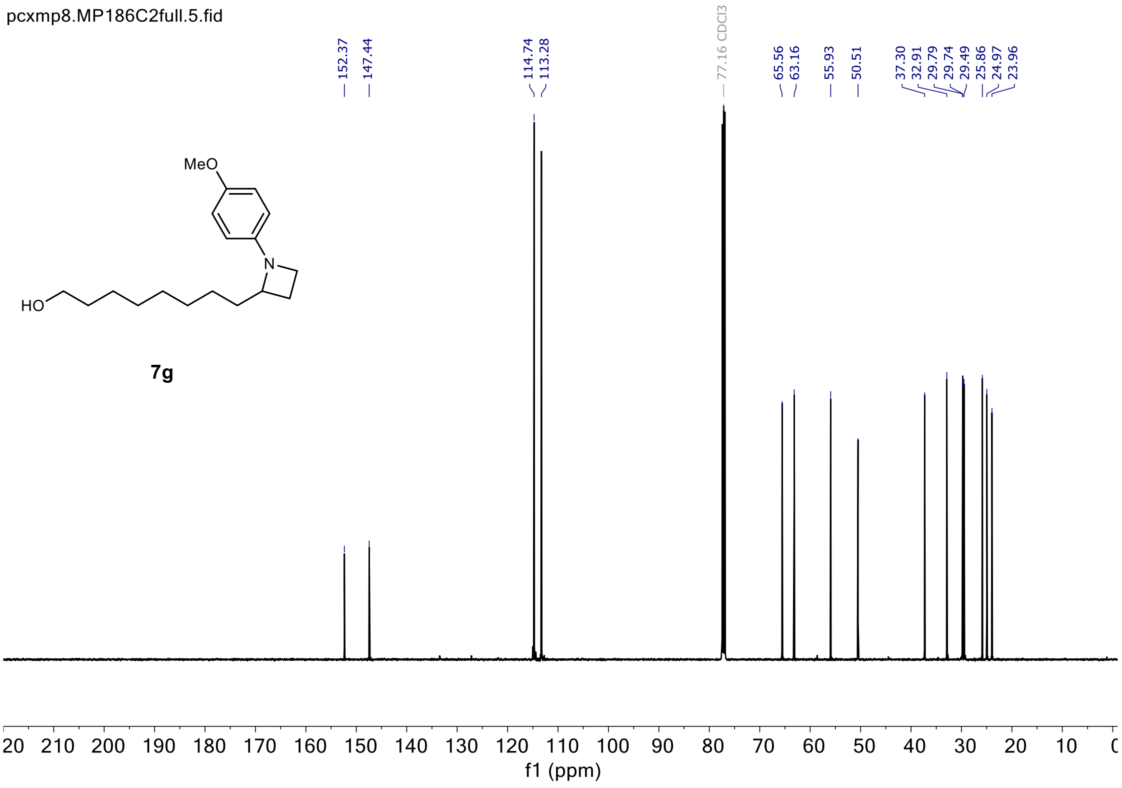

3-(1-(4-methoxyphenyl)azetidin-2-yl)propan-1-ol (7h)

^{1}H-NMR (CDCl$_3$, 400 MHz)

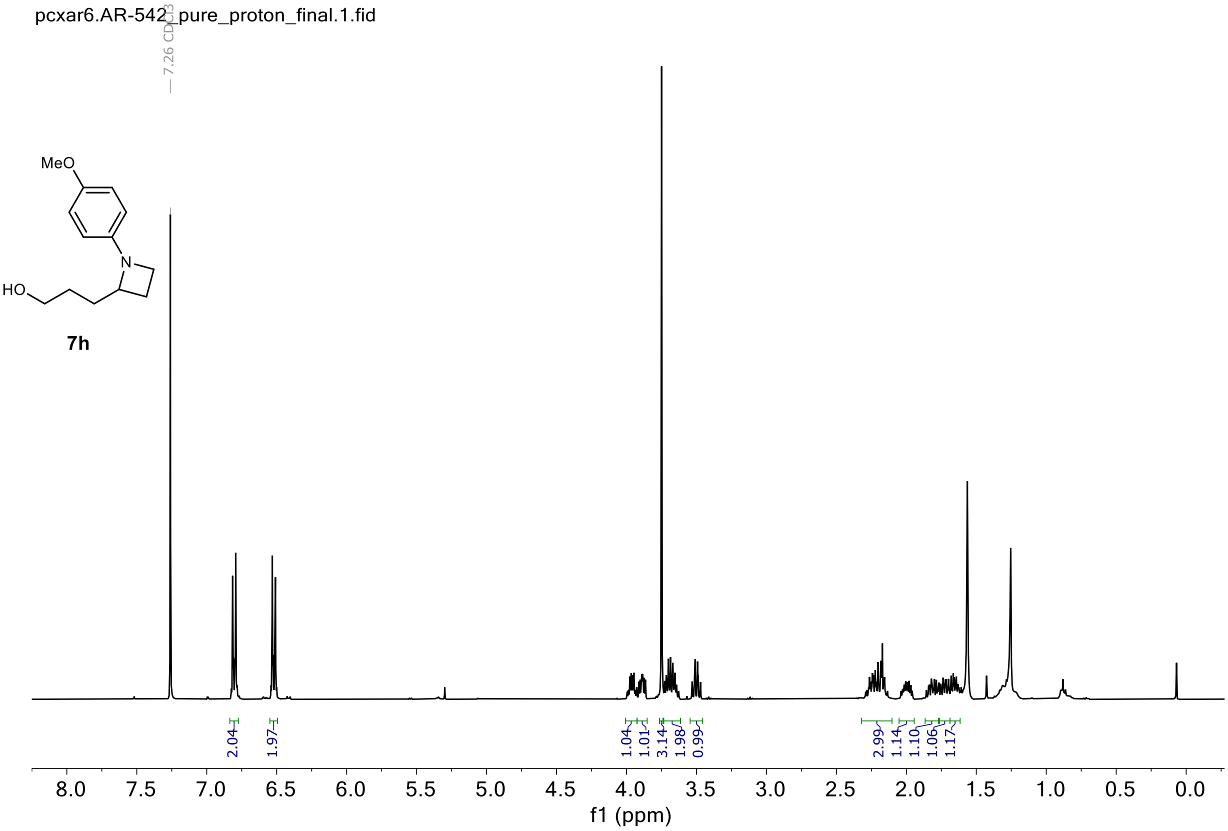

^{13}C-NMR (CDCl$_3$, 101 MHz)

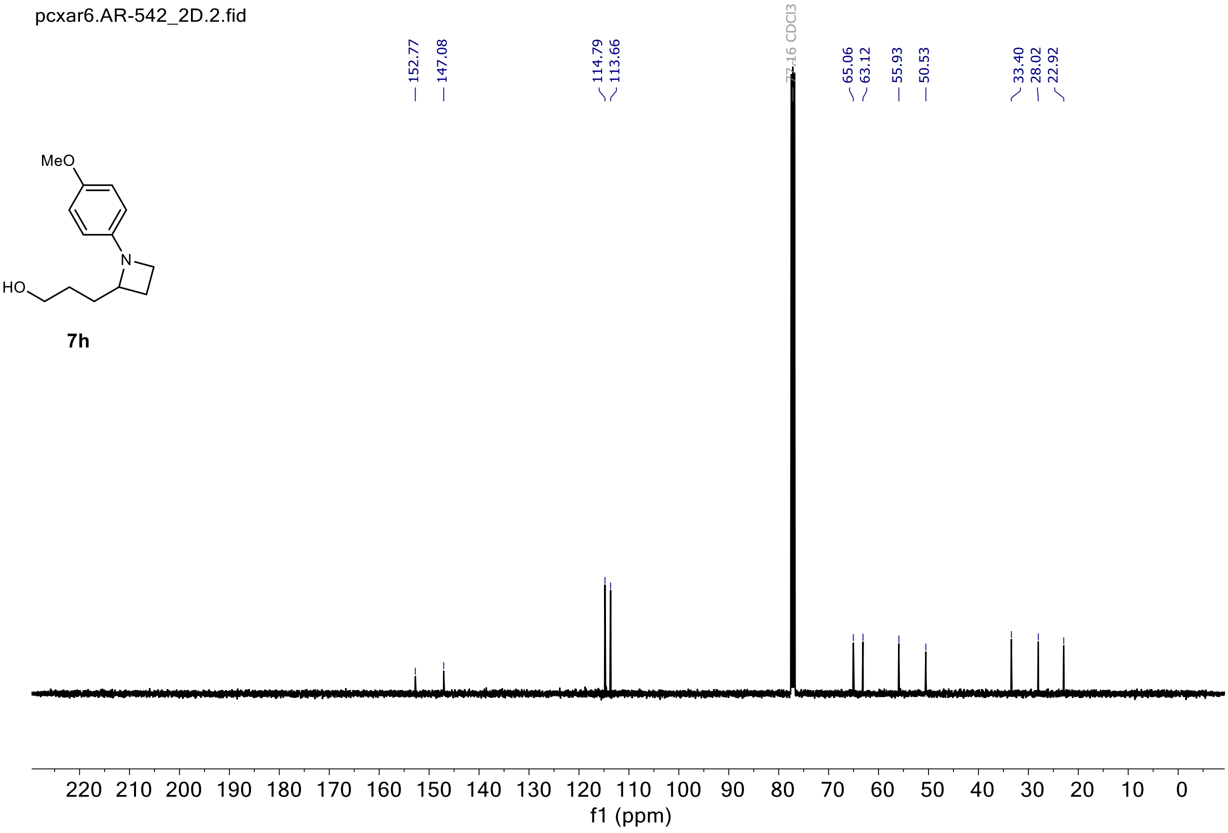

2-(1-(4-methoxyphenyl)azetidin-2-yl)ethan-1-ol (7i)

^{1}H-NMR (CDCl$_3$, 400 MHz)

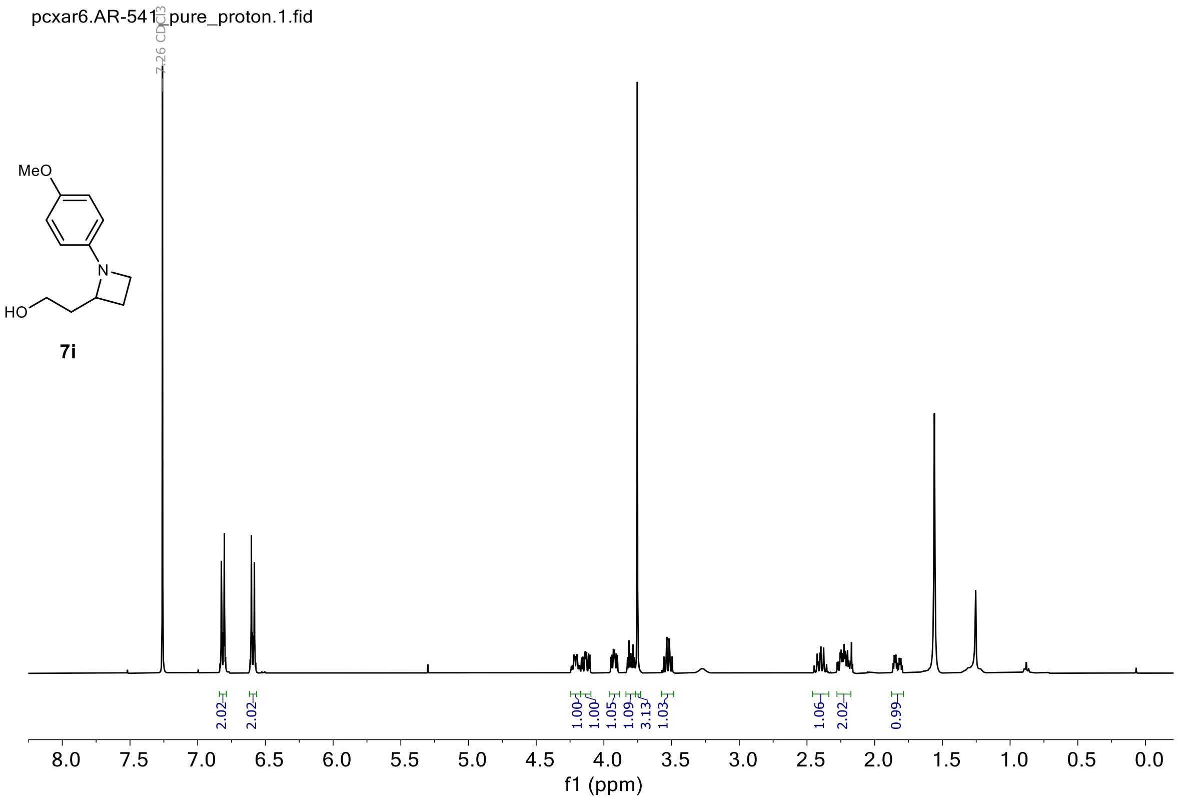

^{13}C-NMR (CDCl$_3$, 101 MHz)

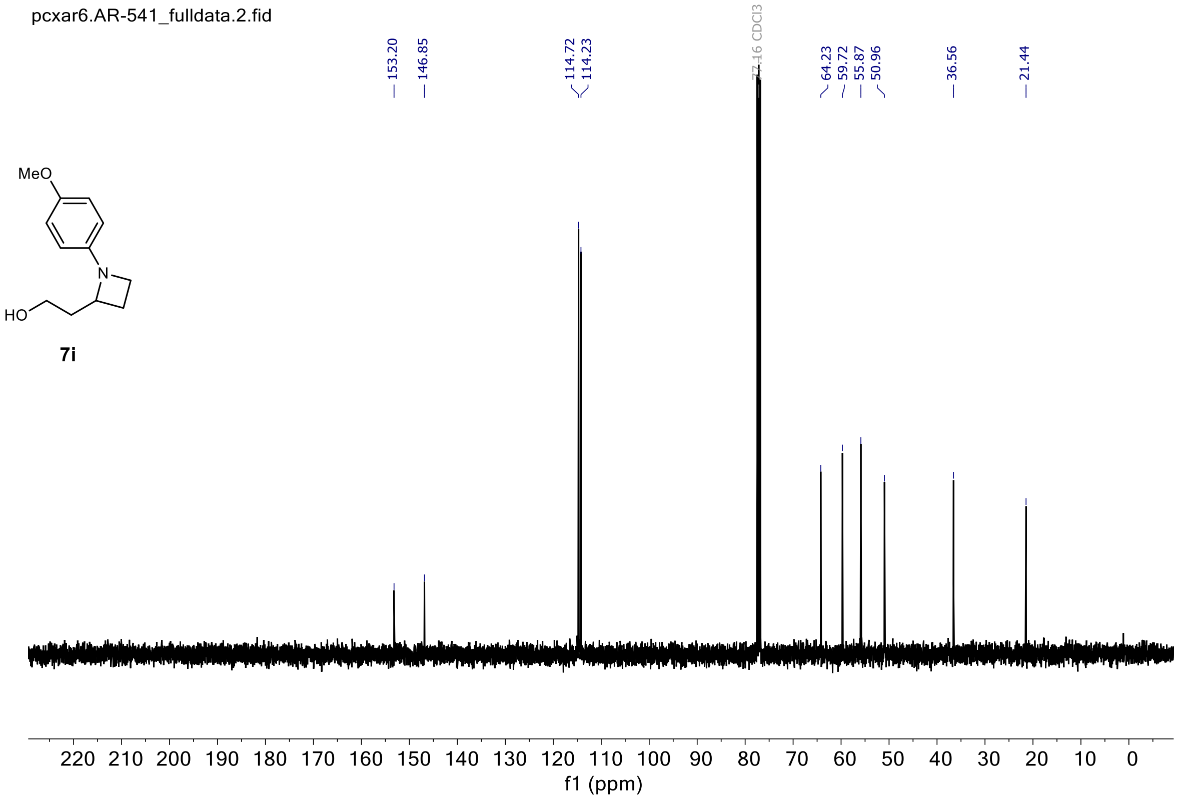

2-(3-((tert-butyldimethylsilyl)oxy)propyl)-1-(4-methoxyphenyl)azetidine (7j)

^{1}H-NMR (CDCl$_3$, 500 MHz)

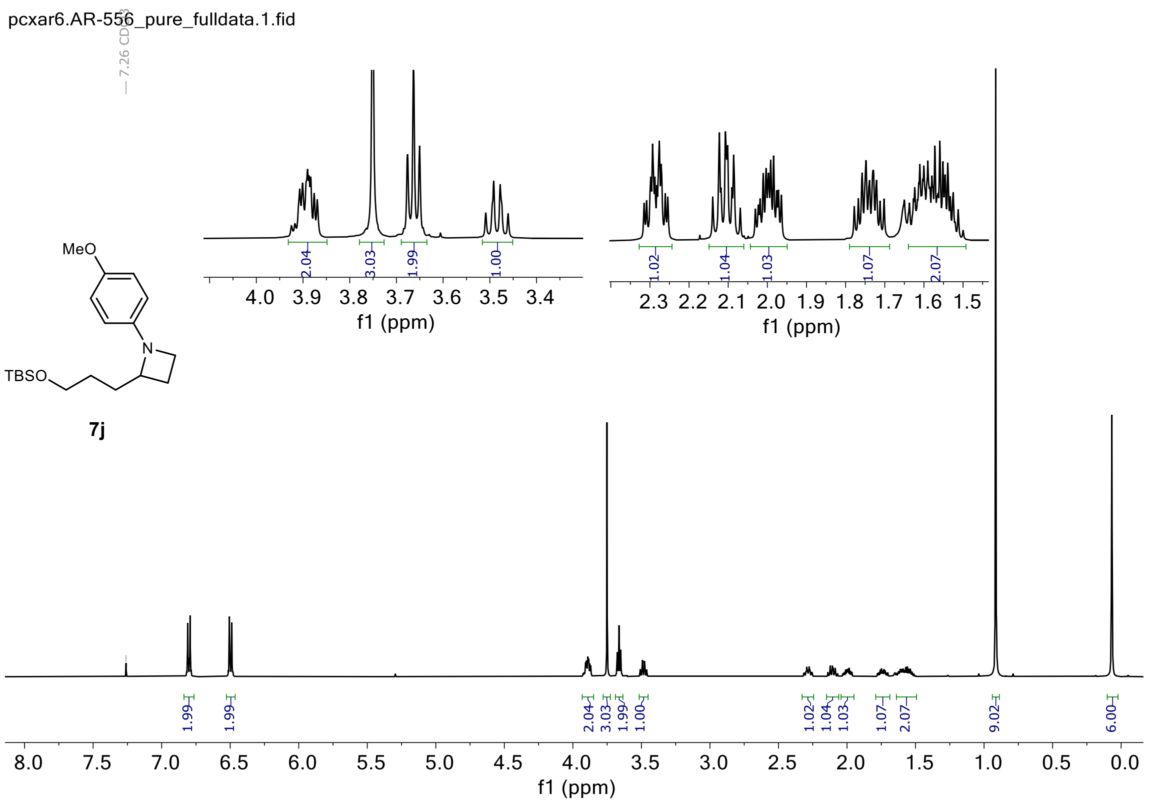

^{13}C-NMR (CDCl$_3$, 126 MHz)

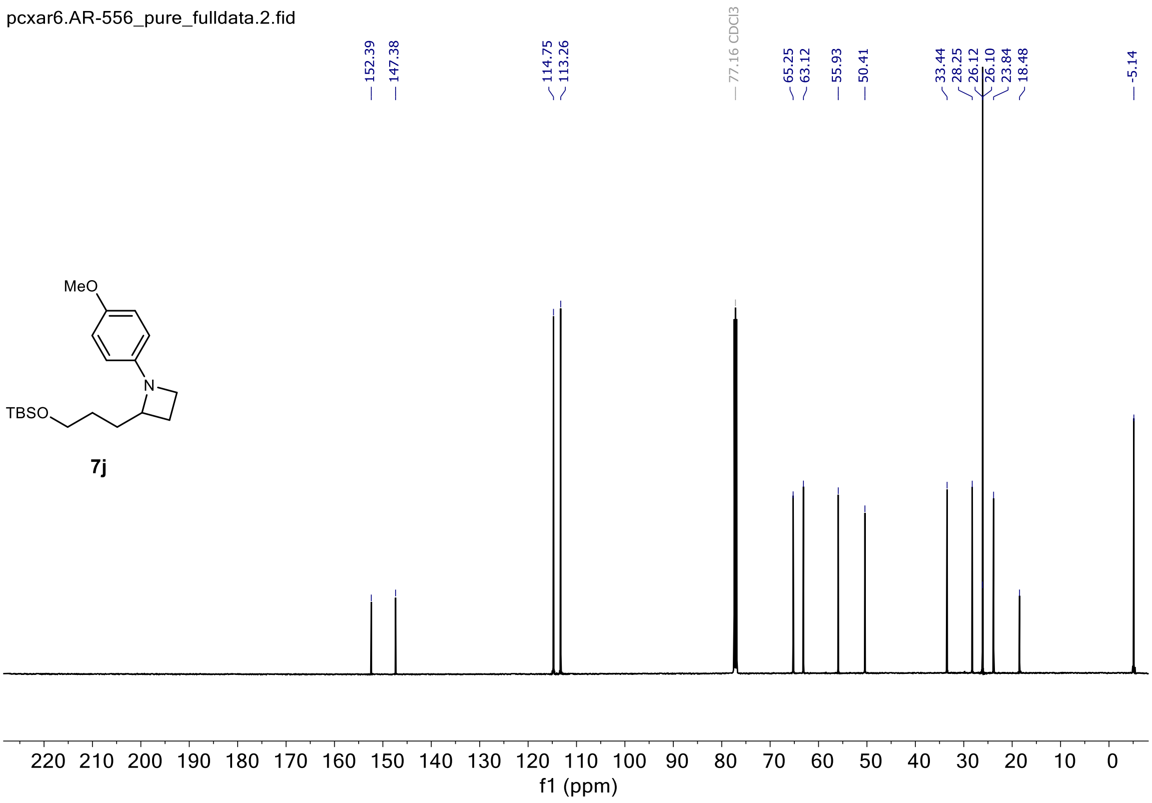

2-(2-((tert-butyldimethylsilyl)oxy)ethyl)-1-(4-methoxyphenyl)azetidine (7k)

^{1}H-NMR (CDCl$_3$, 400 MHz)

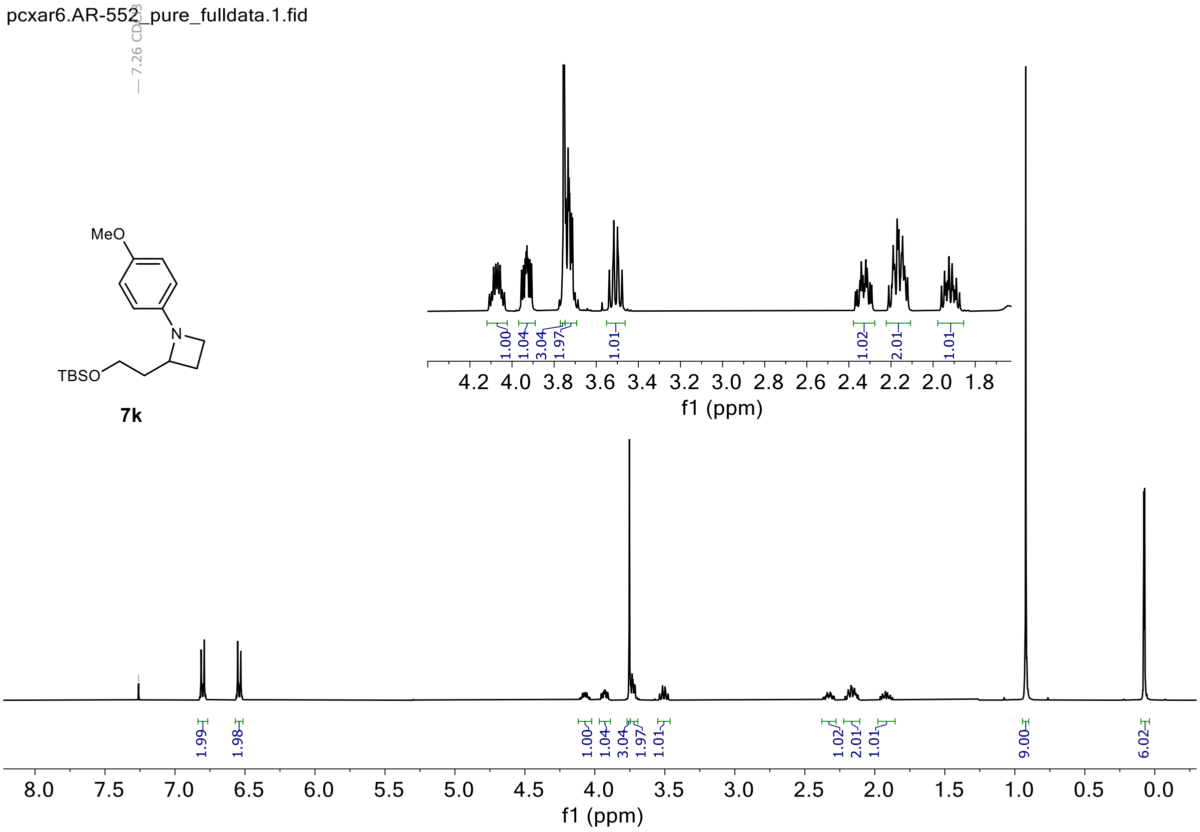

^{13}C-NMR (CDCl$_3$, 101 MHz)

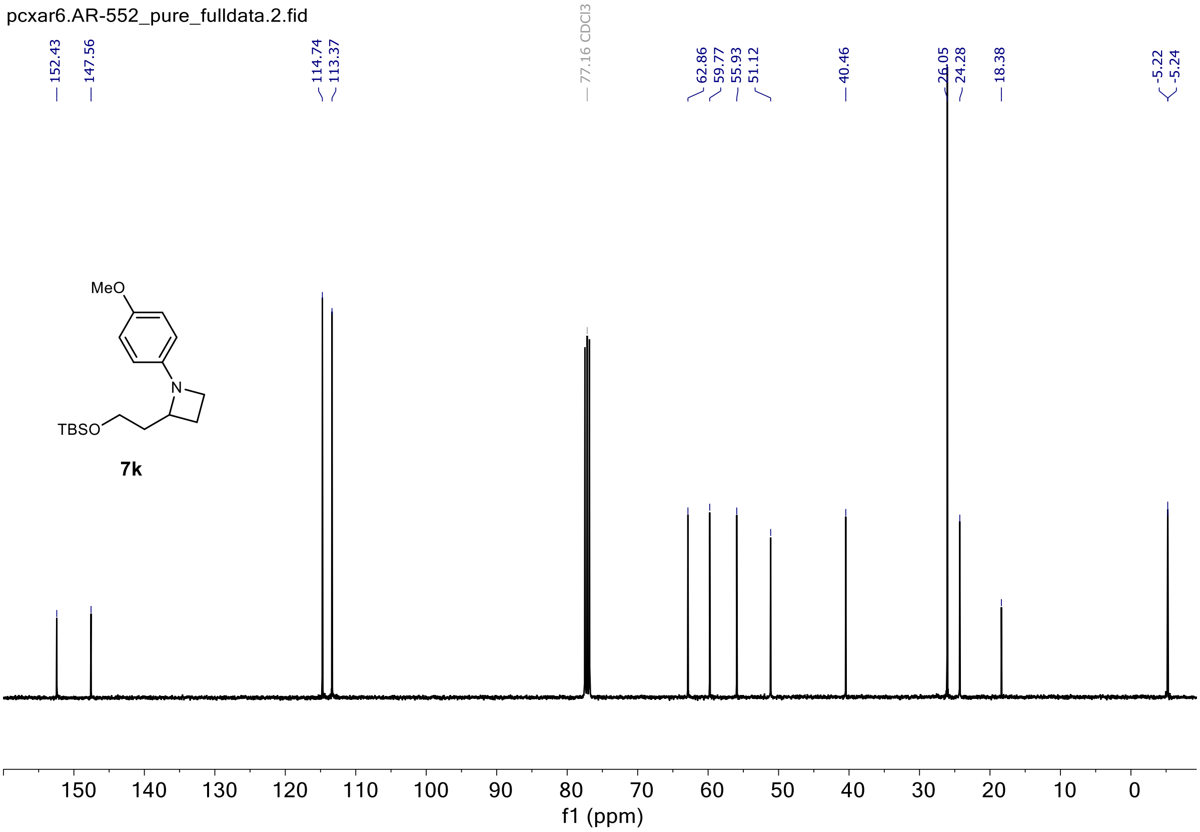

2-(9-chlorononyl)-1-(4-methoxyphenyl)azetidine (7l)

^{1}H-NMR ($CDCl_3$, 500 MHz)

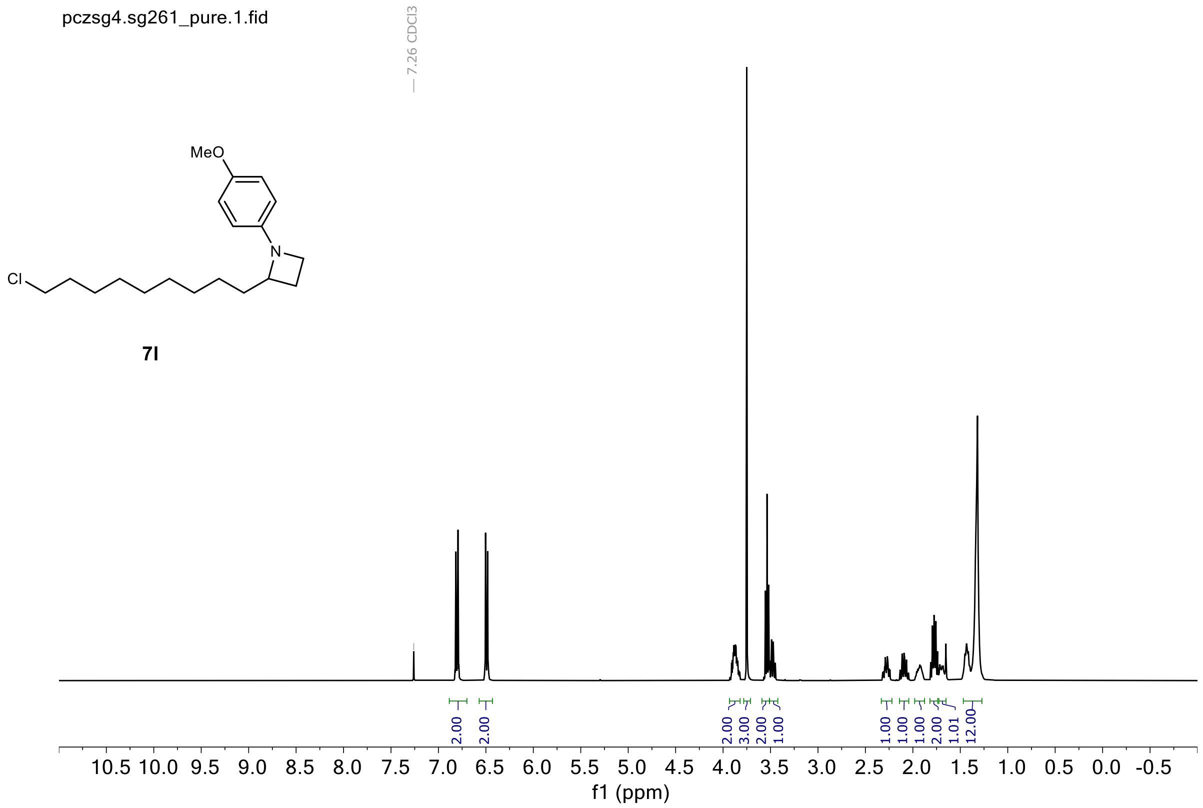

^{13}C-NMR ($CDCl_3$, 126 MHz)

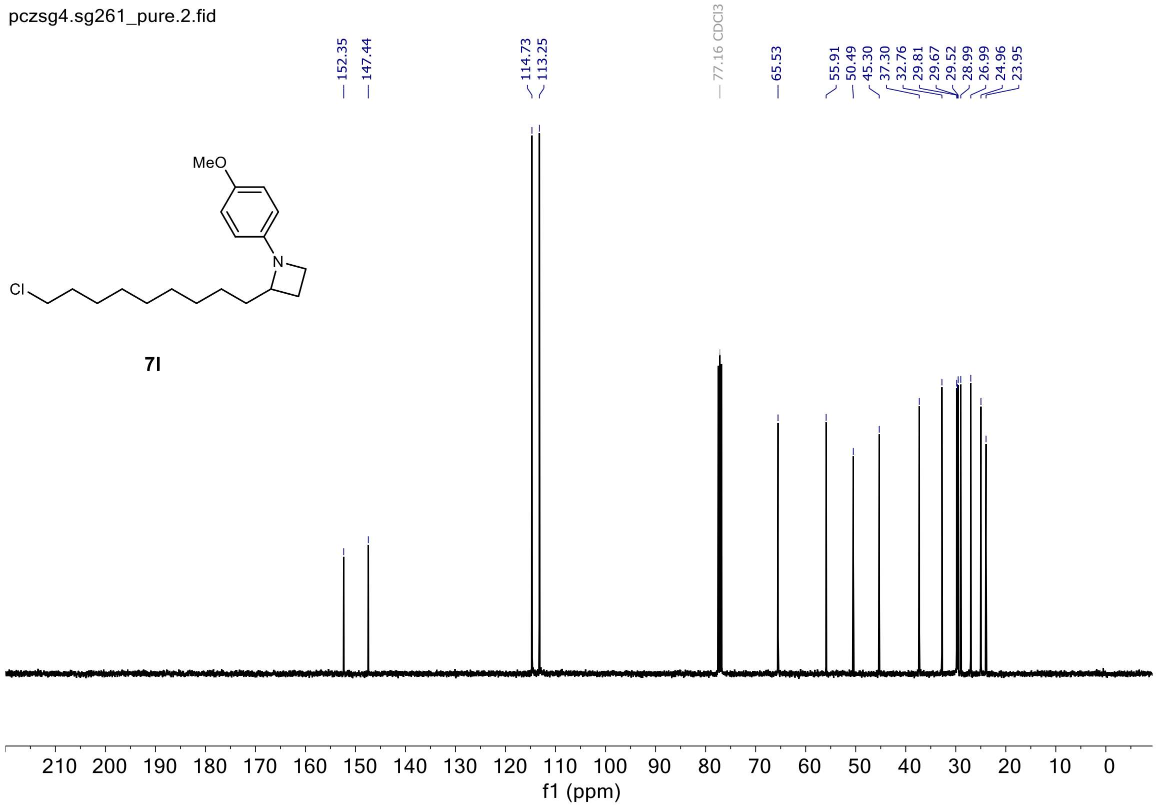

2-(4-(benzyloxy)butyl)-1-(4-methoxyphenyl)azetidine (7m)

^{1}H-NMR ($CDCl_3$, 500 MHz)

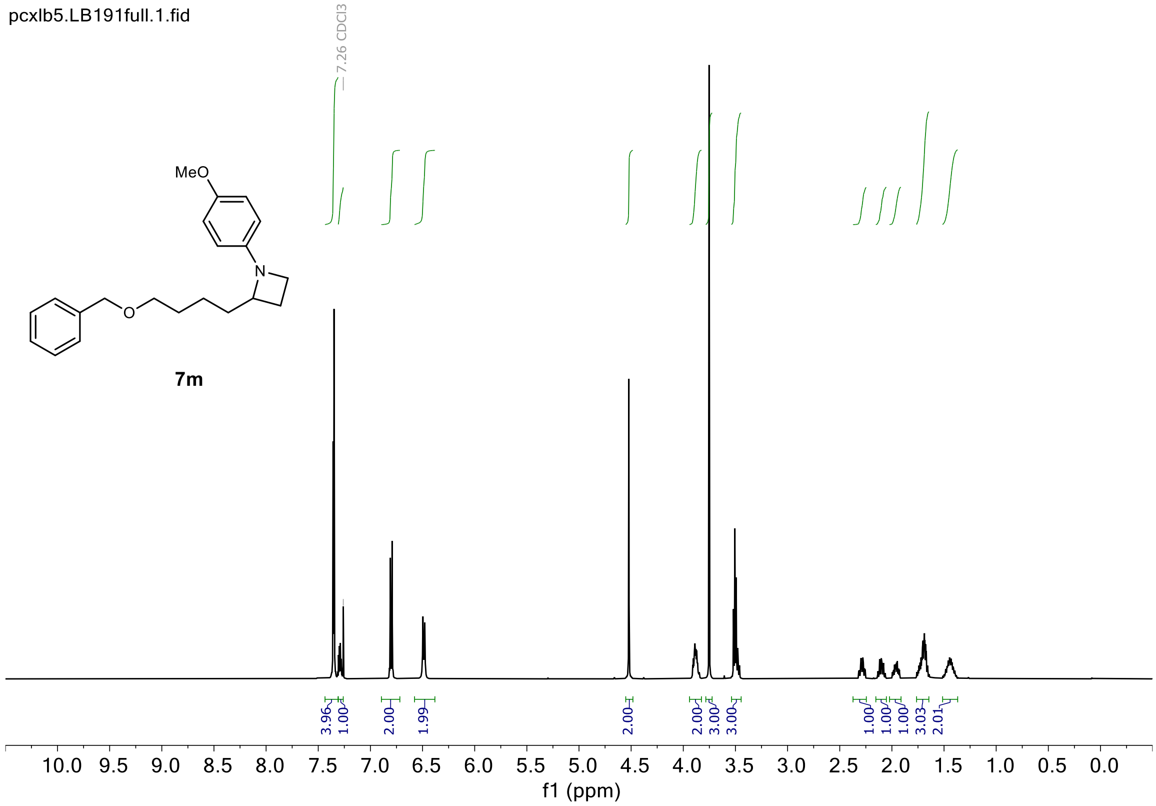

^{13}C-NMR ($CDCl_3$, 126 MHz)

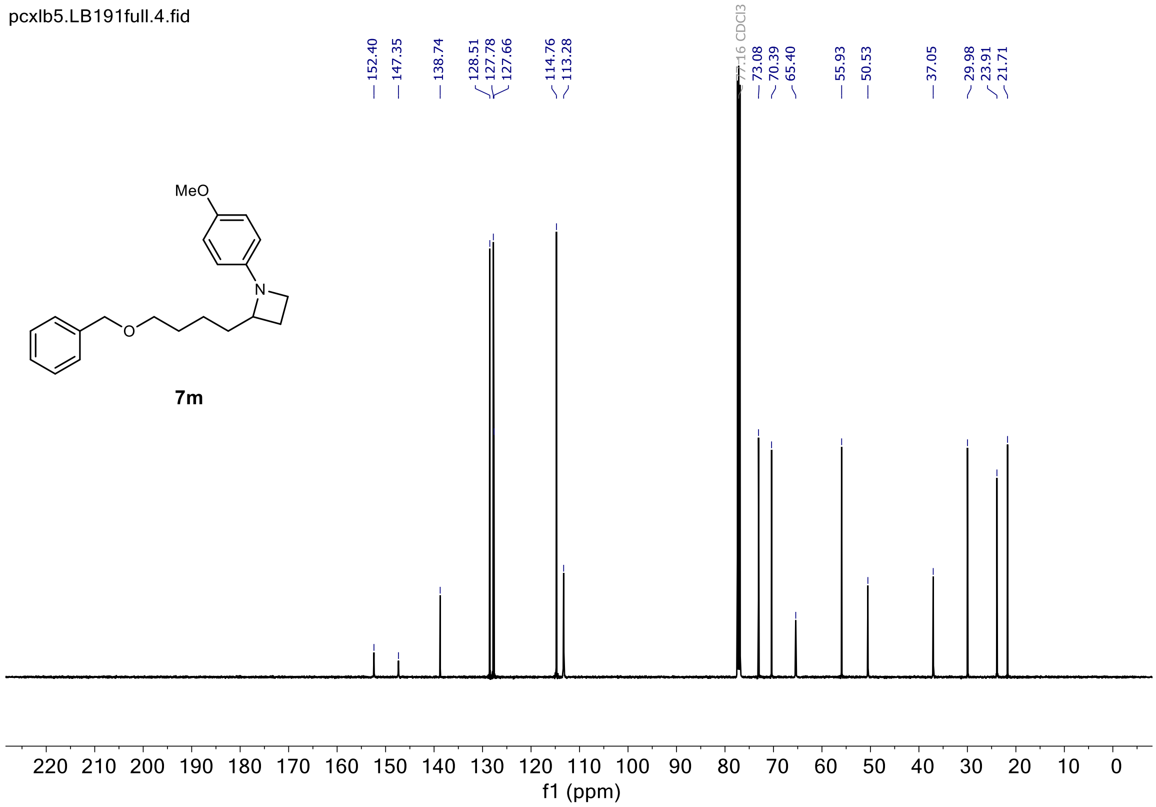

1-(4-methoxyphenyl)-2-(4-(phenylthio)butyl)azetidine (7n)

^{1}H-NMR (CDCl$_3$, 500 MHz)

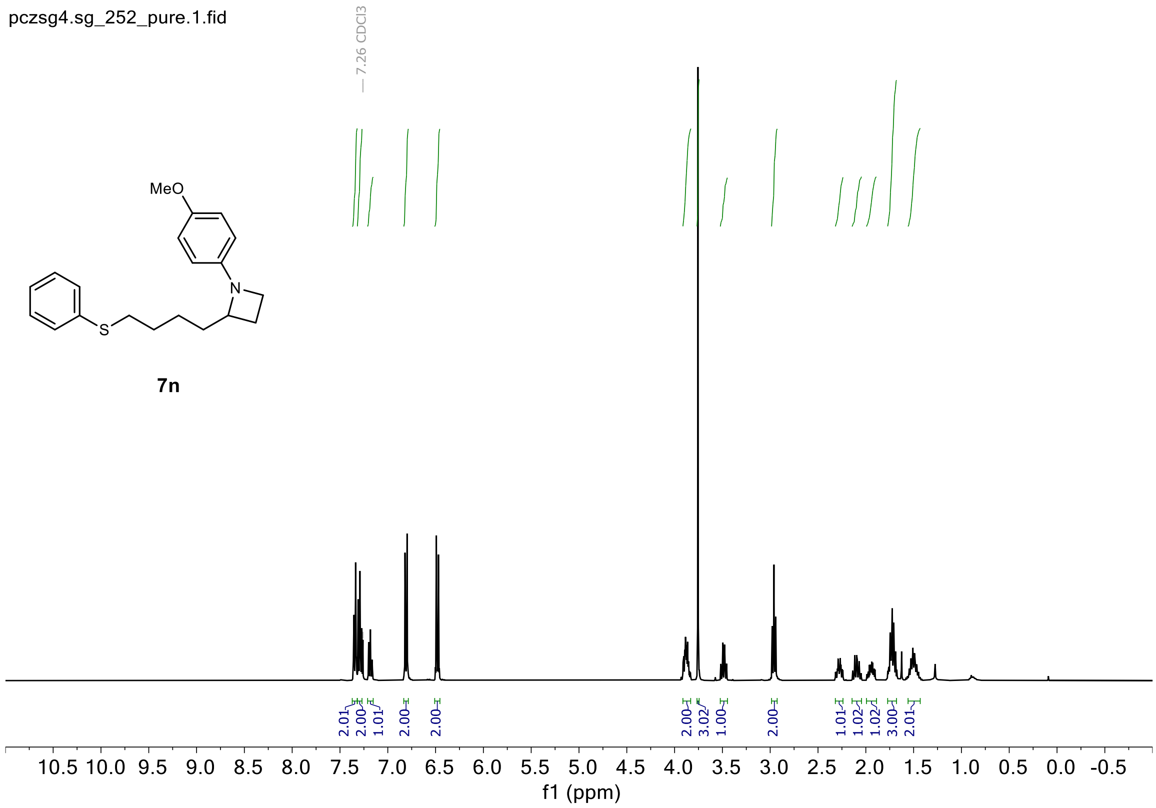

^{13}C-NMR (CDCl$_3$, 126 MHz)

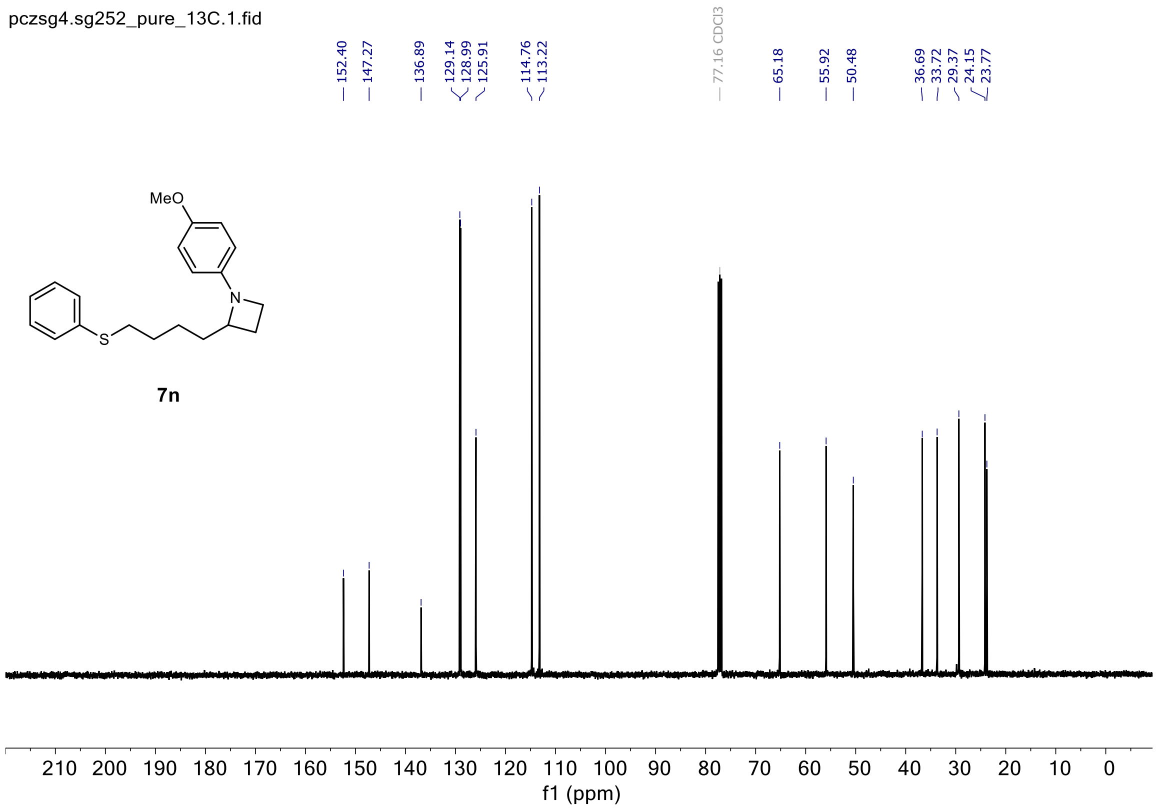

1-(4-methoxyphenyl)-2-(4-(phenylsulfonyl)butyl)azetidine (7o)

^{1}H-NMR ($CDCl_3$, 500 MHz)

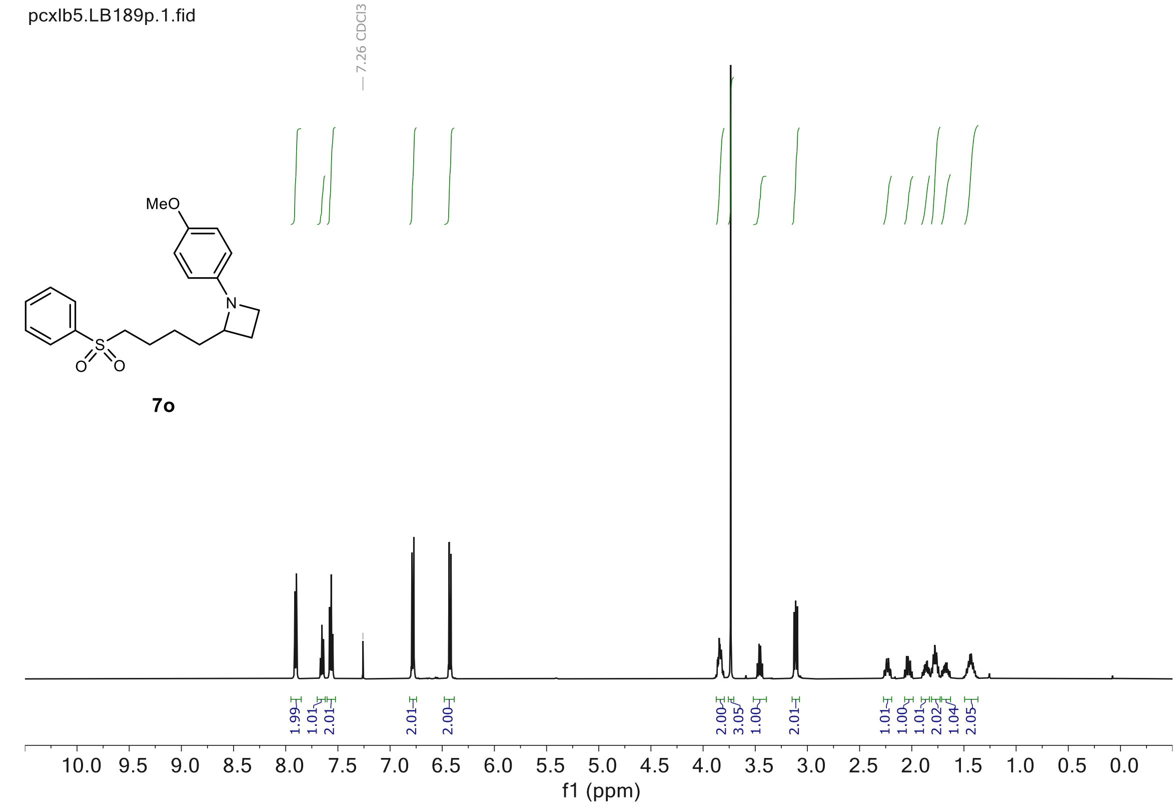

^{13}C-NMR ($CDCl_3$, 126 MHz)

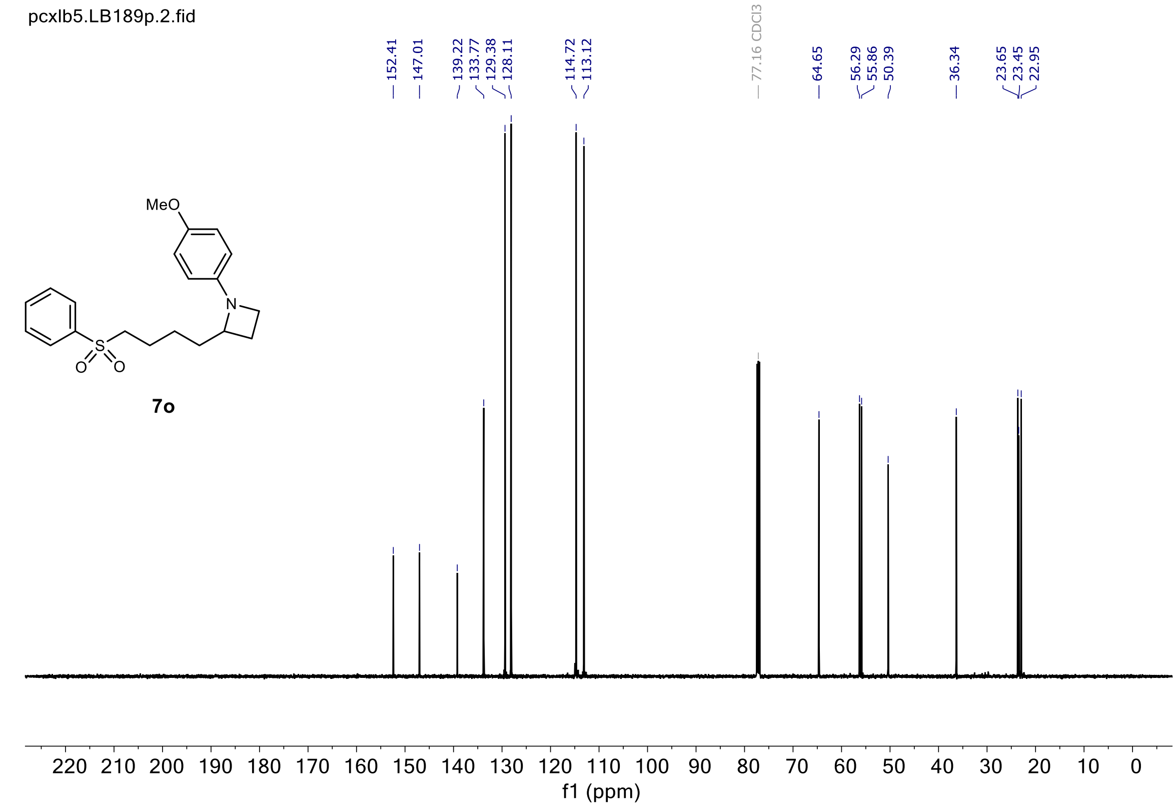

diethyl (4-(1-(4-methoxyphenyl)azetidin-2-yl)butyl)phosphonate (7p)

^{1}H-NMR ($CDCl_3$, 500 MHz)

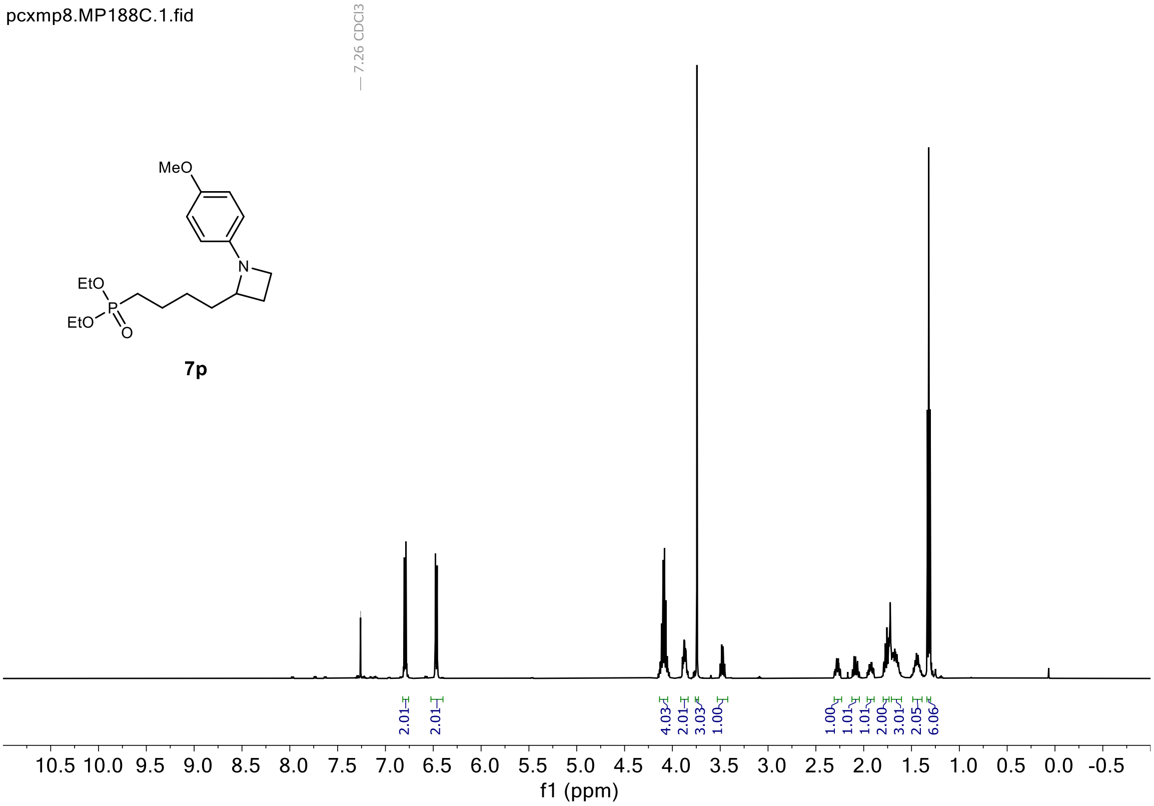

^{13}C-NMR ($CDCl_3$, 126 MHz)

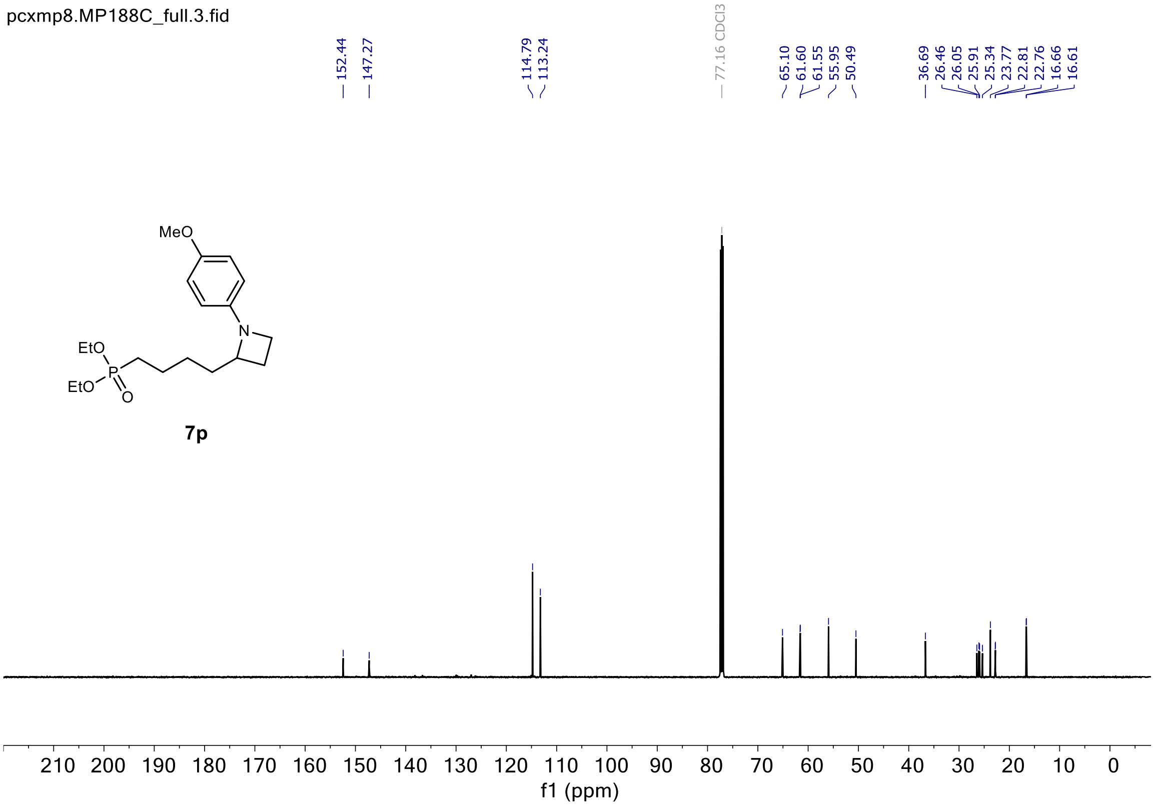

^{31}P-NMR ($CDCl_3$, 202 MHz)

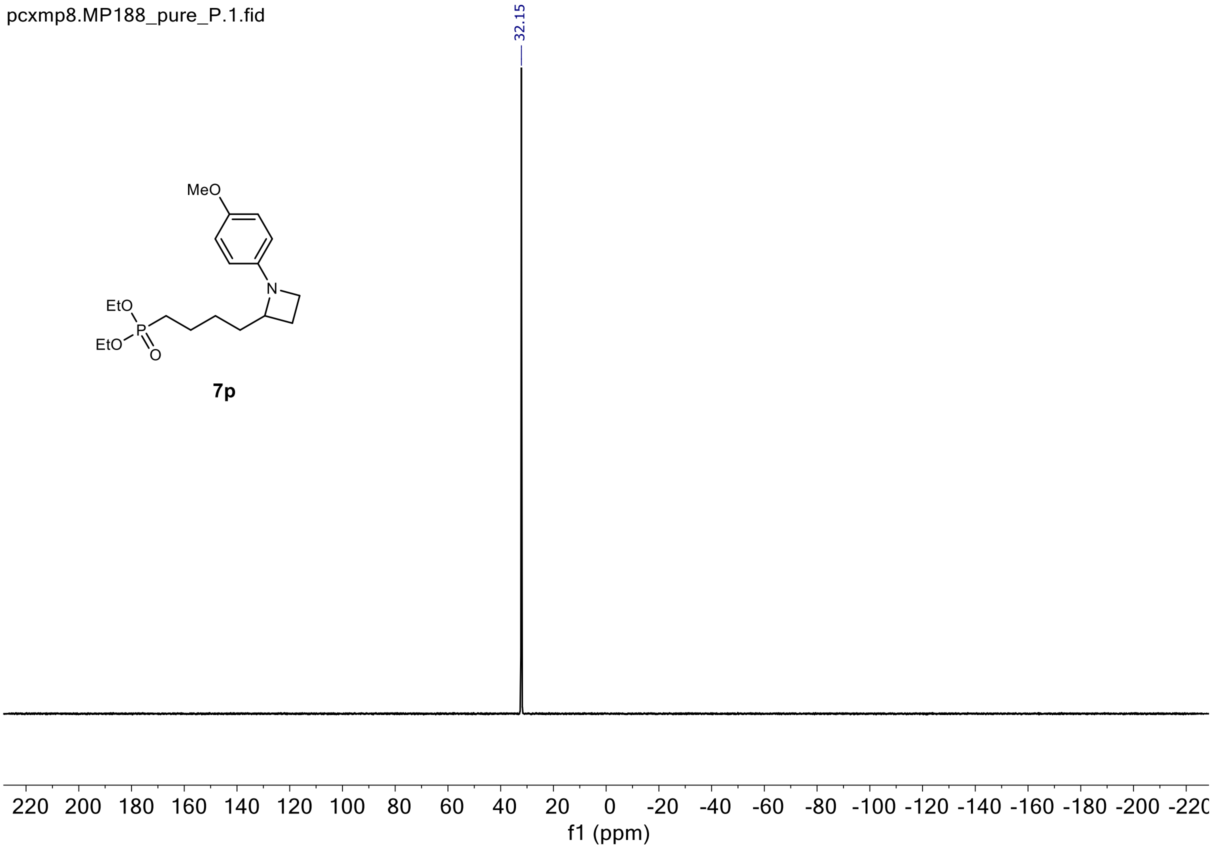

1-(4-methoxyphenyl)-2-(9-(4,4,5,5-tetramethyl-1,3,2-dioxaborolan-2-yl)nonyl)azetidine (7q)

^{1}H-NMR (CDCl$_3$, 500 MHz)

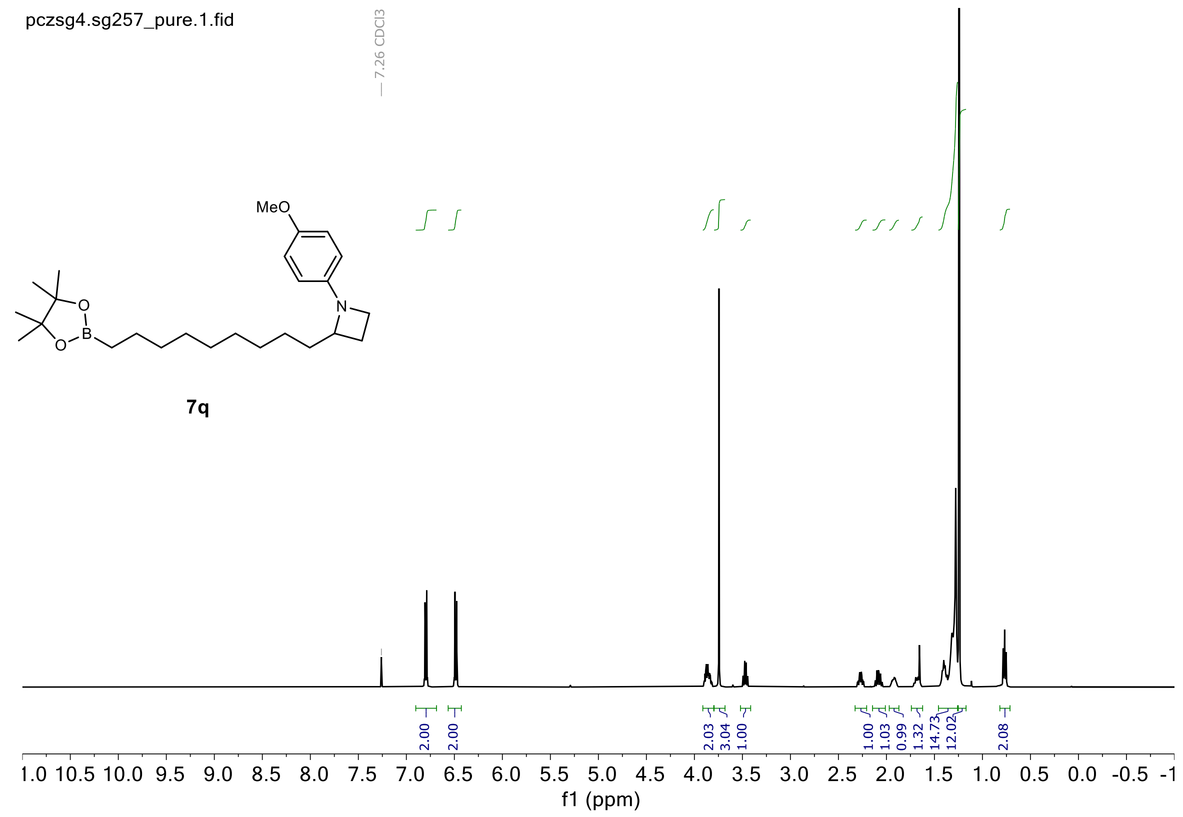

^{13}C-NMR (CDCl$_3$, 126 MHz)

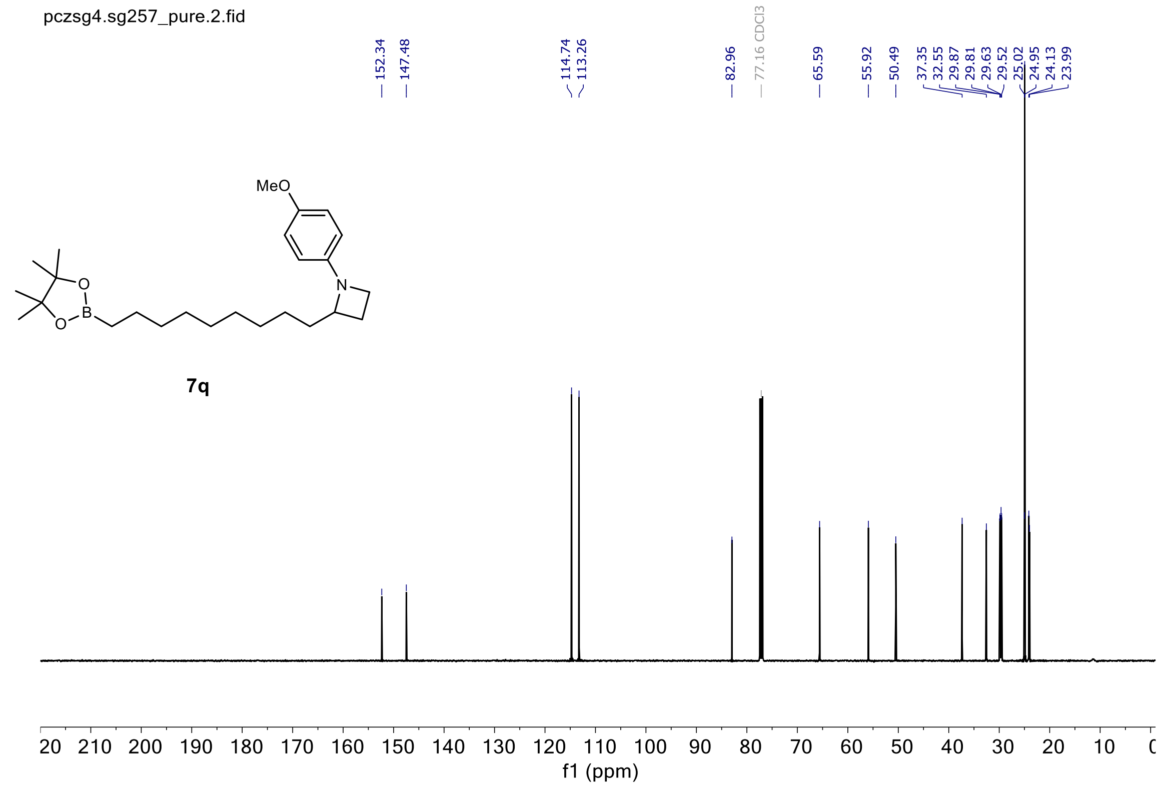

^{11}B-NMR ($CDCl_3$, 128 MHz)

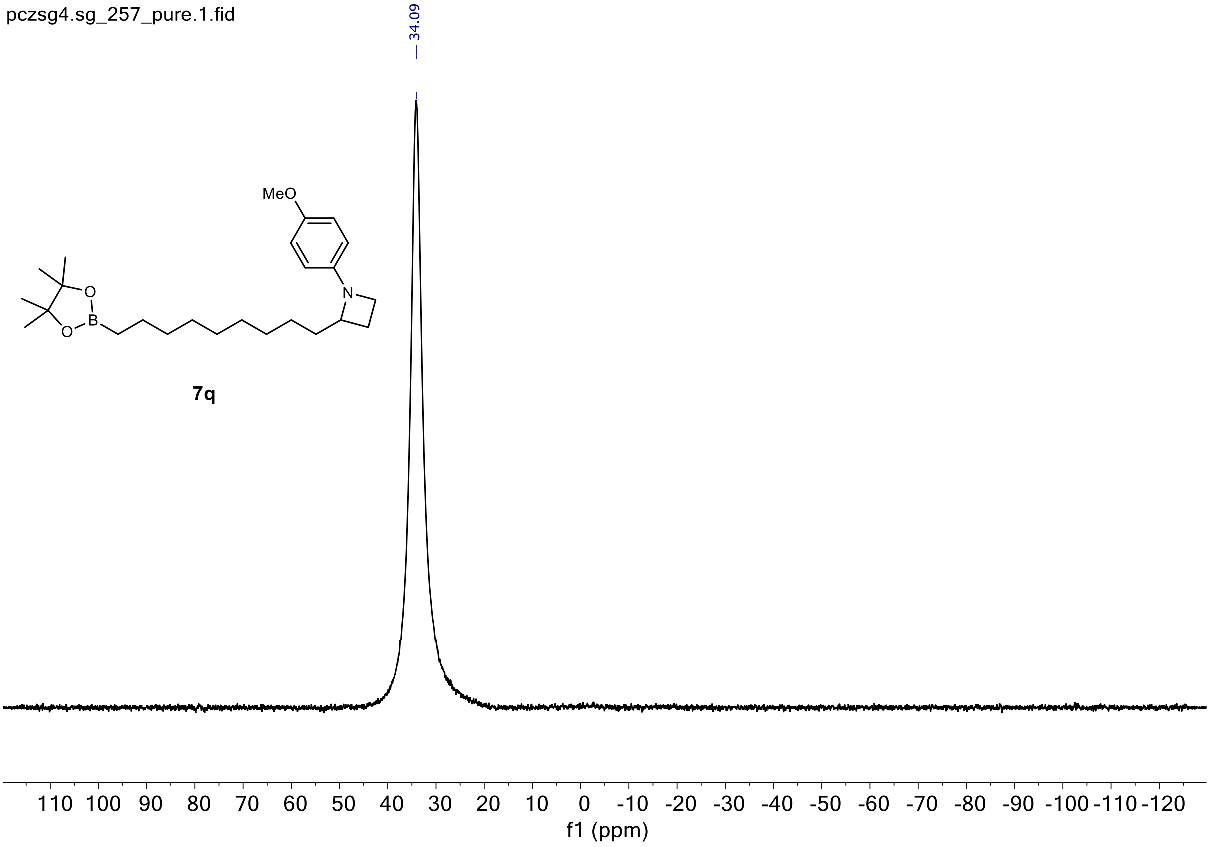

7q

1-(4-methoxyphenyl)-2-(undec-10-yn-1-yl)azetidine (7r)

^{1}H-NMR (CDCl$_3$, 500 MHz)

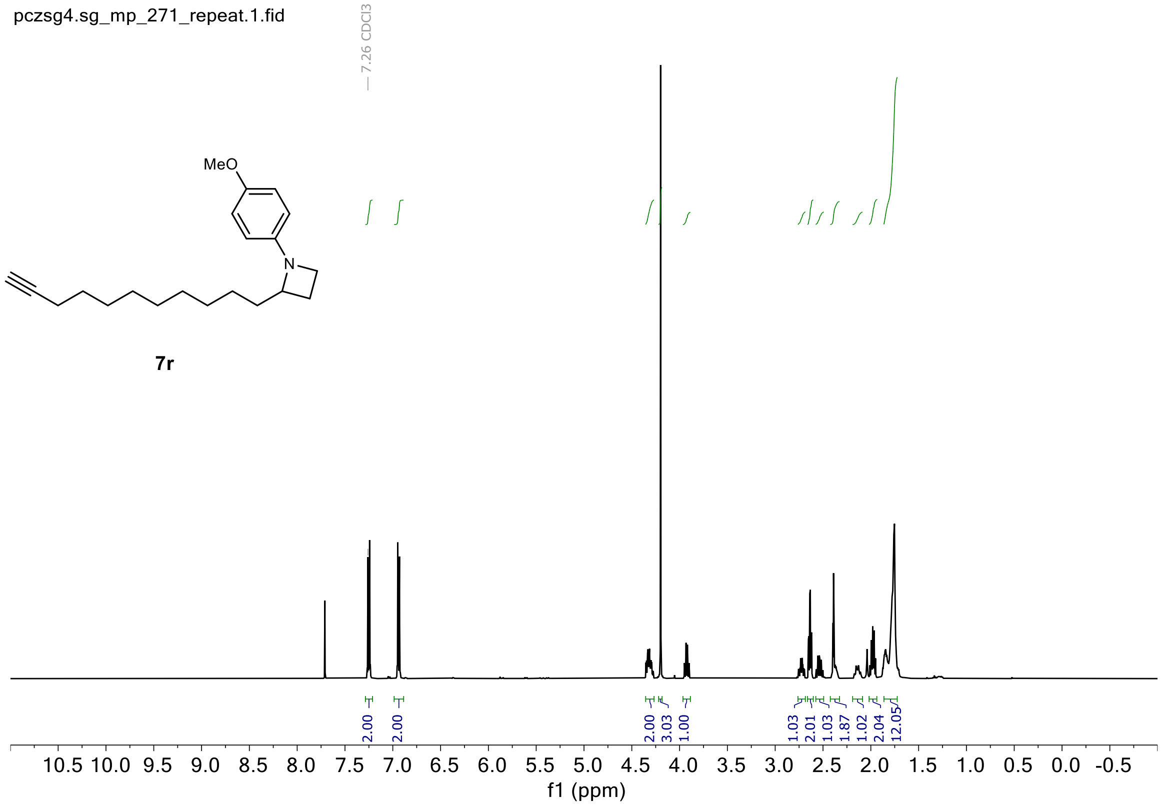

^{13}C-NMR (CDCl$_3$, 126 MHz)

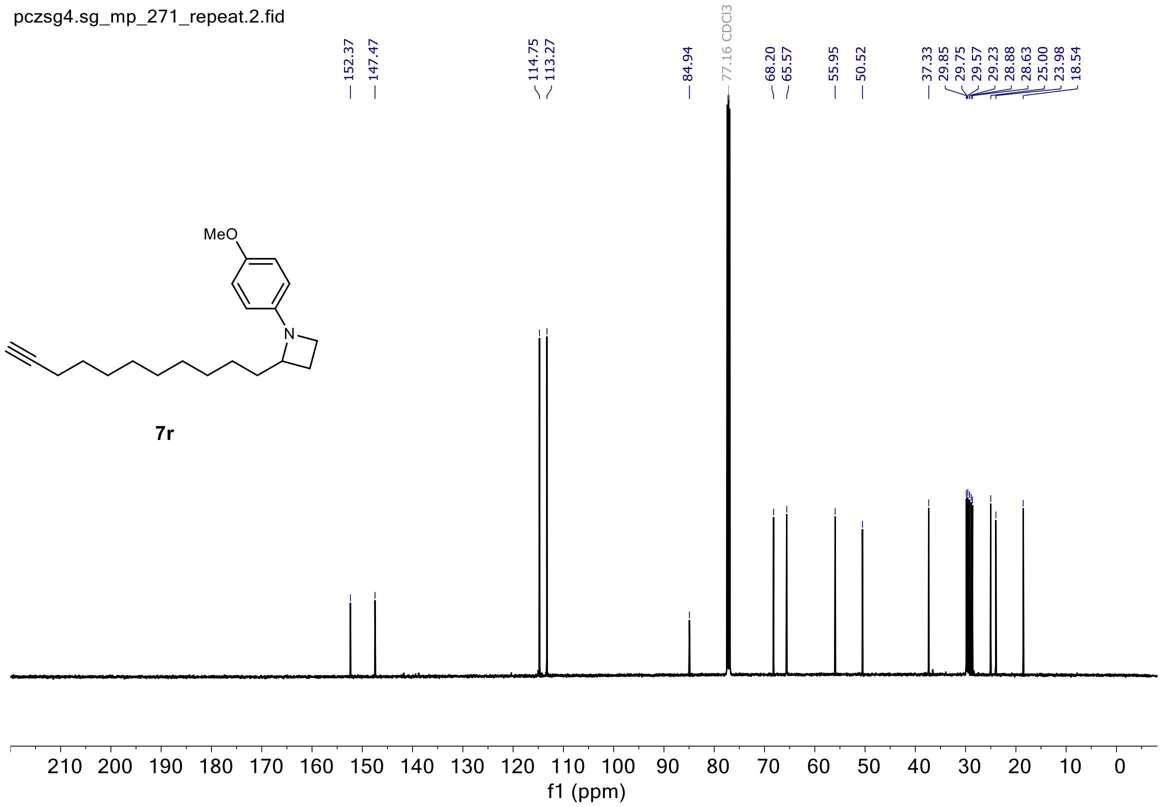

ethyl (E)-10-(1-(4-methoxyphenyl)azetidin-2-yl)dec-2-enoate (7s)

^{1}H-NMR (CDCl$_3$, 500 MHz)

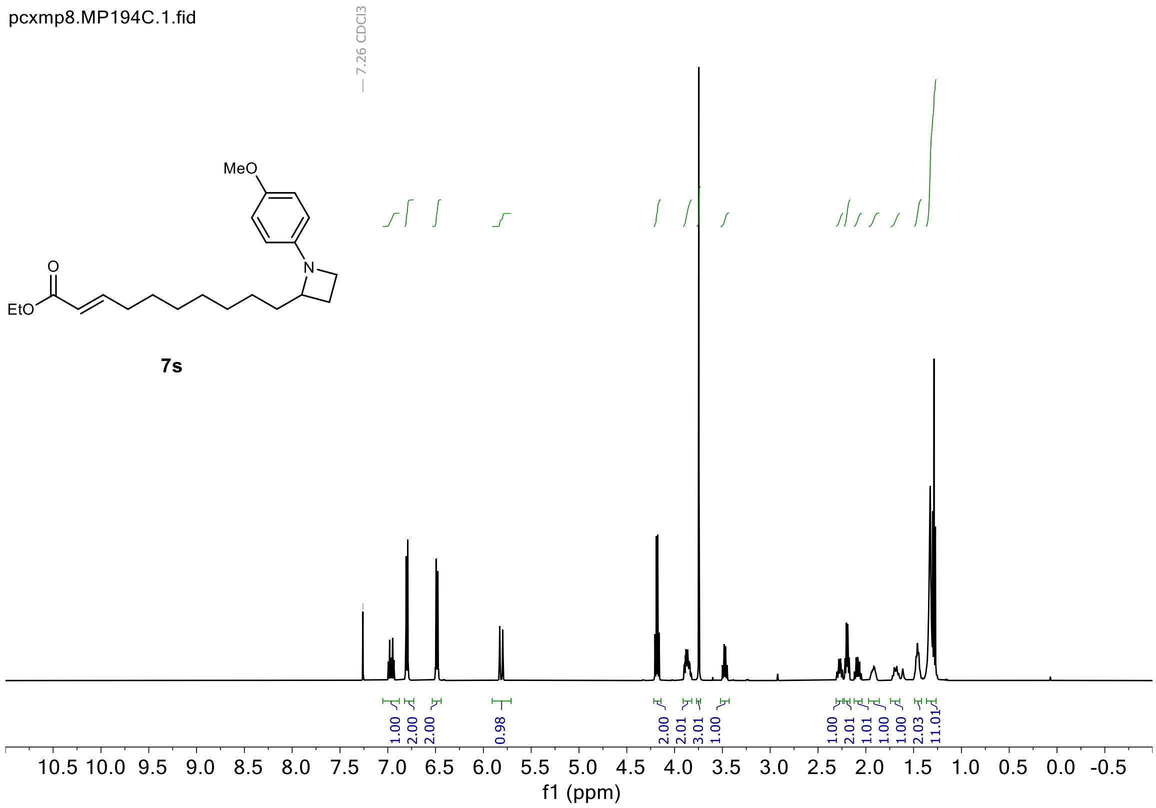

^{13}C-NMR (CDCl$_3$, 126 MHz)

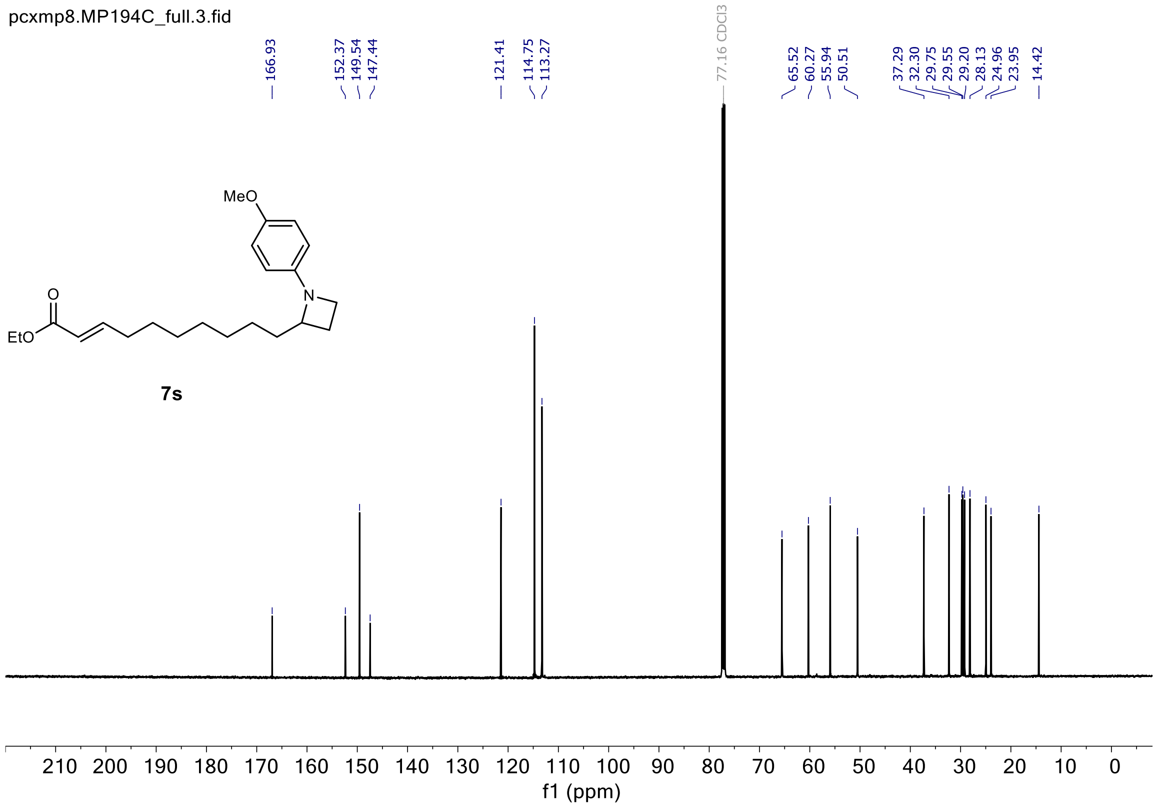

3-(4-(1-(4-methoxyphenyl)azetidin-2-yl)butyl)pyridine (7t)

^{1}H-NMR (CDCl$_3$, 500 MHz)

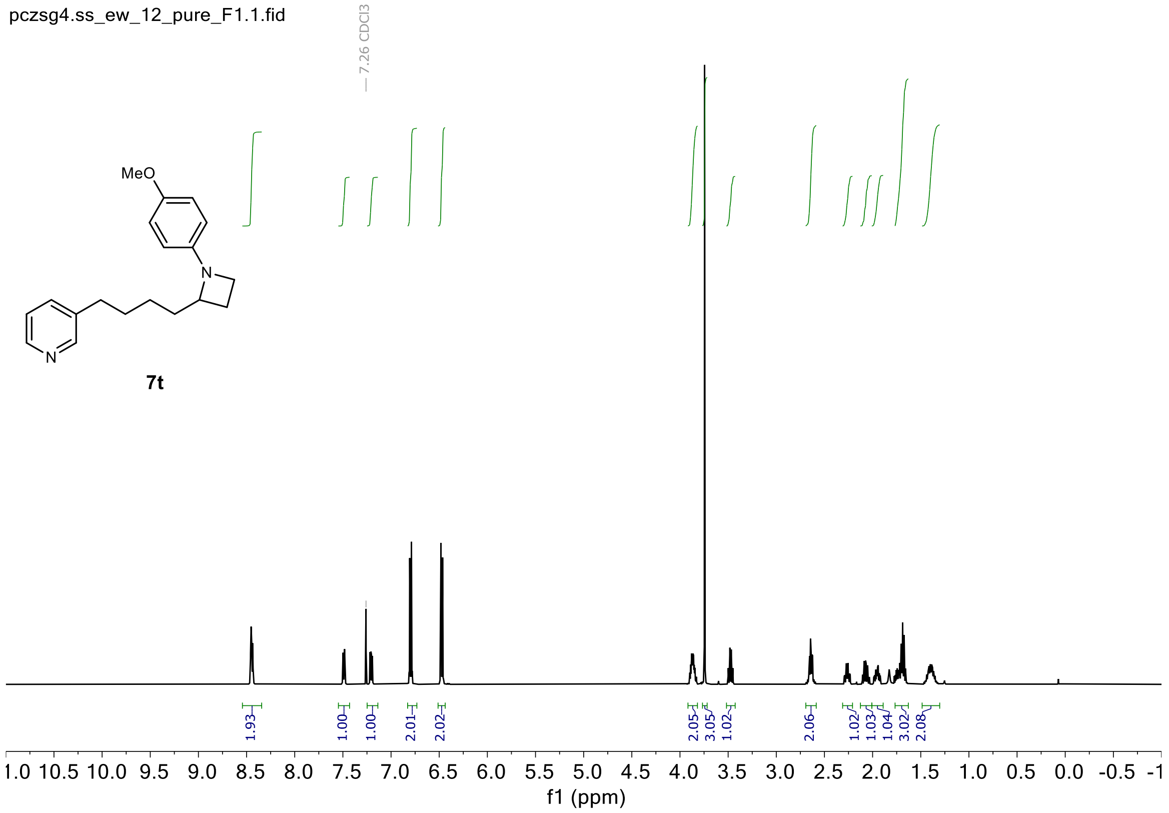

^{13}C-NMR (CDCl$_3$, 126 MHz)

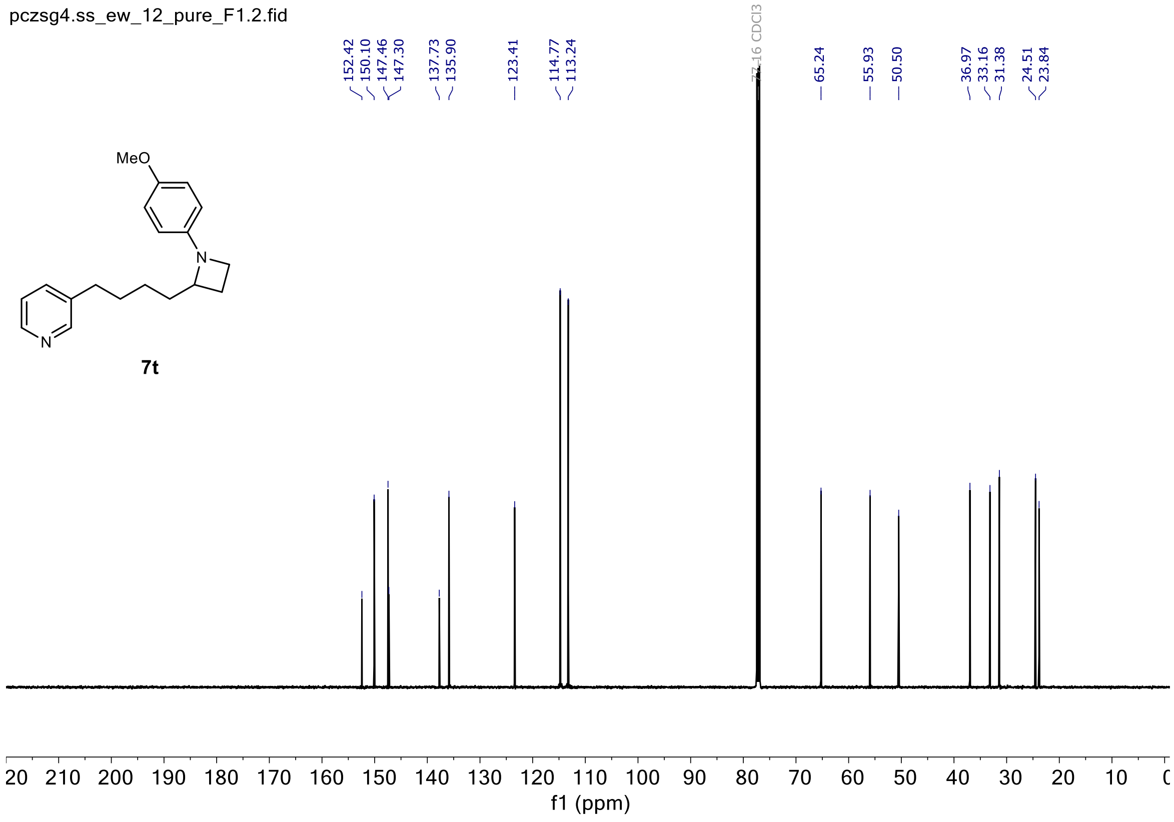

1-(4-methoxyphenyl)-2-phenylazetidine (7u)

^{1}H-NMR ($CDCl_3$, 500 MHz)

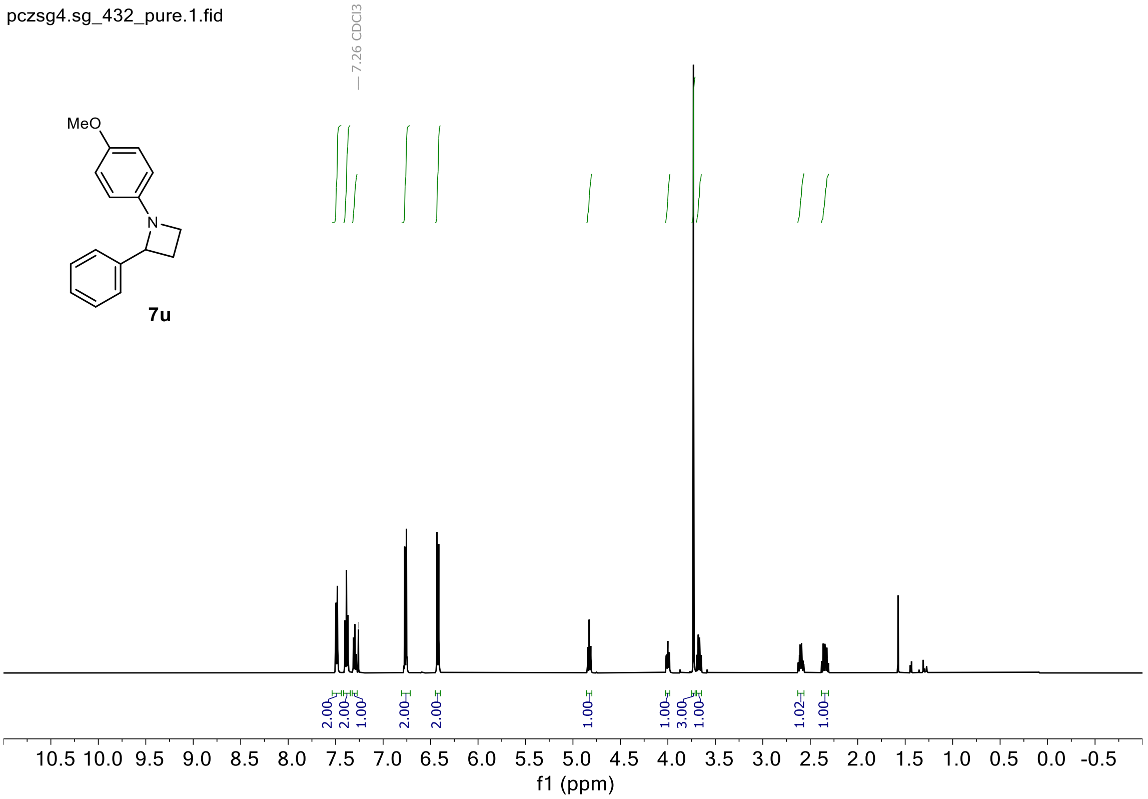

^{13}C-NMR ($CDCl_3$, 126 MHz)

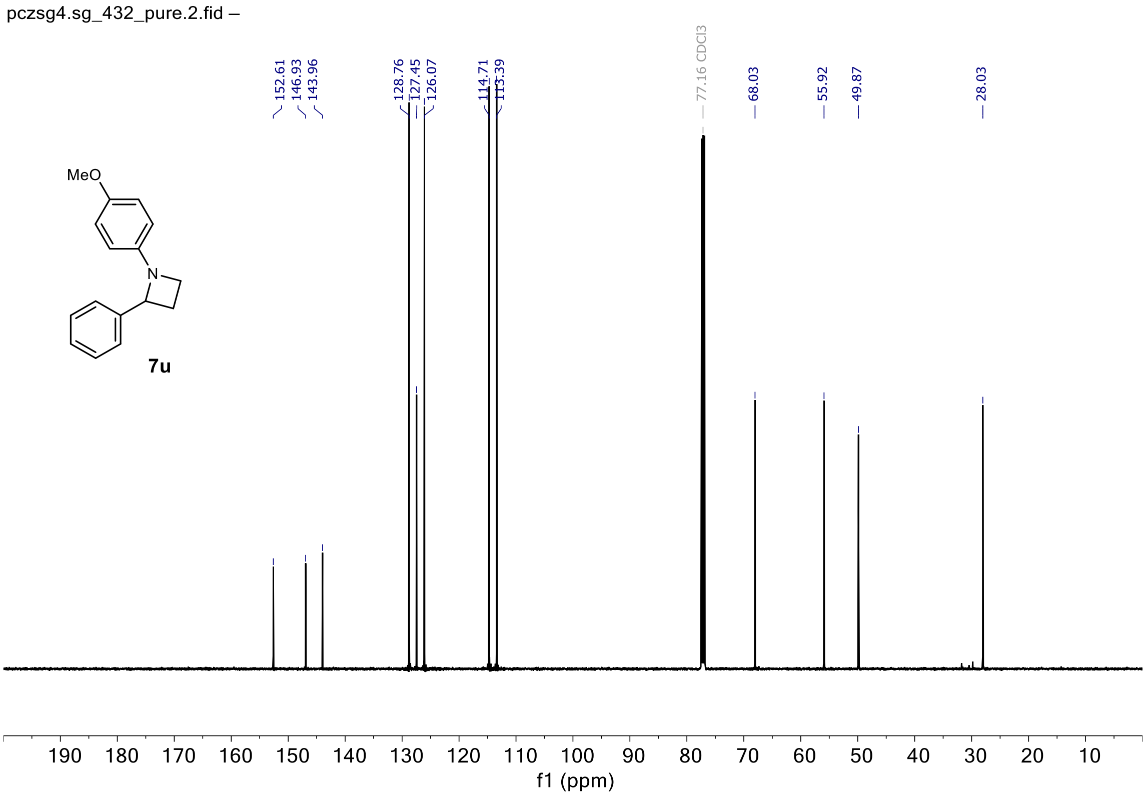

(3R,8R,9S,10S,13R,14S,17R)-17-((2R)-1-(1-(4-methoxyphenyl)azetidin-2-yl)propan-2-yl)-10,13-dimethylhexadecahydro-1H-cyclopenta[a]phenanthren-3-ol (7v)

^{1}H-NMR ($CDCl_3$, 400 MHz)

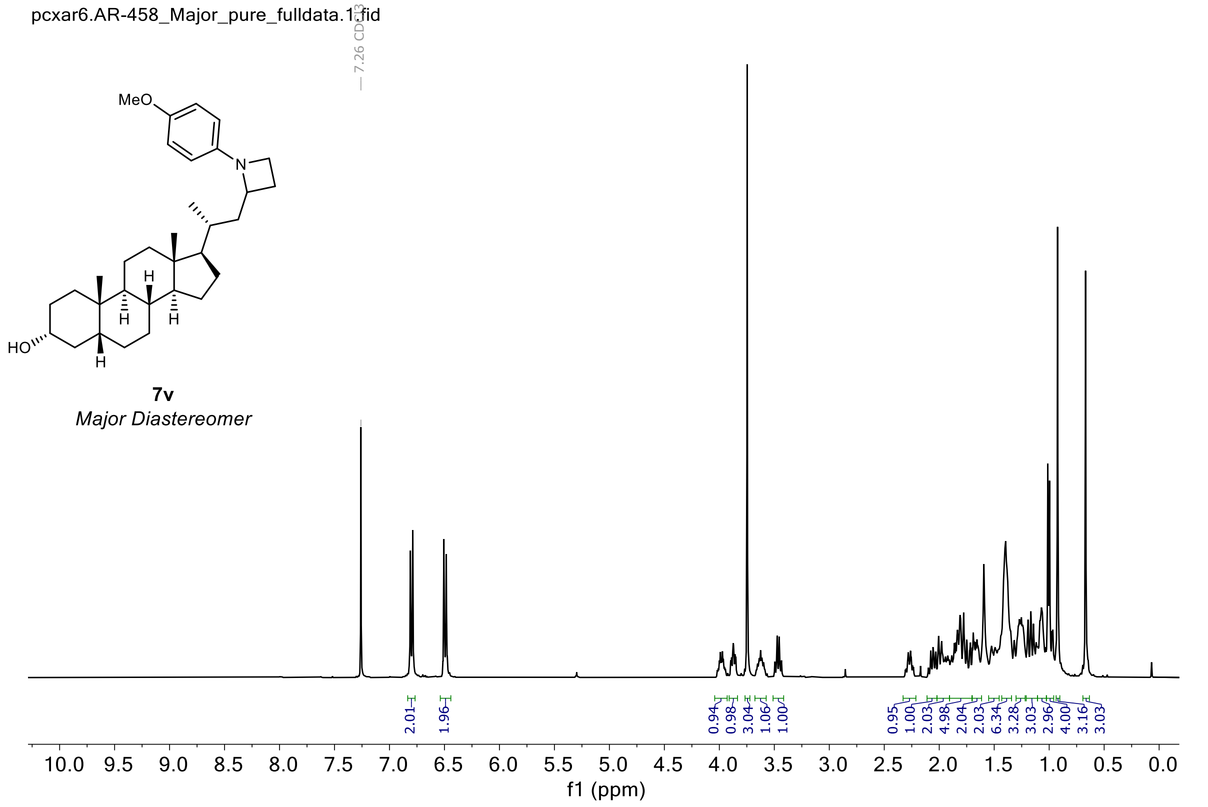

^{13}C-NMR ($CDCl_3$, 101 MHz)

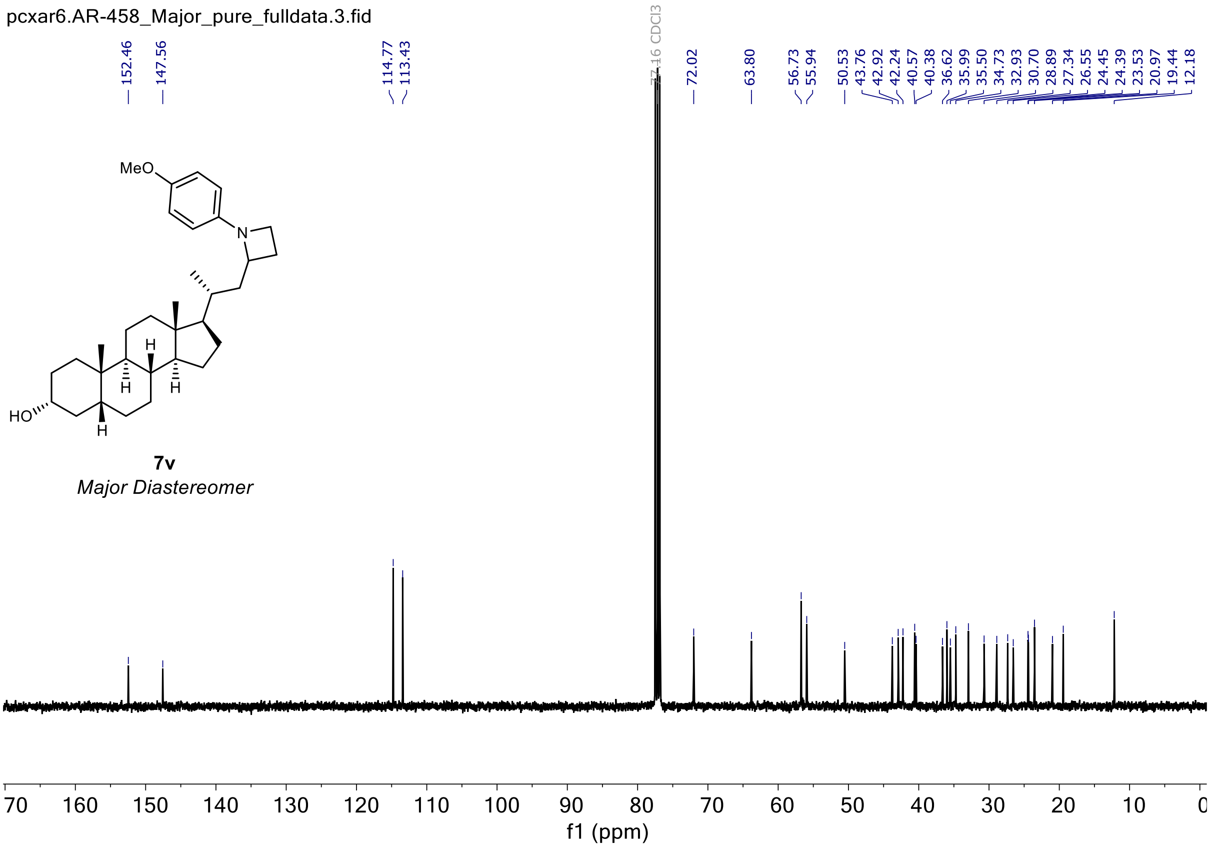

^{1}H-NMR ($CDCl_3$, 400 MHz)

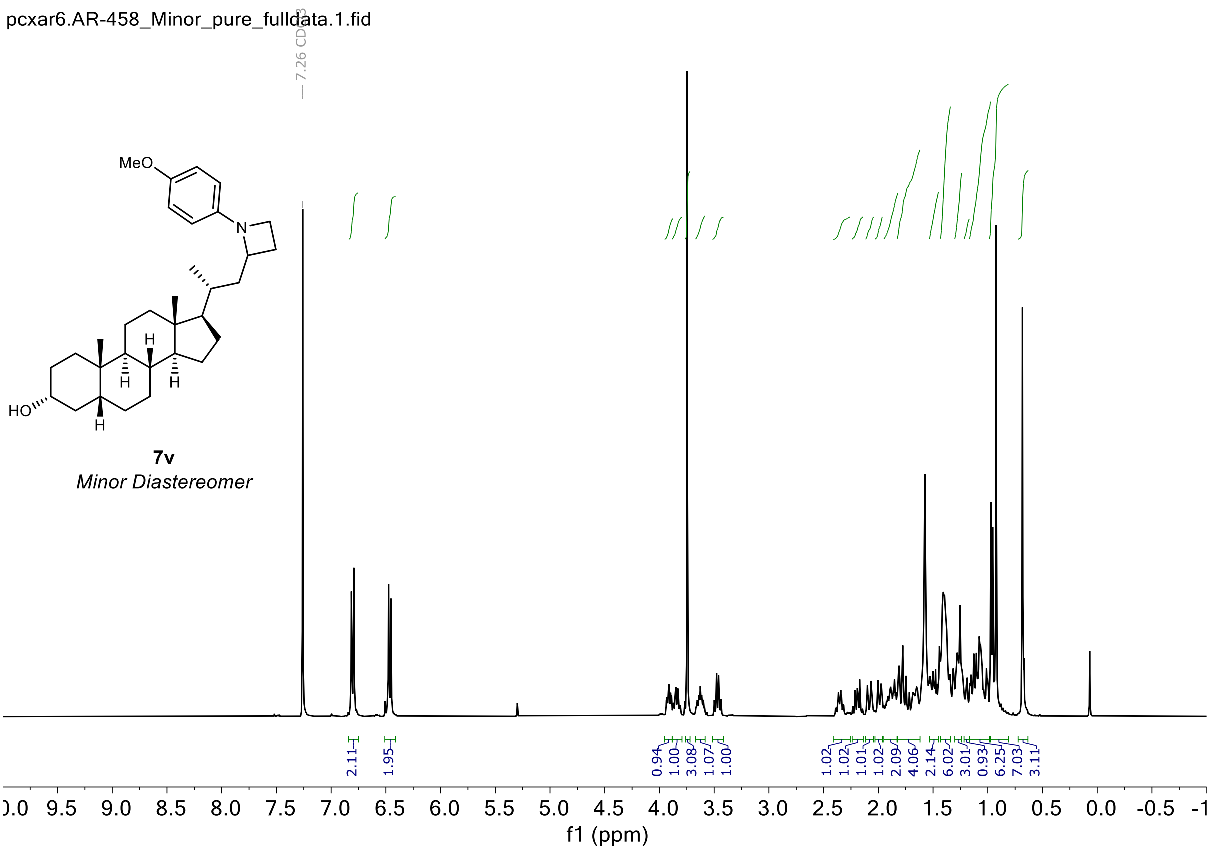

^{13}C-NMR ($CDCl_3$, 101 MHz)

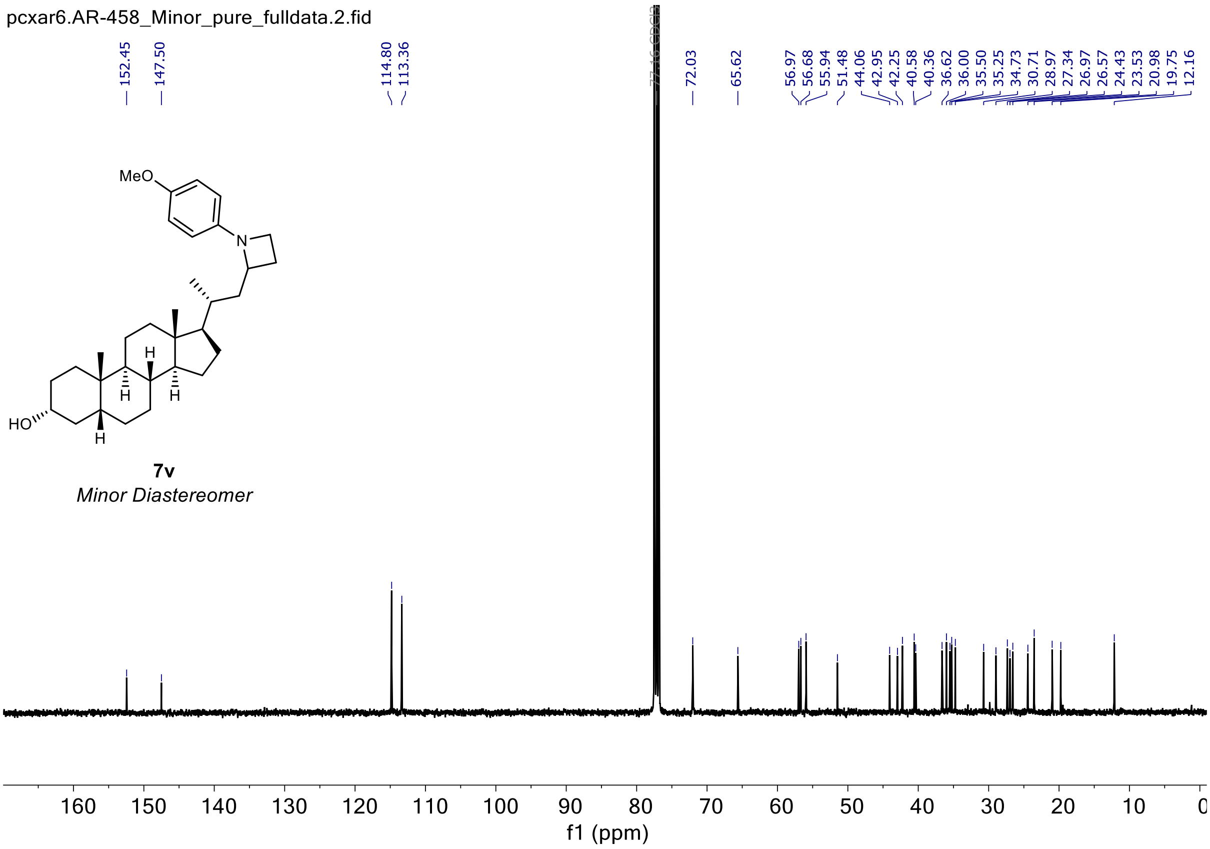

N-(4-(1-(4-methoxyphenyl)azetidin-2-yl)butyl)-4-(5-(p-tolyl)-3-(trifluoromethyl)-1H-pyrazol-1-yl)benzenesulfonamide (7w)

^{1}H-NMR ($CDCl_3$, 500 MHz)

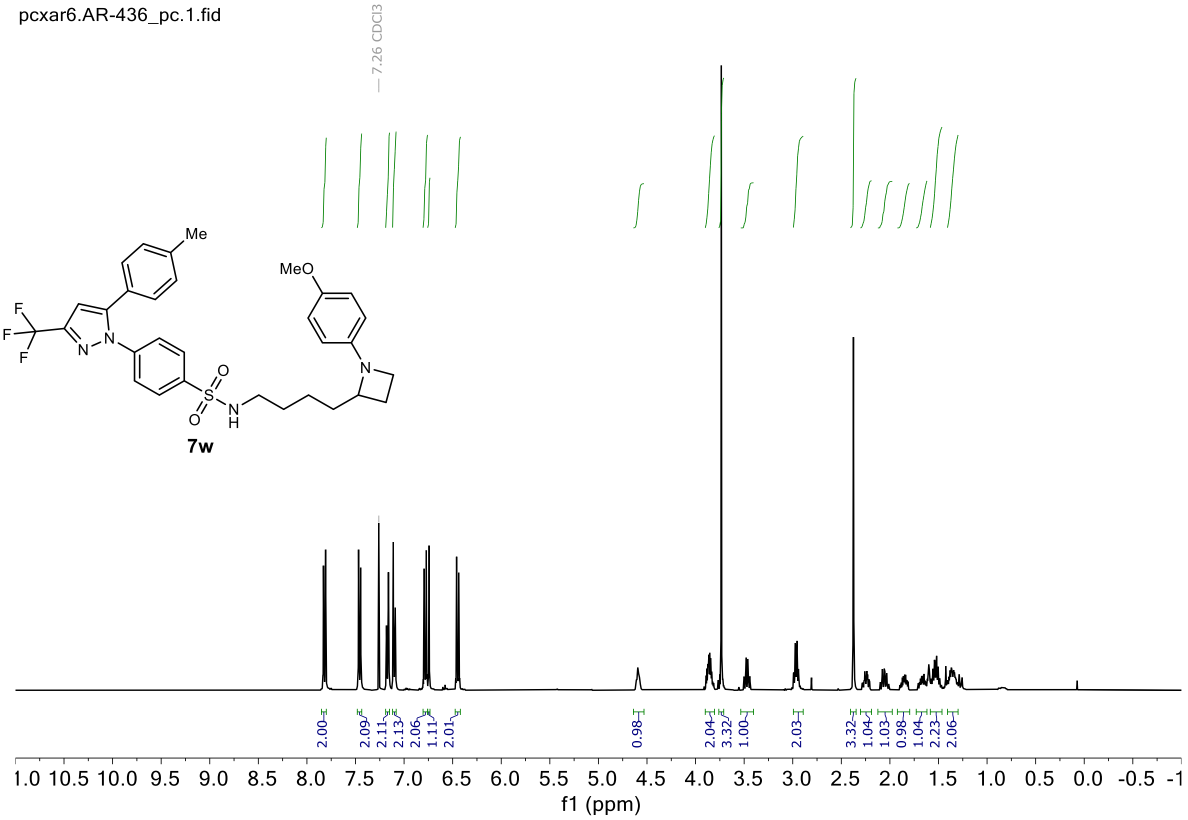

^{13}C-NMR ($CDCl_3$, 126 MHz)

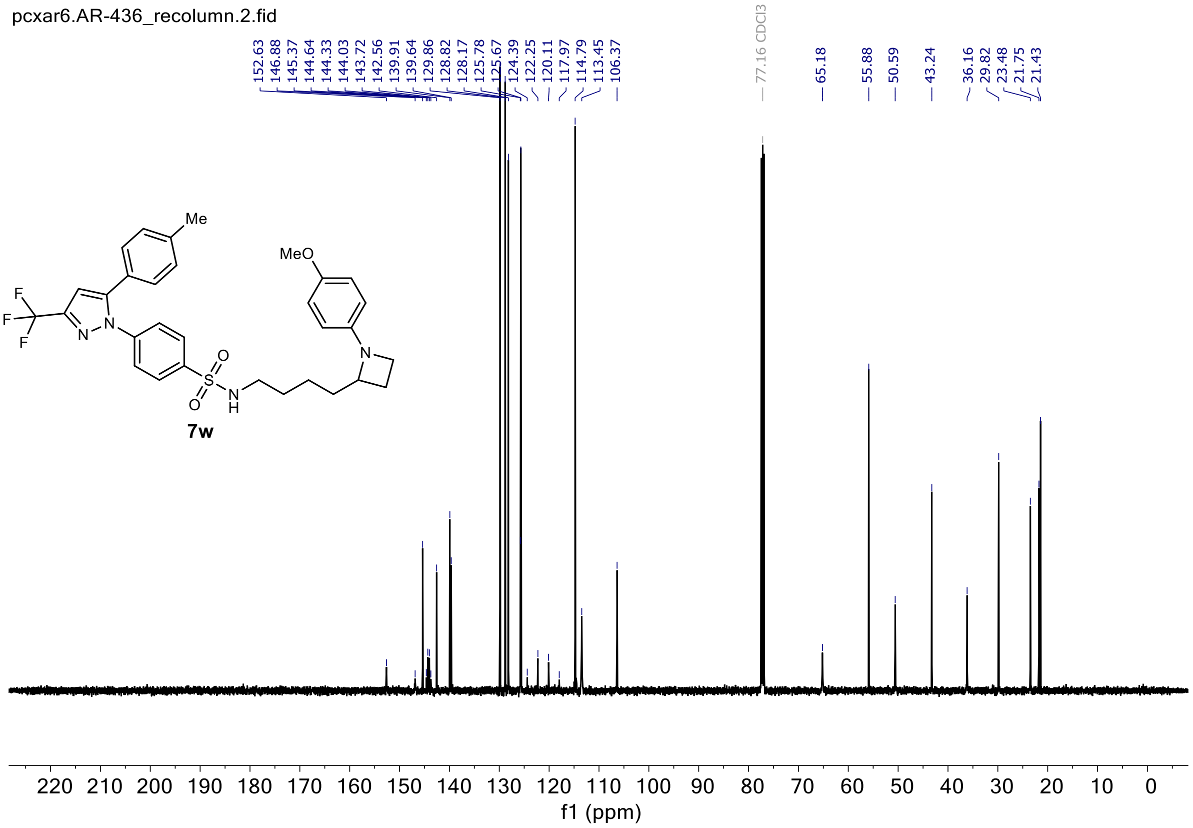

^{19}F-NMR ($CDCl_3$, 376 MHz)

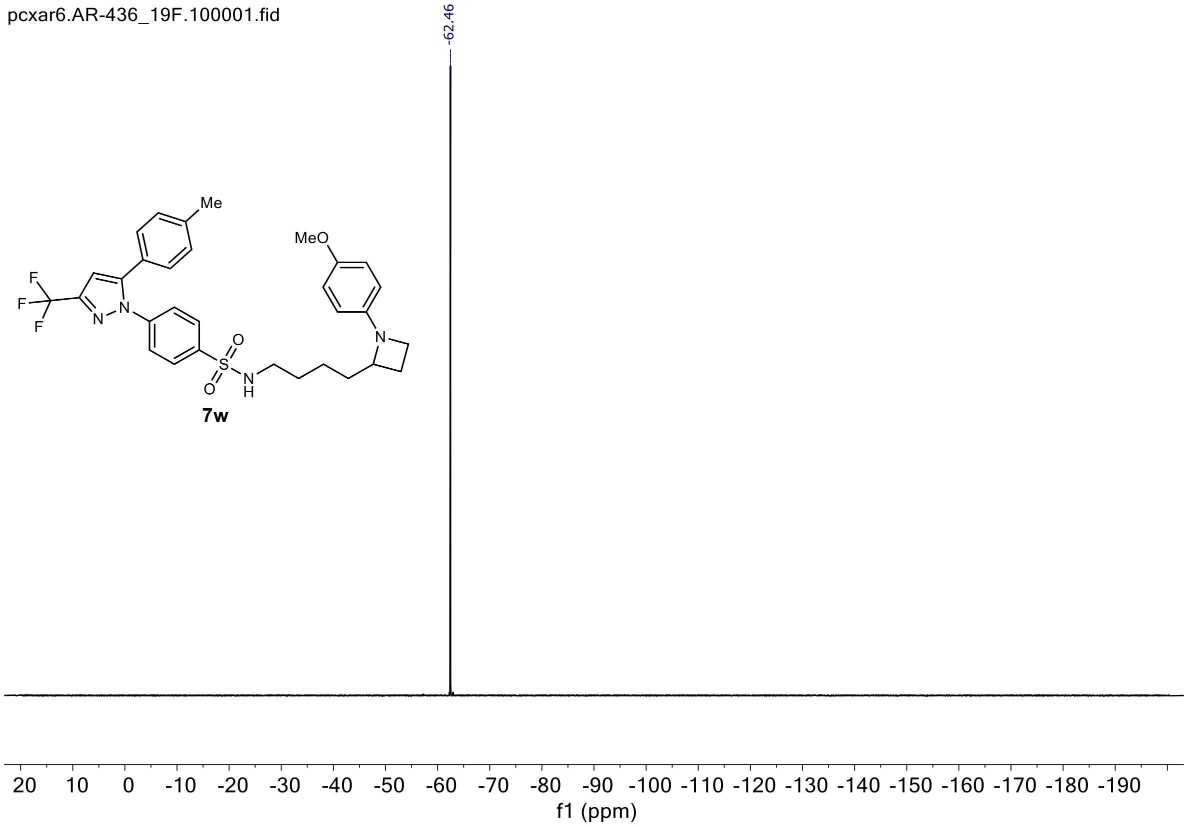

4-(1-(4-methoxyphenyl)azetidin-2-yl)butyl-2-(11-oxo-6,11-dihydrodibenzo[b,e]oxepin-2-yl)acetate (7x)

^{1}H-NMR ($CDCl_3$, 500 MHz)

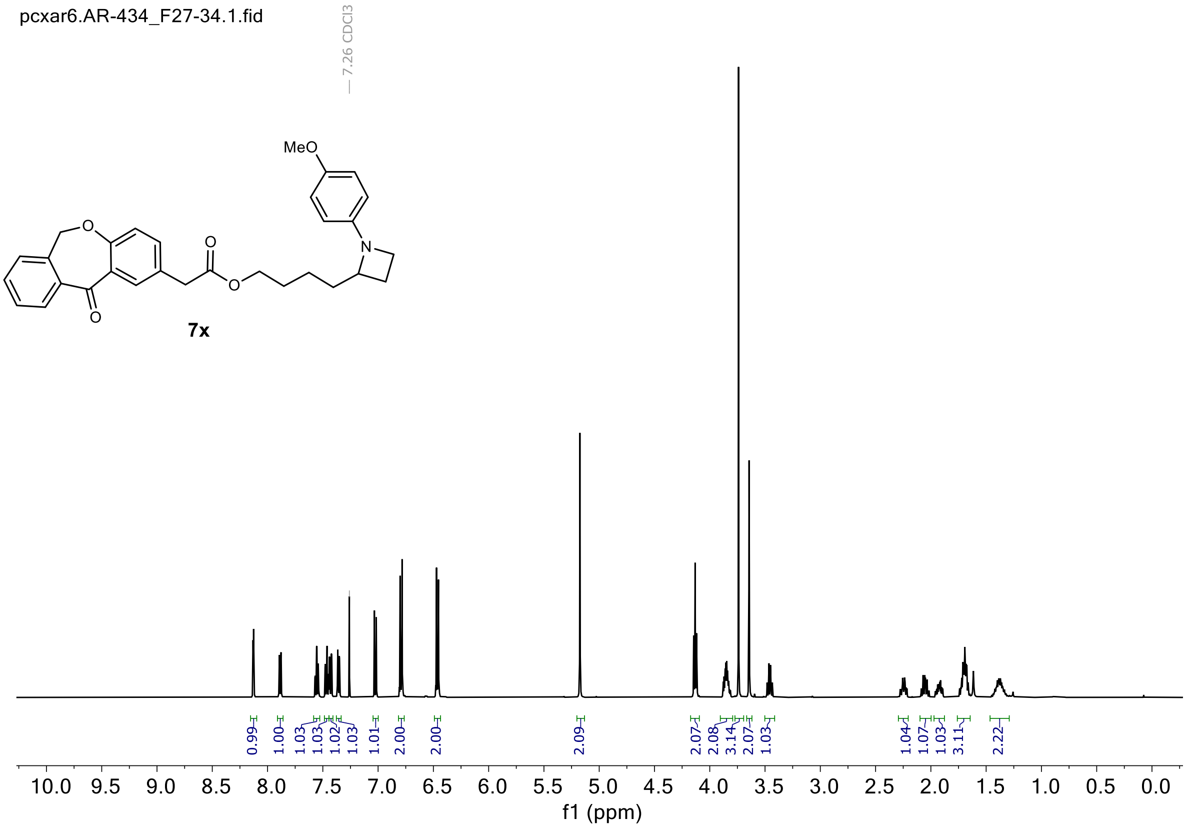

^{13}C-NMR ($CDCl_3$, 126 MHz)

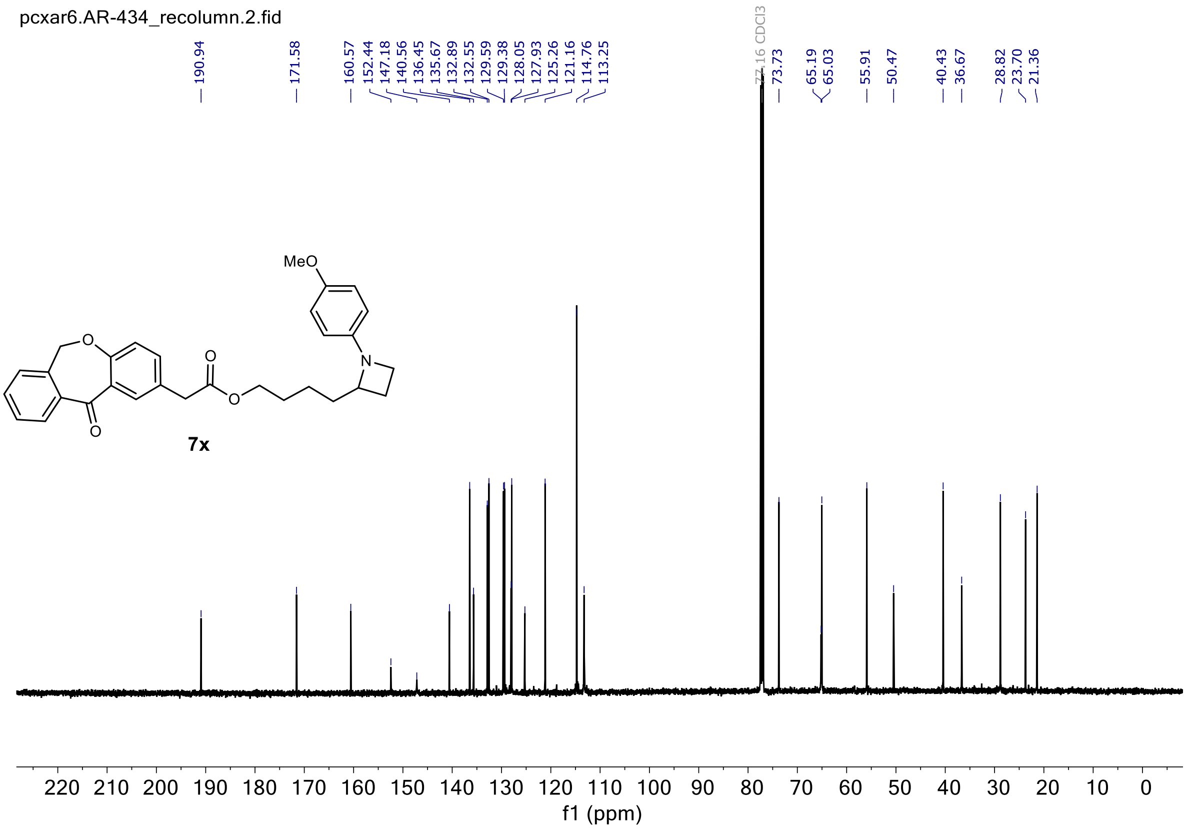

3-(1-(4-methoxyphenyl)azetidin-2-yl)propyl 2-(3-cyano-4-isobutoxyphenyl)-4-methylthiazole-5-carboxylate (7y)

^{1}H-NMR ($CDCl_3$, 500 MHz)

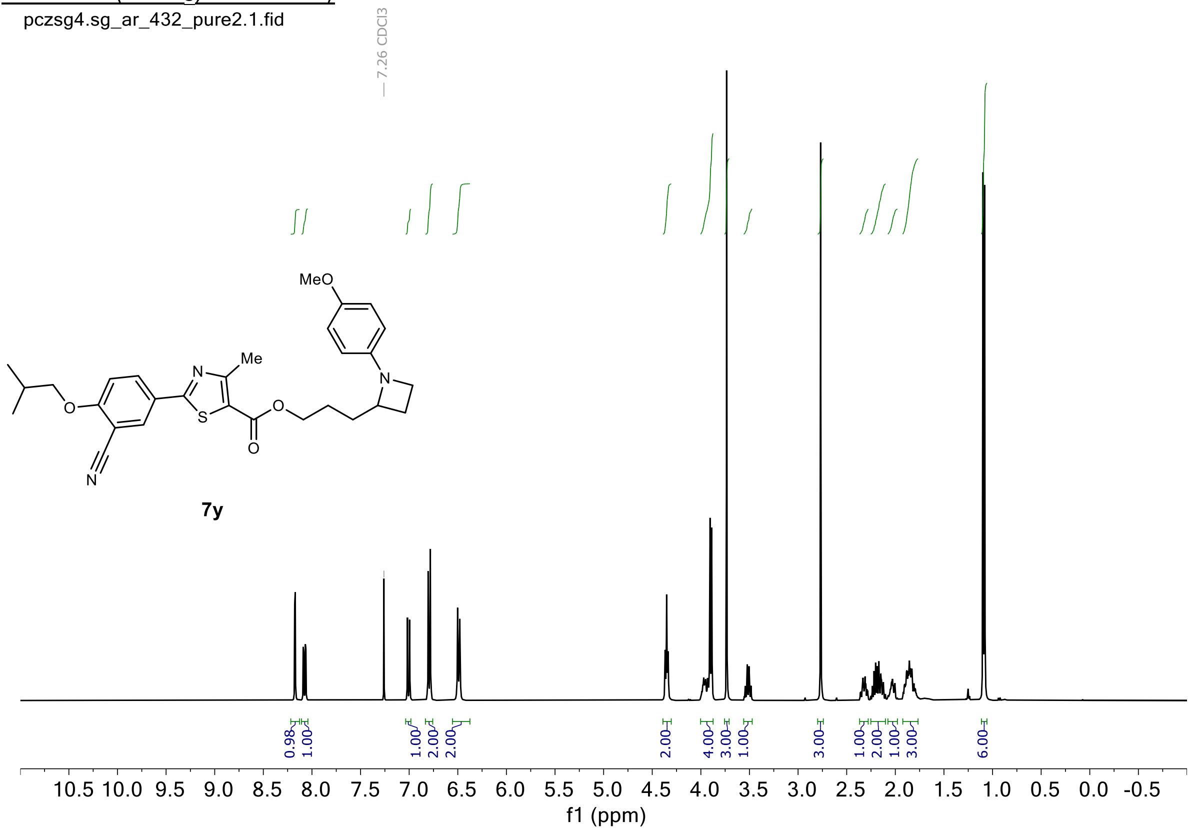

^{13}C-NMR ($CDCl_3$, 126 MHz)

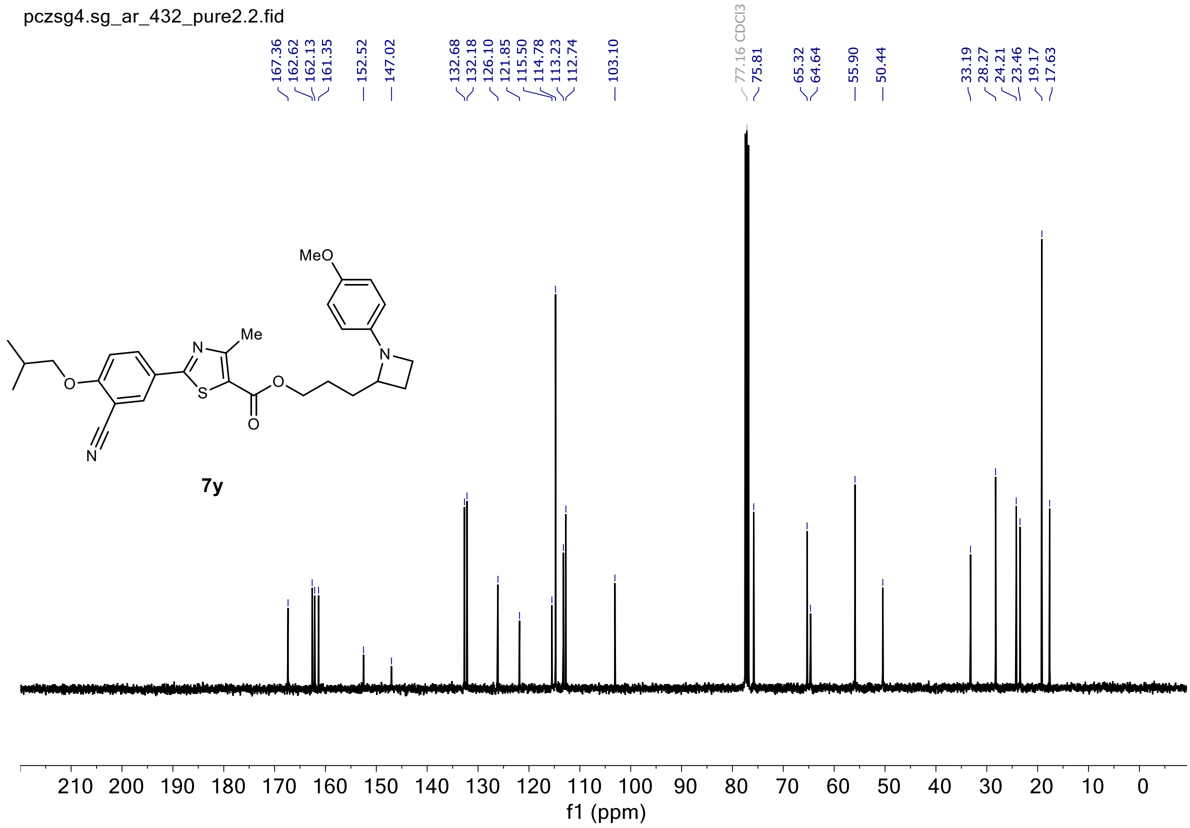

2-Phenethyl-1-phenylazetidine (7aa)

^{1}H-NMR ($CDCl_3$, 500 MHz)

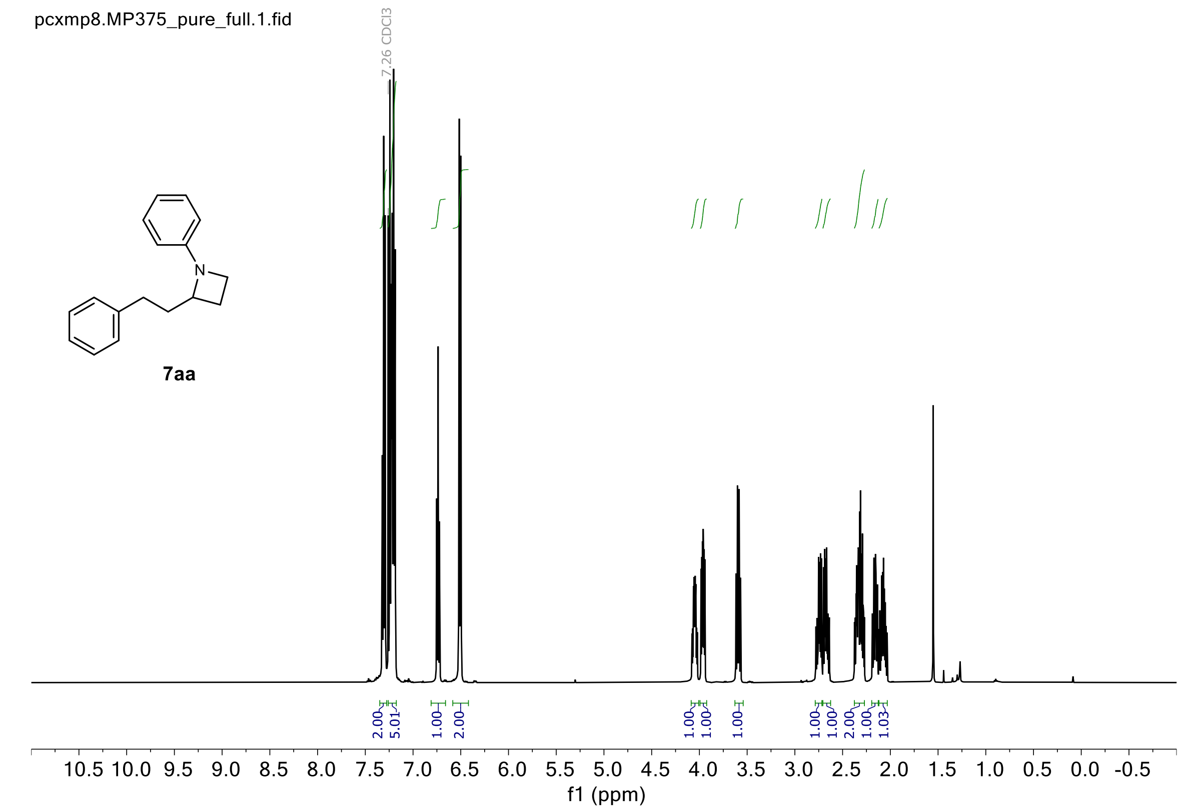

^{13}C-NMR ($CDCl_3$, 126 MHz)

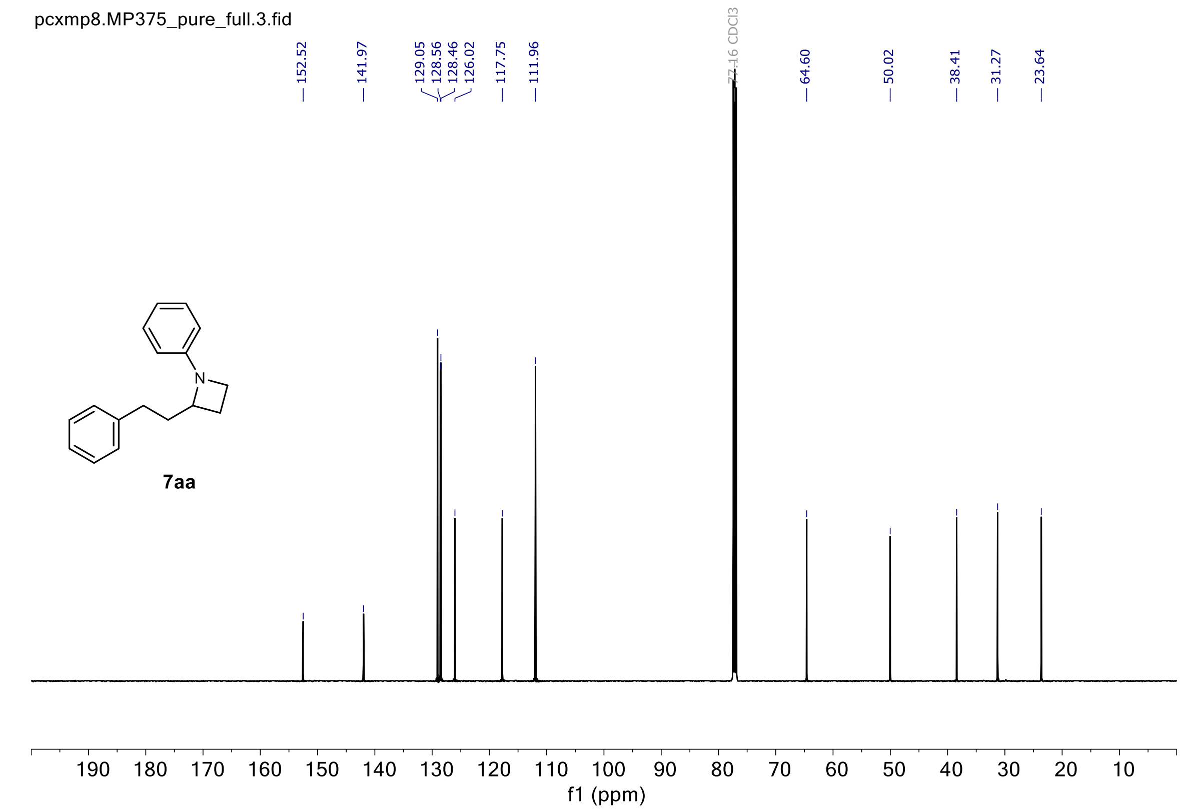

Methyl 3-(2-phenethylazetidin-1-yl)benzoate (7ab)

^{1}H-NMR (CDCl$_3$, 500 MHz)

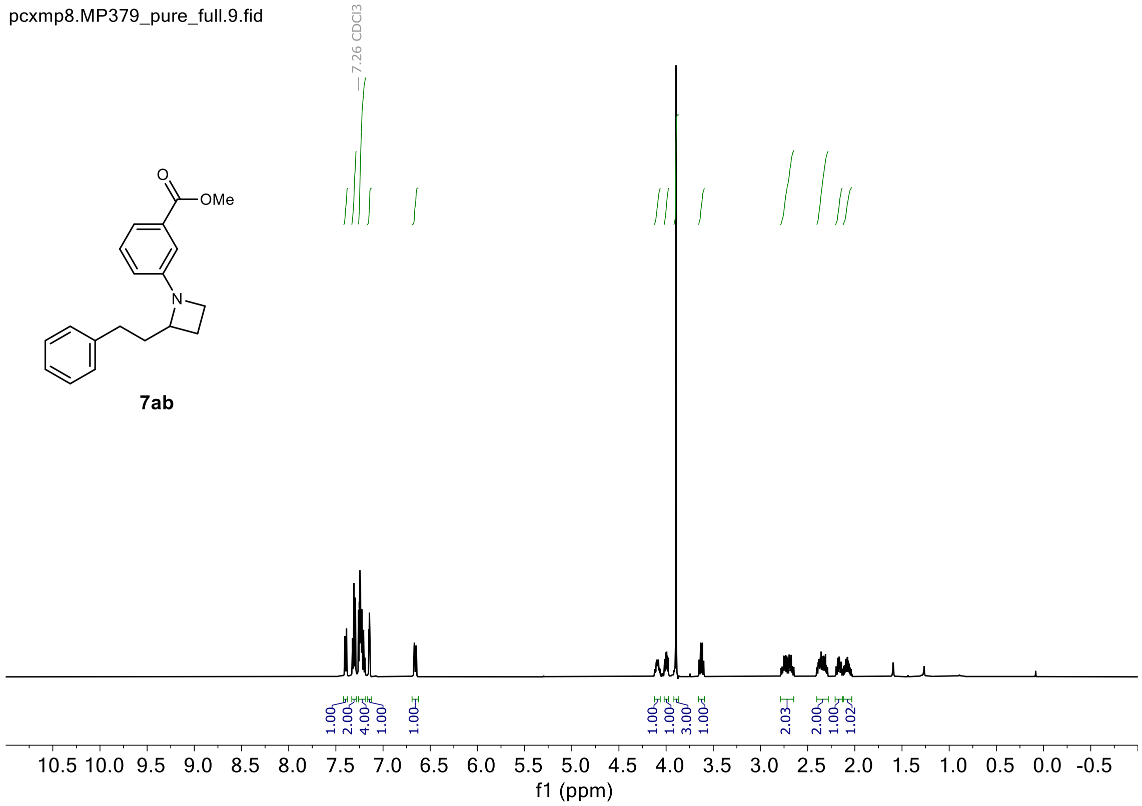

^{13}C-NMR (CDCl$_3$, 126 MHz)

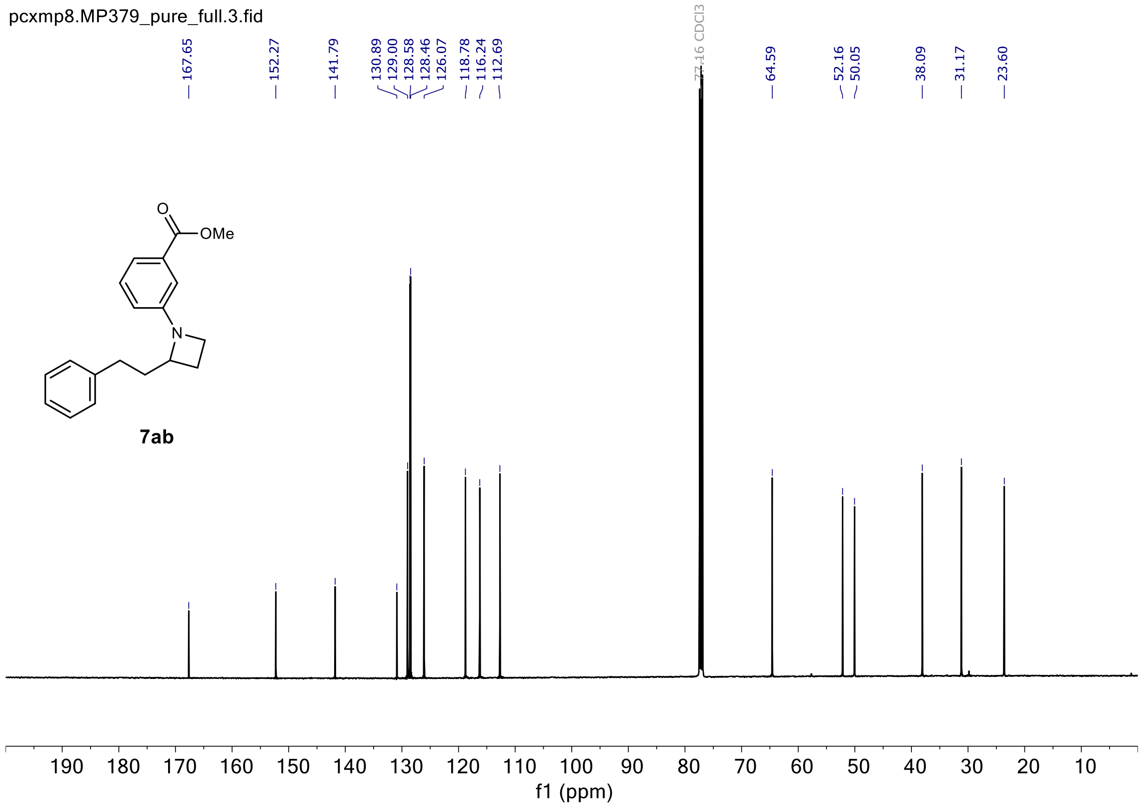

Ethyl 4-(2-phenethylazetidin-1-yl)benzoate (7ac)

^{1}H-NMR (CDCl$_3$, 500 MHz)

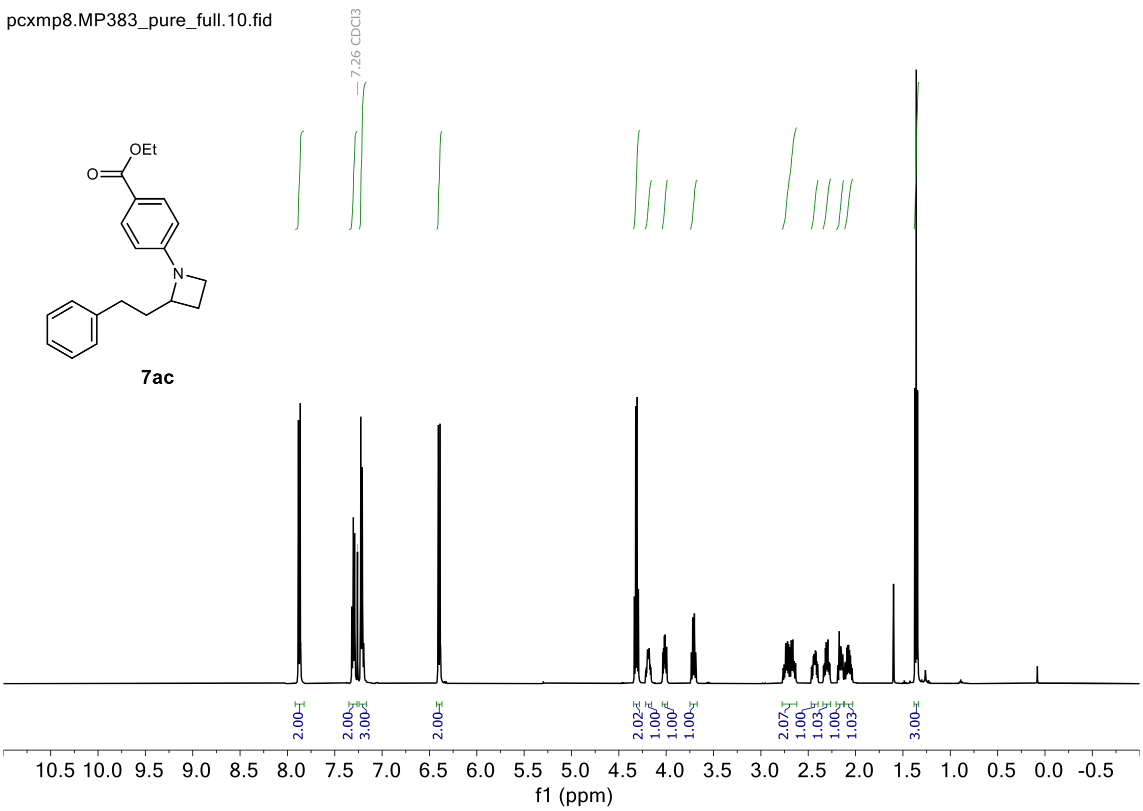

^{13}C-NMR (CDCl$_3$, 126 MHz)

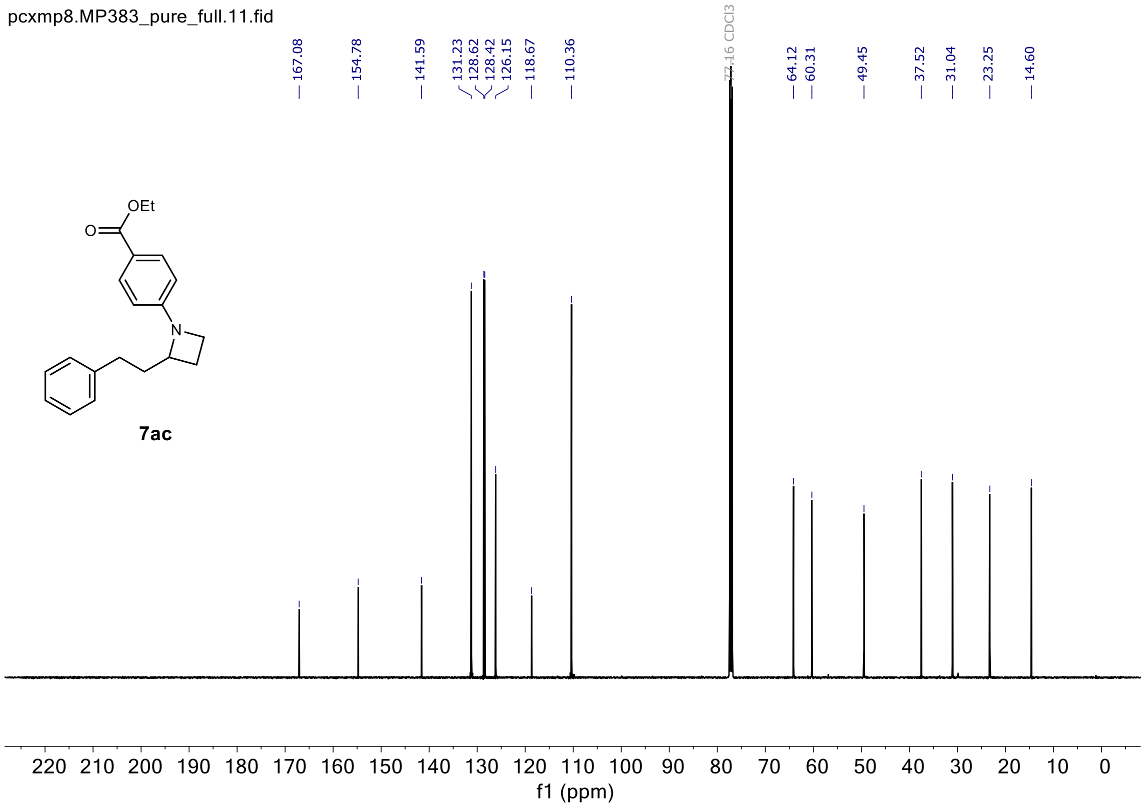

5-(2-phenethylazetidin-1-yl)-1H-indole (7ad)

^{1}H-NMR (CDCl$_3$, 500 MHz)

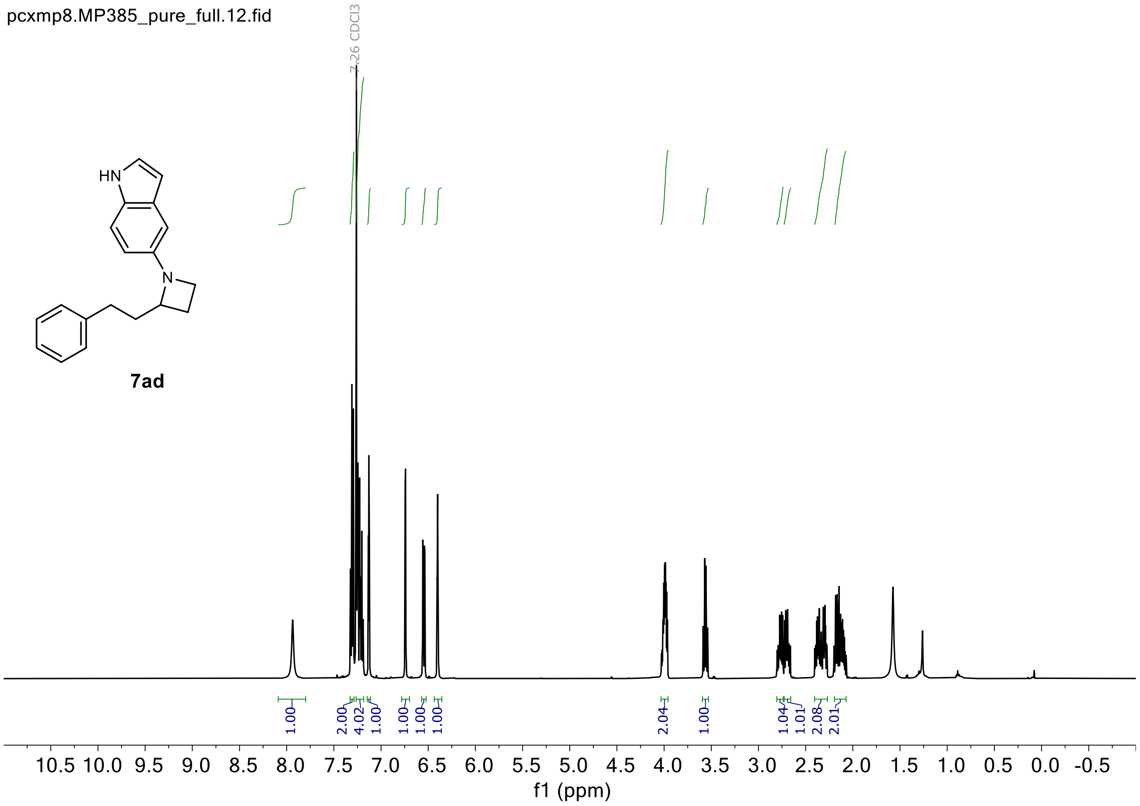

^{13}C-NMR (CDCl$_3$, 126 MHz)

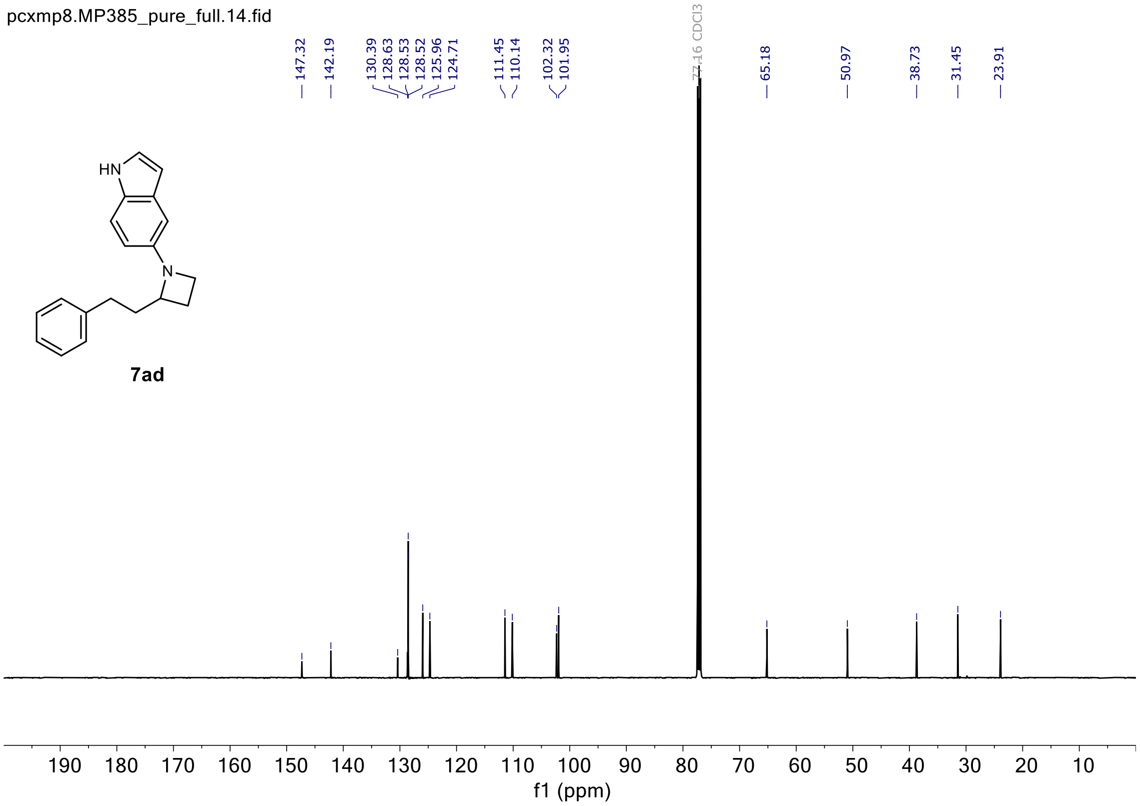

1-benzyl-2-phenethylazetidine (7ae)

^{1}H-NMR ($CDCl_3$, 500 MHz)

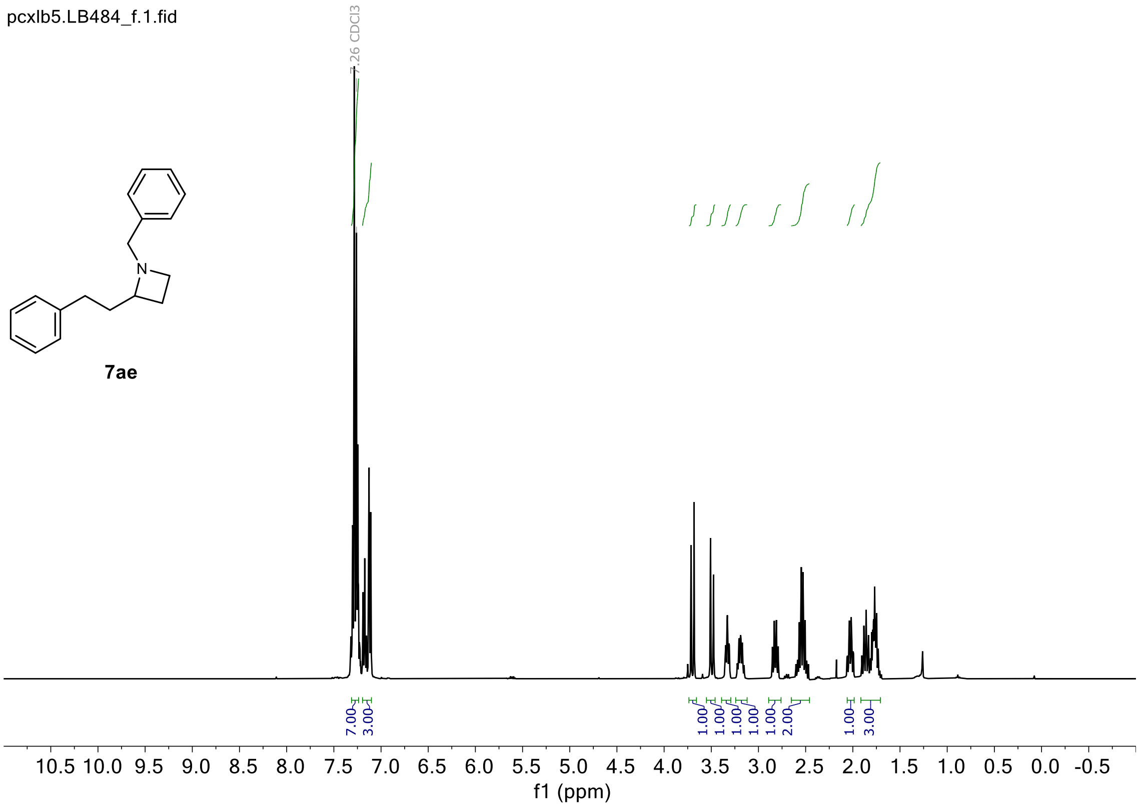

^{13}C-NMR ($CDCl_3$, 126 MHz)

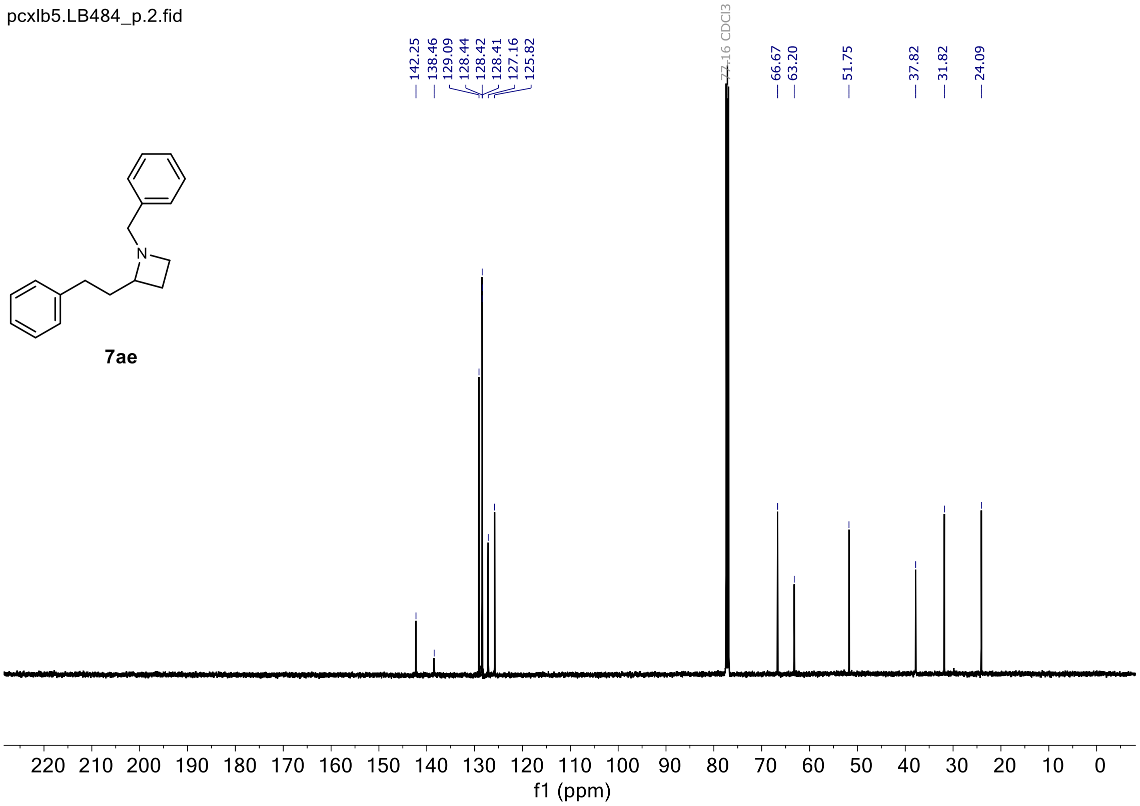

1-(4-methoxybenzyl)-2-phenethylazetidine (7af)

^{1}H-NMR (CDCl$_3$, 500 MHz)

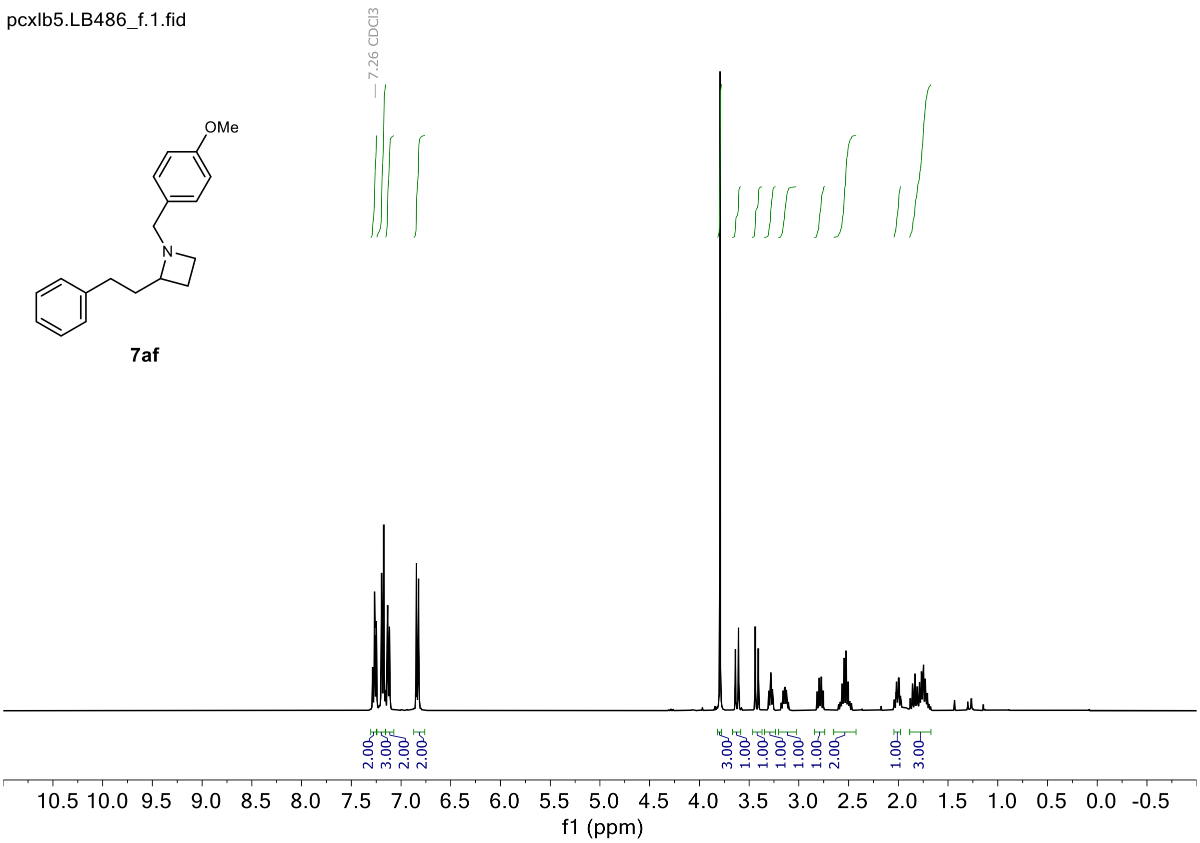

^{13}C-NMR (CDCl$_3$, 126 MHz)

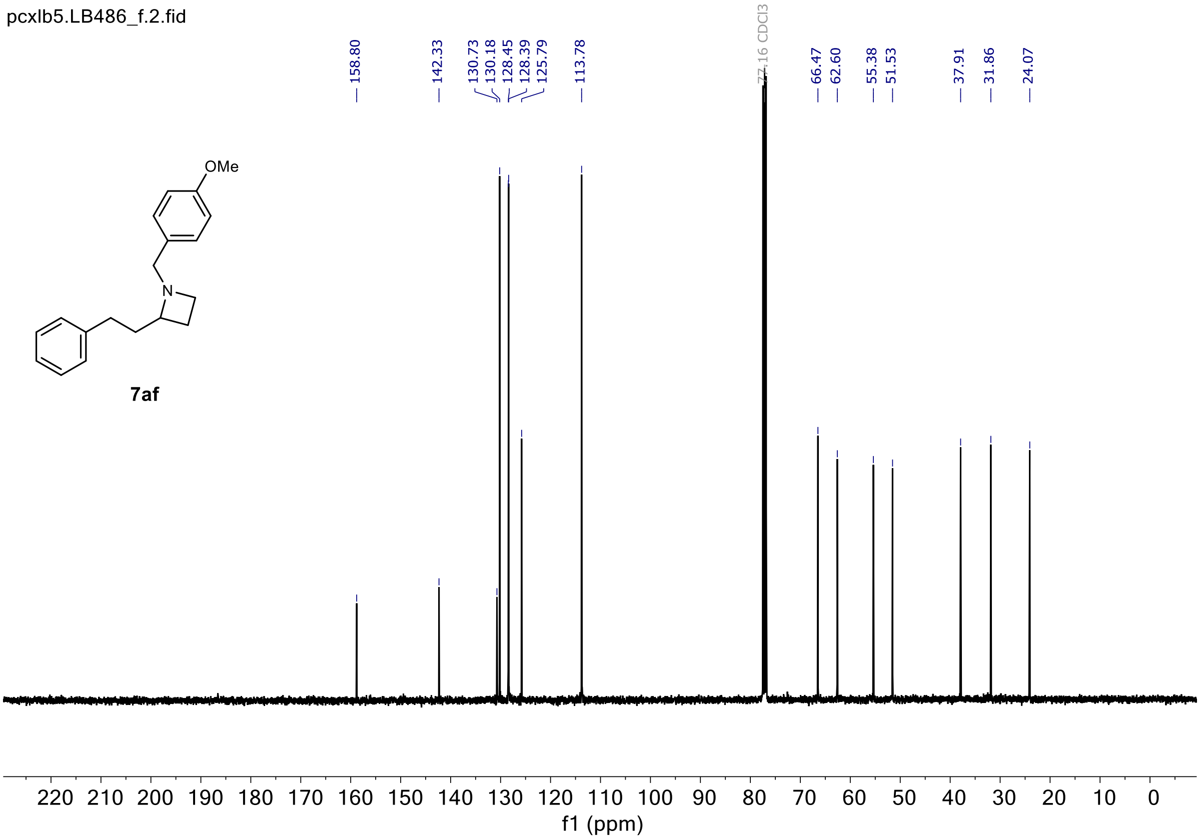

1-cyclohexyl-2-phenethylazetidine (7ag)

^{1}H-NMR ($CDCl_3$, 500 MHz)

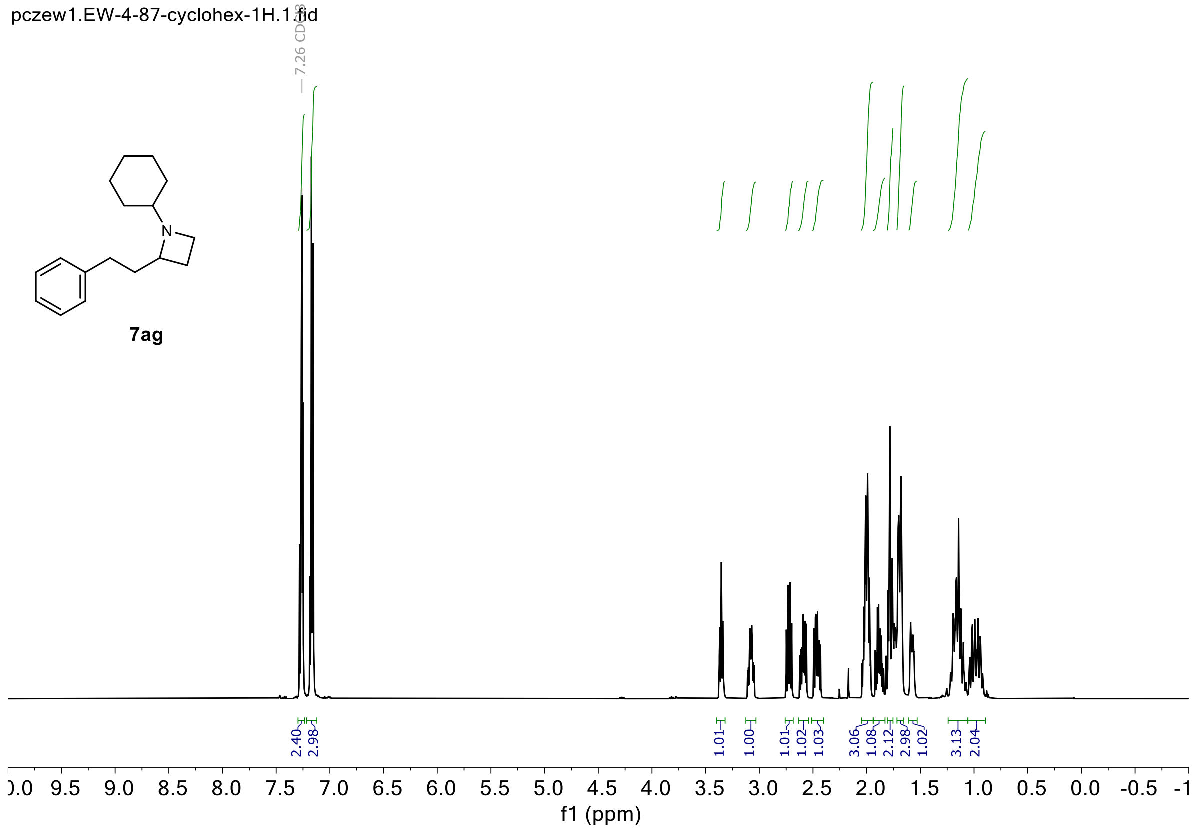

^{13}C-NMR ($CDCl_3$, 126 MHz)

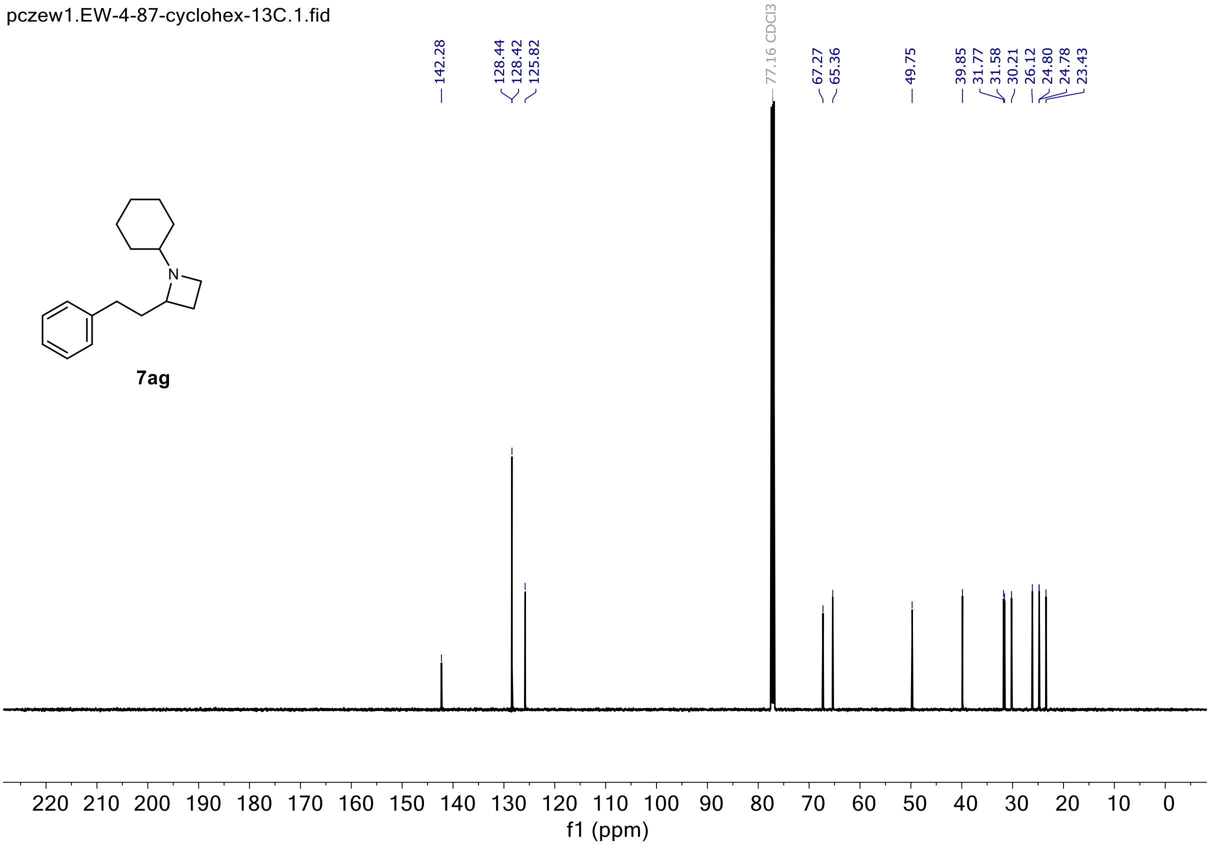

2-phenethyl-1-(tetrahydro-2H-pyran-4-yl)azetidine (7ah)

^{1}H-NMR (CDCl$_3$, 500 MHz)

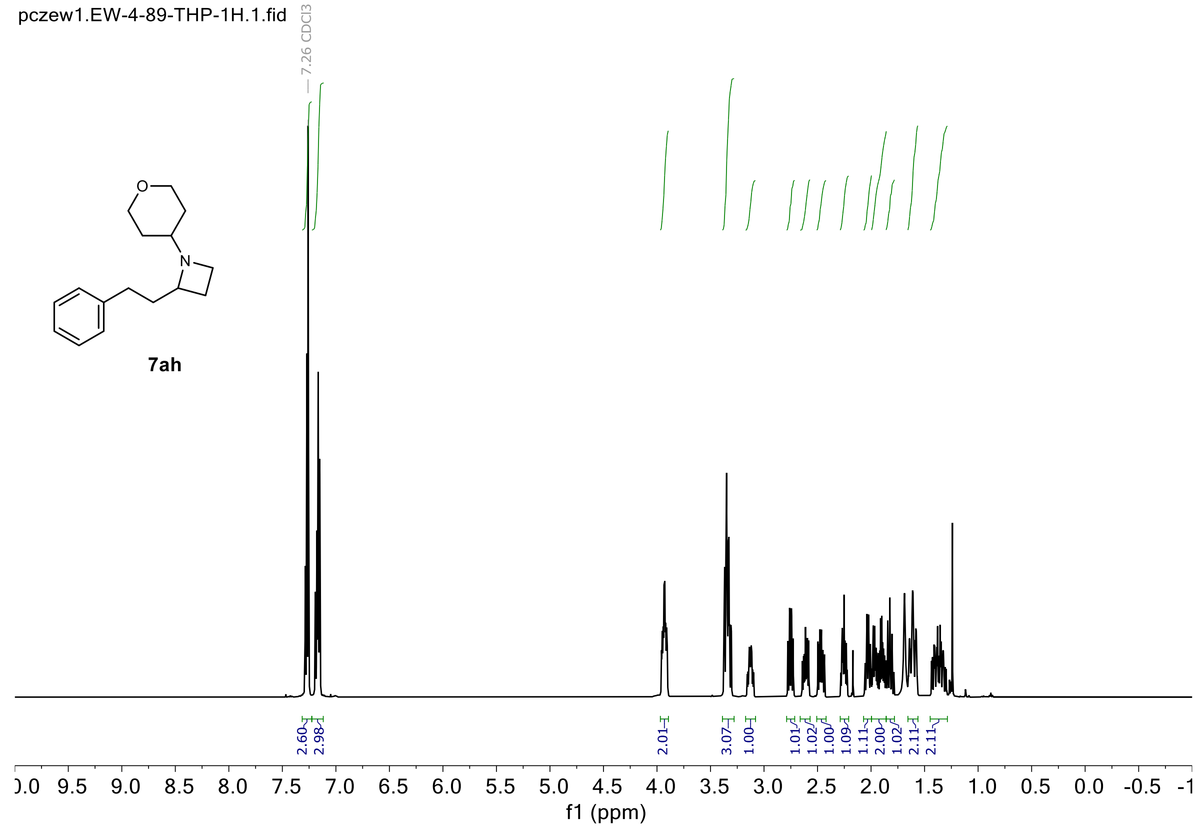

^{13}C-NMR (CDCl$_3$, 126 MHz)

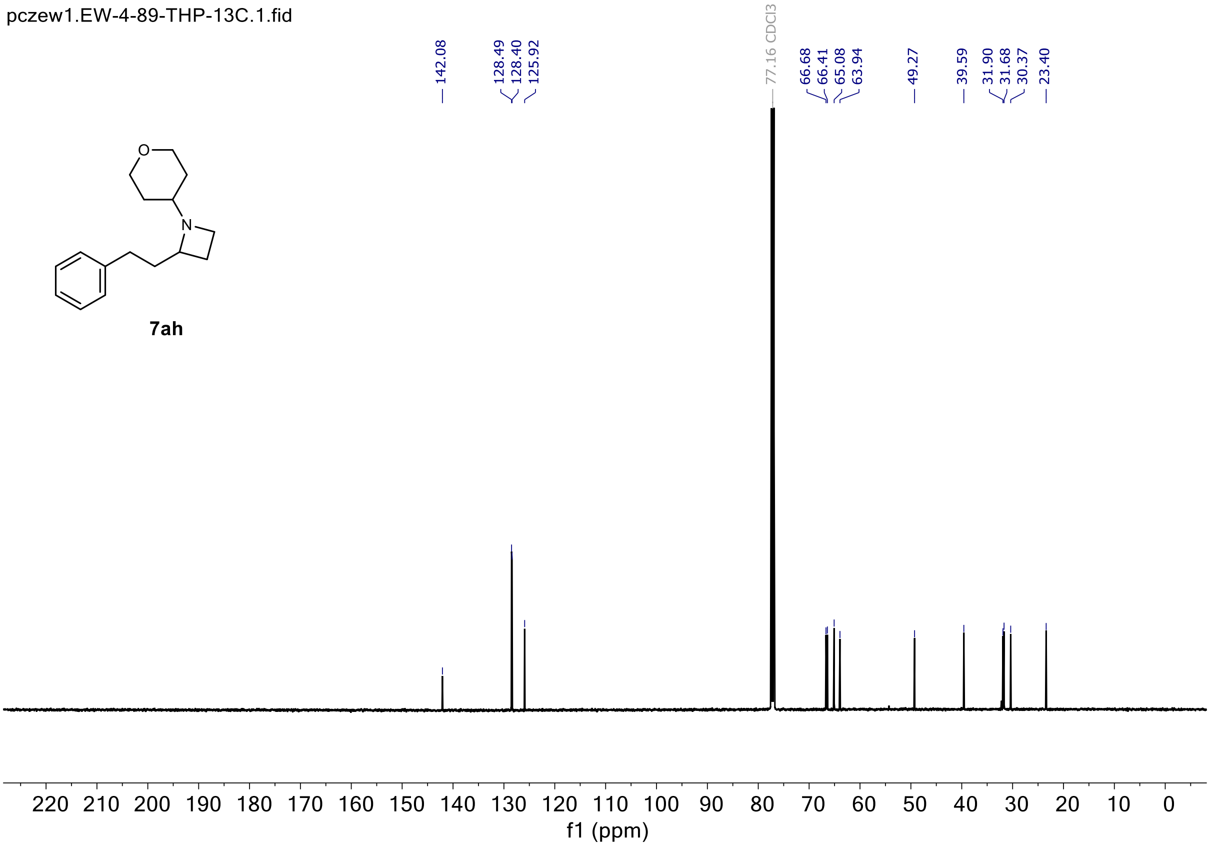

1-cyclopropyl-2-phenethylazetidine (7ai)

^{1}H-NMR ($CDCl_3$, 500 MHz)

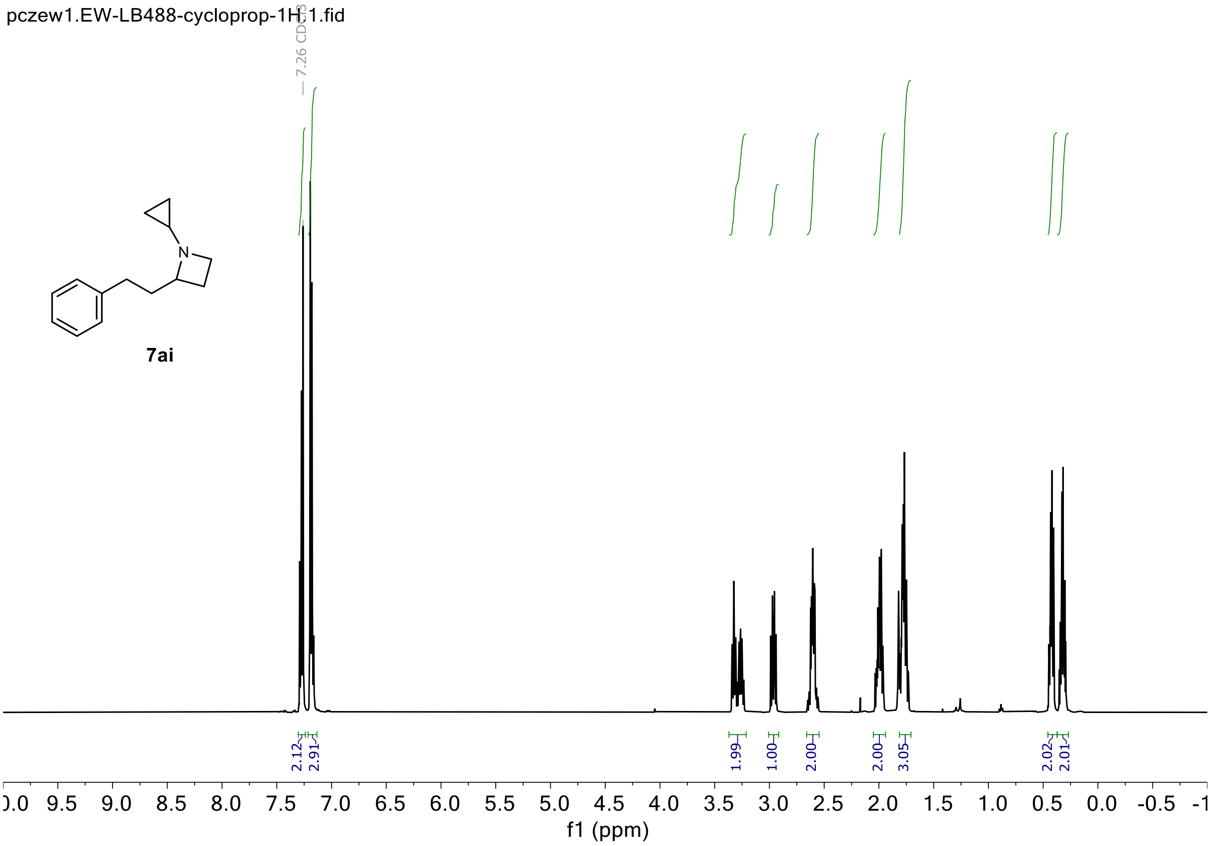

^{13}C-NMR ($CDCl_3$, 126 MHz)

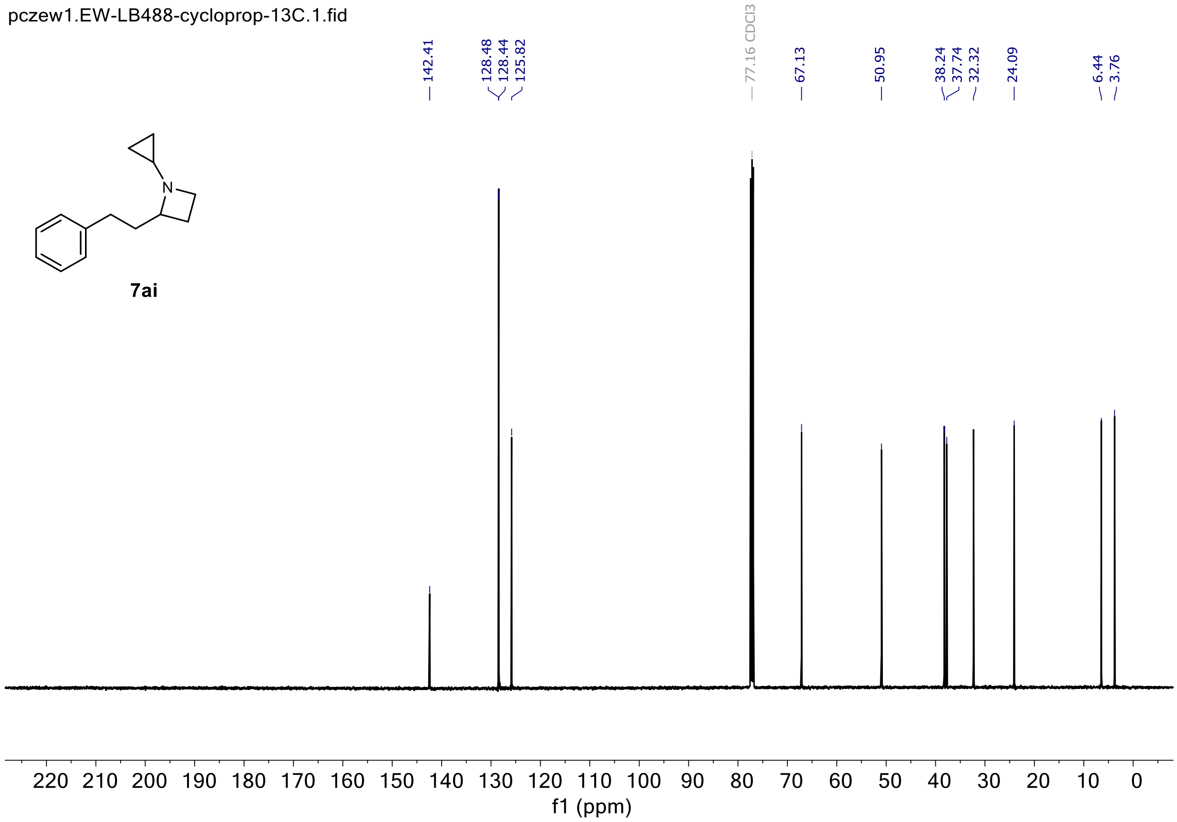

3-(2-phenethylazetidin-1-yl)propan-1-ol (7aj)

^{1}H-NMR ($CDCl_3$, 500 MHz)

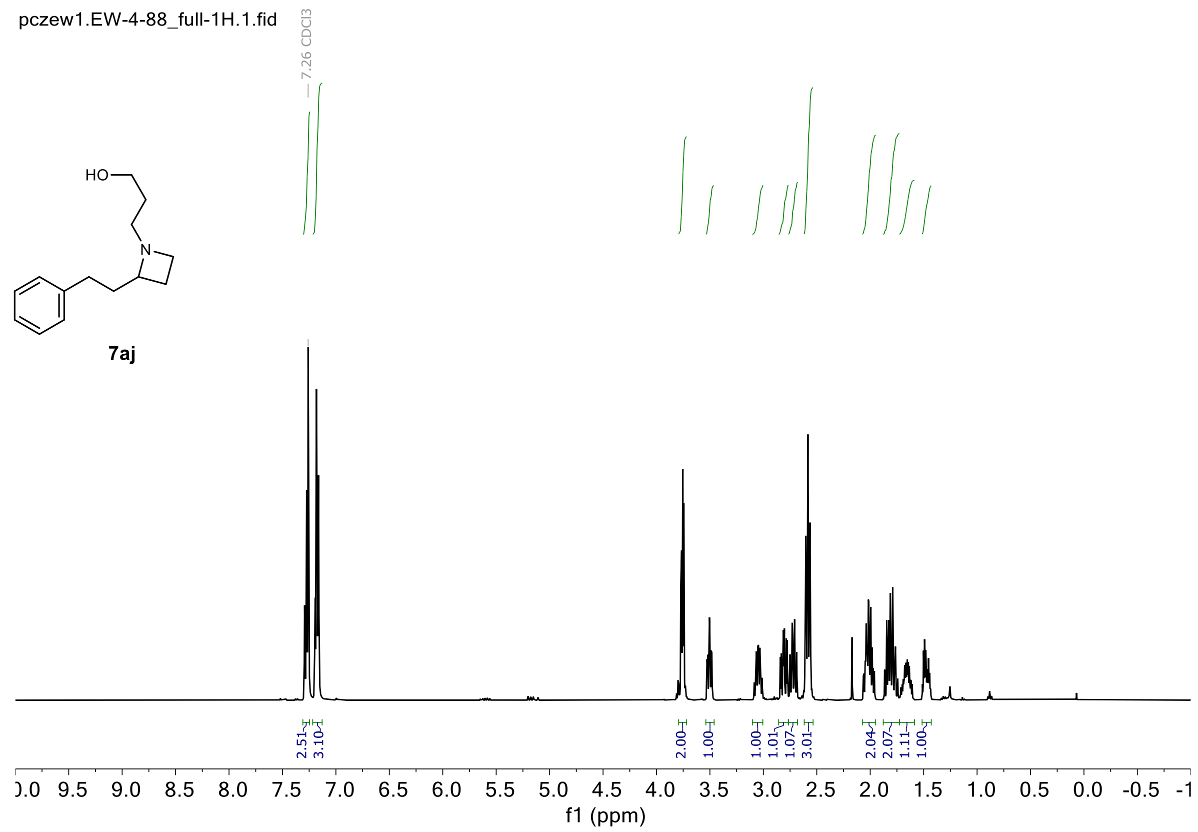

^{13}C-NMR ($CDCl_3$, 126 MHz)

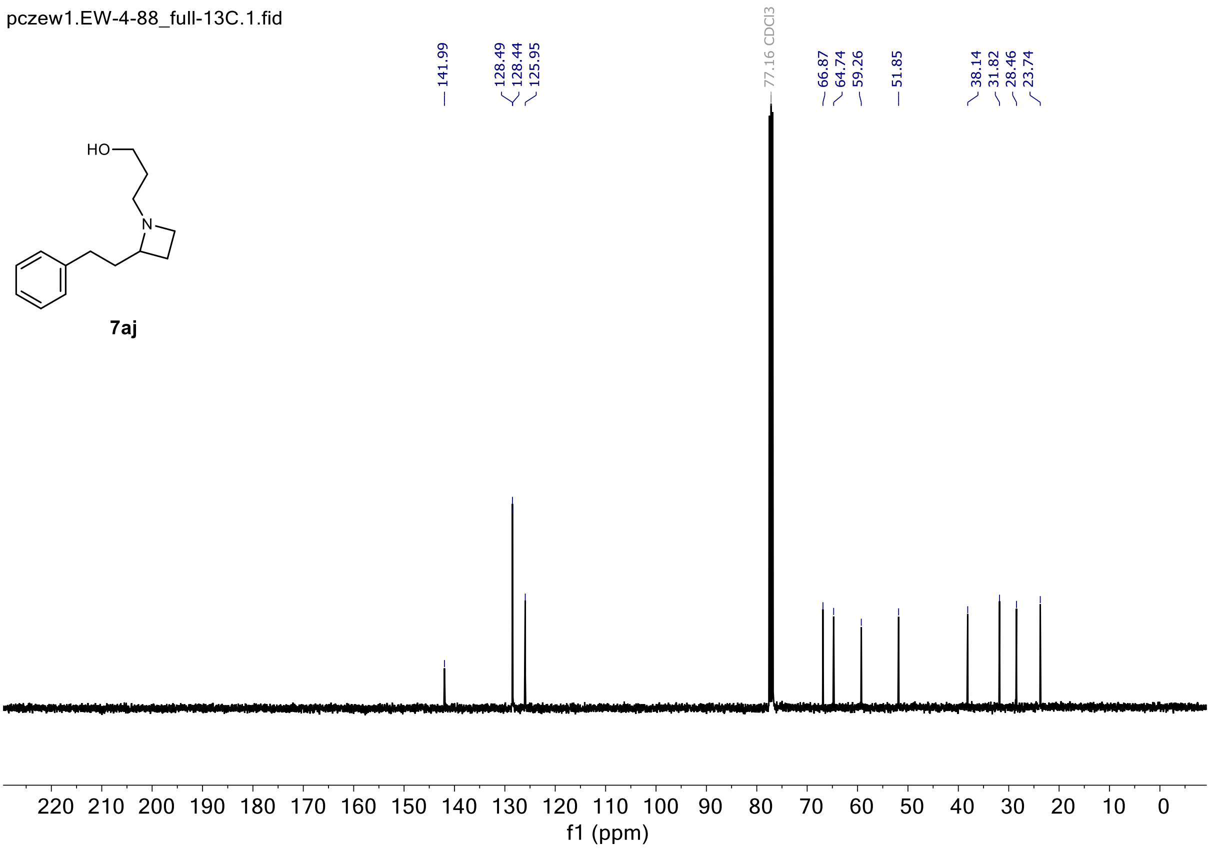

2-phenethylazetidine trifluoroacetic acid (7ba)

^{1}H-NMR (CD_3OD, 500 MHz)

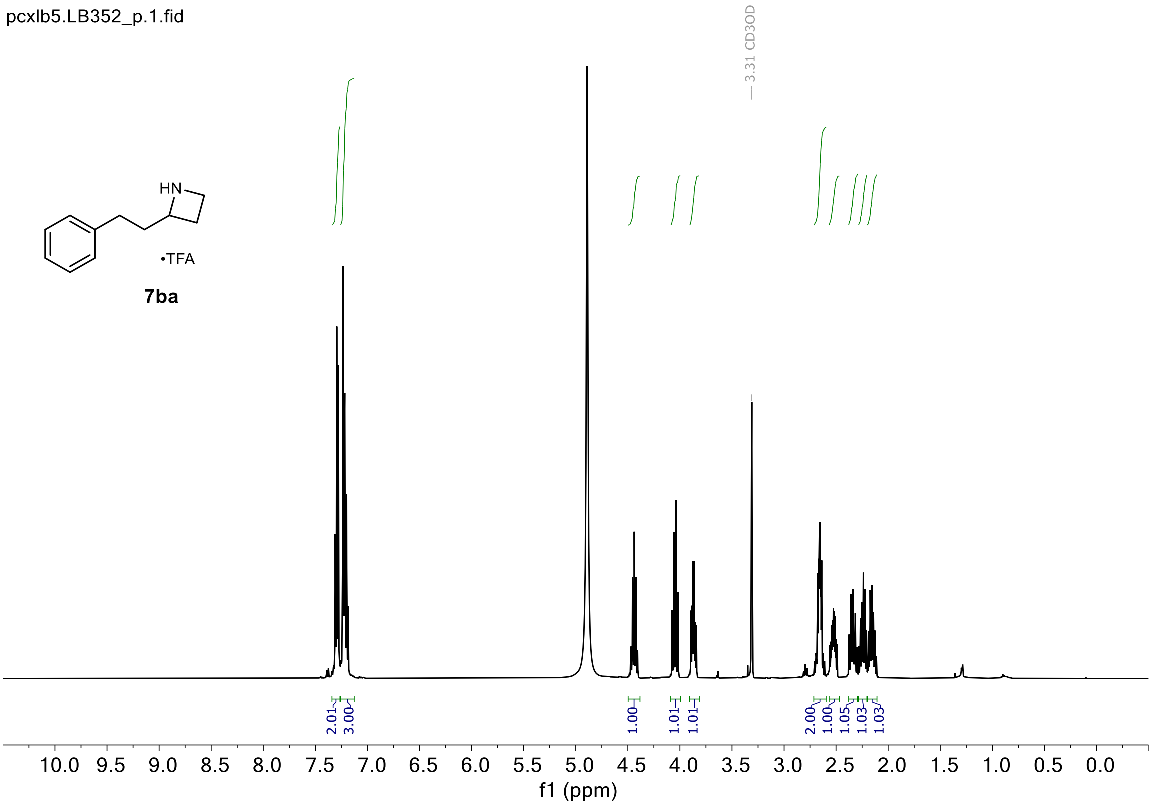

^{13}C-NMR (CD_3OD, 126 MHz)

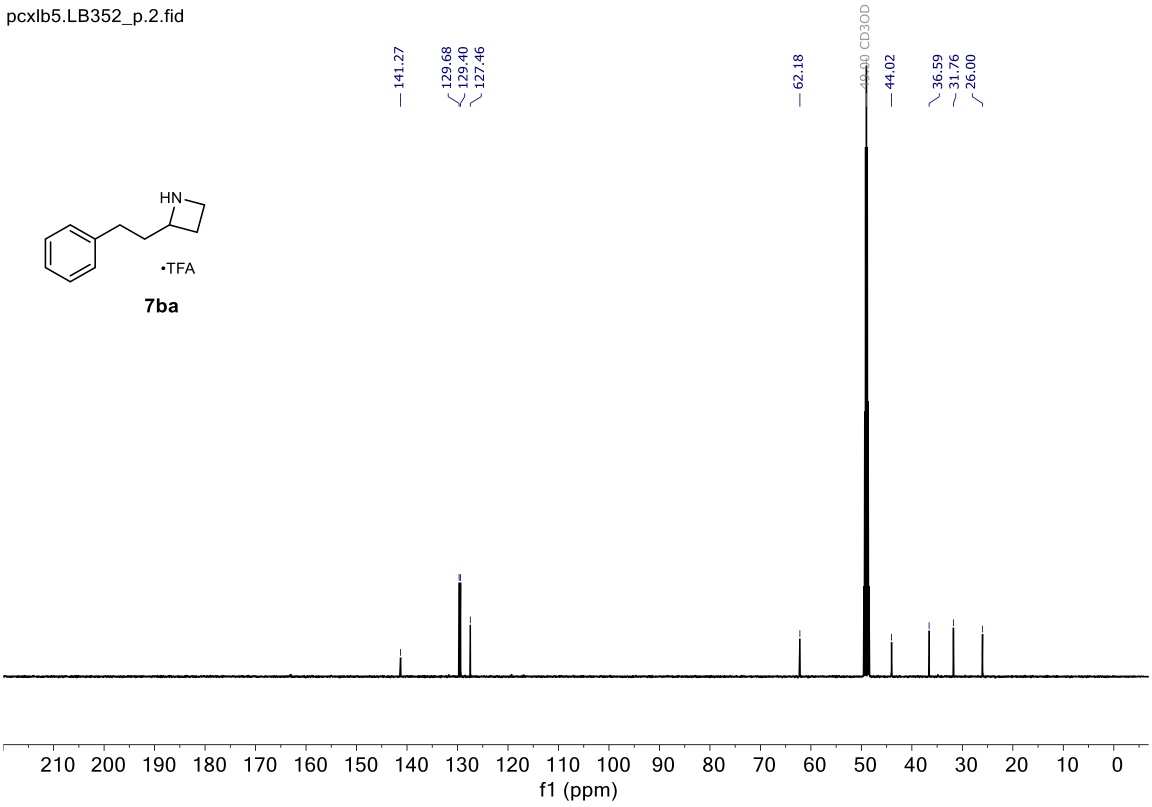

^{19}F-NMR (CD_3OD, 376 MHz)

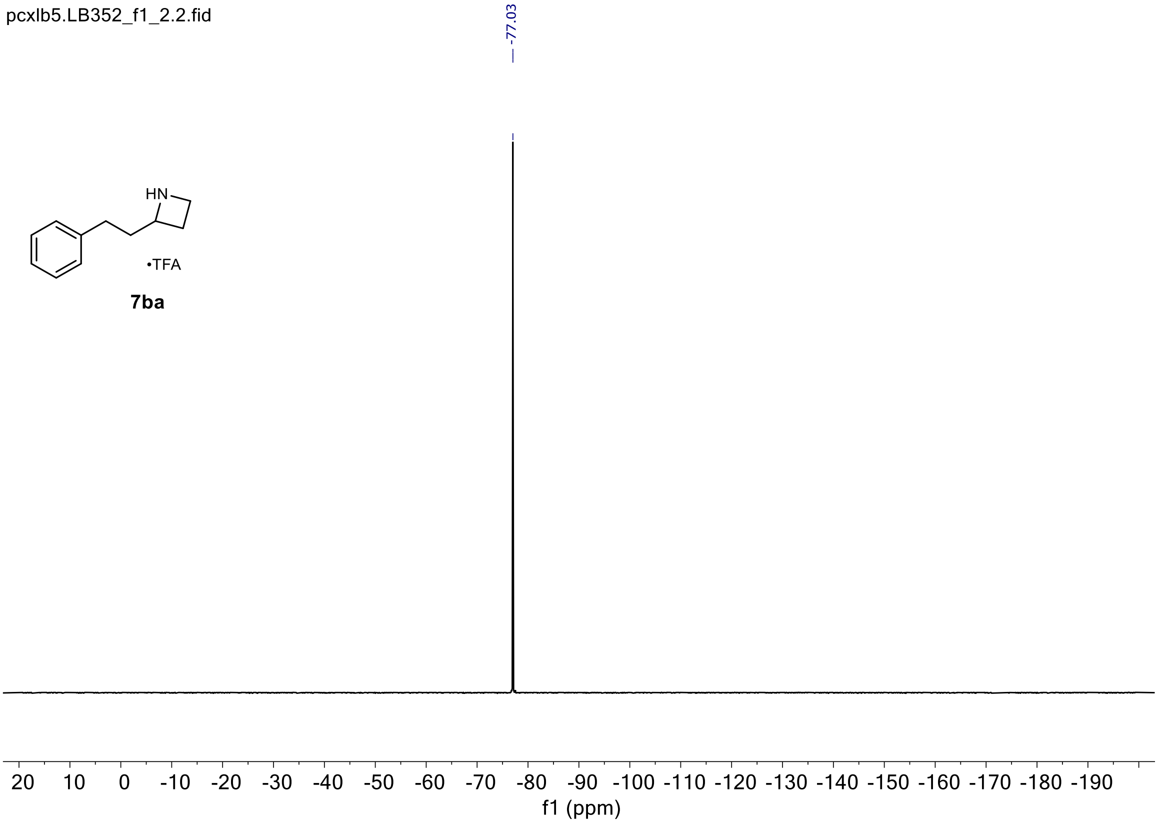

9-(azetidin-2-yl)nonanamide (7bb)

^{1}H-NMR ($CDCl_3$, 500 MHz)

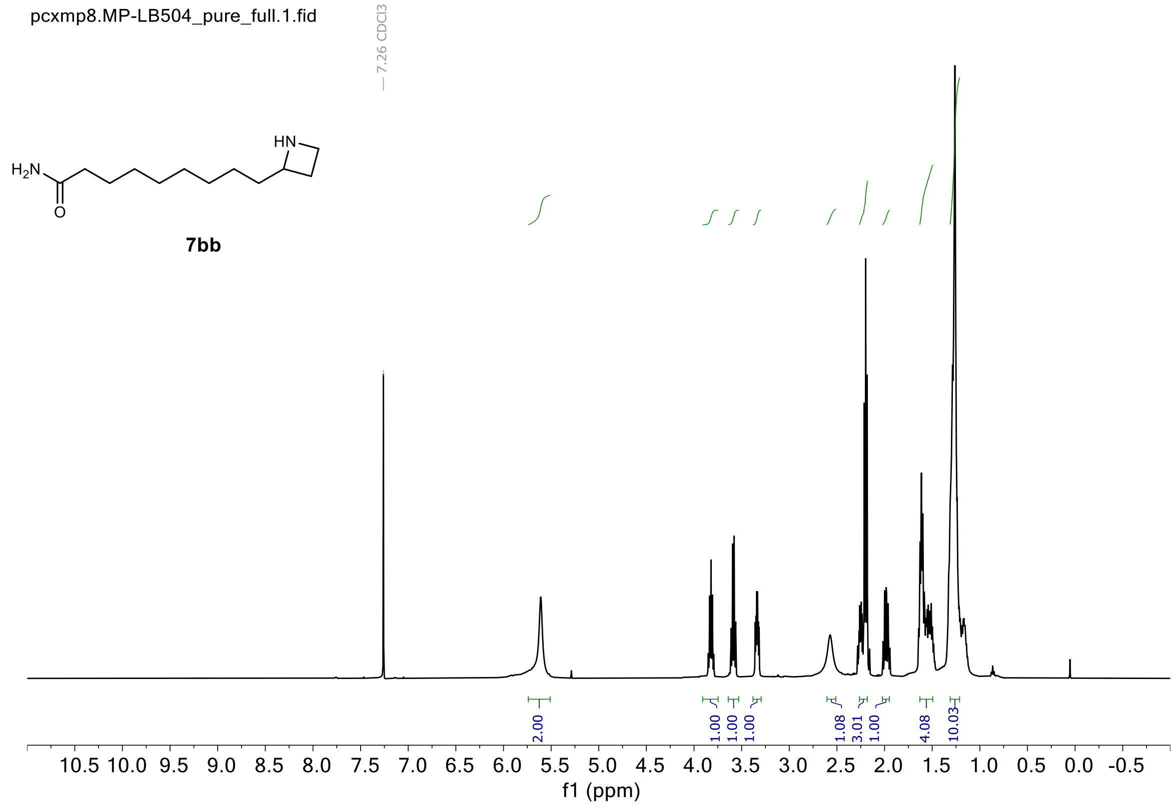

^{13}C-NMR ($CDCl_3$, 500 MHz)

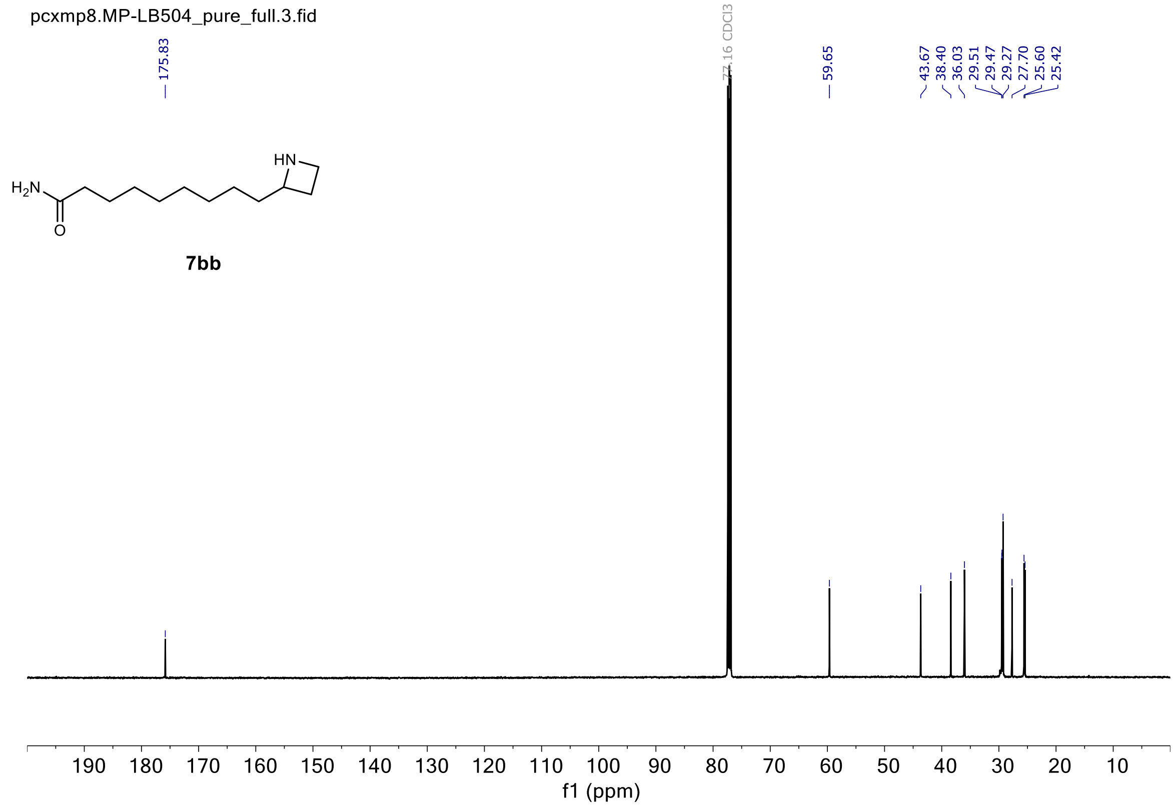

8-(azetidin-2-yl)octan-1-ol trifluoroacetic acid (7bc)

^{1}H-NMR (CD_3OD, 500 MHz)

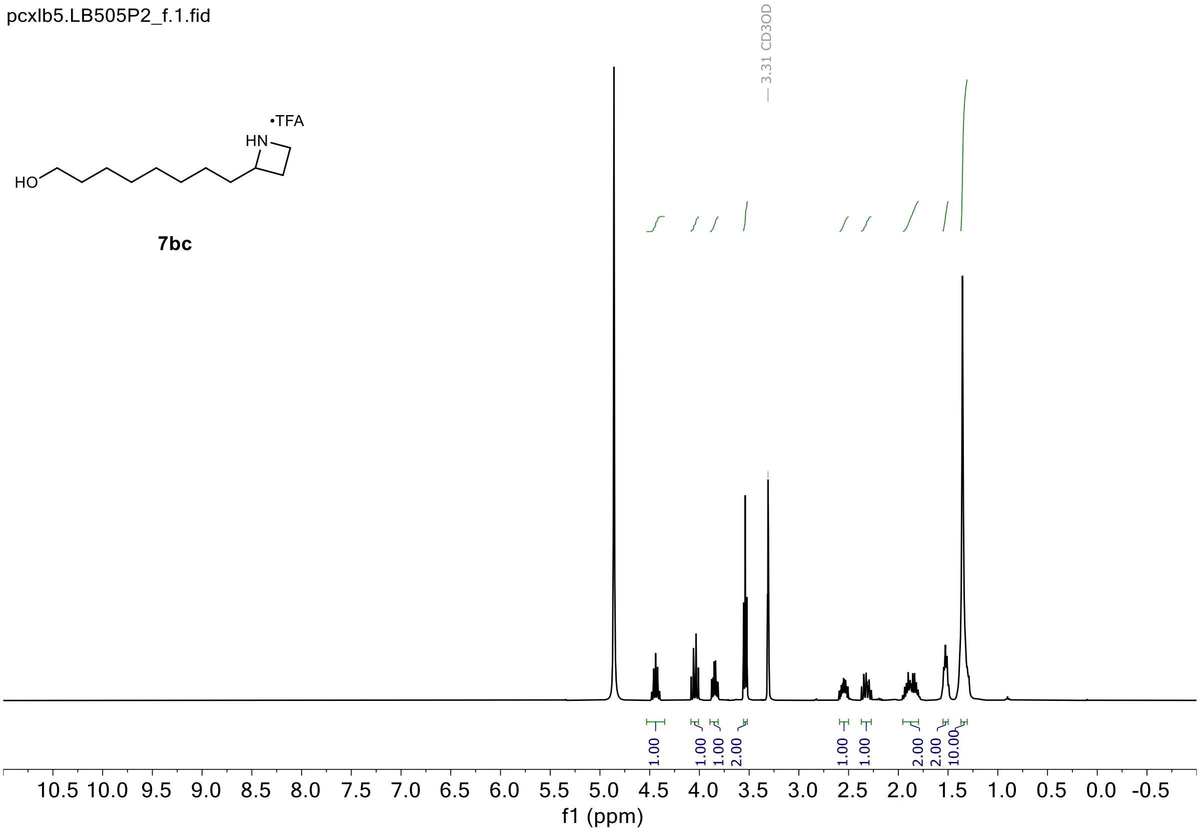

^{13}C-NMR (CD_3OD, 500 MHz)

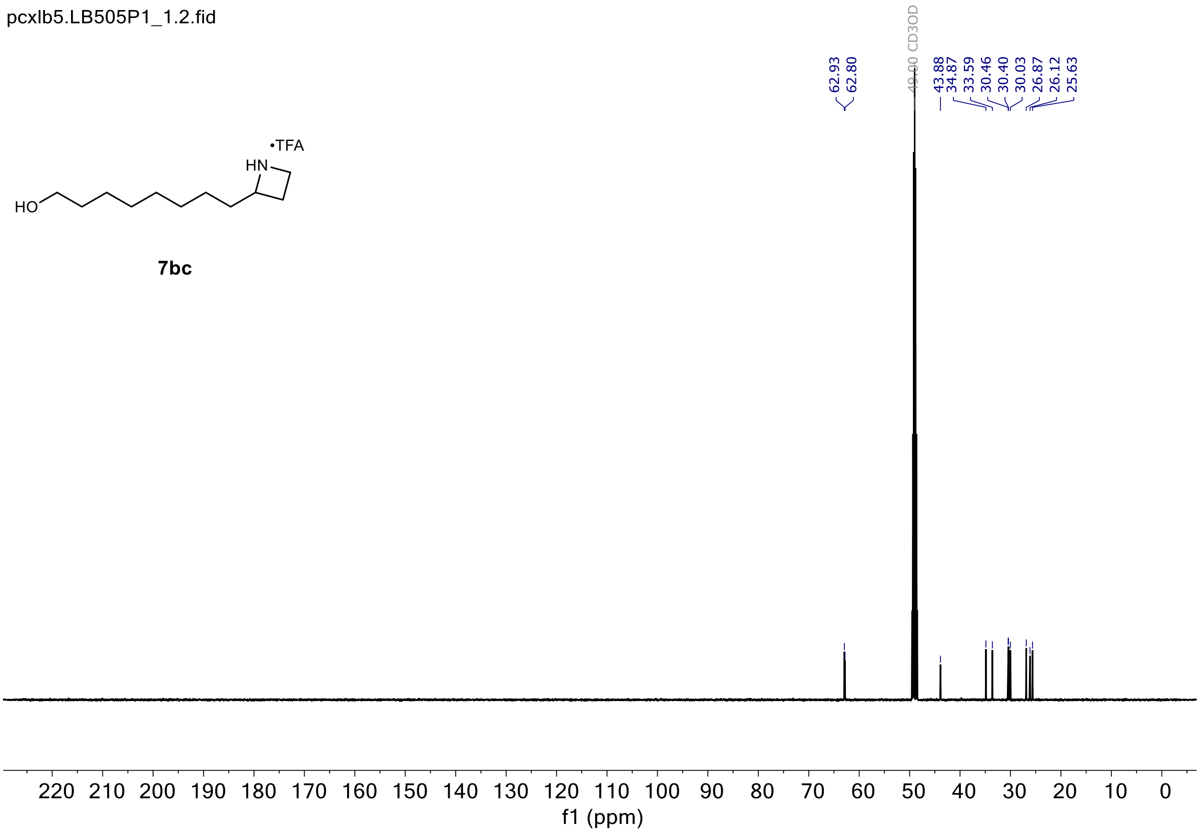

^{19}F-NMR (CD_3OD, 376 MHz)

pcxlb5.LB505P2_f.2.fid

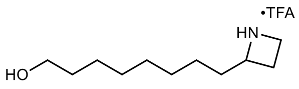

7bc

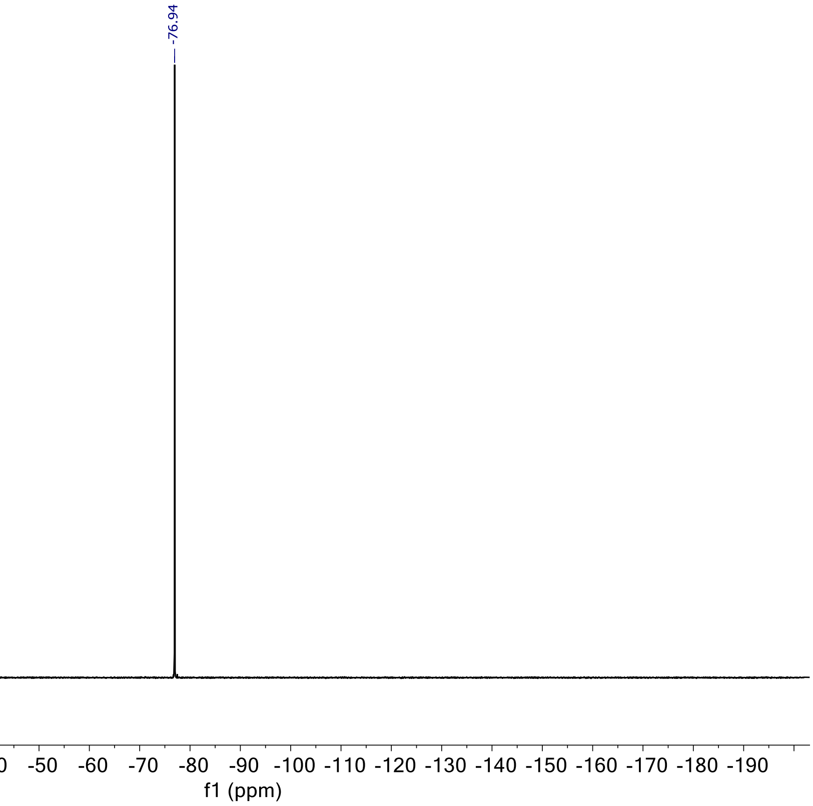

2-phenethyloxetane (8a)

<u>^{1}H-NMR ($CDCl_3$, 500 MHz)</u>

pcxmp8.MP277check_2C_pure_full.1.fid

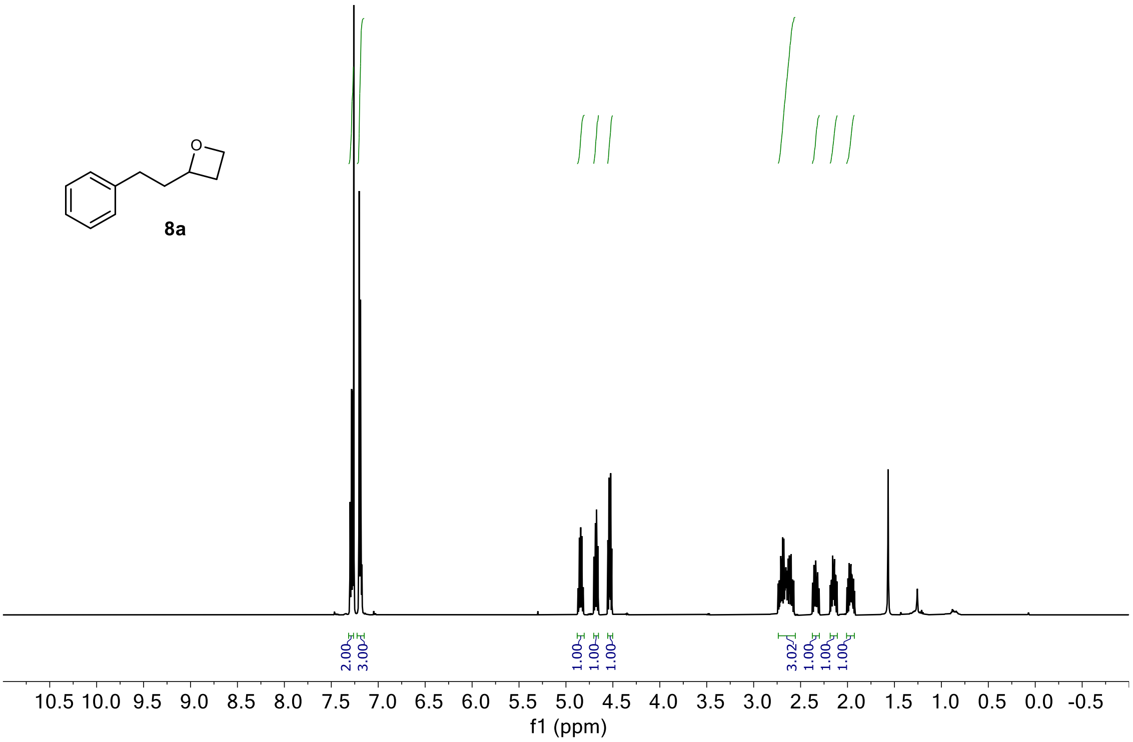

<u>^{13}C-NMR ($CDCl_3$, 126 MHz)</u>

pcxmp8.MP277check_2C_pure_full.2.fid

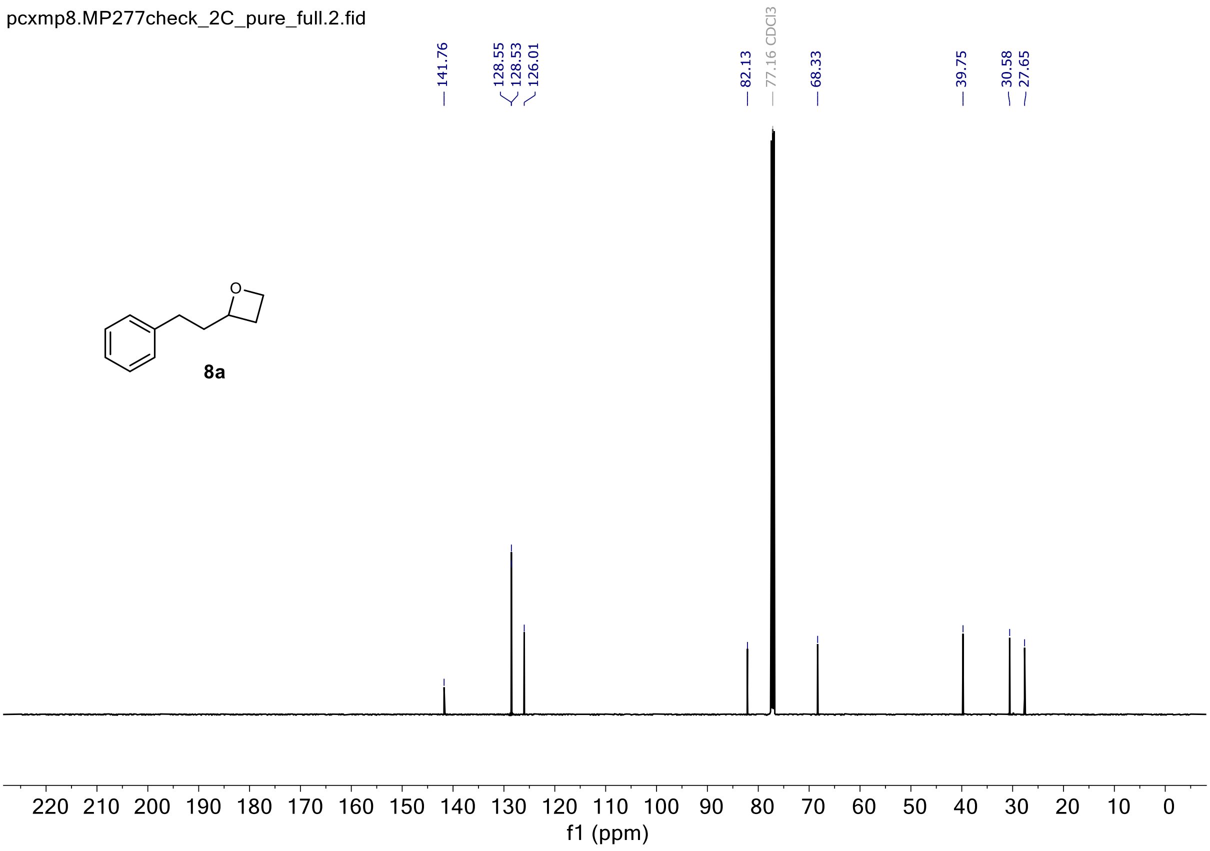

tert-butyl 9-(oxetan-2-yl)nonanoate (8b)

^{1}H-NMR ($CDCl_3$, 500 MHz)

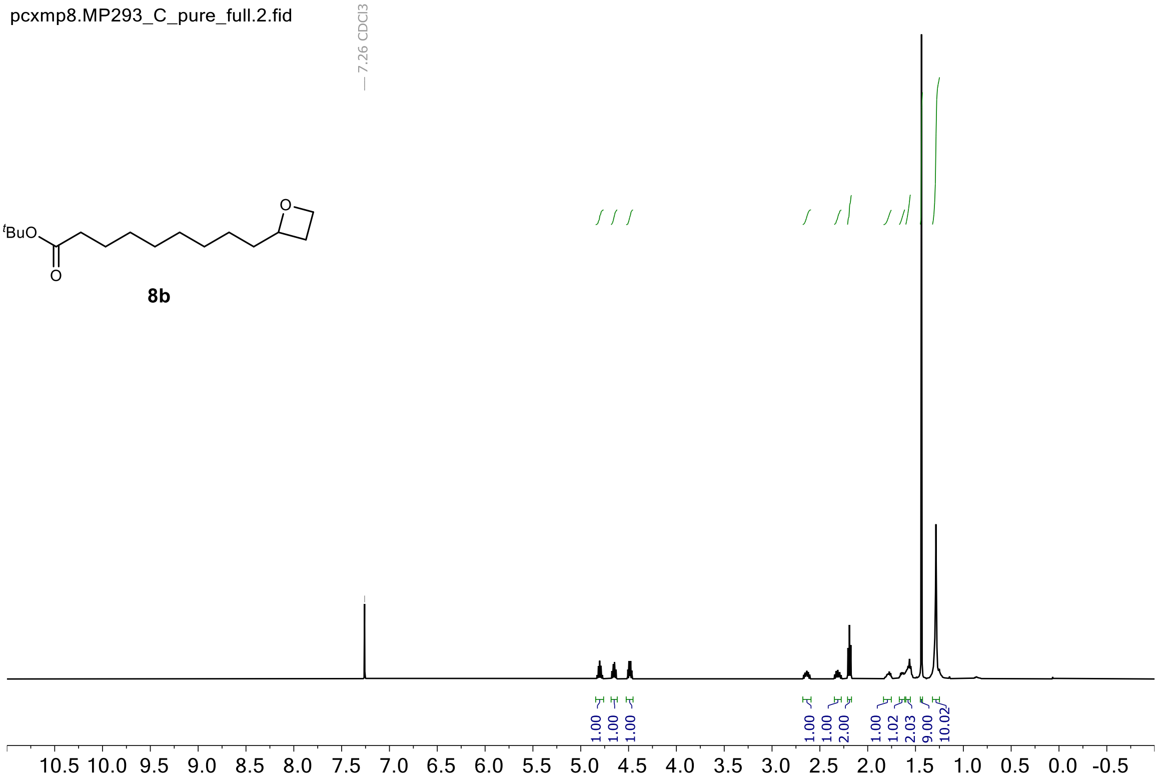

^{13}C-NMR ($CDCl_3$, 126 MHz)

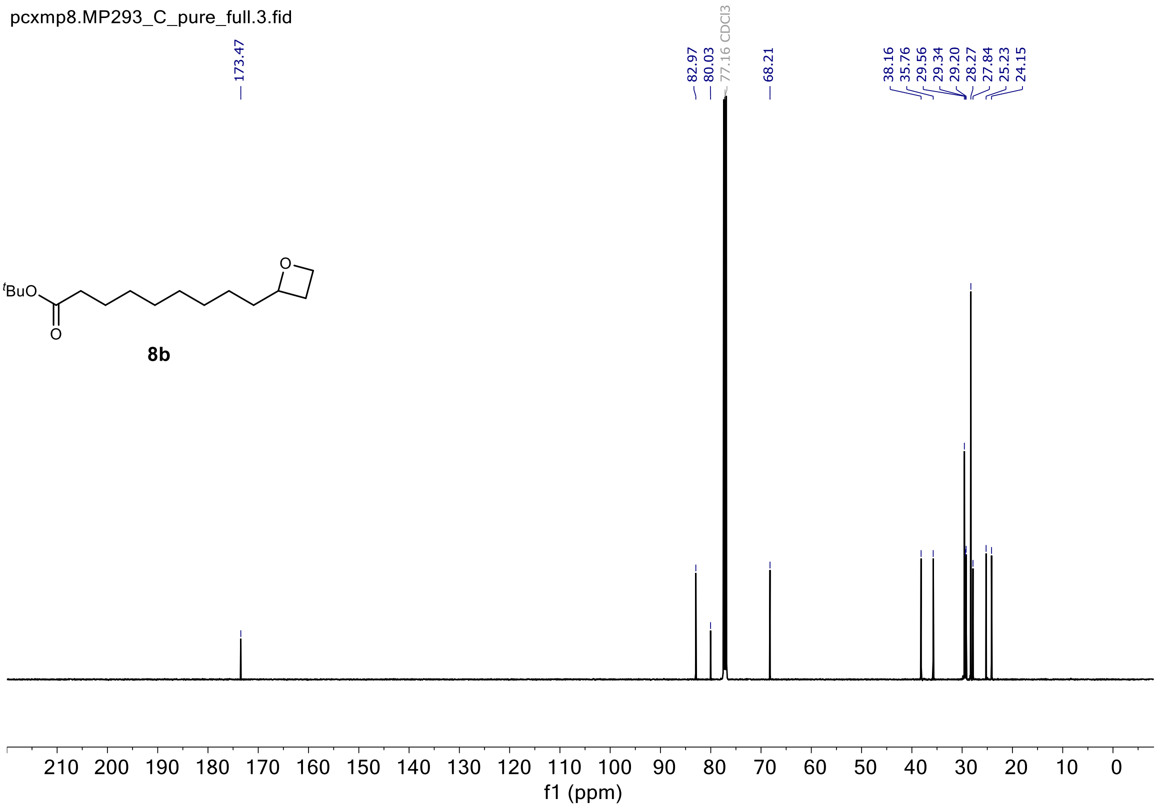

10-(oxetan-2-yl)decanenitrile (8c)

^{1}H-NMR ($CDCl_3$, 500 MHz)

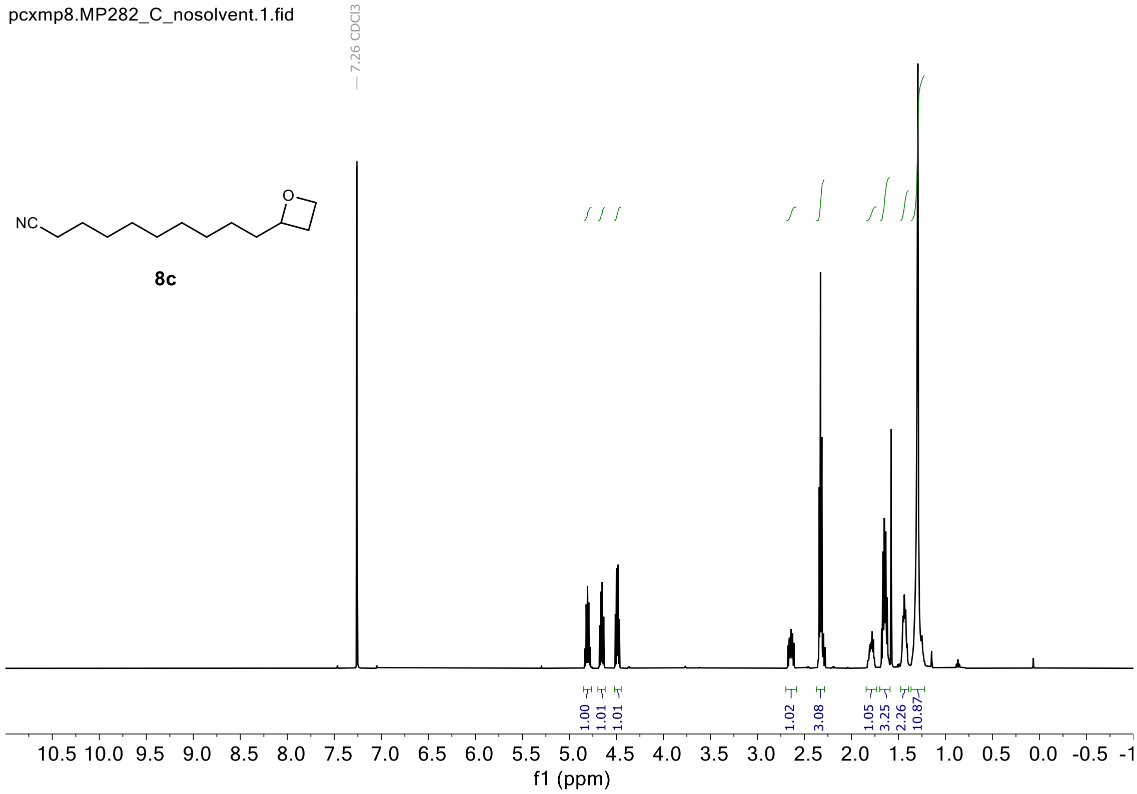

^{13}C-NMR ($CDCl_3$, 126 MHz)

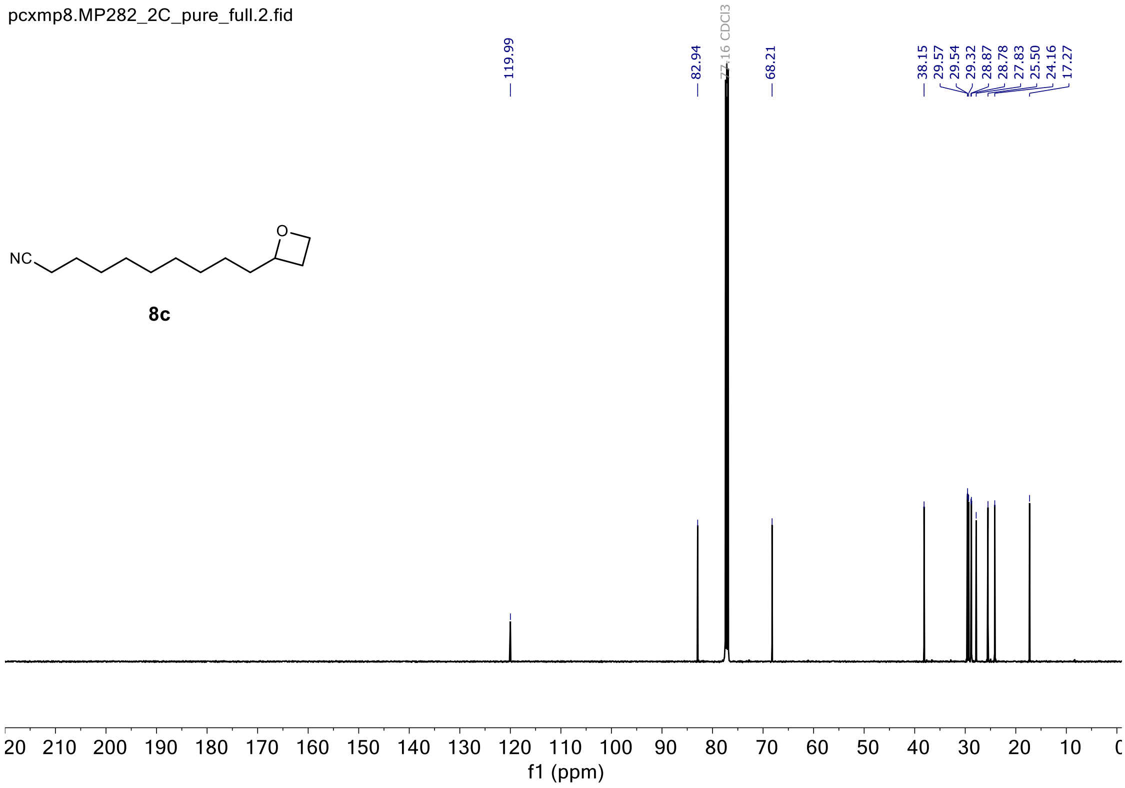

2-(4-(phenylsulfonyl)butyl)oxetane (8d)

^{1}H-NMR (CDCl$_3$, 500 MHz)

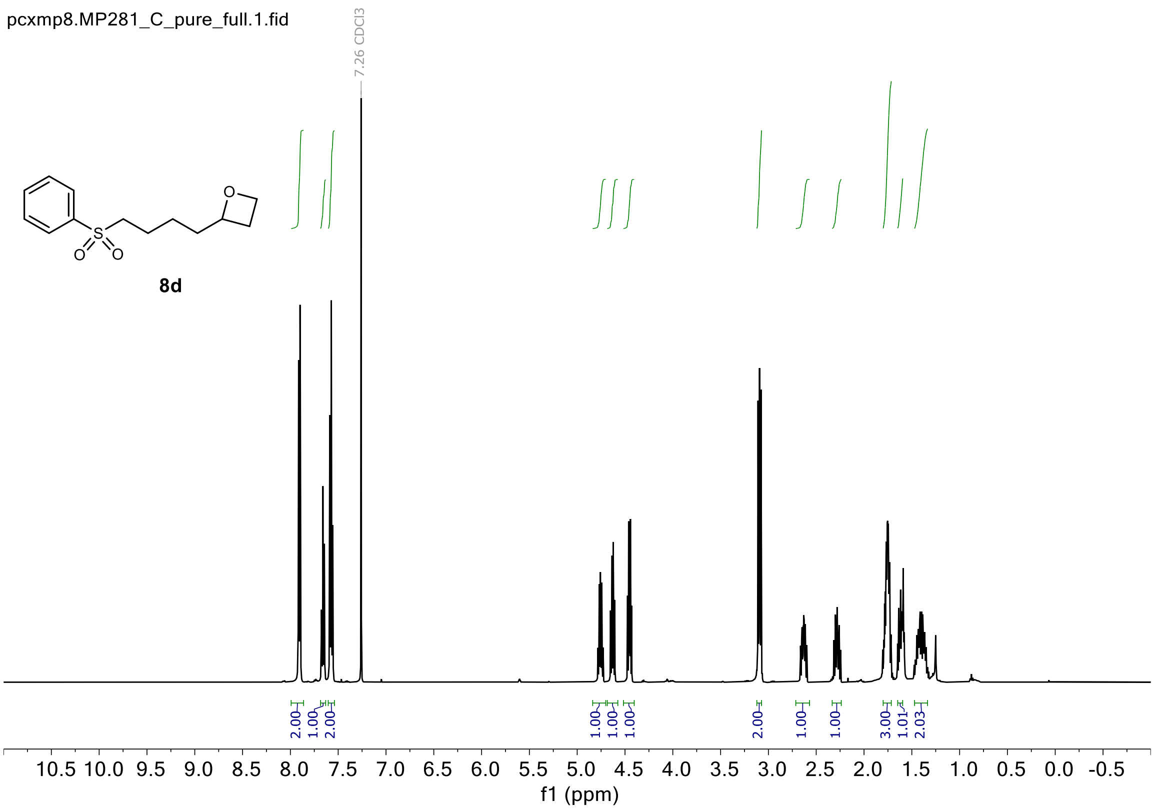

^{13}C-NMR (CDCl$_3$, 126 MHz)

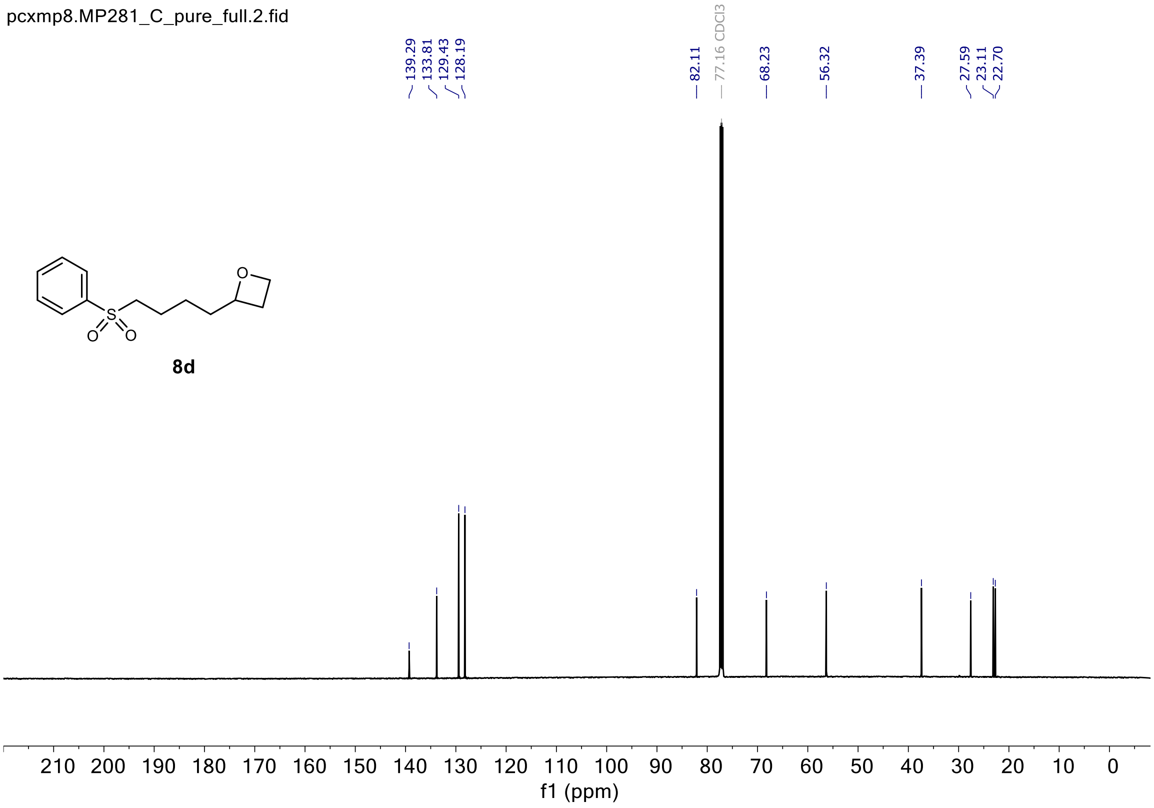

2-(4-(benzyloxy)butyl)oxetane (8e)

^{1}H-NMR ($CDCl_3$, 500 MHz)

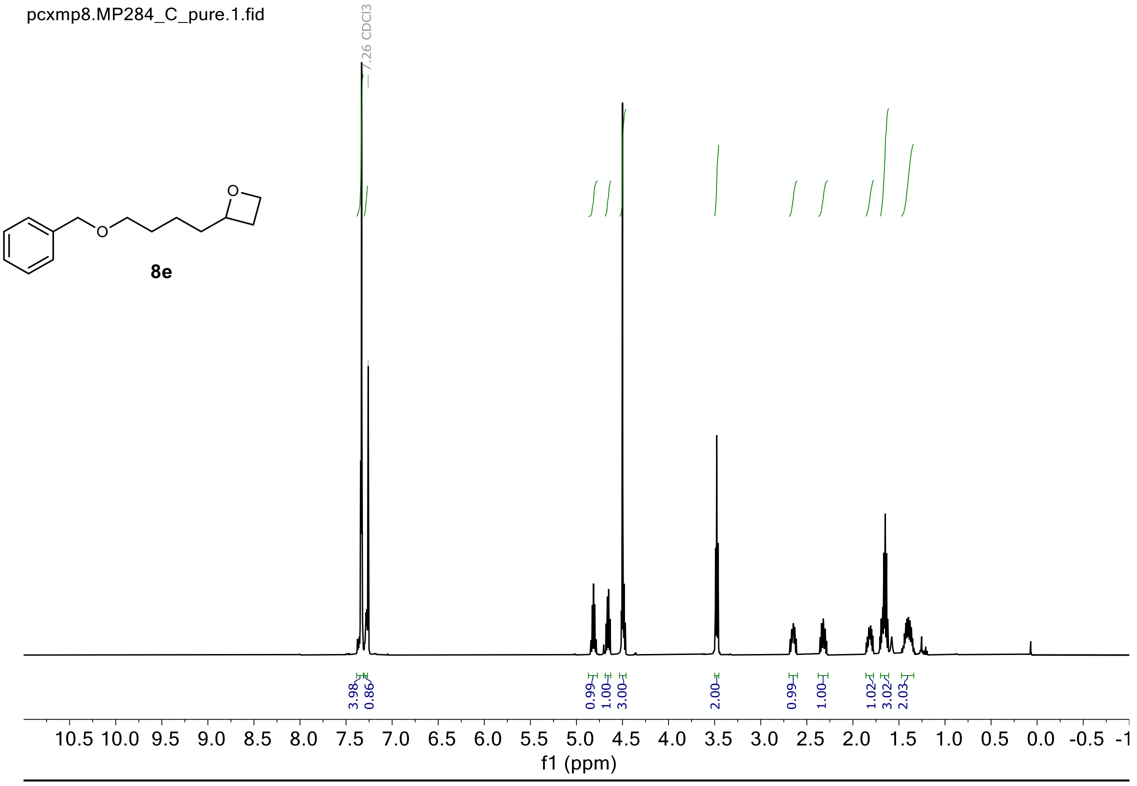

^{13}C-NMR ($CDCl_3$, 126 MHz)

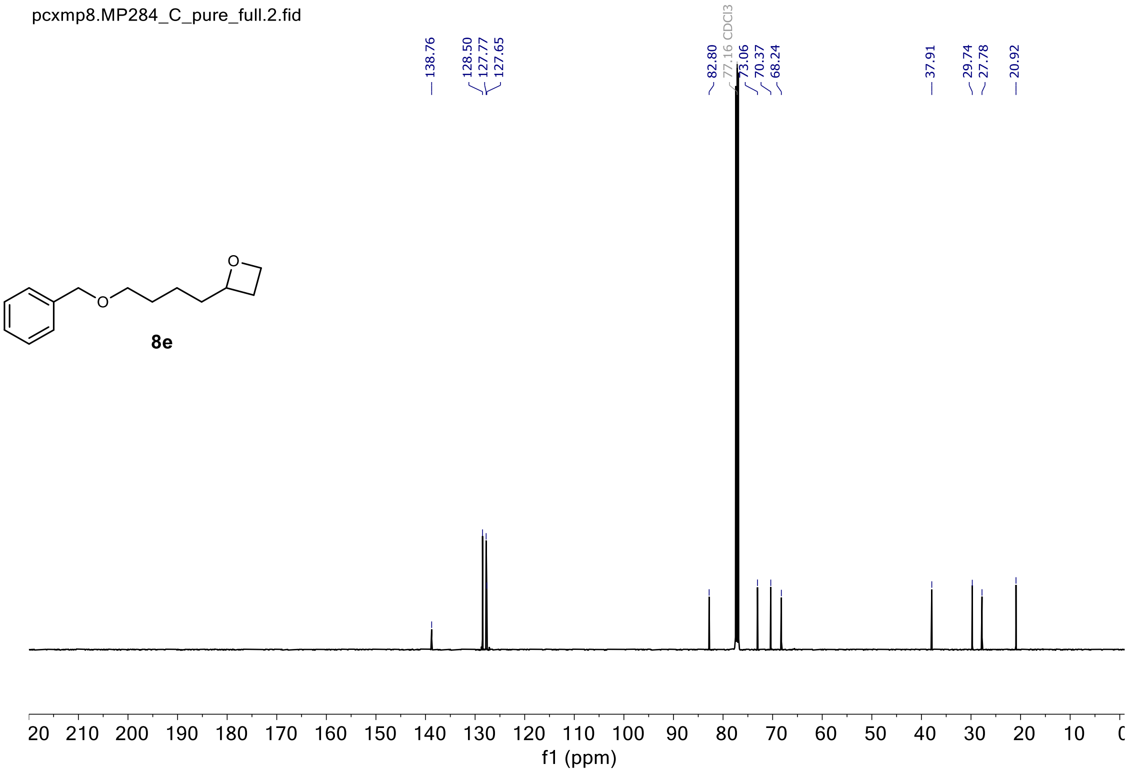

N-(4-(oxetan-2-yl)butyl)-4-(5-(p-tolyl)-3-(trifluoromethyl)-1H-pyrazol-1-yl) benzenesulfonamide (8f)

^{1}H-NMR (CDCl$_3$, 500 MHz)

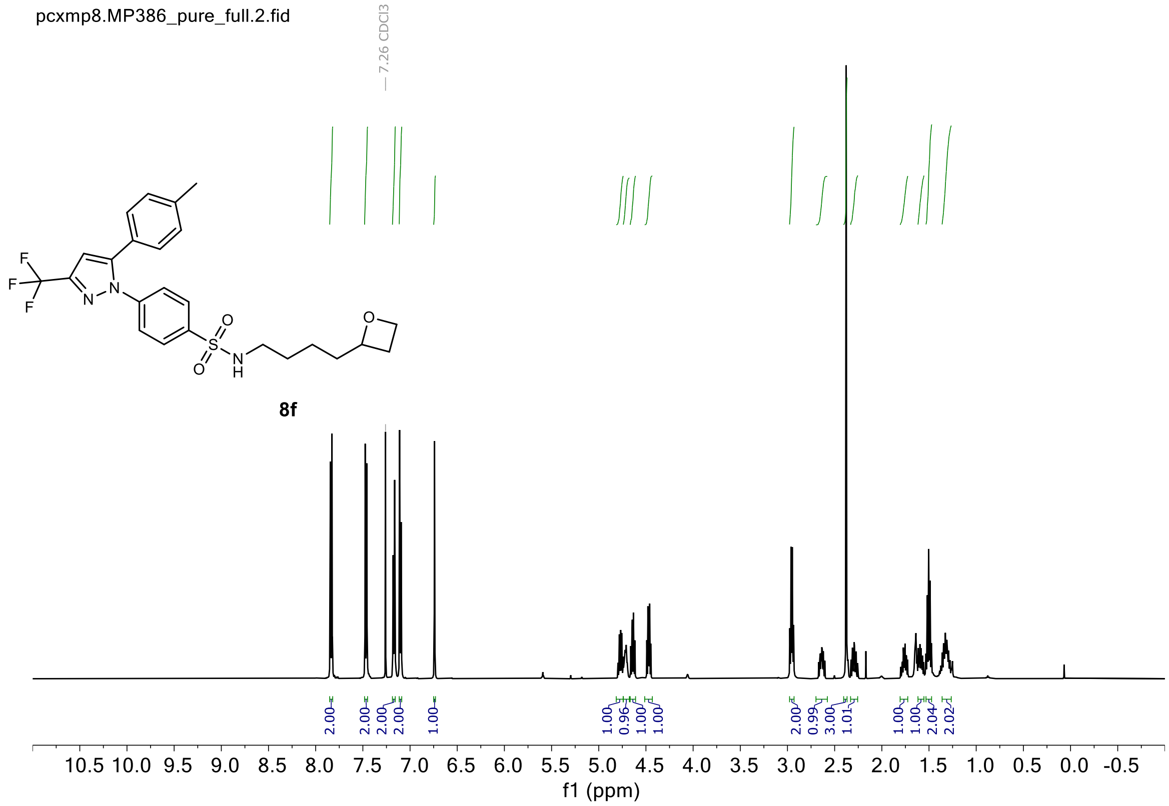

^{13}C-NMR (CDCl$_3$, 126 MHz)

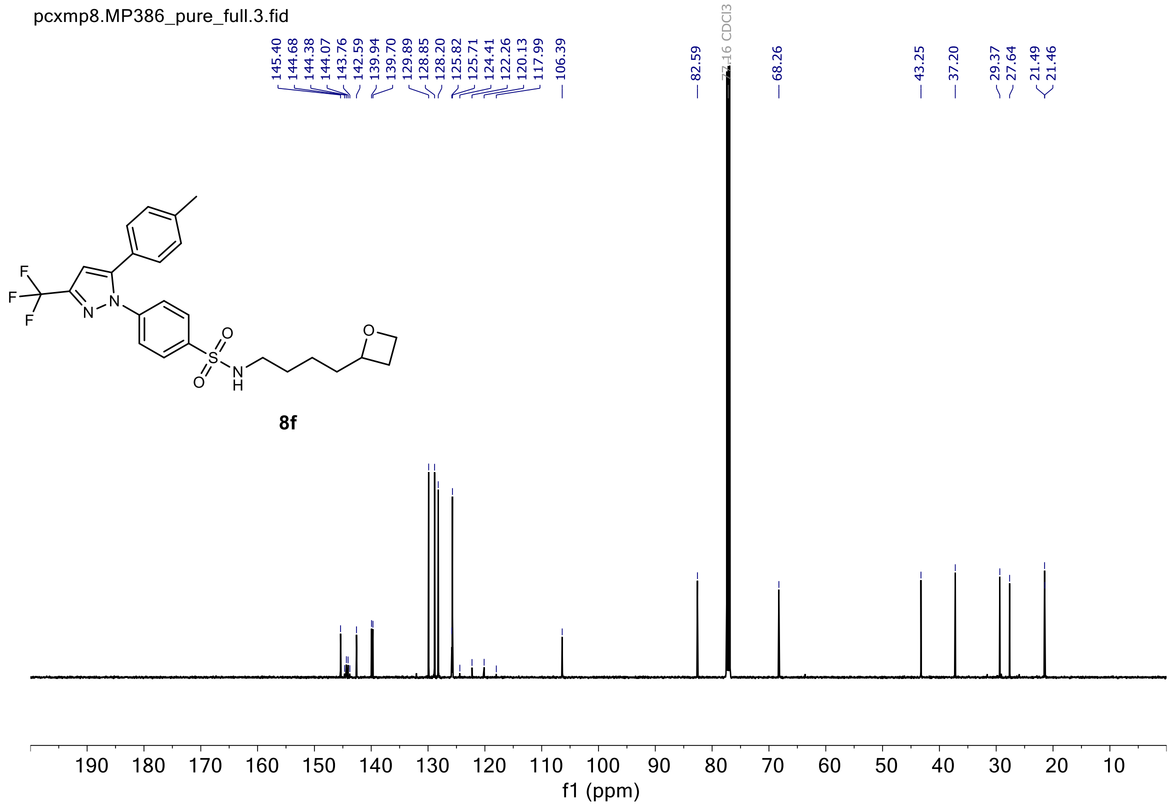

19F-NMR (CDCl3, 376 MHz)

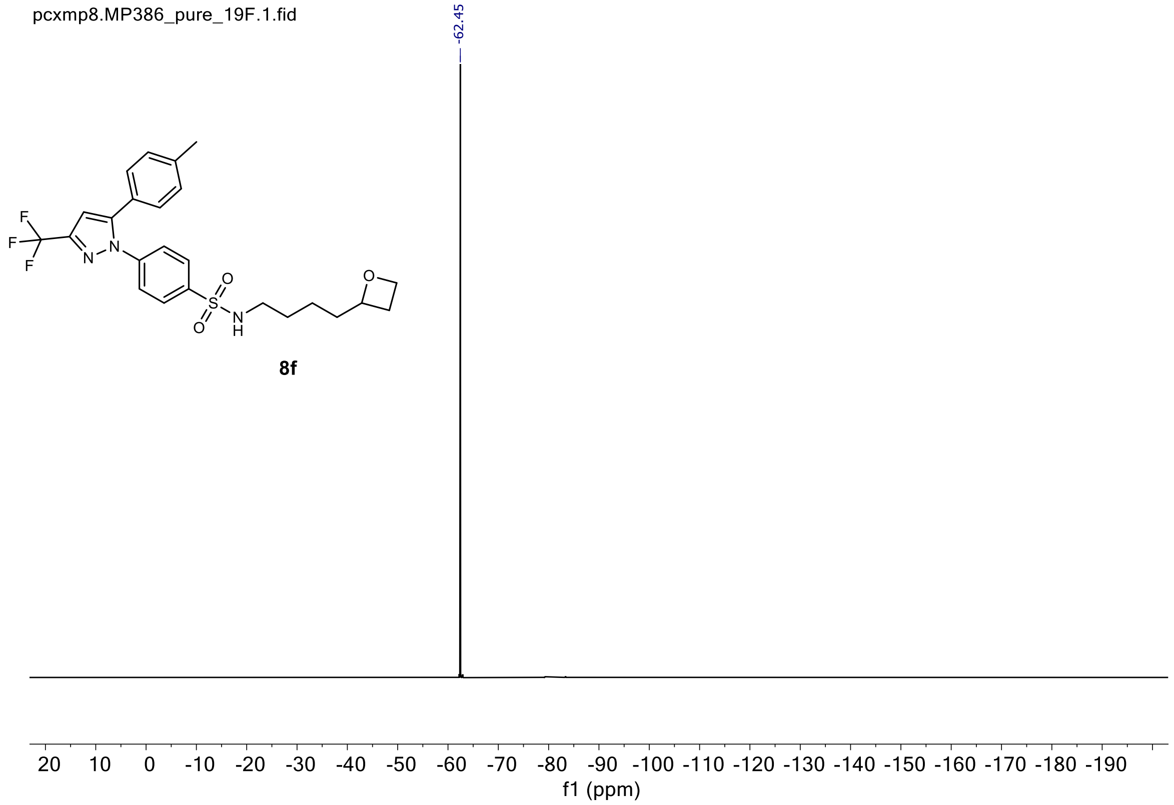

2-phenethylthietane (9a)

^{1}H-NMR ($CDCl_3$, 500 MHz)

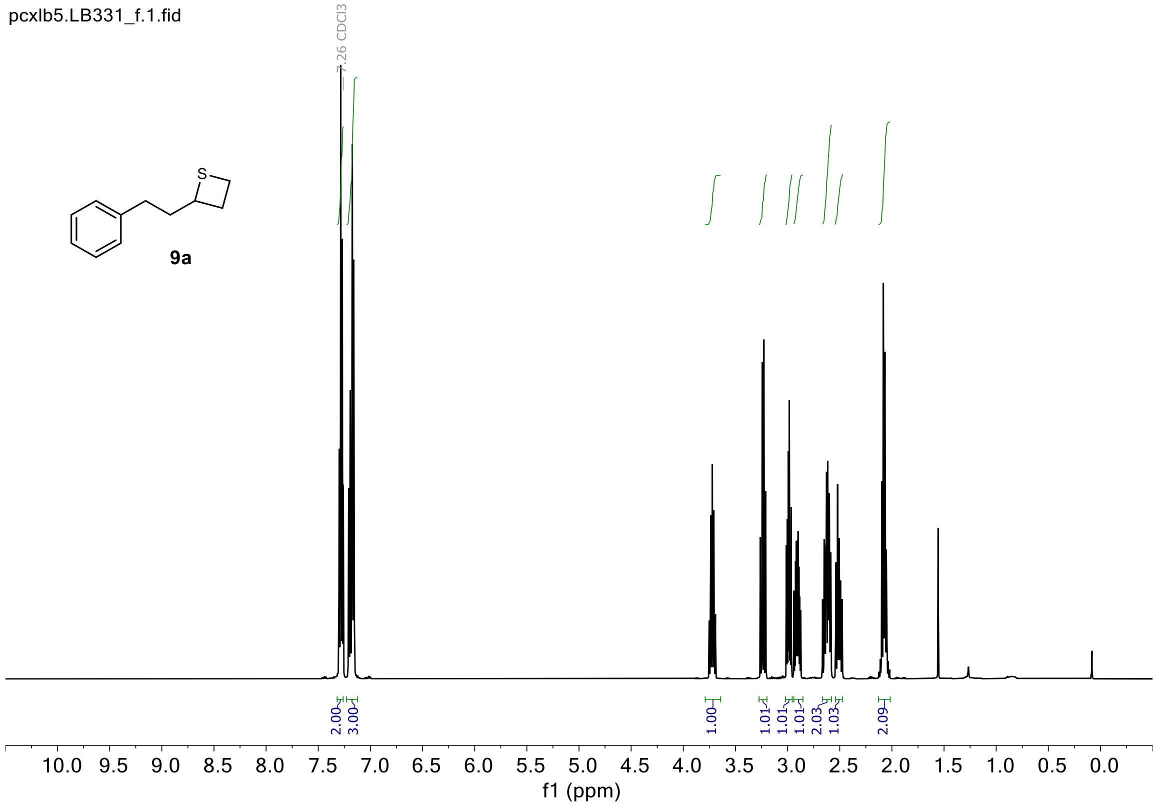

^{13}C-NMR ($CDCl_3$, 126 MHz)

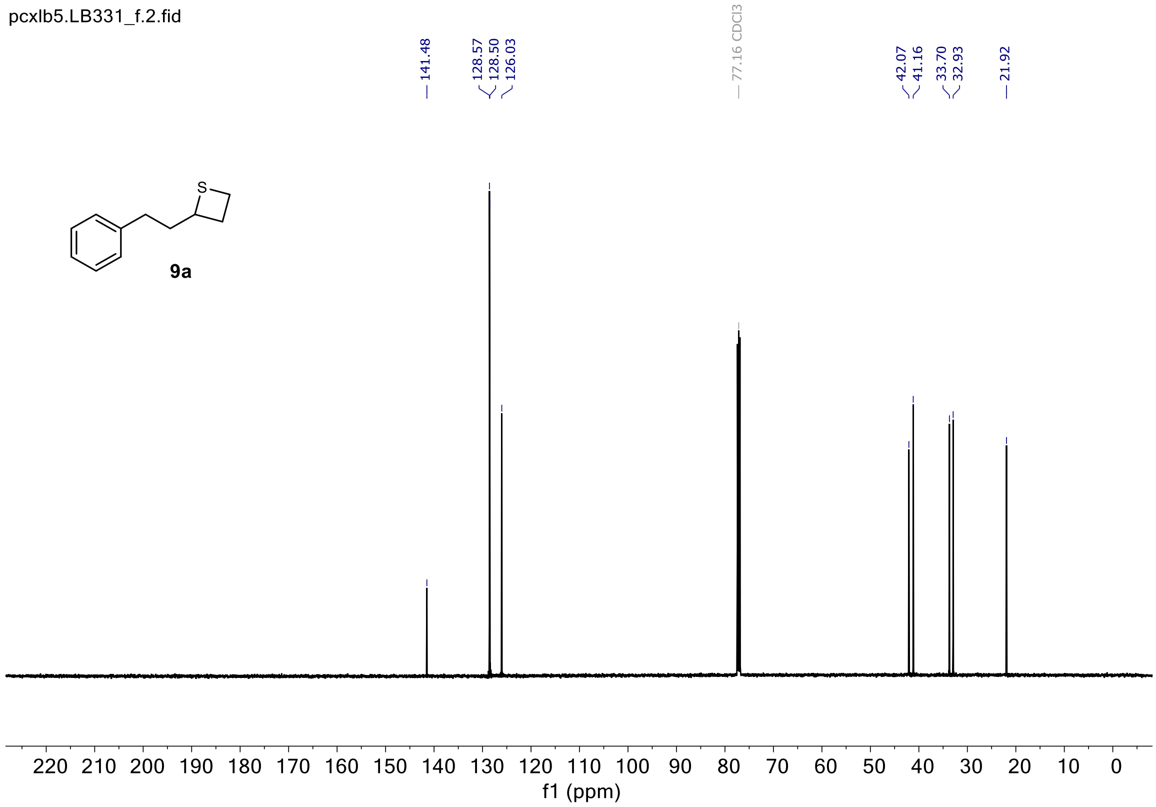

methyl 9-(thietan-2-yl)nonanoate (9b)

^{1}H-NMR (CDCl$_3$, 500 MHz)

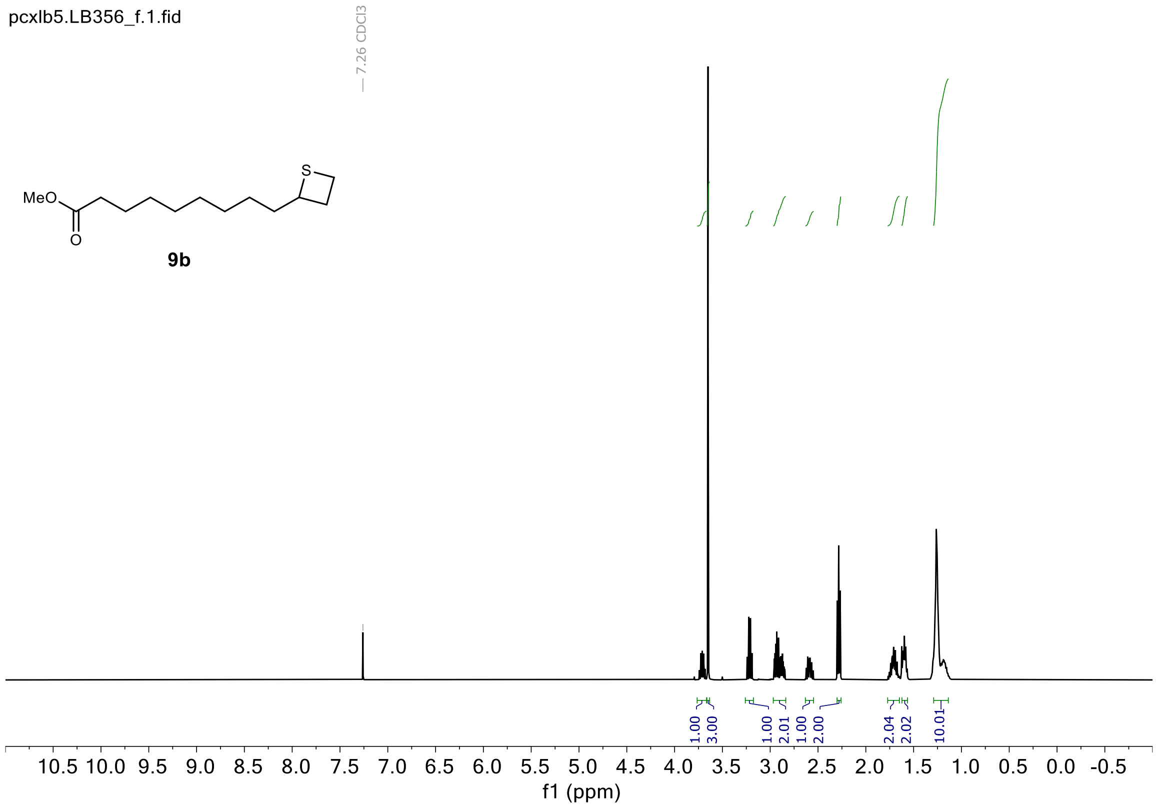

^{13}C-NMR (CDCl$_3$, 126 MHz)

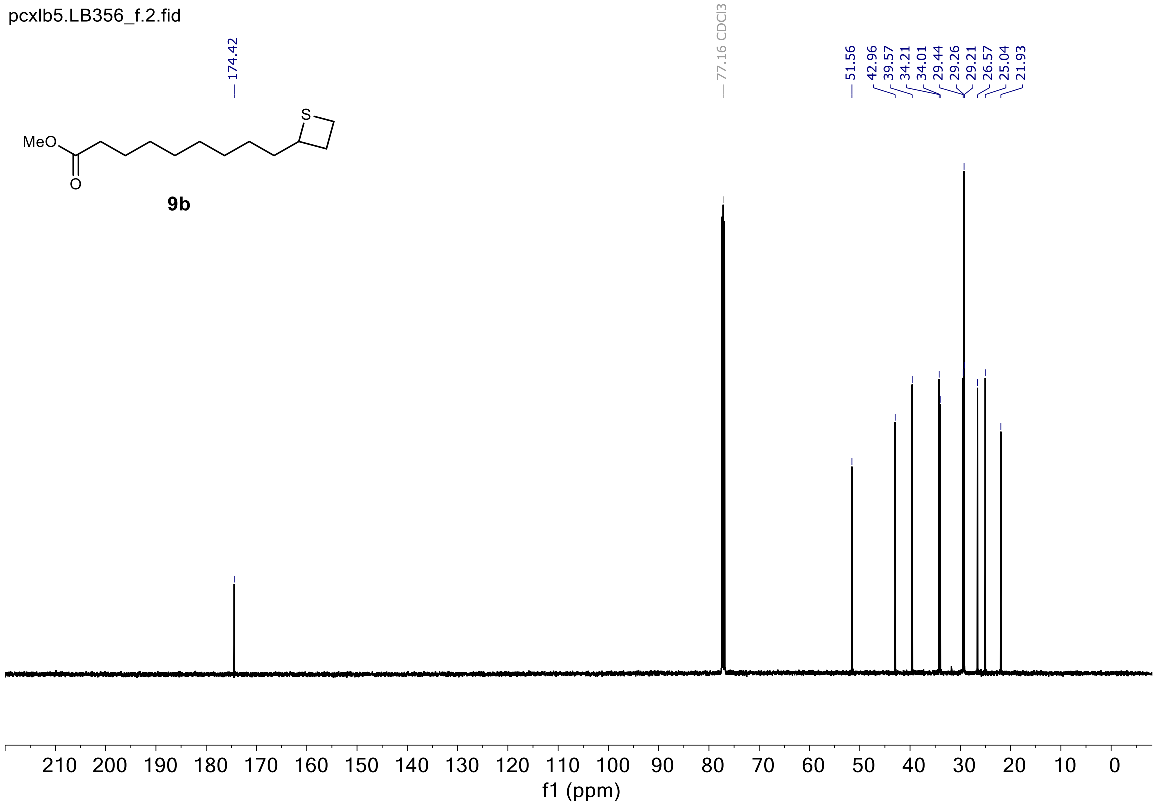

10-(thietan-2-yl)decanenitrile (9c)

^{1}H-NMR ($CDCl_3$, 500 MHz)

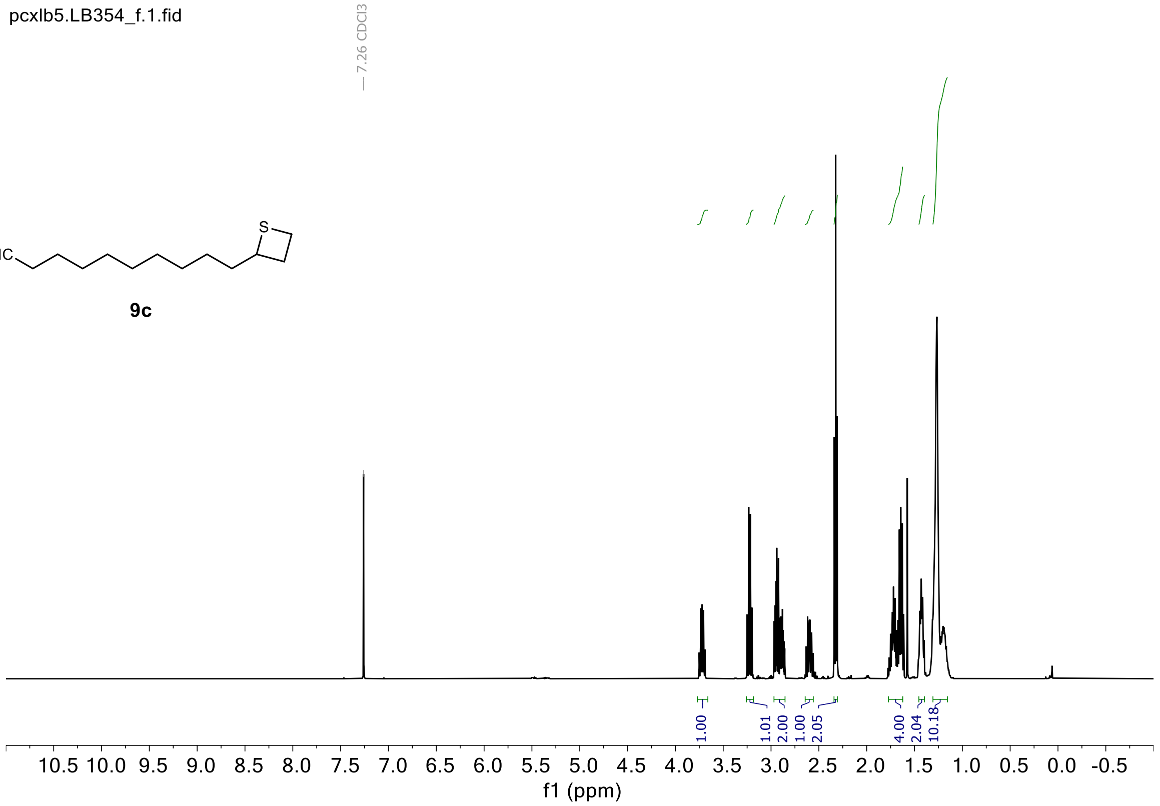

^{13}C-NMR ($CDCl_3$, 126 MHz)

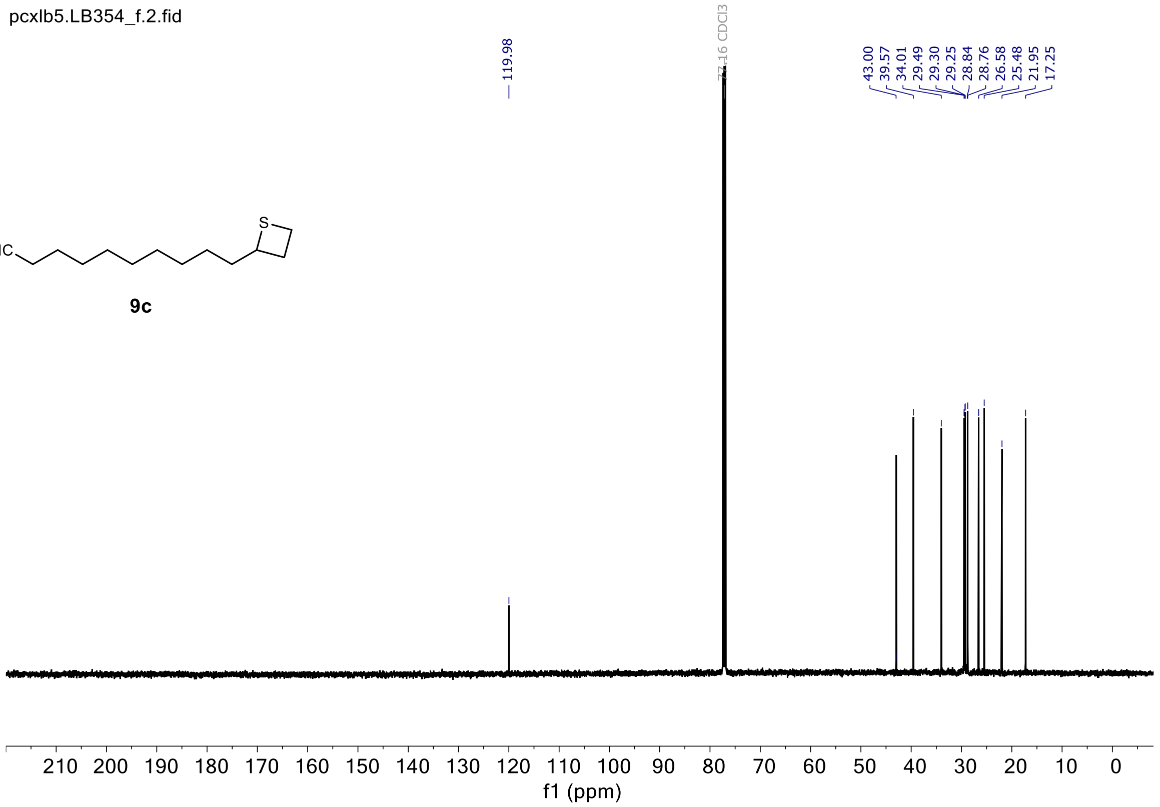

2-(4-(phenylsulfonyl)butyl)thietane (9d)

^{1}H-NMR ($CDCl_3$, 500 MHz)

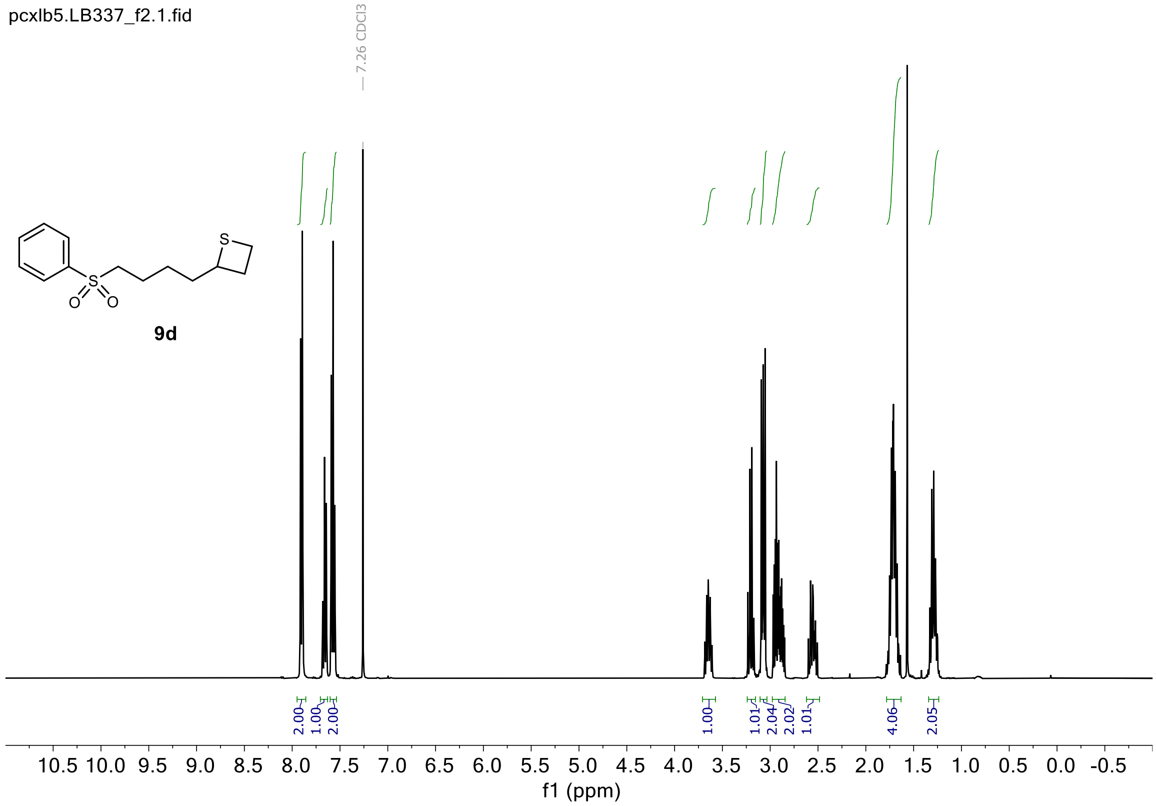

^{13}C-NMR ($CDCl_3$, 126 MHz)

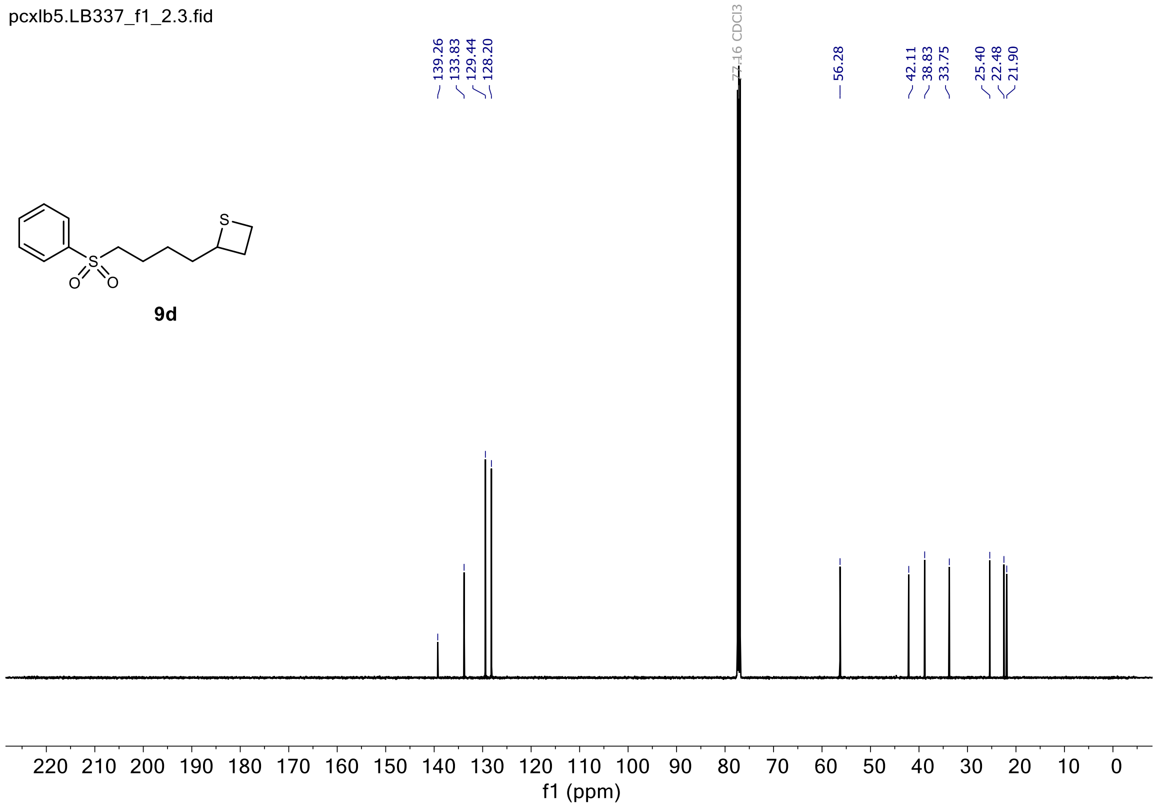

2-(4-(benzyloxy)butyl)thietane (9e)

^{1}H-NMR (CDCl$_3$, 500 MHz)

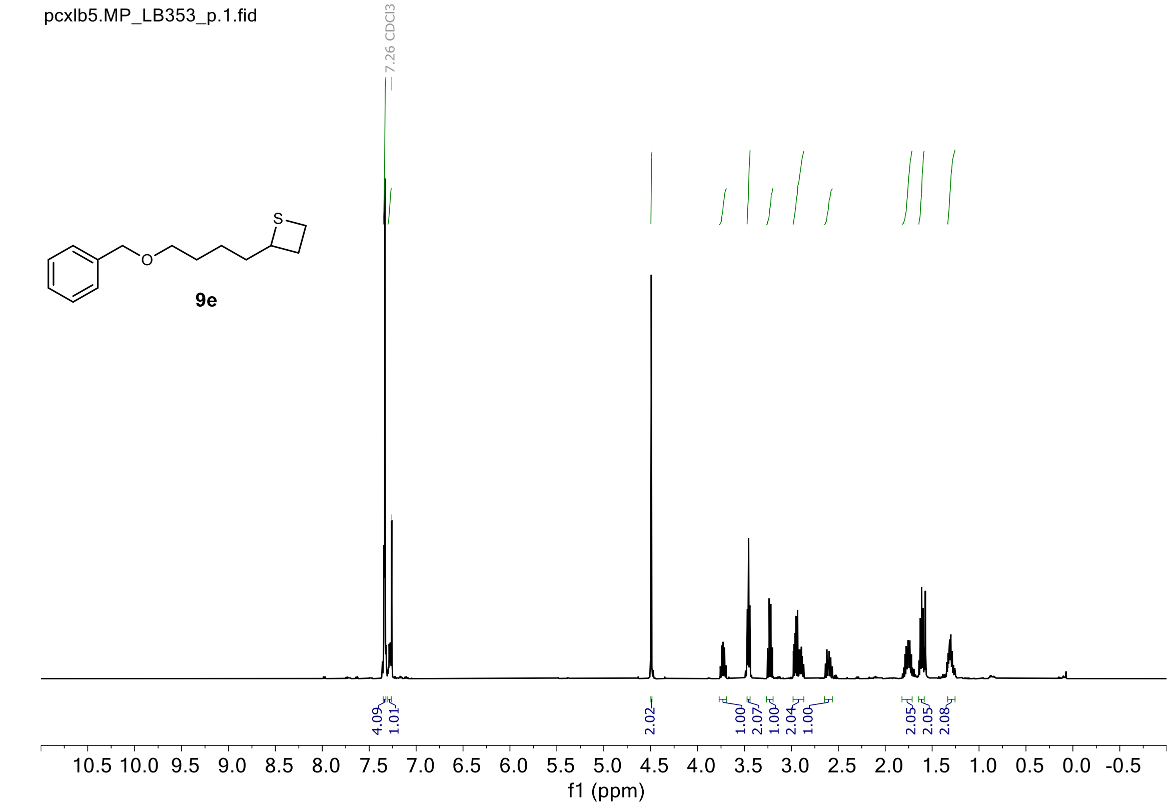

^{13}C-NMR (CDCl$_3$, 126 MHz)

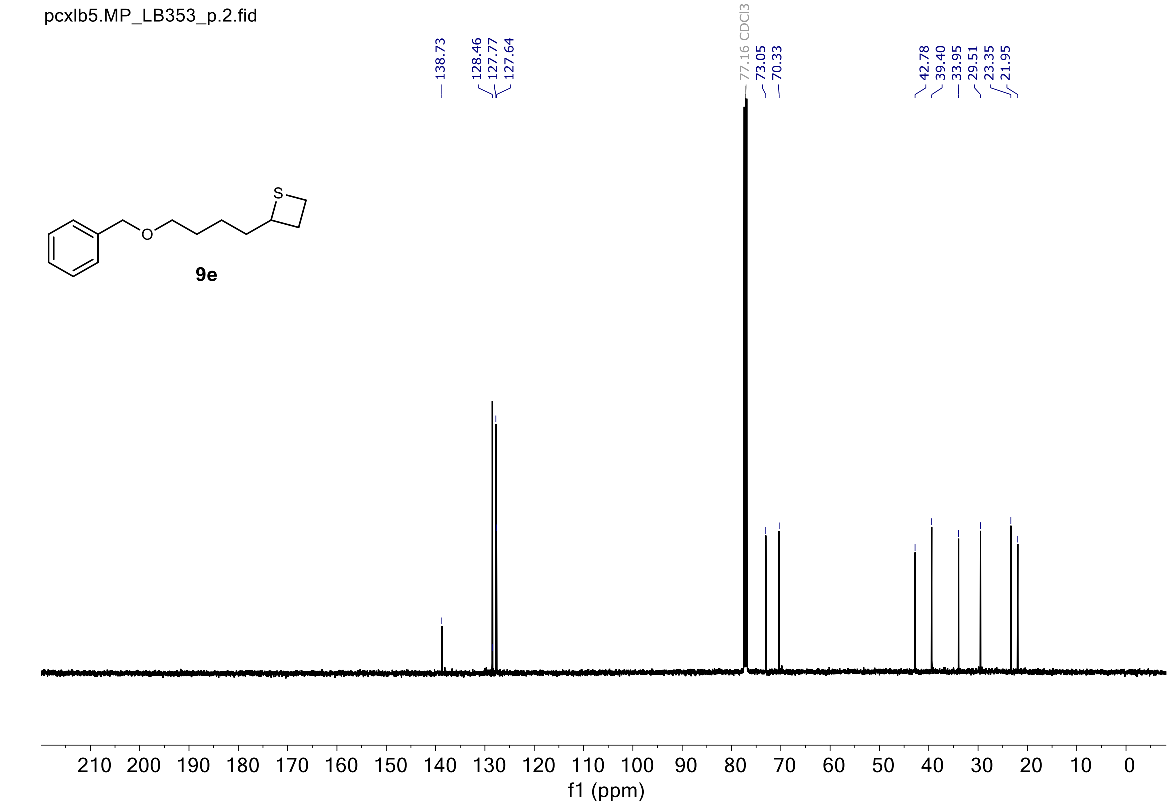

diethyl 2-phenethylcyclobutane-1,1-dicarboxylate (10a)

^{1}H-NMR ($CDCl_3$, 500 MHz)

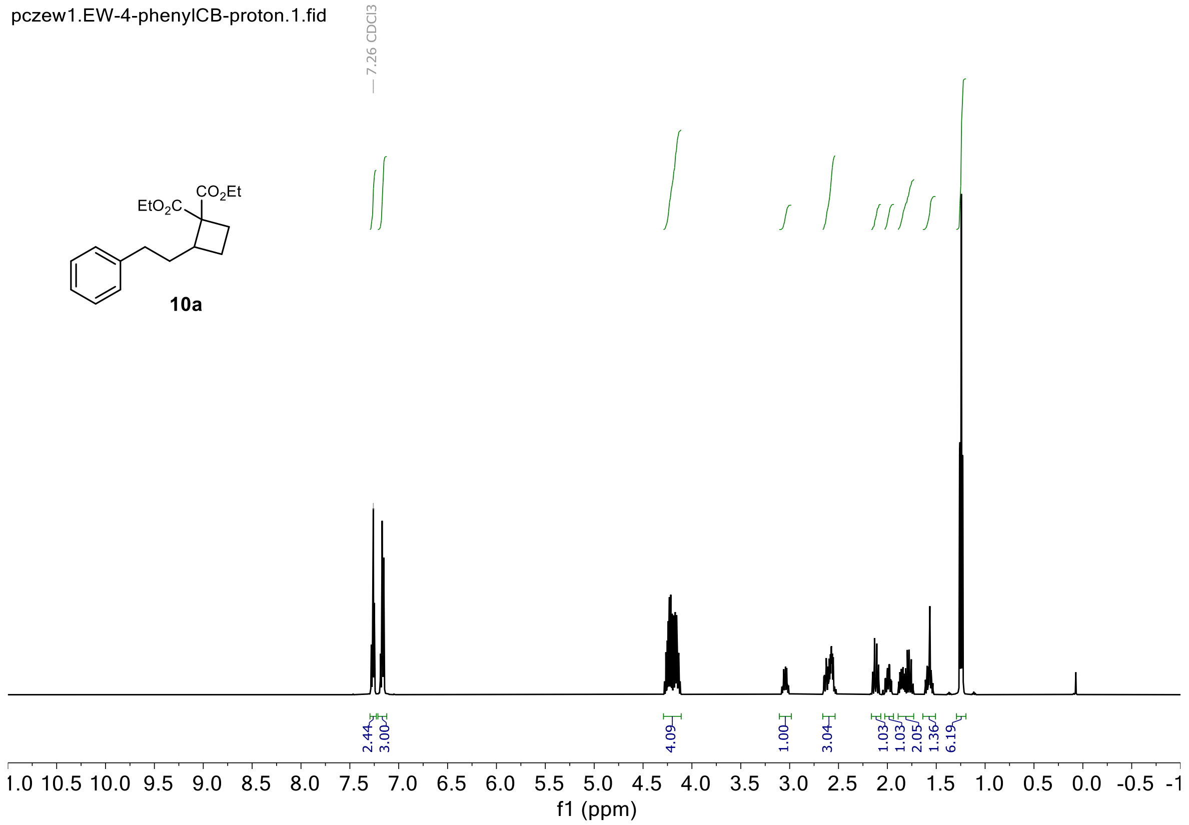

^{13}C-NMR ($CDCl_3$, 126 MHz)

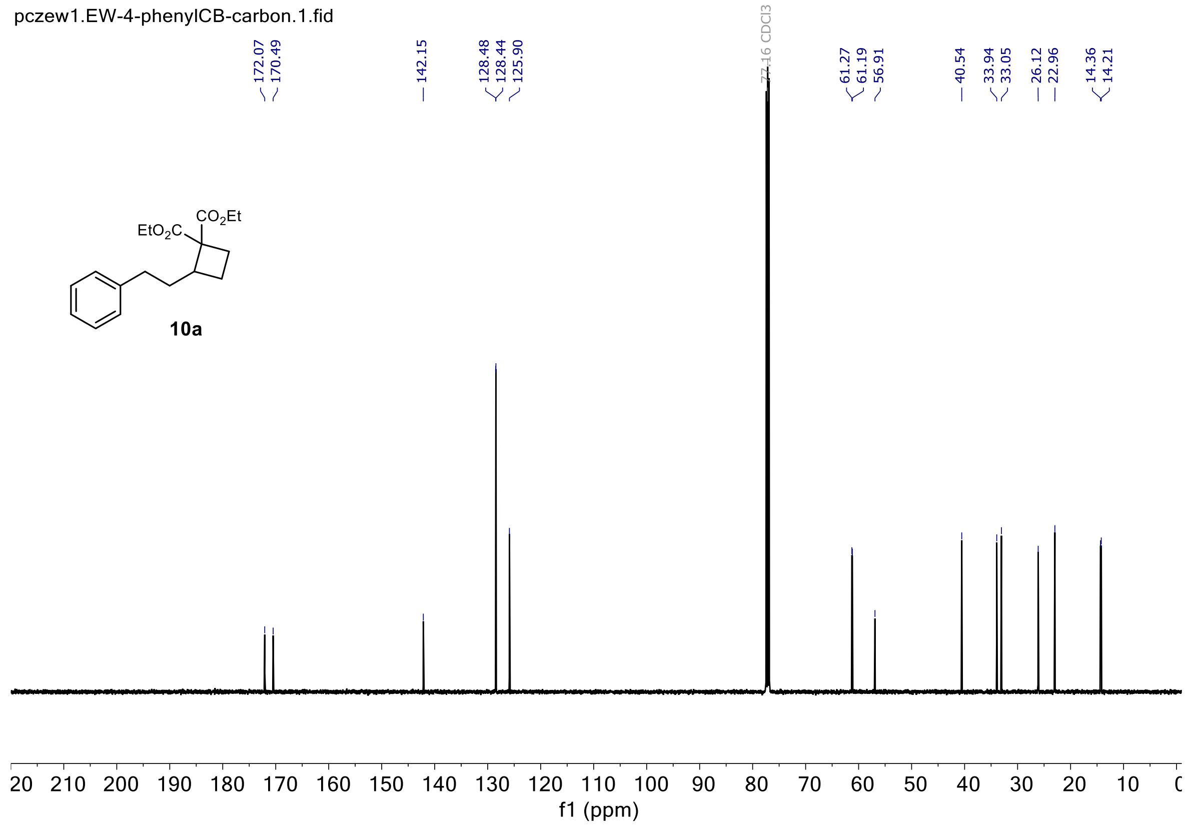

diethyl 2-(9-(tert-butoxy)-9-oxononyl)cyclobutane-1,1-dicarboxylate (10b)

^{1}H-NMR ($CDCl_3$, 500 MHz)

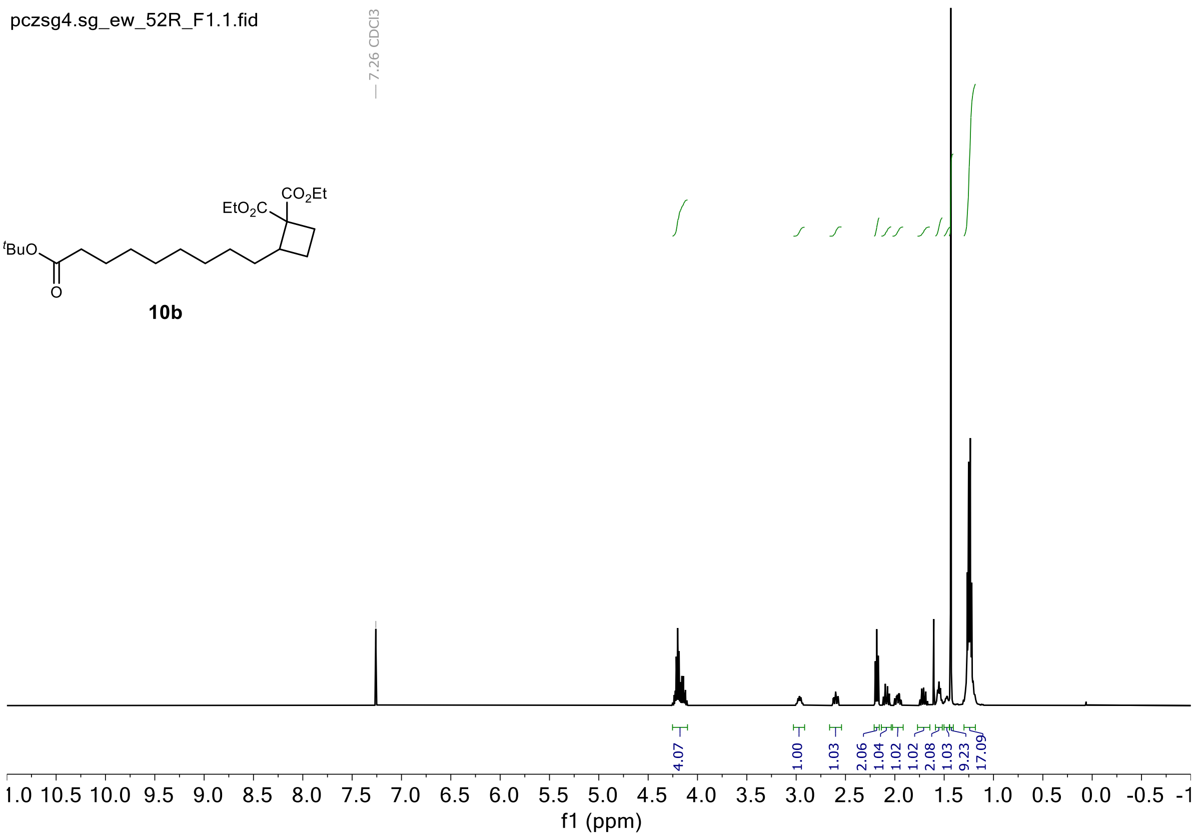

^{13}C-NMR ($CDCl_3$, 126 MHz)

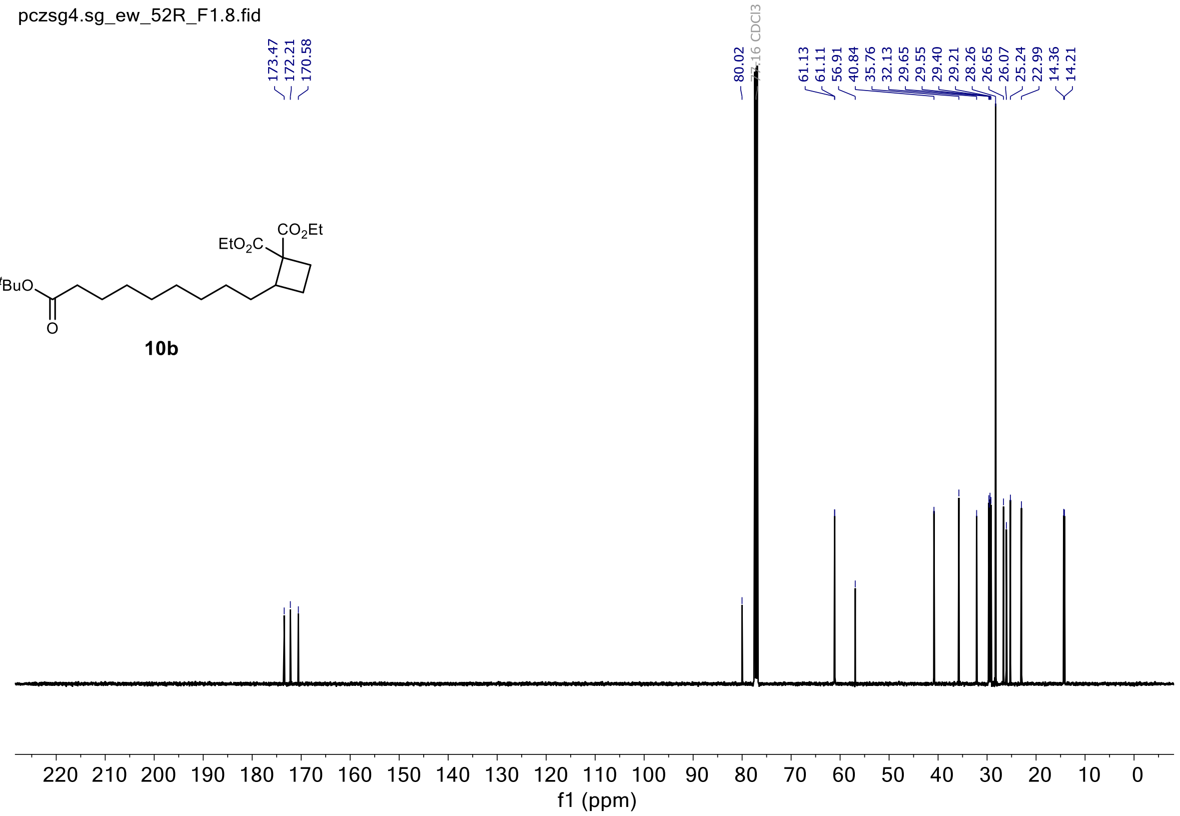

diethyl 2-(9-cyanononyl)cyclobutane-1,1-dicarboxylate (10c)

^{1}H-NMR ($CDCl_3$, 500 MHz)

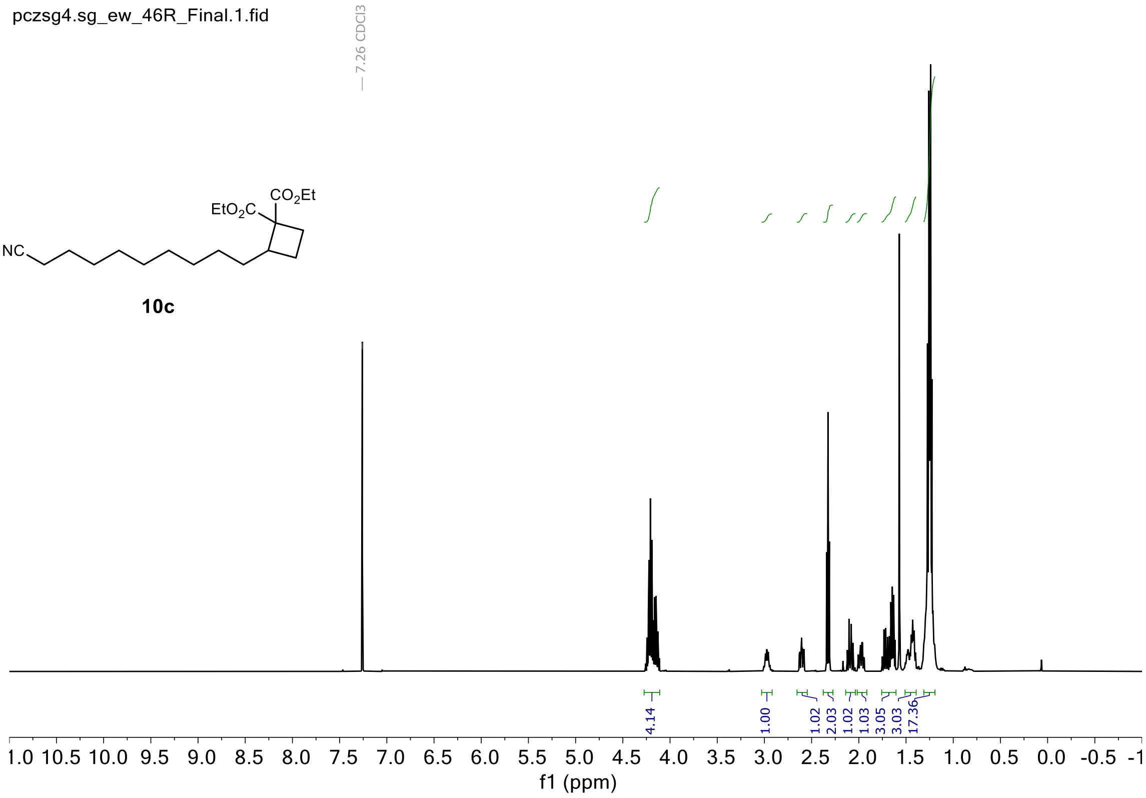

^{13}C-NMR ($CDCl_3$, 126 MHz)

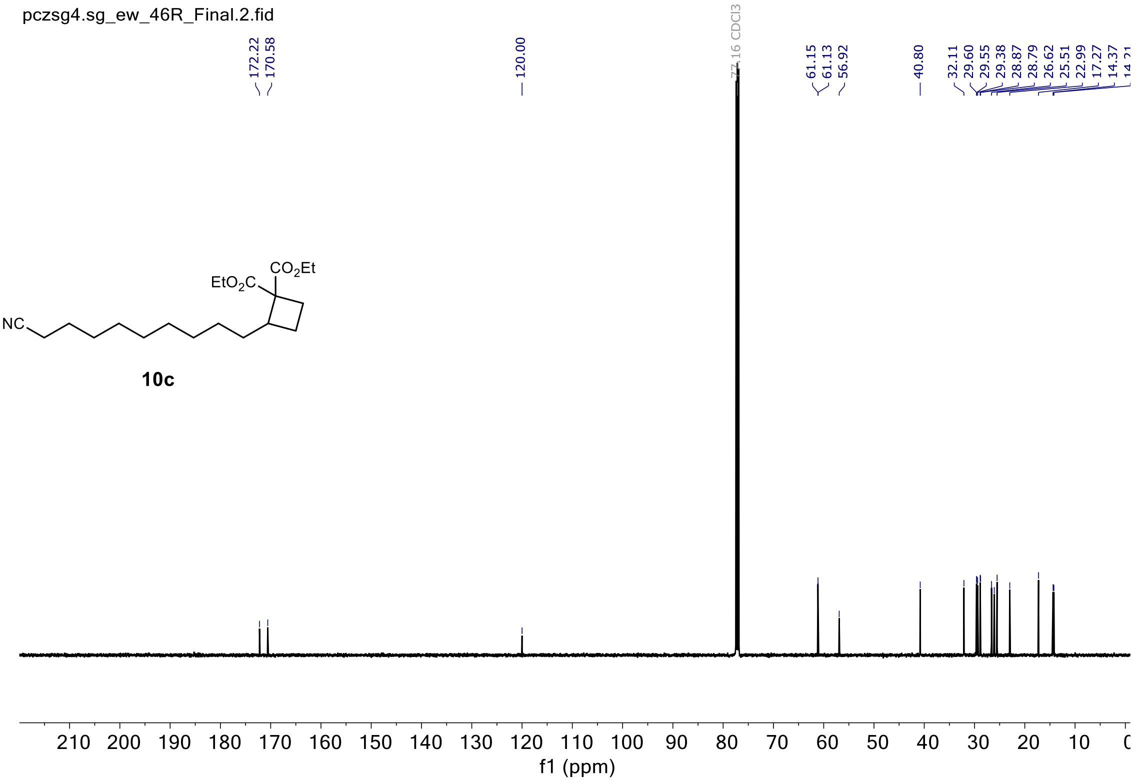

diethyl 2-(4-(phenylsulfonyl)butyl)cyclobutane-1,1-dicarboxylate (10d)

^{1}H-NMR ($CDCl_3$, 500 MHz)

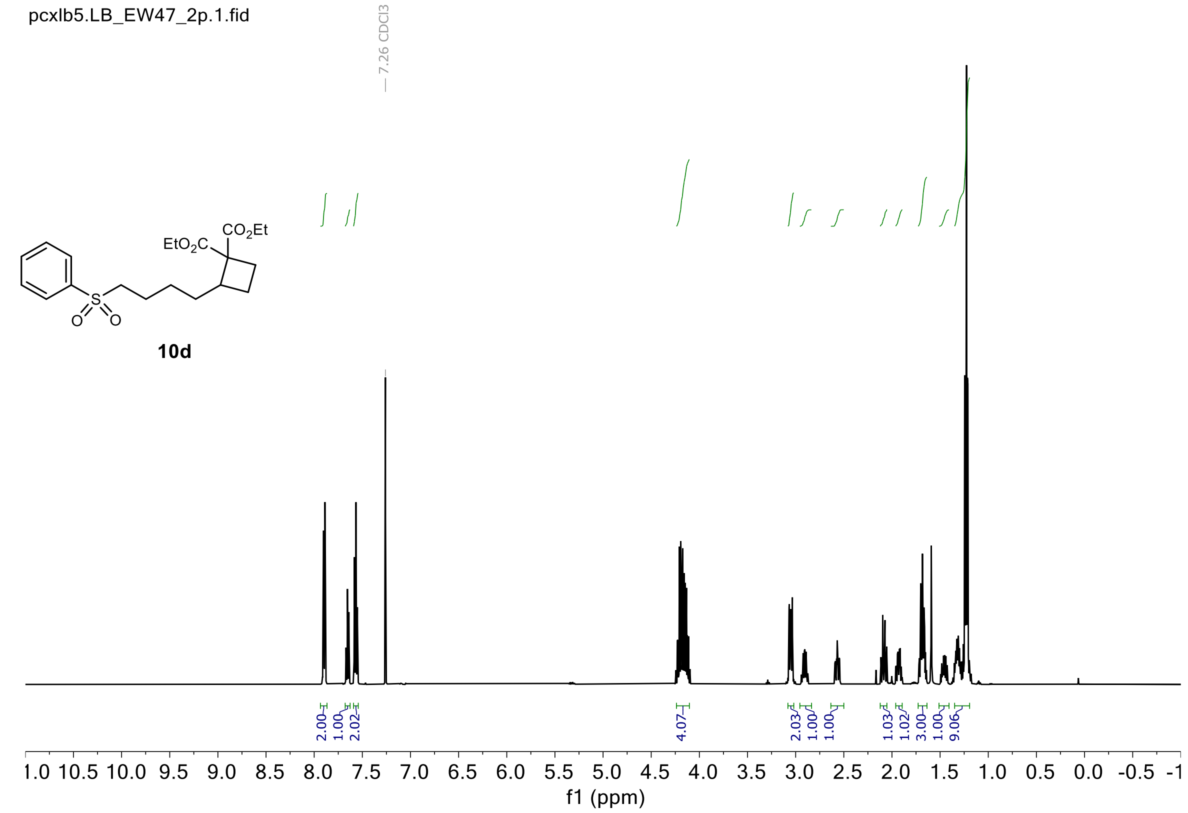

^{13}C-NMR ($CDCl_3$, 126 MHz)

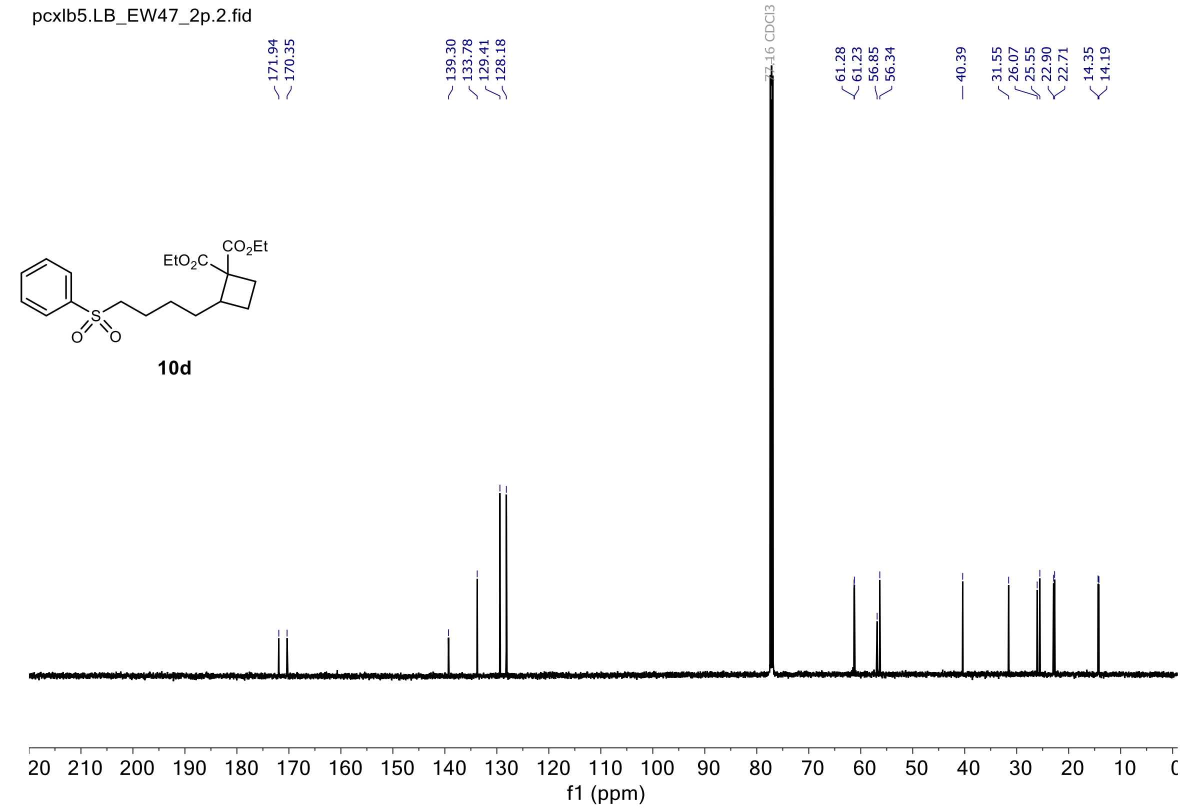

diethyl 2-(4-(benzyloxy)butyl)cyclobutane-1,1-dicarboxylate (10e)

^{1}H-NMR ($CDCl_3$, 500 MHz)

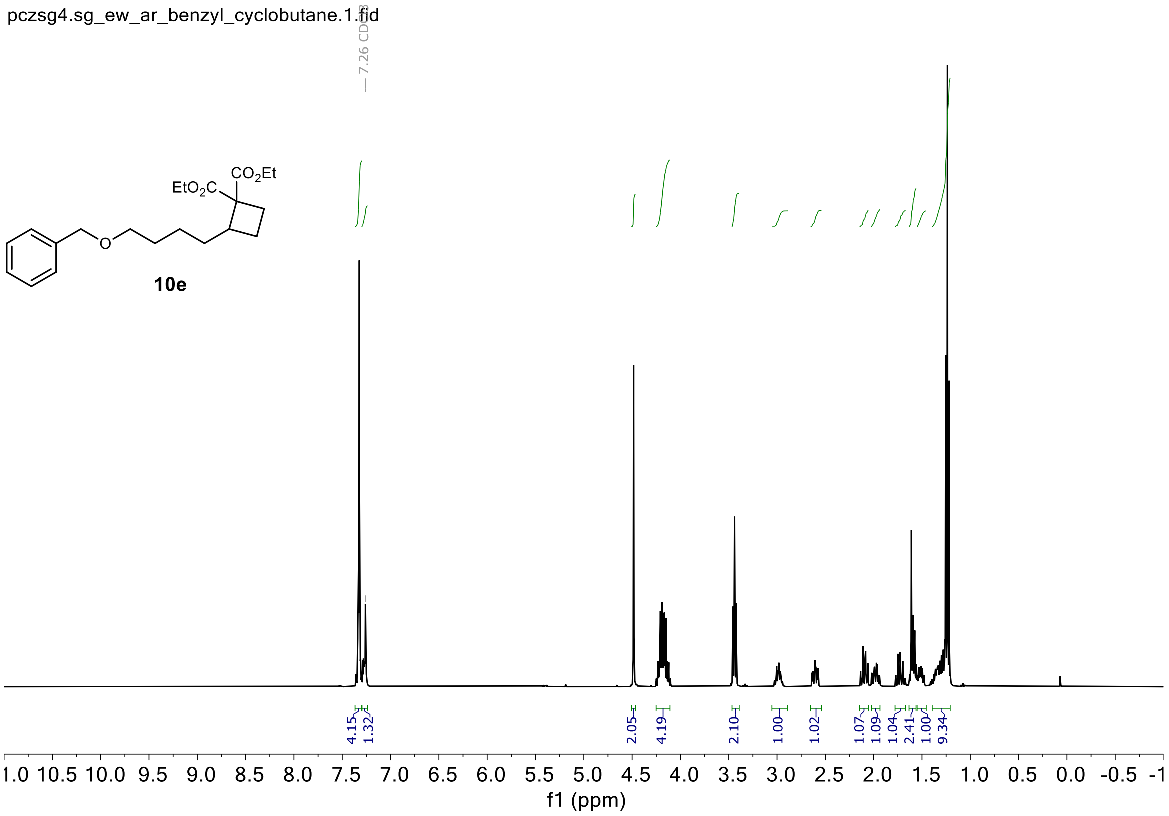

^{13}C-NMR ($CDCl_3$, 126 MHz)

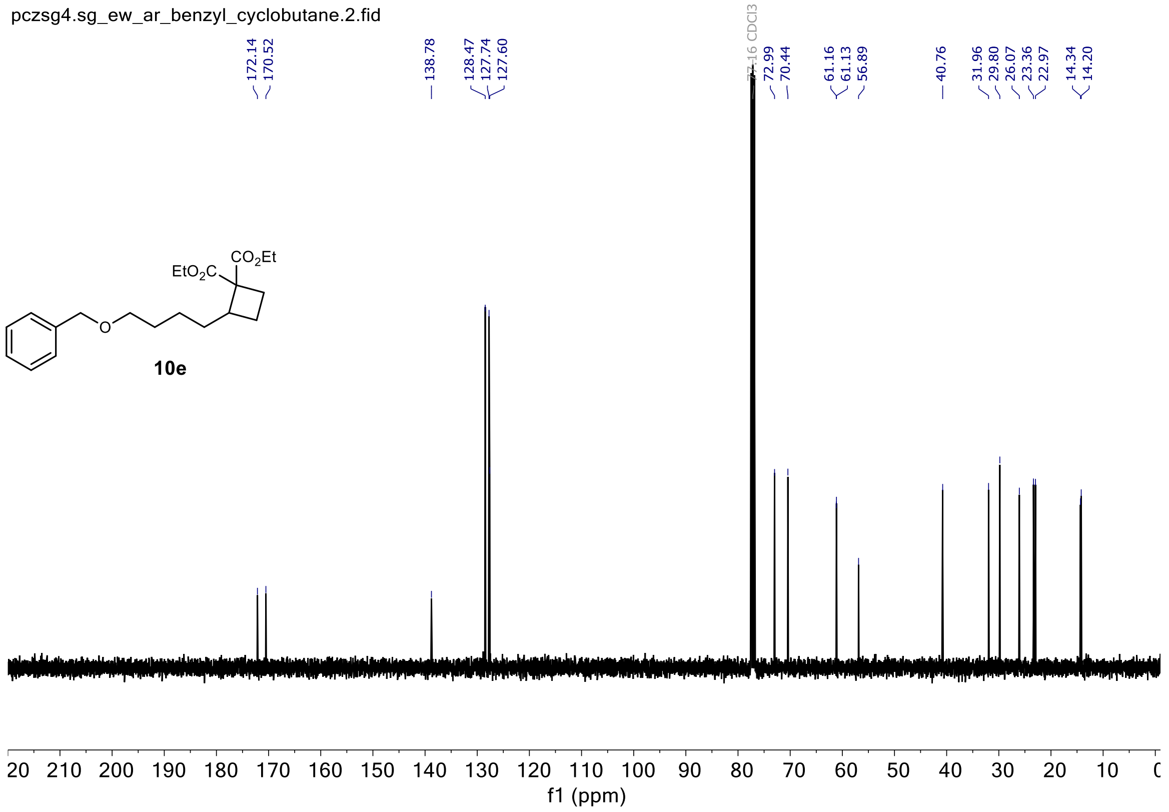

2-phenethylcyclobutane-1,1-dicarbonitrile (10f)

^{1}H-NMR (CDCl$_3$, 500 MHz)

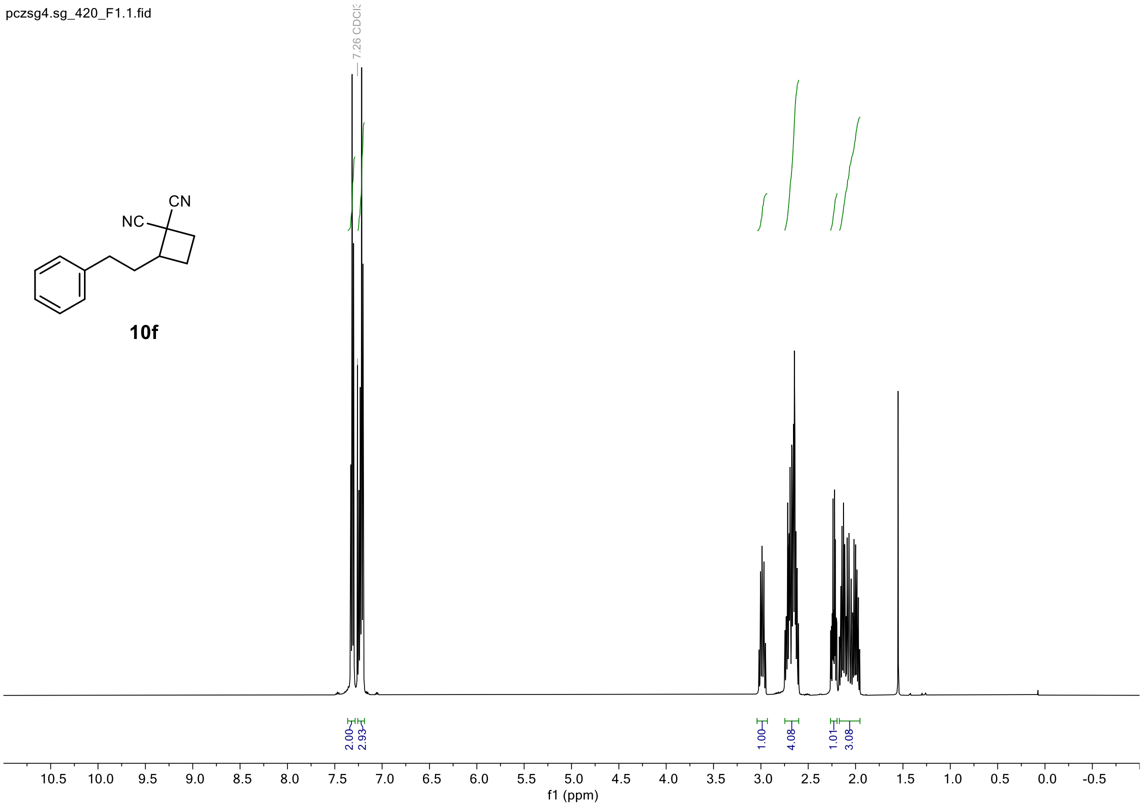

^{13}C-NMR (CDCl$_3$, 126 MHz)

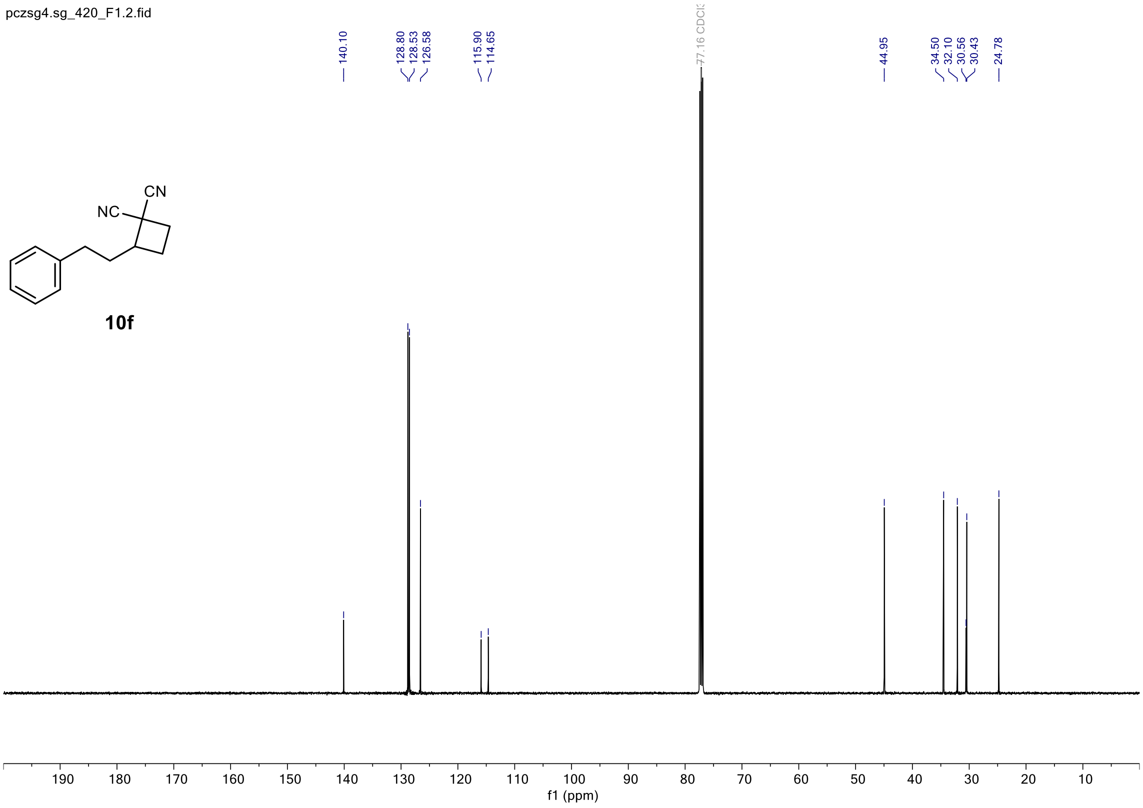

ethyl 1-cyano-2-phenethylcyclobutane-1-carboxylate (10g)

^{1}H-NMR ($CDCl_3$, 500 MHz)

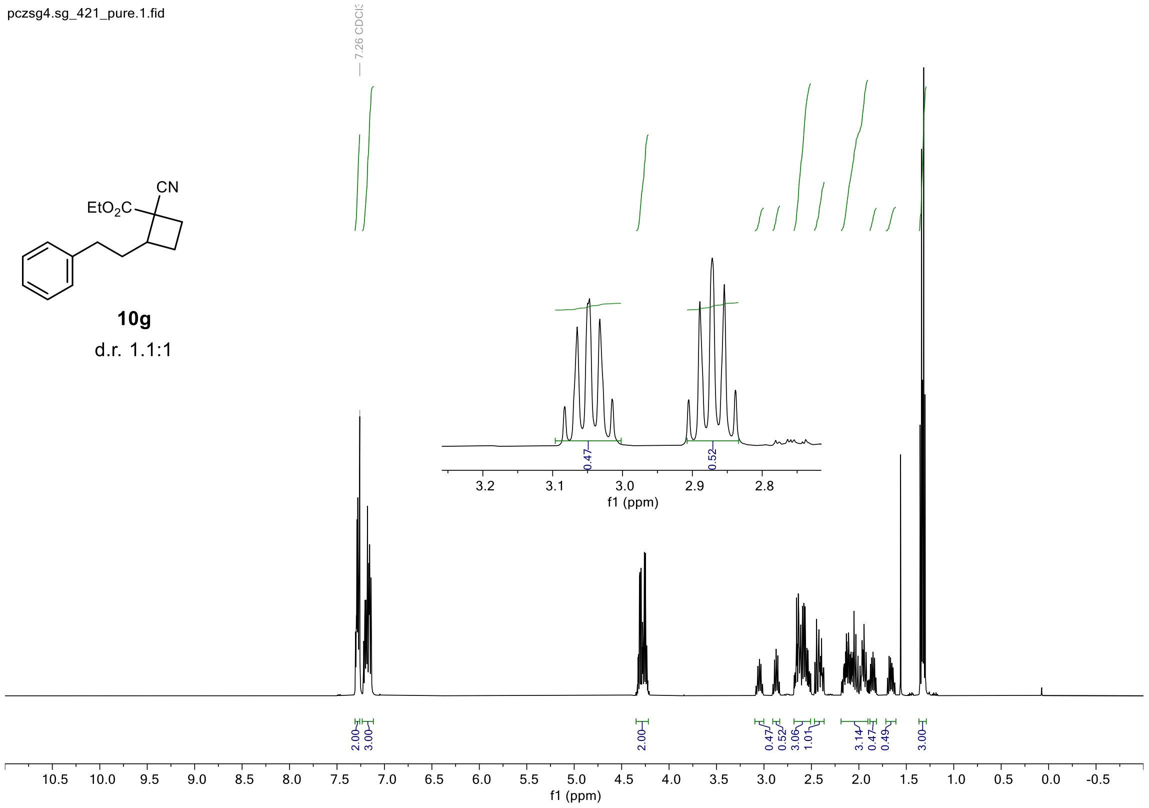

^{13}C-NMR ($CDCl_3$, 126 MHz)

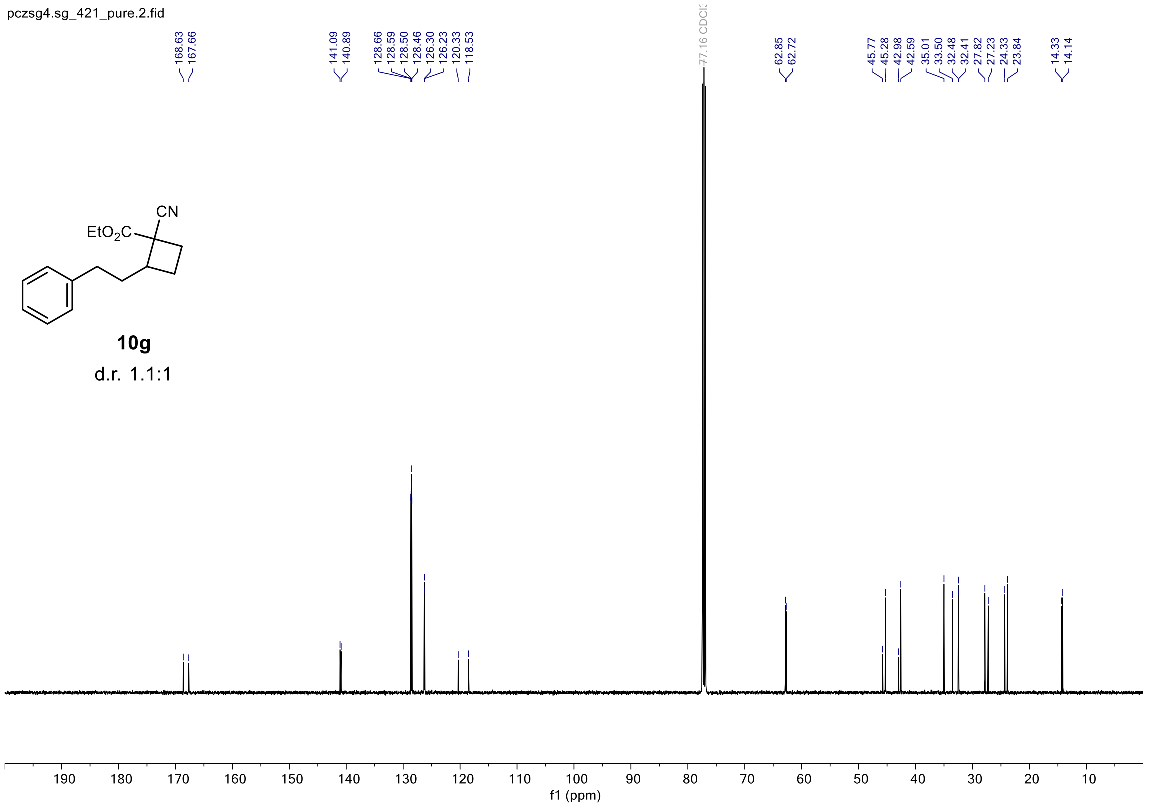

(2-phenethylcyclobutane-1,1-disulfonyl)dibenzene (10h)

^{1}H-NMR ($CDCl_3$, 500 MHz)

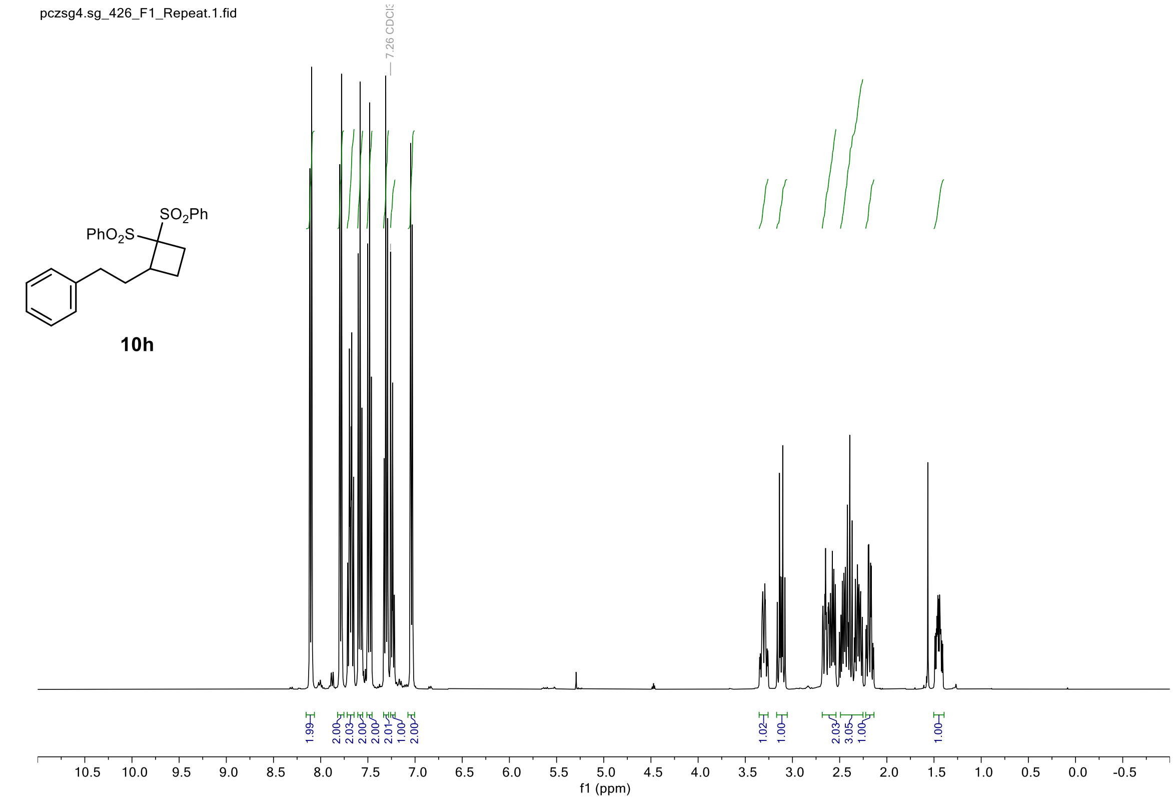

^{13}C-NMR ($CDCl_3$, 126 MHz)

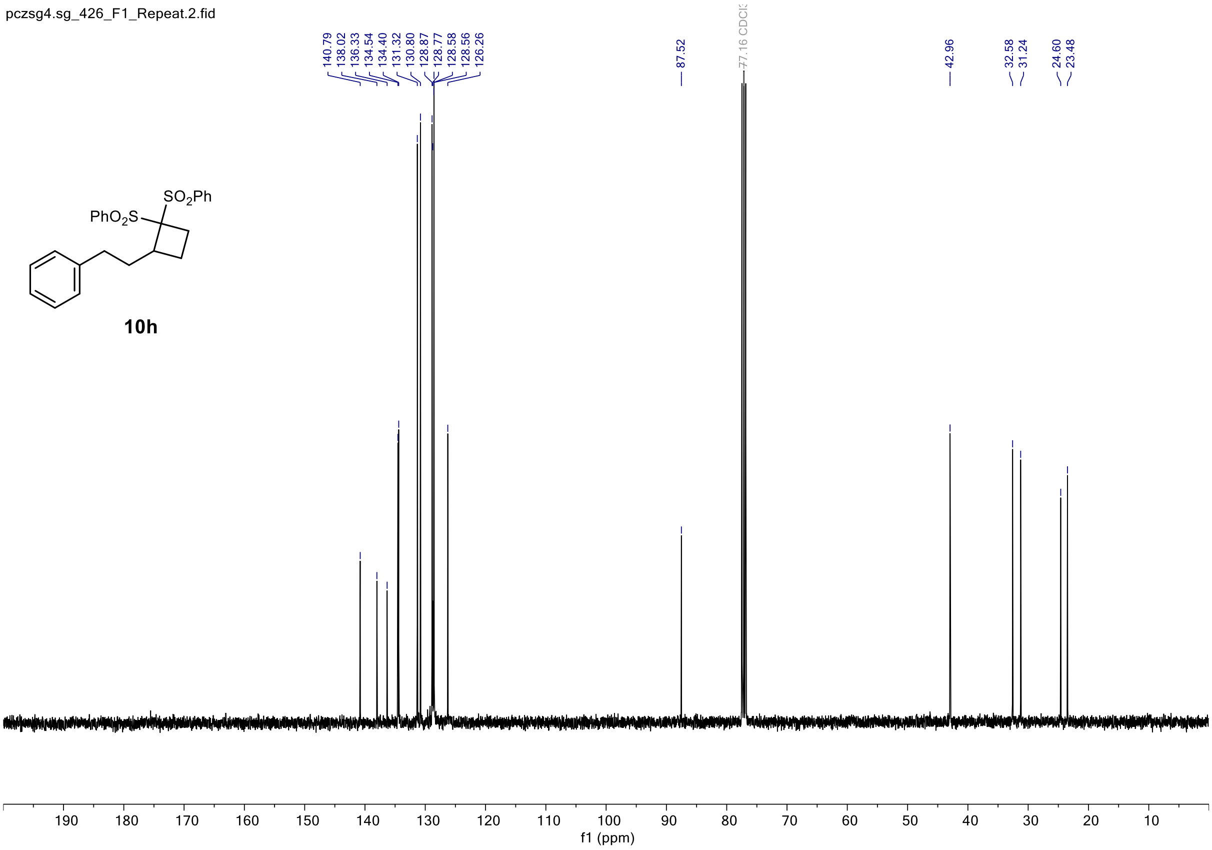

2-phenethyl-1'-phenylspiro[cyclobutane-1,3'-indolin]-2'-one (10i)

^{1}H-NMR ($CDCl_3$, 500 MHz)

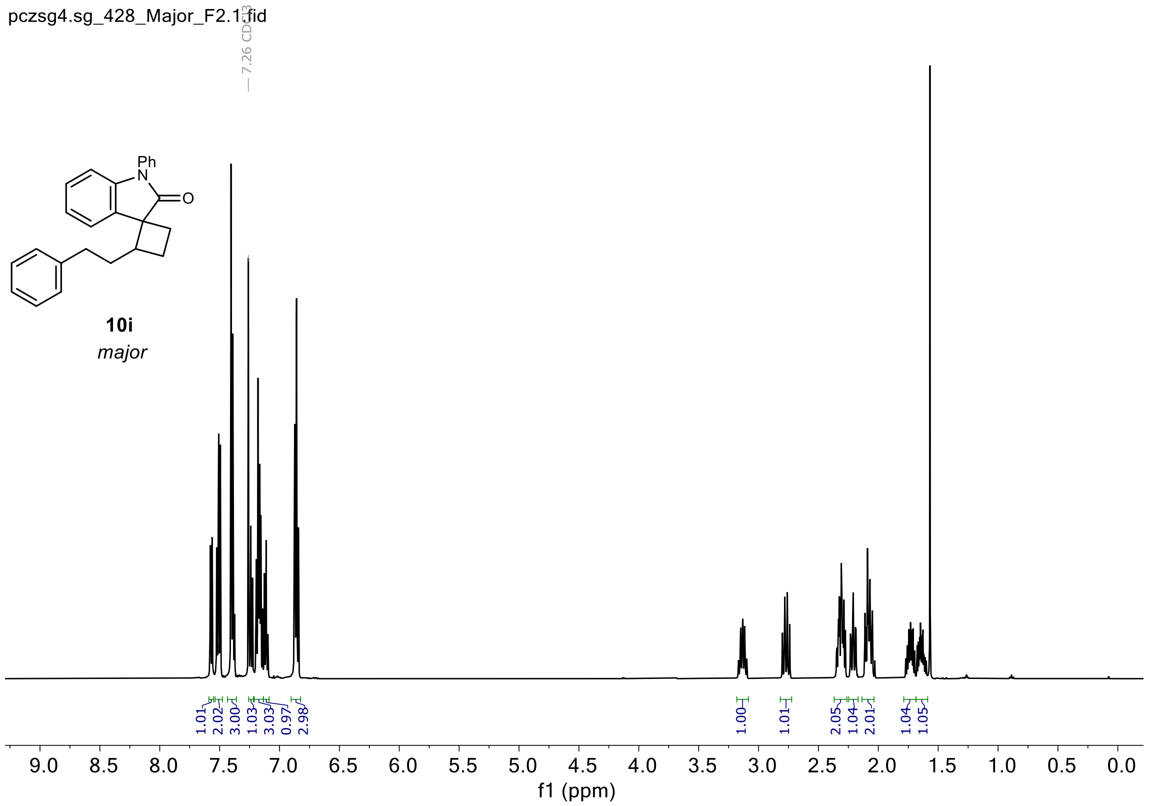

^{13}C-NMR ($CDCl_3$, 126 MHz)

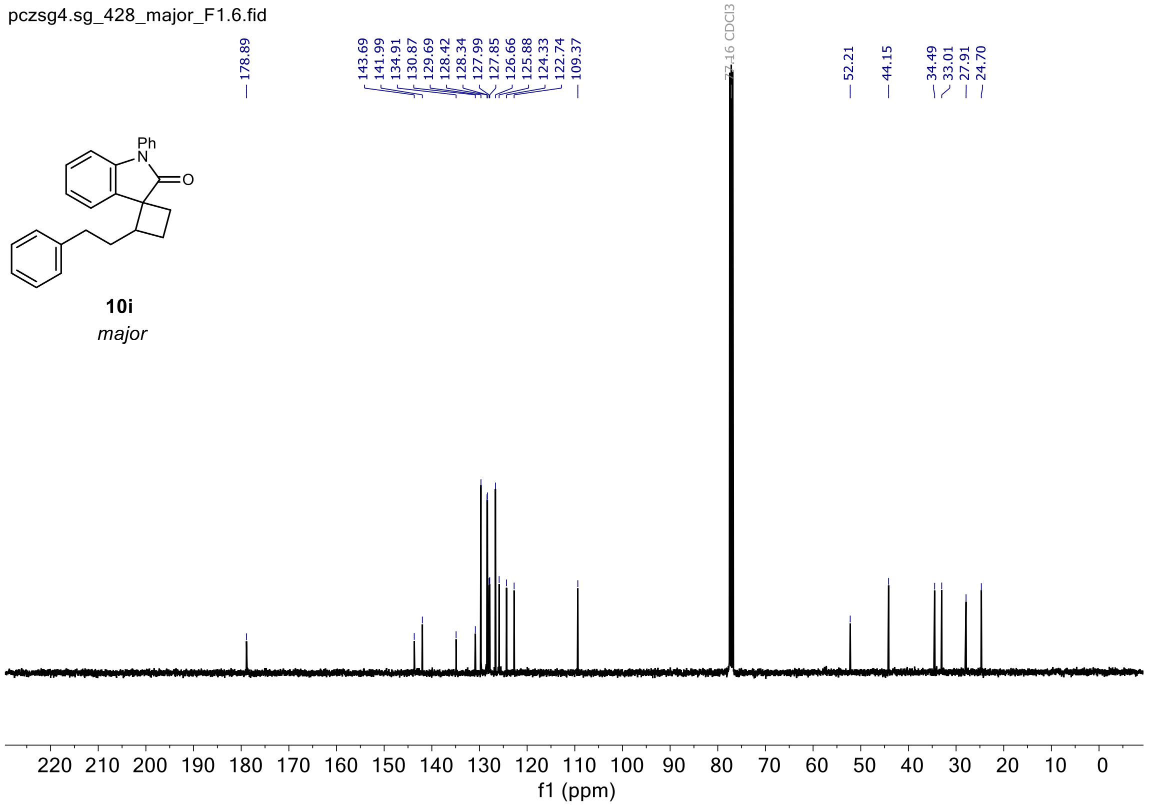

^{1}H-NMR ($CDCl_3$, 500 MHz)

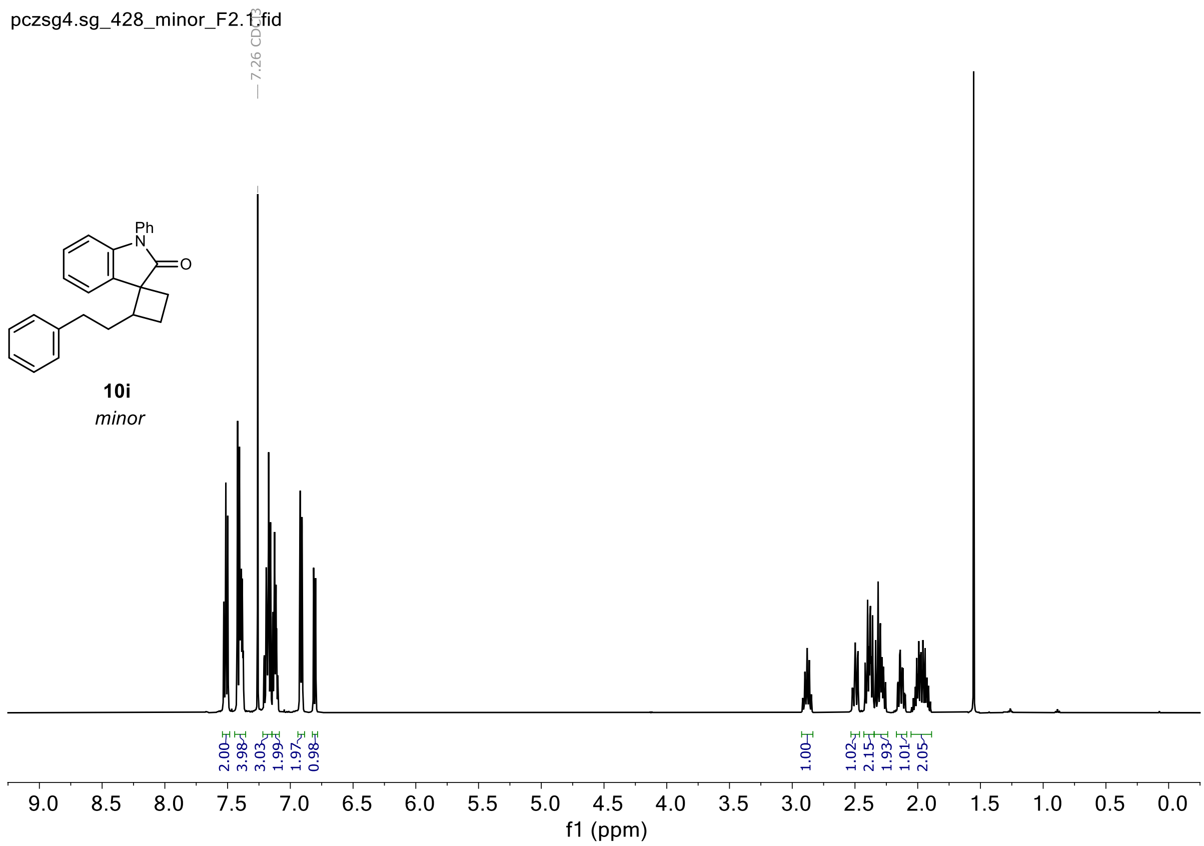

^{13}C-NMR ($CDCl_3$, 126 MHz)

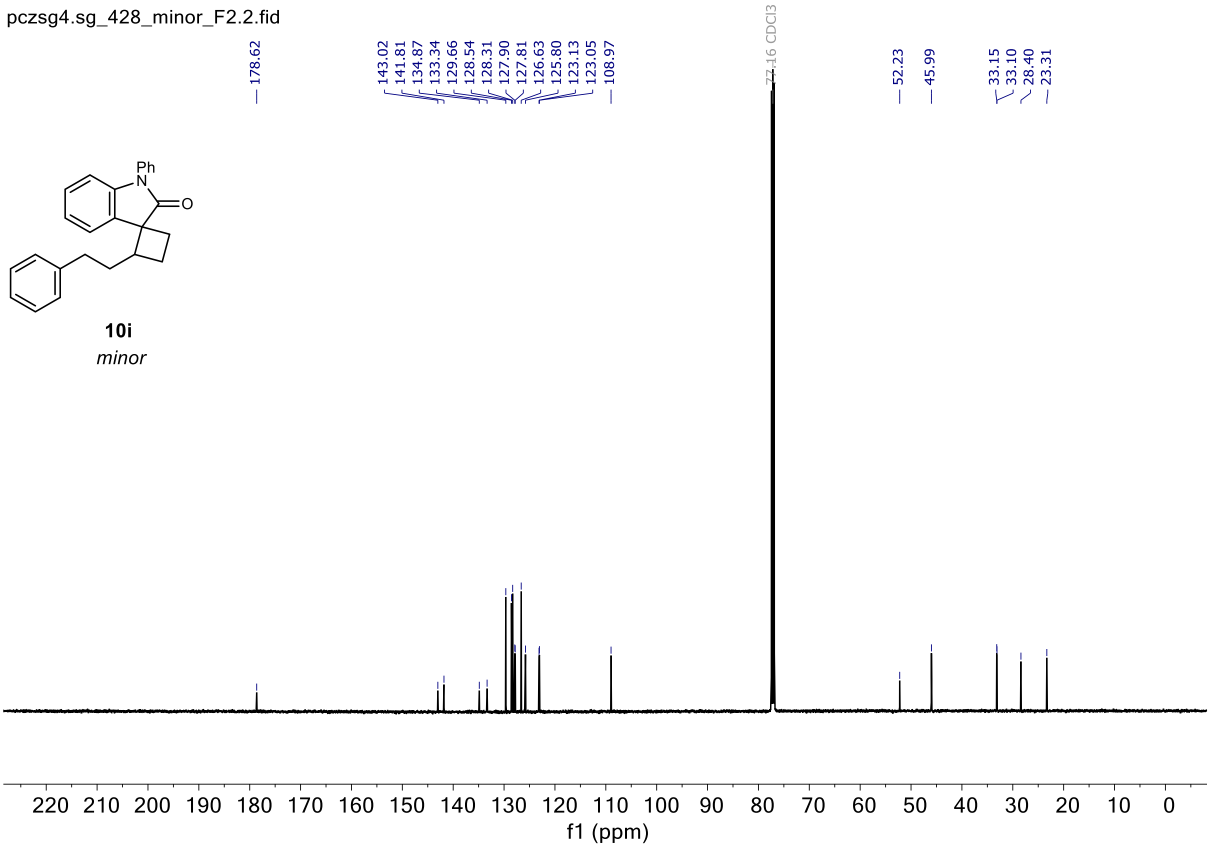

2-phenethylspiro[cyclobutane-1,4'-isochroman]-3'-one (10j)

^{1}H-NMR ($CDCl_3$, 500 MHz)

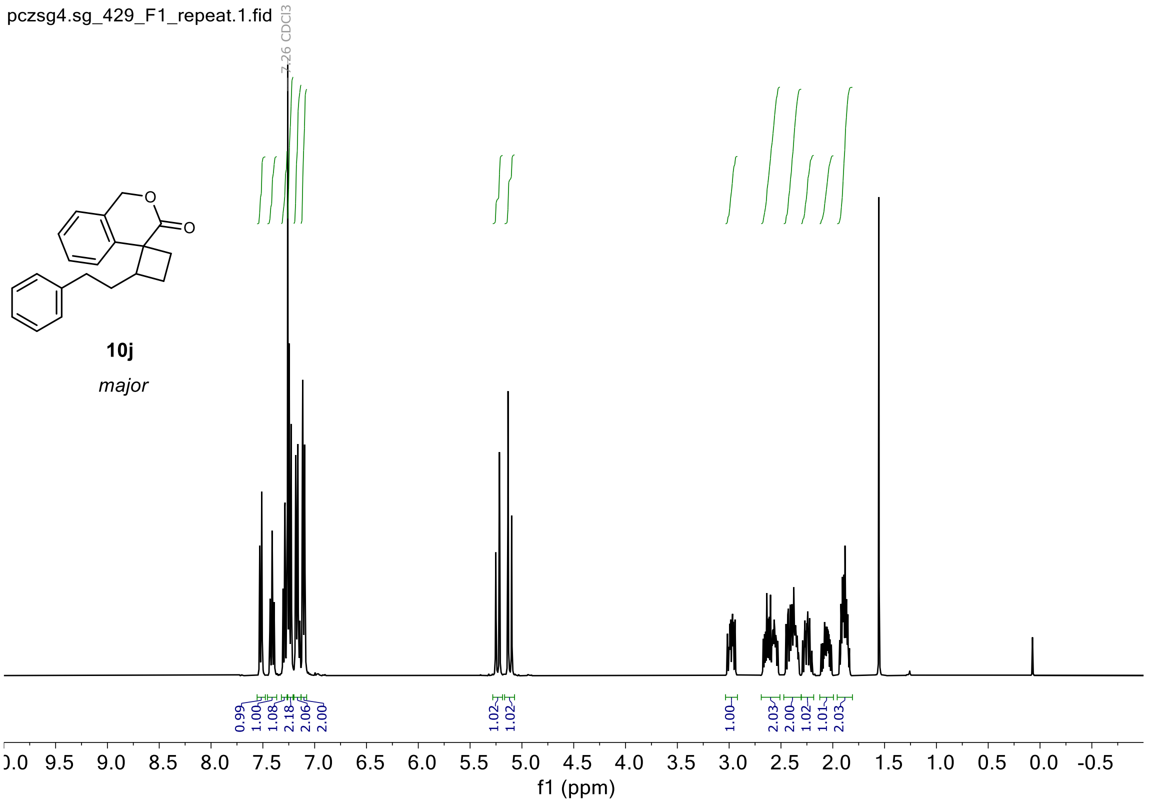

^{13}C-NMR ($CDCl_3$, 126 MHz)

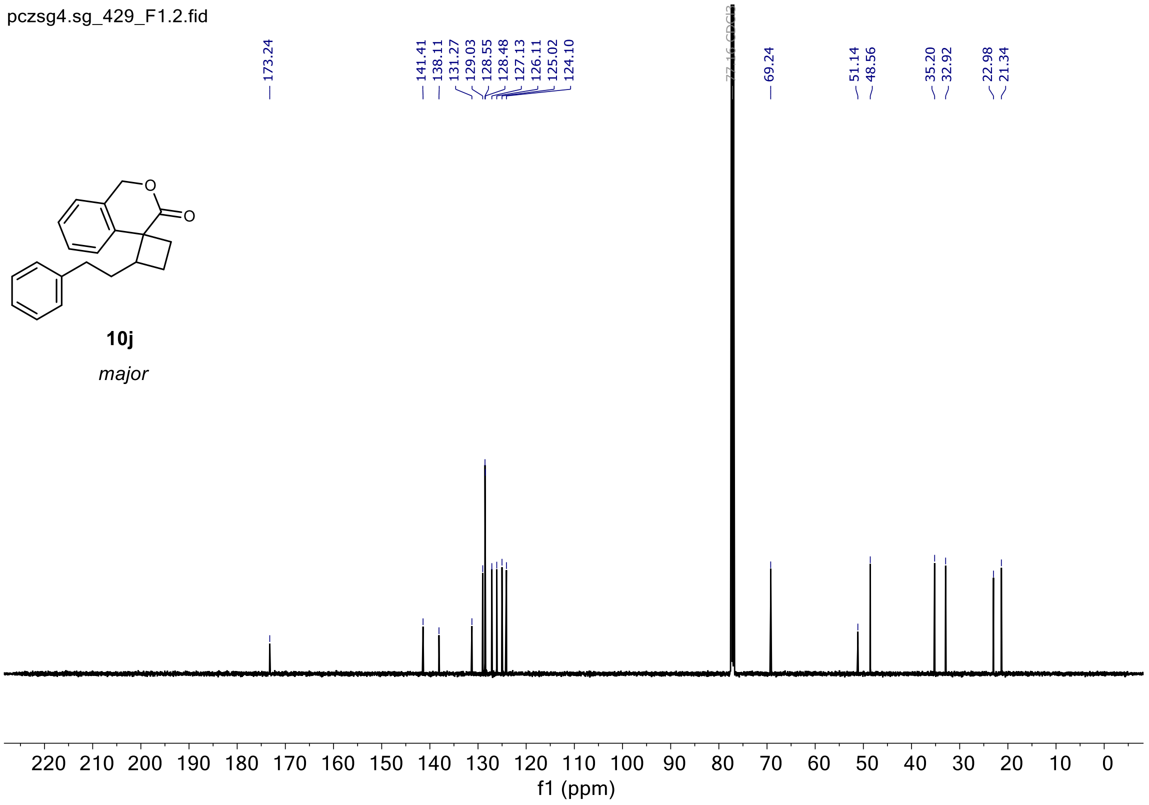

^{1}H-NMR ($CDCl_3$, 500 MHz)

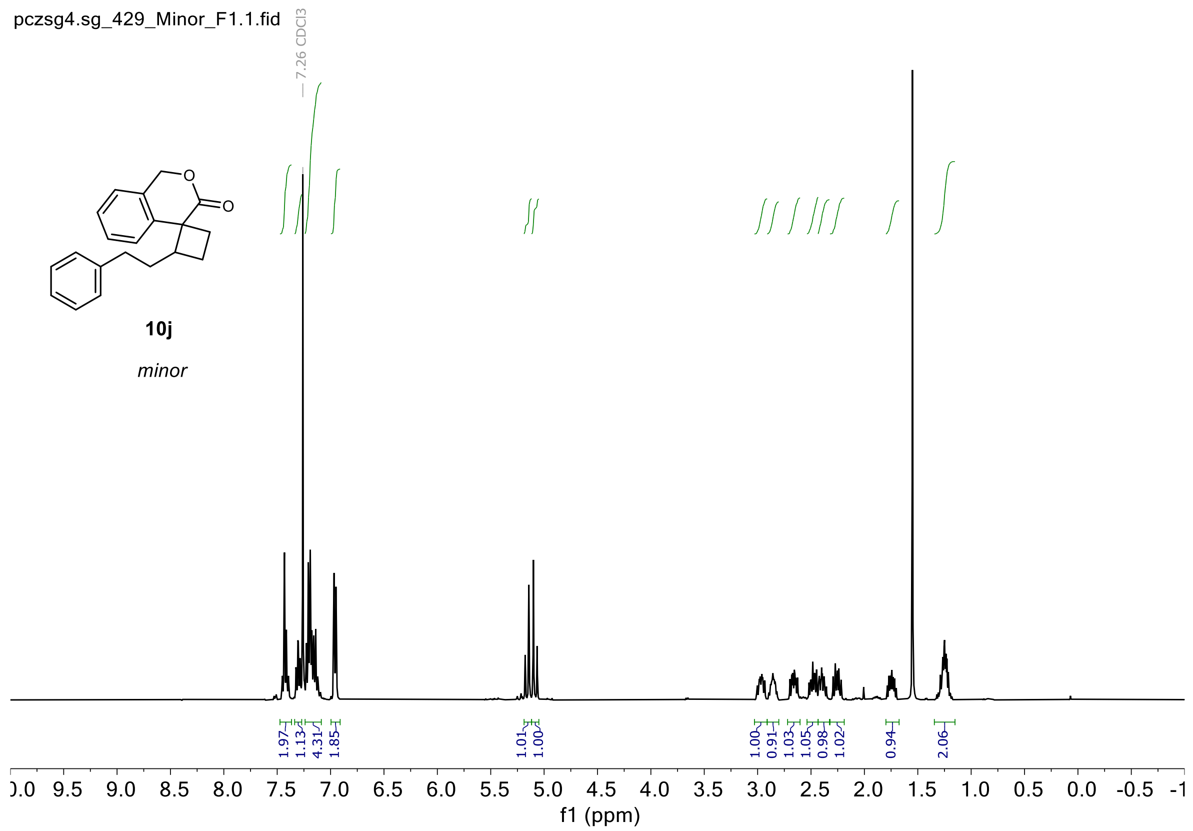

^{13}C-NMR ($CDCl_3$, 126 MHz)

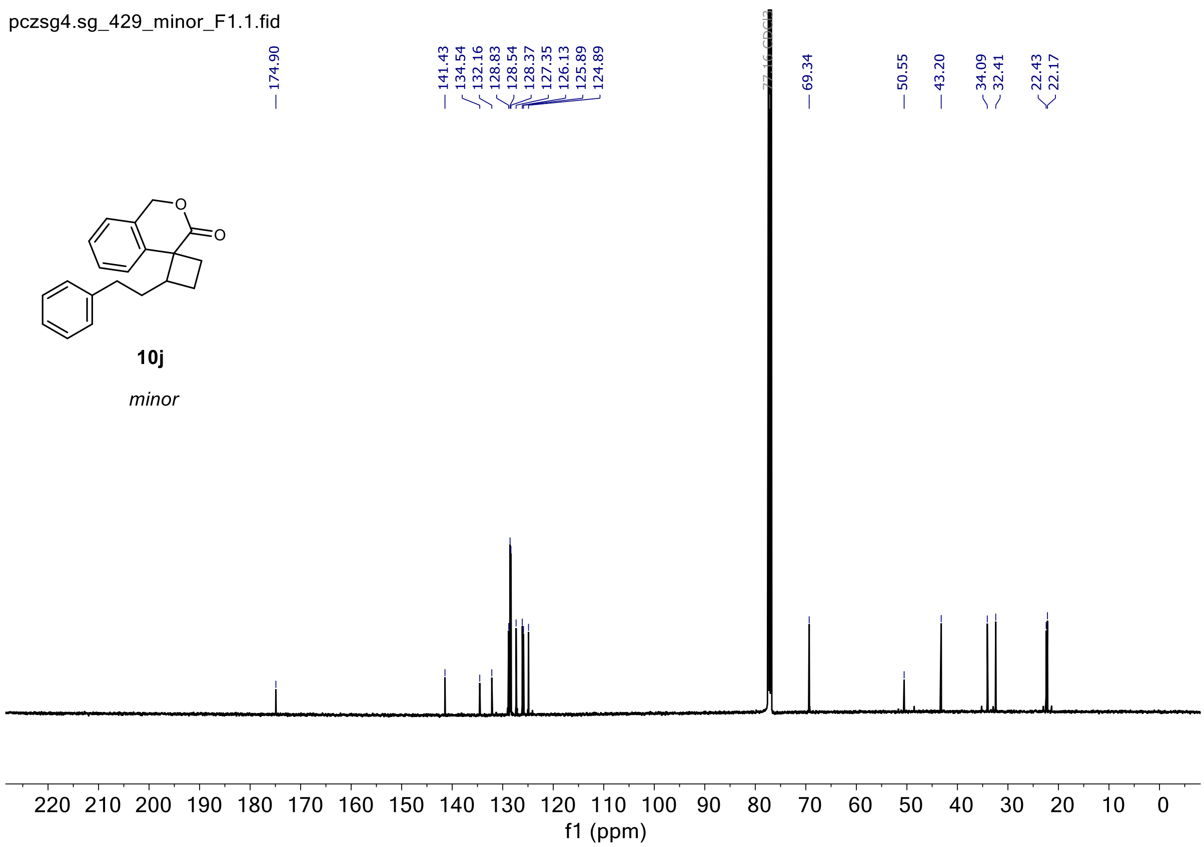

ethyl 2-phenethylcyclobutane-1-carboxylate (10k)

^{1}H-NMR ($CDCl_3$, 500 MHz)

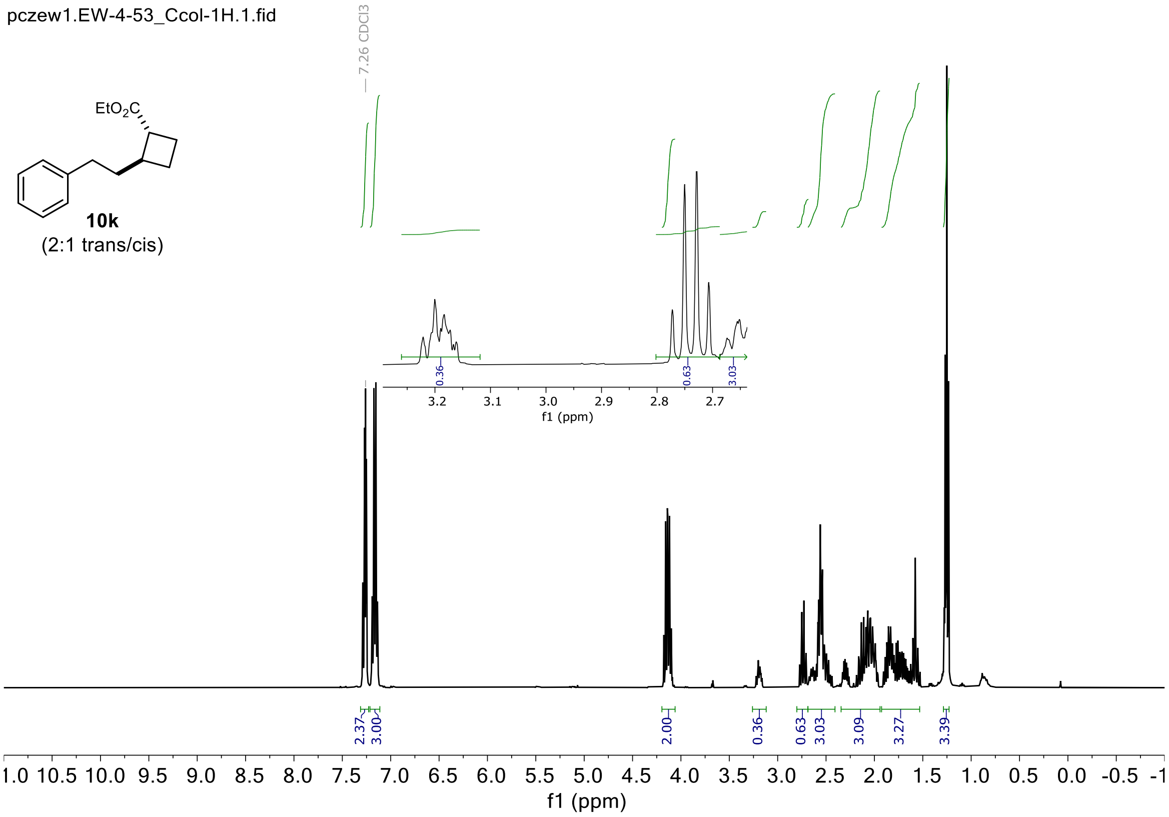

^{13}C-NMR ($CDCl_3$, 126 MHz)

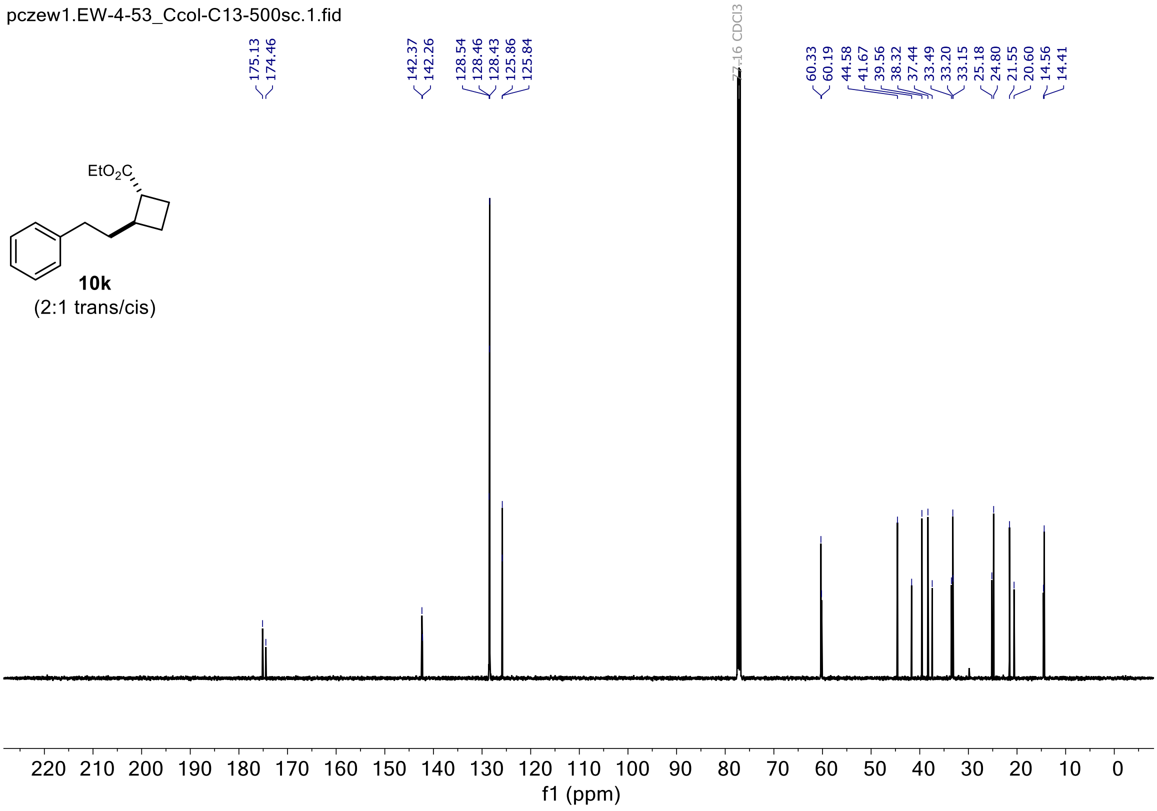

Supplement: Supplementary file 1 [file ja5c11758_si_001.pdf]
